# Targeting Shikimate Kinase Pathway of *Acinetobacter baumannii*: A Structure-Based Computational Approach to Identify Antibacterial Compounds

**DOI:** 10.1155/2023/6360187

**Published:** 2023-03-29

**Authors:** Aparna Shil, Most. Afrin Akter, Arafin Sultana, Sajal Kumar Halder, Mahbubul Kabir Himel

**Affiliations:** ^1^Department of Botany, Jahangirnagar University, Savar, Dhaka 1342, Bangladesh; ^2^Padma Bioresearch, Dhaka, Bangladesh; ^3^Department of Pharmacy, Jahangirnagar University, Savar, Dhaka 1342, Bangladesh; ^4^Department of Biochemistry and Molecular Biology, Jahangirnagar University, Savar, Dhaka 1342, Bangladesh

## Abstract

*Acinetobacter baumannii* (*A. baumannii*) is an opportunistic bacterium that has developed multidrug resistance (MDR) to most of today's antibiotics, posing a significant risk to human health. Considering the fact that developing novel drugs is a time-consuming and expensive procedure, this research focuses on utilizing computational resources for repurposing antibacterial agents for *A. baumannii*. We targeted shikimate kinase, an essential enzyme in *A. baumannii*, that plays a significant role in the metabolic process. The basis for generating new therapeutic compounds is to inhibit the shikimate kinase and thereby targeting the shikimate pathway. Herein, 1941 drug-like compounds were investigated in different *in silico* techniques for assessing drug-likeness properties, ADMET (absorption, distribution, metabolism, excretion, and toxicity) profiling, binding affinity, and conformation analysis utilizing Autodock-vina and SwissDock. CHEMBL1237, CHEMBL1237119, CHEMBL2018096, and CHEMBL39167178 were determined as potential drug candidates for suppressing shikimate kinase protein. Molecular Dynamics Simulation (MDS) results for root mean square deviation, root mean square fluctuation, hydrogen bond, and gyration radius confirm the drug candidates' molecular stability with the target protein. According to this study, CHEMBL1237 (Lisinopril) could be the most suitable candidate for *A. baumannii*. Our investigation suggests that the inhibitors of shikimate kinase could represent promising treatment options for *A. baumannii*. However, further *in vitro* and *in vivo* studies are necessary to validate the therapeutic potential of the suggested drug candidates.

## 1. Introduction

Resistance to antibiotics is currently a global concern to public health that causes trouble in disease management, infection control, duration of treatment, and patient care, thereby increasing the cost of healthcare. Antibiotic activity towards organisms worsens over time because of multidrug resistance (MDR), which increases because of the misuse and overuse of antibiotics, poor ailment managements, and the evading characteristic of microorganisms [1, 2]. *Acinetobacter baumannii* also has an extraordinary ability to acquire or upregulate resistance determinants representing it as one of the organisms that pose threats to present antibiotics [3, 4]. *Acinetobacter baumannii* is a Gram-negative, nonmotile, obligate aerobic, oxidase-negative, catalase-positive, and nonfermentative coccobacillus that harbors a number of successful virulence factors which enable its multidrug resistance phenomenon [5]. Species *A. baumannii*, which predominantly causes nosocomial infections, belongs to the Moraxellaceae family [6] and is responsible for other health issues such as hospital-acquired pneumonia (HAP), ventilator-associated pneumonia (VAP), septicaemias, endocarditis, secondary meningitis, and various infections of skins, soft tissues, and urinary tracts [7]. The worldwide incidence of multidrug-resistant *A. baumannii* strains in hospital-associated pneumonia and ventilator-associated pneumonia patients is 79.9% and ranges from 56.5% in *Argentina* and 61.8% in Taiwan to 100% (79.9%) in Central America, Pakistan, Lebanon, Qatar, and Croatia, and the overall mortality rate can reach 56.2% [7].

The ability of *Acinetobacter baumannii* to acquire MDR has drawn a lot of interest in our study [8]. The WHO ranked carbapenem-resistant *A. baumannii* as the world's leading priority organism for research and development of antibiotics in 2018; since resistance to carbapenem often denotes a broad spectrum of resistance to other classes of available antibiotics, the drug is listed as a marker [9]. Alongside its incredible resistance abilities, the organism can survive in the hospital environment for a prolonged period, enhancing its capacity to cause various infections [9]. In addition, although *A. baumannii* infections are so important, effective therapeutic options to combat them are limited, which impose a significant burden on the world healthcare system. Furthermore, the multidrug resistance ability of the organism posed a problem in controlling infections [10]. The bacteria have already demonstrated resistance to all antibiotics, including the last-resort antibiotics, such as carbapenem, demanding the attention of the healthcare community for the necessity of new drug development that will fight against the organism's various weapons. As a result, there is a pressing need for novel drug-like molecules as treatment options against multidrug-resistant* A. baumannii*.

Since the shikimate pathway is necessary for the survival of algae, vascular plants, fungus, microbes, however not present in humans, it represents a promising new source for the synthesis of antibacterial drugs and herbicides [1]. Chorismic chemical is synthesized by bacteria through the shikimate pathway from which many amino acid precursors are derived. These include anthranilate (a source of tryptophan), prephenate (a source of phenylalanine and tyrosine), para-hydroxybenzo (shared source of the compounds: mycobactins, menaquinones, and naphthoquinones), and aminodeoxychorismate (a source of para-aminobenzoic acid) [1]. The shikimate kinase is located at the top of chorismate synthase and 5-enol-pyruvyl-shikimate3-phosphate, and it facilitates the fifth phase in the passageway, which is the conversion of shikimate to shikimate 3-phosphate utilising ATP as a cosubstrate [2]. It is conceivable to construct a multitarget antibacterial drugs potent against multiple enzymes in the cascade because the substrates of these enzymes have a homologous scaffold. Implementing method will substantially reduce on the possibility of the development of a resistant strain [2]. There are 3 separate regions inside shikimate kinase: the CORE motif, that comprises amino acids from the preserved binding loop (P-loop) that constitutes the binding pocket of ADP and ATP; the LID area, that shuts over the catalytic site and bears essential sequences for ATP interaction; and the NMP-binding area, which correlates to the shikimate binding area [7]. Our proposed ligands have the ability to bind to any of the binding pockets and elicit competitive inhibition. This procedure causes an interruption inside the enzyme's binding pockets.

Many studies have suggested that phytocompounds are the best alternative for generating therapies for multidrug-resistant bacterial infections [11] although synthetic chemicals are also used to develop novel treatment. Therefore, the available plant-originated and chemical compounds in the databases can be screened for their compatibility to develop drugs to fight the multidrug-resistant *A. baumannii*. The process of developing a new drug is both time-consuming and costly. Nowadays, computer-aided drug innovation technique is frequently used to investigate phytochemicals against pathogens such as *A. baumannii*. Therefore, the computational approaches are of great importance for the development of new drugs against *A. baumannii*. The binding mechanism between the ligand and the target protein could be determined by employing docking techniques to screen phytocompound databases. The findings from our current study can identify lead molecules and also indicate required mechanism of the lead drug-like molecules, thereby shortening the length of the drug discovery process and cost. The process of discovering drugs involves evaluating potential compounds for drug development and identifying potential targets to be inhibited to improve disease prognosis. To combat antimicrobial resistance, a combination strategy of medicinal chemistry and bioinformatics was utilized to identify possible targets and candidate drug-like compounds [12]. The conformational stability of targeted protein-ligand complexes was evaluated by molecular dynamics simulation at 100 nanoseconds. This suggests a prospective antibacterial molecule against *A. baumannii* and demonstrates the interaction of present small drug-like compounds implicated in antibacterial activities.

Our present findings suggest potential drug-like compounds with pharmacological possibilities that can be used to start a new drug development work. To assess the effectiveness of the compounds and develop new therapeutic medicines against *A. baumannii*, additional *in vitro* and *in vivo* investigation is required. Before these compounds could be used in treatments, however, more research into their therapeutic potential and safety is needed. Here, we found CHEMBL1237 (Lisinopril) as a putative drug candidate against *A. baumannii* after screening their compatible drug-like properties and pharmacokinetics characteristics, evaluating binding interaction and molecular stability of lisinopril and shikimate kinase protein.

## 2. Materials and Methods

A total of 1,941 chemical compounds and phytochemicals were retrieved from NPASS (https://bidd.group/NPASS/) and PubChem (https://pubchem.ncbi.nlm.nih.gov/) data sources. The compounds were considered based on previous research that showed they could be used to treat a variety of human pathogenic viruses [12]. Doxycycline was also employed as a control drug in this investigation.

### 2.1. Identification of Drug-Like Properties

The drug-like characteristics represent the potential physicochemical properties of a small molecule. The QikProp module of Schrödinger software was used to predict the drug-like properties [13]. Canonical smiles of the ligands were collected from PubChem (https://pubchem.ncbi.nlm.nih.gov/) database. The Lipinski's rule of five was used to evaluate the drug-likeness of the compounds [13]. Here, we submitted 1,941 small compounds for initial screening in the QikProp module.

### 2.2. Identification of ADMET Properties by pkCSM Server

To predict the ADMET data of small molecules, pkCSM online tool (https://structure.bioc.cam.ac.uk/pkcsm) was utilized. It incorporates graph-based structural signatures with an optimum threshold value to filter drug-like compounds. The following parameters such as water solubility, Caco-2 permeability, intestinal absorption (human), P-gp substrate, P gp-I inhibitor, P gp-II inhibitor, BBB permeability, CYP2D6 substrate, CYP3A4 substrate, CYP1A2 inhibitor, CYP2C19 inhibitor, CYP2C9 inhibitor, CYP2D6 inhibitor, CYP2C3A4 inhibitor, AMES toxicity, hERG I inhibitor, hERG II inhibitor, and hepatotoxicity were considered for predicting ADMET properties. After initial screening in the QikProp setup, we found 1,065 small compounds and utilized them in ADMET evaluation.

### 2.3. Molecular Docking by Autodock-Vina

The ligands were downloaded from PubChem (https://pubchem.ncbi.nlm.nih.gov/) database. These ligands were energy minimized and converted to Autodock-vina supported pdbqt format by an open babel module of PyRx software [14]. The 3D structure of the shikimate kinase from *A. baumannii* in complex with shikimate (PDB ID: 4Y0A) was retrieved from Protein Data Bank (https://www.rcsb.org/). PyMOL was utilized to eliminate water and other hetero molecules from the crystal structure of the protein [13]. Swiss-PdbViewer software was employed to energy minimize the protein [15]. Subsequently, the protein was loaded on the Autodock-vina to incorporate polar hydrogen bonds to the protein and convert them from pdb to Pdbqt format. The active site of the protein was identified using CASTp 3.0 (https://sts.bioe.uic.edu/castp/) server. Based on the information of the binding pocket, the center of the grid box for the shikimate kinase (PDB ID: 4Y0A) was fixed where *X* = 3.924, *Y* = 9.759, and *Z* = 18.945 with a dimension of 62 × 58 × 66 Å. One hundred and twenty-five compounds were screened in the Autodock-vina to find the best binding interaction between ligand and protein [16].

### 2.4. Molecular Docking by Swissdock Server

The SwissDock server provides a simple and user-friendly GUI for analyzing a protein-ligand docking [17]. The ligands were converted to MOL2 format by Avogadro software for the Swissdock server. Out of 125, a total of 36 small molecules had the highest binding affinity with increased nonbonded interactions, and they were submitted to the server for docking analysis. The server uses full-fitness and estimated Δ*G* value to represent the binding interaction.

### 2.5. Molecular Dynamics Simulation by GROMACS

MDS (molecular dynamics simulation) is a thermodynamics-based method that aids the investigation of dynamic fluctuations in protein-ligand complexes. The best ligands from the earlier phases were put through molecular dynamics simulation (MDS) with their respective proteins. To simulate protein-ligand conformations, the GROMACS (https://simlab.uams.edu/) service was used, and the GROMOS96 43a1 force field was used to produce topological data for the complex structures [18]. The GROMACS (https://simlab.uams.edu/) software was used to simulate protein-ligand conformations, and the GROMOS96 43a1 force field was used to create the complex topological data [19]. To render ligand topology and coordinate information, the PRODRG (https://davapc1.bioch.dundee.ac.uk/cgi-bin/prodrg) server was applied [20]. The SPC water model (simple point-charge) was used to construct the aqueous phase of macromolecules, which was then neutralized with a 0.15 M NaCl solution [8]. The biomolecular environment was kept in a triclinic box, and the energy was reduced using the 5000 steepest decline stages. At 310 K and 1.0 bar, the NPT (constant pressure) and NVT (constant volume) setups were used to achieve ion-molecule equilibrium around the macromolecule. It generated simulated structural dynamics, including the root mean square deviation (RMSD), root mean square fluctuation (RMSF), radius of gyration (Rg), solvent-accessible surface area (SASA), and hydrogen bonds (HBs) at 100 nanoseconds of simulation.

### 2.6. Molecular Dynamics Simulation by Desmond

We utilized MD simulation in Desmond to further analyze the binding stability of the Shikimate kinase_CHEMBL1237, Shikimate kinase_CHEMBL1237119, Shikimate kinase_CHEMBL2018096, Shikimate kinase_CHEMBL3916717, and Shikimate kinase_Doxycycline complexes [21]. These complex structures were solvated employing the system designer tool on the cubic TIP3P simulation (3-point water model). The solvated region was at least 10 Å distant from the protein-ligand structure. Afterwards, Na+ and Cl− charged ions were provided to the resulting model to normalize subsequently, bringing it to the physiological salt content of 0.15 M. The integrated OPLS3e force field was applied to optimize the energy of the solvated complex structures. At 310 K and 1.013 bar, the MDS was conducted utilizing isothermal isobaric composition (NPT). This was a 100 ns simulation period and a 100 ps capturing interval during which 1000 frames were stored in the trajectory's memory. Eventually, we analyzed the trajectory with the help of the simulation interaction diagram (SID) tool, and the extracted findings comprised protein contact mapping, RMSD, RMSF, and RMSD, for the ligand. The Shikimate kinase_ CHEMBL1237, Shikimate kinase_CHEMBL2018096, Shikimate kinase_CHEMBL3916717, and Shikimate kinase_Doxycycline complexes were subjected to a postsimulation MM-GBSA assessment utilising thermal MM-GBSA.py package. Binding-free energy was calculated after MM-GBSA analysis, yielding a range of 0–1000 [8].

## 3. Results

### 3.1. Analysis of Drug-Like Properties

Lipinski's rule of five filtration technique incorporates the following parameters: molecular weight (recommended range: <500), the number of hydrogen bond donors (ideal range: ≤5), the number of hydrogen bond acceptors (standard range: ≤10), and lipophilicity (represented as LogP, normal range: <5). Out of 1,941 small molecules, 1,065 of them showed optimal drug-like properties based on Lipinski's rule of five rule (Supplementary table 1: Drug-like properties of the downloaded compounds). The drug-like properties of the top lead compounds and Doxycycline are provided in Table 1.

### 3.2. Analysis of ADMET Properties

The pkCSM web server integrates pharmacokinetic properties ADMET (chemical absorption, distribution, metabolism, excretion, and toxicity) of drug-like compounds based on cut-off scores. All ligands have a high absorption rate and water solubility in the absorption section. Each of the compounds evaluated was able to penetrate Caco-2 cell lines. In terms of distribution, however, not all of them are permeable to the blood-brain barrier (BBB). Following that, a few of the substances act as substrates for CYP3A4, CYP2C9, and CYP2D6 during distribution and excretion. There were no hepatotoxic ligands in the study. For further investigation, a total of 125 drug-like compounds were considered (Supplementary table 2: Pharmacokinetic properties of the selected drug-like molecules). The pharmacokinetics properties of the top lead compounds and Doxycycline are provided in Table 2.

### 3.3. Analysis of Molecular Docking Results by Autodock-Vina

Previously filtered drug-like molecules were screened in Autodock-vina docking software. Here, Doxycycline was used as the control drug for comparing our docking results. We set-up −11.0 kcal/mol as the cut-off docking score for all the ligands. Out of 125, 36 ligands showed better binding affinity in our study (Table 3).

### 3.4. Analysis of Molecular Docking Results by Swissdock

**Figure 1:**
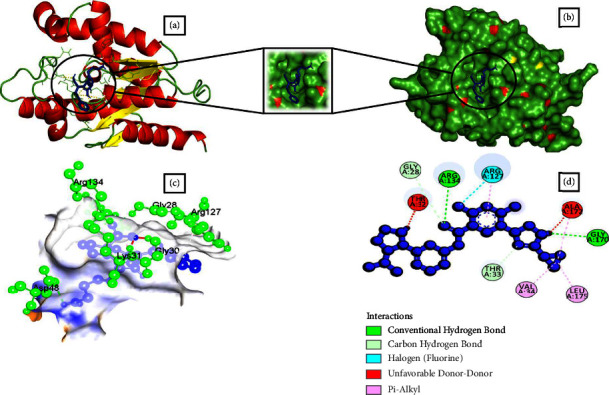
Schematic representation of Shikimate kinase_CHEMBL1237 complex. (a) Pose view of Shikimate kinase_CHEMBL123 complex. (b) Surface view of Shikimate kinase_CHEMBL123 complex. Here, protein is in red, yellow, and green color and ligand is in blue color. (c, d) 3D and 2D interaction of Shikimate kinase_CHEMBL123 complex. Here, protein is in green color and ligand is in blue color.

**Figure 2:**
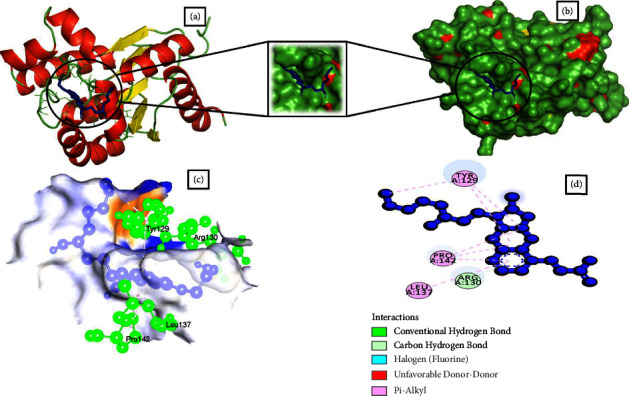
Schematic representation of Shikimate kinase_CHEMBL1237119 complex. (a) Pose view of Shikimate kinase_CHEMBL1237119 complex. (b) Surface view of Shikimate kinase_CHEMBL1237119 complex. Here, protein is in red, yellow, and green color and ligand is in blue color. (c, d) 3D and 2D interaction of Shikimate kinase_CHEMBL1237119 complex. Here, protein is in green color and ligand is in blue color.

**Figure 3:**
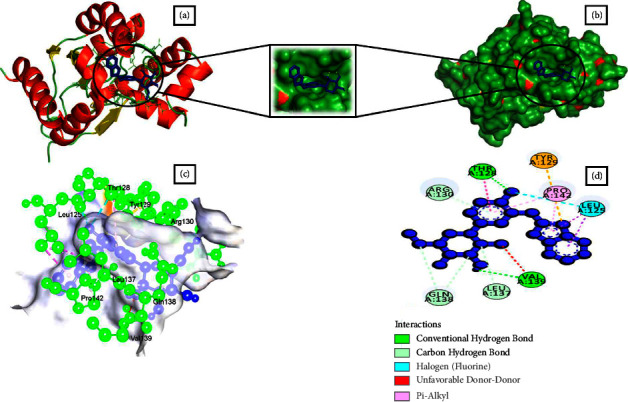
Schematic representation of Shikimate kinase_CHEMBL2018096 complex. (a) Pose view of Shikimate kinase_CHEMBL2018096 complex. (b) Surface view of Shikimate kinase_CHEMBL2018096 complex. Here, protein is in red, yellow, and green color and ligand is in blue color. (c, d) 3D and 2D interaction of Shikimate kinase_CHEMBL2018096 complex. Here, protein is in green color and ligand is in blue color.

**Figure 4:**
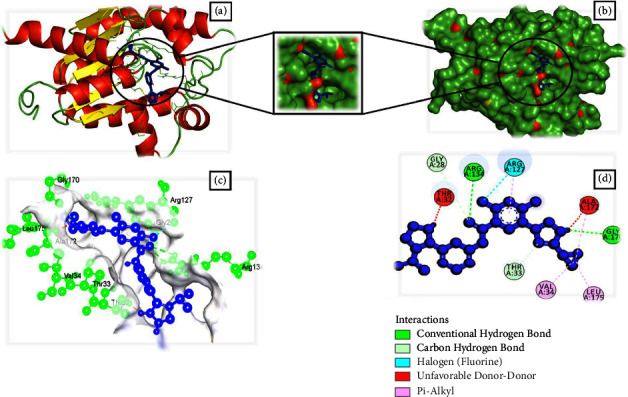
Schematic representation of Shikimate kinase_CHEMBL3916717 complex. (a) Pose view of Shikimate kinase_CHEMBL3916717 complex. (b) Surface view of Shikimate kinase_CHEMBL3916717 complex. Here, protein is in red, yellow, and green color and ligand is in blue color. (c, d) 3D and 2D interaction of Shikimate kinase_CHEMBL3916717 complex. Here, protein is in green color and ligand is in blue color.

Based on full fitness and estimated Δ*G* score, 16 small molecules showed higher binding energy than the control drug. Besides, four of them had a binding estimated Δ*G* lower than −8.0 kcal/mol. Among them, CHEMBL1237 (Figure 1), CHEMBL1237119 (Figure 2), CHEMBL2018096 (Figure 3), and CHEMBL3916717 (Figure 4) were selected considering all features as potential drug candidates for *Acinetobacter baumannii* (Table 3).

### 3.5. Analysis of MD Simulation by GROMACS

In this research, the conformational stability of protein-ligand complexes was assessed employing molecular dynamics analysis. The average outcomes of the characteristics considered are recorded in Table 4.

**Figure 5:**
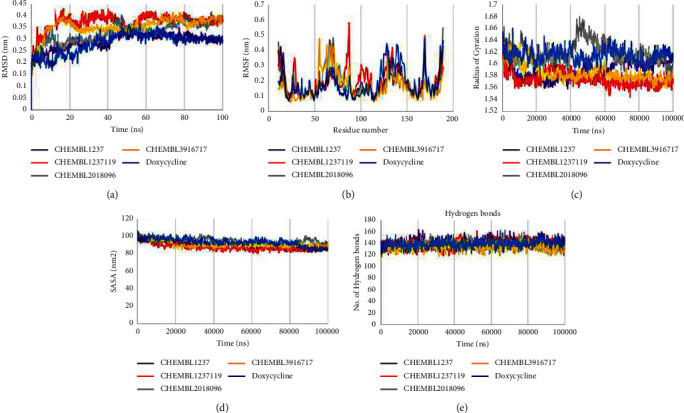
Schematic presentation of simulation plots. The plots represent features of (a) root mean square deviation (RMSD), (b) root mean square fluctuation (RMSF), (c) radius of gyration (Rg), (d) solvent-accessible surface area (SASA), and (e) hydrogen bonds (H-bonds) at 100 nanoseconds of molecular dynamics simulation using the GROMACS software.

The binding stability of four drugs with target proteins was examined using the root mean square deviation (RMSD) method. The RMSD fluctuation for the protein and ligand complex structures in the MD simulation trajectory is thoroughly analyzed, and the complex is considered stable if the fluctuation is less than 4 nm.  Figure 5(a) demonstrates the RMSD of protein-ligand complexes: Doxycycline, CHEMBL1237, CHEMBL1237119, CHEMBL2018096, and CHEMBL39167178. The average RMSD values of the protein-ligand complexes of CHEMBL1237, CHEMBL1237119, CHEMBL2018096, CHEMBL3916717, and Doxycycline were 0.292741261, 0.372786232, 0.328247228, 0.354916442, and 0.28501144 nm, respectively. Between 85 ns and 100 ns timescales, CHEMBL1237 showed a similar tendency to the control Doxycycline at about 0.3 nm. On the contrary, CHEMBL1237119, CHEMBL2018096, and CHEMBL3916717 complex had an increased RMSD value more than 0.3 nm after 40 ns. Alteration in the conformation of the *Cα* backbone of the systems was assessed using root mean square fluctuation (RMSF) analysis per residue. CHEMBL1237, CHEMBL1237119, CHEMBL2018096, CHEMBL3916717, and Doxycycline have average RMSFs of 0.18, 0.19, 0.18, 0.17, and 0.18 nm, respectively, confirming strong conformational interaction between protein and ligands (Figure 5(b)). However, the higher CHEMBL1237119 RMSF fluctuation revealed the presence of a loop in this site. The radius of gyration was used to detect the changes in compactness following ligand interaction to receptors.  Figure 5(c) shows the radius of gyration of CHEMBL1237, CHEMBL1237119, CHEMBL2018096, CHEMBL3916717, and Doxycycline. The average Rg values of CHEMBL1237, CHEMBL1237119, CHEMBL2018096, CHEMBL3916717, and Doxycycline were 1.591054436, 1.575640969, 1.620291858, 1.586389291, and 1.614269121 nm, respectively, suggesting that the CHEMBL1237 complex is more compact. The average SASA values of CHEMBL1237, CHEMBL1237119, CHEMBL2018096, CHEMBL3916717, and Doxycycline were 89.80918282, 87.57855544, 94.80261938, 90.62411988, and 93.36690809 nm^2^, respectively, as shown in Figure 5(d).  Figure 5(e) demonstrates that the average hydrogen bond interactions for the complexes CHEMBL1237, CHEMBL1237119, CHEMBL2018096, CHEMBL3916717, and Doxycycline were 142.0, 139.0, 137.0, 134.0, and 139.0.

### 3.6. Analysis of MD Simulation and Postsimulation MM-GBSA by Desmond

**Figure 6:**
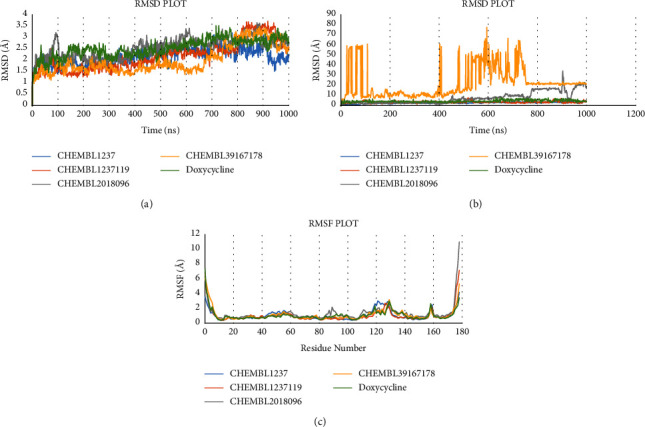
Graphical presentation of simulation curves. The plots represent features of (a, b) root mean square deviation (RMSD) and (c) root mean square fluctuation (RMSF), at 100 nanoseconds of molecular dynamics simulation using Desmond software.

We retrieved RMSD, RMSF, ligand behaviour, and protein-ligand interaction representation from the MD profile. Data analysis of the root mean square difference (RMSD) plot reveals the stabilization of complexes. The average RMSD of the complexes such as Shikimate kinase_CHEMBL1237, Shikimate kinase_CHEMBL1237119, Shikimate kinase_CHEMBL2018096, and Shikimate kinase_CHEMBL3916717 were 2.17, 2.19, 2.52, and 1.99 Å. The variation curve remained below 4.00 Å throughout 100 ns period, indicating a stable protein-ligand union. All complexes had fluctuations of less than 4.00 during a timeframe of 100 ns. It indicates that the ligands (Doxycycline, CHEMBL1237, CHEMBL1237119, CHEMBL2018096, and CHEMBL39167178) remained within the active pocket of shikimate kinase. A higher fluctuation around 3 Å was evident from 118 to 130 residues (loop region) (Figure 6).

**Figure 7:**
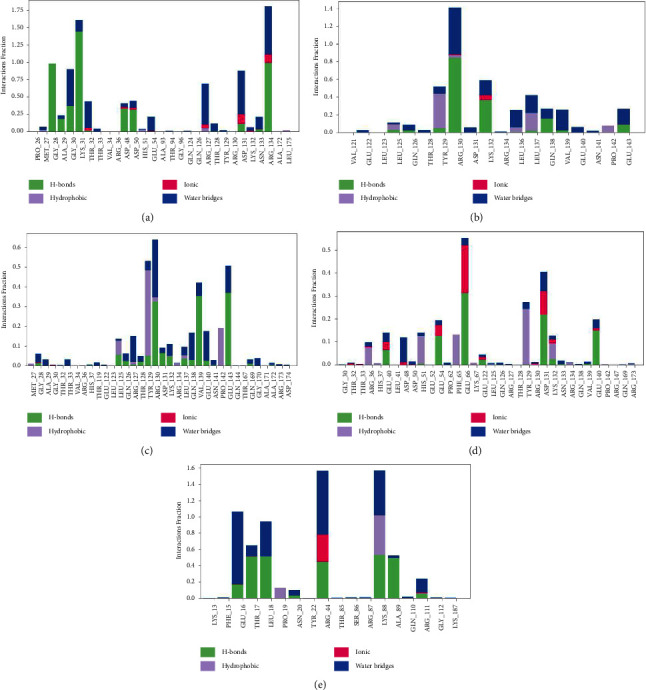
Diagram of protein-ligand complex chart of (a) Shikimate kinase_CHEMBL1237, (b) Shikimate kinase_CHEMBL1237119, (c) Shikimate kinase_CHEMBL2018096, (d) Shikimate kinase_CHEMBL3916717, and (e) Shikimate kinase_Doxycycline complexes.

The nonbonded contacts between the proteins and ligands were evaluated during the 100 ns timeframe. Ligand CHEMBL1237 produces MET27 (Hydrogen Bonds), ALA29 (hydrogen bonds and water bridges), GLY30 (hydrogen bonds and water bridges), GLN126 (hydrophobic, ionic bonds, and water bridges), ARG130 (hydrogen bonds, ionic bonds, and water bridges), ARG134 (hydrogen bonds, ionic bonds, and water bridges) contacts with shikimate kinase for 100%, 90%, 160%, 60%, 80%, and 175% of 100 ns timescale. Ligand CHEMBL1237119 completes bonds with THR128 (ionic bonds, hydrogen bonds, and water bridges), TYR129 (ionic bonds, hydrophobic, hydrogen bonds, and water bridges), ARG130 (hydrogen bonds, ionic bonds, and Water Bridges) for 50%, 140%, and 60% of simulation. Throughout the simulation period, ligand CHEMBL2018096 formed bonds with four amino acids - THR128, TYR129, GLN138, and PRO142. The types of bonds formed with each amino acid were hydrogen bonds, hydrophobic interactions, and water bridges for THR128 and TYR129, while GLN138 and PRO142 had hydrophobic interactions and hydrogen bonds. Specifically, the ligand completed these bonds for 55%, 65%, 45%, and 50% of the simulation period with THR128, TYR129, GLN138, and PRO142, respectively. Ligand CHEMBL39167178 interacts with PHE65 (hydrogen bonds, ionic bonds, and water bridges), and ARG130 (hydrogen bonds, ionic bonds, and water bridges) for 55% and 40% of simulation. On the other hand, Doxycycline forms interactions with PHE15 (hydrogen bonds and water bridges), THR17 (hydrogen bonds and water bridges), TYR22 (hydrogen bonds, ionic bonds, and water bridges), and ARG87 (hydrogen bonds, hydrophobic bonds, and water bridges) for 110%, 90%, 155%, and 155% of simulation (Figure 7). Analyzing the after-simulation MM-GBSA, we found free binding energy of −47.50 ± 16.33, −48.60 ± 14.39, −32.55 ± 22.29, and −42.13 ± 10.01 kcal mol^−1^ for Shikimate kinase_CHEMBL1237, Shikimate kinase_CHEMBL1237119, Shikimate kinase_CHEMBL2018096, and Shikimate kinase_CHEMBL3916717 complexes. On the contrary, Shikimate kinase_Doxycycline complex had a binding-free score of −33.50 ± 3.10 kcal mol^−1^ (Table 5). It indicates that our predicted ligand hits have greater binding interactions with the shikimate kinase compared with the control compound.

## 4. Discussion

*A. baumannii* is considered to be one of the world's most notorious superbugs, and the bacteria are listed as one of the most critical pathogens [8]. The organism has extended its spread throughout and from human to cattle to additional animal species. A novel drug is a must to combat *A. baumannii*. The resistance mechanism existed in *A. baumannii* is very efficient to hydrolyse currently used antibiotics. Therefore, the treatment option is limiting day-by-day. However, there is no progress regarding new effective drugs against it.

After the emergence of the multidrug-resistant variant of *Acinetobacter baumannii*, the race for a breakthrough in therapeutic research accelerated. Last-resort antibiotics: aminoglycosides, broad-spectrum cephalosporins, carbapenems, tigecycline, and colistin acquired resistance against *A. baumannii*. Our study is designed to identify potential small drug-like molecules against *A. baumannii* by employing a structure-based drug development (SDD) strategy [10]. Computational techniques are vital resources for evaluating and conducting research to speed up the development of antibiotic drugs [8]. SDD approach incorporates building protein structures, optimizing ligand molecules, evaluating drug-likeness properties and pharmacokinetic properties, binding interaction and affinity prediction, and validating structural stability and compactness [22].

Computer-aided drug discovery is one of the effective means to screen database and identify novel therapeutic agents against multidrug-resistant *A. baumannii*. In addition, the determination of novel drug targets is another important phase in the drug discovery process. Analysis of structural and functional roles of important proteins and identification of potential drug targets suggest possible targets for a new antimicrobial development. Moreover, different features such as screening, ADMET properties, permeability, Lipinski's rule of five, and drug likeliness enhance the acceptance, safety, and efficiency of a suggested drug-like compound. The stability of target protein-druglike compound refers to higher possibilities of a compound to work on a certain target [22]. Furthermore, the prediction of absorption of an oral drug is well determined using Caco-2 cell models derived from human colon carcinoma cells [23]. Our selected ligands such as CHEMBL1237, CHEMBL1237119, CHEMBL2018096, and CHEMBL39167178 qualified either of the criteria (Lipinski's rule of five and ADMET rules).

Identifying putative drug targets in the drug design approach is vital for the downstream study [24]. We selected shikimate kinase as a possible target due to its role in the chemical process of the shikimate pathway, which is necessary for the formation of chorismate, an important biochemical intermediate in the shikimate pathway that acts as a source of aromatic amino acids [2, 25]. This process considerably reduces the possibility of the incursion of resistance due to the inclusion of several targets in the same metabolic pathway. Previous research on *Mycobacterium tuberculosis* identified shikimate Kinase as a potential therapeutic target [25]. The large dataset of 2,041 chemical and phytochemical compounds was created with antimicrobial activity. A potential drug molecule should follow the physicochemical parameters enlisted in Lipinski's Rule of Five. A total of 1065 drug-like molecules comply with the guideline (hydrogen bond acceptors ≤10, hydrogen bond donors ≤5, logP <5, molecular mass <500, rotatable bonds <10, and polar surface area ≤140 Å) [26]. Given the compounds satisfy the key criteria, they might have better physicochemical characteristics and bioavailability in the metabolic activities. Following the drug-like property study, the toxicity and pharmacokinetic features of the small ligands were analyzed. It is widely accepted to evaluate ADMET (absorption, distribution, metabolism, excretion, and toxicity) properties before clinical trials of drugs as highly toxic and poor pharmacokinetics could ruin the expensive phases of drug designing [27]. We selected 125 small molecules from the pkCSM server based on pharmacokinetics and toxicity. Molecular docking is a well-established structure-based computational approach commonly utilized in drug designing [28].

The binding affinity and interaction of the chosen ligands were successfully predicted using multistep molecular docking. One of the previous studies revealed that natural epiestriol-16 showed potential inhibitory activity against *Acinetobacter baumannii* with −7.3, −8.0, and −6.0 kcal/mol against Pyr E, Pyr F, and Omp38 proteins [24]. Another research identified ligand ZINC01155930 (XP G-score: −4.953 kcal/mol) as a potential drug candidate for the efflux pump of *Acinetobacter baumannii* [23]. Regarding the most promising ligands, we analyzed binding energy and hydrogen bonds to determine their optimal binding interaction between protein and ligand. After a thorough analysis utilizing the structure-based drug development (SDD) procedure, we identified CHEMBL1237, CHEMBL1237119, CHEMBL2018096, and CHEMBL3916717 as the potential drug candidates. Ligands such as CHEMBL1237, CHEMBL1237119, CHEMBL2018096, and CHEMBL3916717 have a binding energy of −13.0, −13.1, −13.3, and −12.9 kcal/mol with 4 (GLY28, LYS31, ARG130), 1 (ARG130), 6 (THR128, VAL139, GLN138, LEU137, GLN138, ARG130), and 5 (ARG134, ARG130, GLY170, GLY28, THR33) hydrogen bonds. Similarly, they had an estimated ΔG value of −8.16, 8.42, and −8.14 kcal/mol. In comparison to earlier research, the results revealed that our therapeutic candidates have a greater binding affinity. A comparison study with the control drug Doxycycline helps identify the best ligand hits. One can estimate how distinct parts of the biomolecule fluctuate at equilibrium and experience structural variations by looking at a simulation of a structure. The study can also highlight the dynamic behaviour of water and salt ions, both of which are necessary for protein activity and small molecule attachment [23]. The results from simulation data conveyed that all of the four ligands remained stable throughout the 100 ns timescale. However, CHEMBL1237 (Lisinopril) has the most stable interaction with the protein. The RMSF plot shows that all ligands are only fluctuating within loop regions except CHEMBL1237119. Analysis from the Rg plot portrays that CHEMBL2018096 and Doxycycline are less stable compared to other ligands. As the protein begins to unfold during conformational changes, its hydrophobic, nonpolar interactions become exposed to the solvent. As a consequence, the composition of the protein becomes increasingly unstable. Protein solvent accessibility is identified using SASA calculation. The SASA study confirms that the ligands are stable upon binding with the protein. The number of hydrogen bonds always predicts the stability of protein-ligand complexes [29]. CHEMBL1237 (Lisinopril) has the highest number of hydrogen bonds, confirming its robust binding with the protein. Further analyzing the RMSD, RMSF, protein-ligand interaction diagram, and MM-GBSA evaluation from Desmond simulation, we found similar trend for CHEMBL1237 (Lisinopril) with a strong binding interaction with shikimate kinase. However, further studies are needed to confirm the potency and efficacy of the *in silico* evaluated compounds.

## 5. Conclusions

The current work focuses on screening available databases to find new drug-like compounds against *A. baumannii* based on the computational approaches. Research included the phyto and chemical compounds against a potent target, shikimate kinase of the organism, and characterised the features of the compounds so that they can be exploited for developing new drugs against the bacteria. *In silico* evaluation of the compounds suggested four drug-like molecules, CHEMBL1237, CHEMBL1237119, CHEMBL2018096, and CHEMBL3916717, in comparison to Doxycycline, that have met the physical properties and biological acceptance of a drug. Molecular docking results of the ligands with shikimate kinase suggested that the compounds have binding pockets on further evaluation using MD simulation showed that the complexes have high affinities and stability, thereby suggesting their potentiality to act as drugs. The present findings can be the basis of starting new drug development work; however, further *in vitro* and *in vivo* studies are essential to validate the effectivity of the compounds and to develop new therapeutic agents against *A. baumannii*.

## Figures and Tables

**Table 1:** Drug-like properties of the top lead compounds and doxycycline from QikProp module of Schrödinger software.

Name	MW (g/mol)	AlogP	HBA	HBD	RB	PSA (Å^2^)
CHEMBL1237	390.5131	4.41	3	2	10	86.99
CHEMBL1237119	390.5131	4.41	3	2	10	86.99
CHEMBL2018096	404.4518	2.26	5	4	4	118.39
CHEMBL39167178	445.4921	3.8751	5	1	6	90.52
Doxycycline	444.43	−0.28	9	6	2	181.62

**Table 2:** Pharmacokinetics properties of the top lead compounds and Doxycycline from the pkCSM web server.

CHEMBL	Water solubility	Caco-2permeability	Intestinal absorption (human)	P-gpsubstrate	P gp-Iinhibitor	BBB permeability	CYP2D6 substrate	CYP1A2 inhibitor	CYP2C19 inhibitor	CYP2C3A4 inhibitor	AMES toxicity	Hepatotoxicity
CHEMBL1237	−4.307	0.941	91.321	Yes	No	−0.747	No	No	No	No	No	No
CHEMBL1237119	−4.307	0.941	91.321	Yes	No	−0.747	No	No	No	No	No	No
CHEMBL2018096	−3.66	1.055	93.499	Yes	Yes	−0.999	No	Yes	No	No	No	No
CHEMBL39167178	−4.49	1.391	95.72	No	No	0.347	No	Yes	Yes	Yes	No	Yes
Doxycycline	−2.449	0.154	44.517	Yes	No	−0.802	No	No	No	No	No	No

**Table 3:** Binding affinity, estimated Δ*G*, and hydrogen bonds of the selected drug-like molecules and shikimate kinase.

Drug	Binding affinity (kcal/mol)	Estimated Δ*G*(kcal/mol)	Full fitness (kcal/mol)	Hydrogen bonds	Amino acid number
CHEMBL2107005	−11.5	−7.27	−1057.43	3	ARG130, PRO142
CHEMBL304266	−13.6	−7	−1051.73	3	THR128, LEU125, THR128
CHEMBL304818	−12.0	−7.52	−1105.7	1	ARG130
CHEMBL2107131	−12.0	−7.45	−1090.95	5	ASN133, ASP48, GLU54, ARG134
CHEMBL2107774	−11.8	−7.29	−1069.97	6	ARG130, ASP131, THR128, GLN138, ARG130
CHEMBL2220486	−14.2	−7.97	−822.22	5	THR128, ARG130, GLN138, GLN138,GLN138
CHEMBL309962	−12.3	−7.17	−1123.03	2	LYS88
CHEMBL1516410	−11.2	−8.14	−1096.97	6	ALA29, GLY30, LYS31, LYS31, ARG134, ARG134
CHEMBL471498	−11.2	−7.76	−1132.53	1	ARG130
CHEMBL161702	−11.4	−7.99	−1098.73	7	ARG130,GLU140, ARG130, GLN138, PRO142, ARG130,
CHEMBL2104660	−12.2	−7.92	−1097.45	1	ARG130
CHEMBL491510	−12.8	−7.51	−1103.68	8	ARG130, GLN138, LEU125, LEU137,ARG130, ARG130, ARG130
CHEMBL2104358	−16.8	−7.54	−1071	2	ARG74, ASN133
CHEMBL54440	−13.4	−7.16	−1130	2	GLN138, GLN138
CHEMBL1200374	−13.6	−6.93	−1059.06	2	ARG74, ASN133
CHEMBL3039507	−13.5	−7.51	−1015.5	3	LEU137, THR128, LEU125
CHEMBL1697842	−11.4	−7.12	−1097.55	1	THR128
CHEMBL1237119	−13.1	−8.42	−1084.84	1	ARG130
CHEMBL223228	−11.2	−6.55	−863.54	1	ASN133
CHEMBL1508	−12.9	−7.08	−1049.61	1	THR128
CHEMBL1908332	−14.3	−7.22	−1136.95	1	GLN138
CHEMBL783	−11.3	−7.44	−1095.06	0	N/A
CHEMBL1614644	−11.7	−7.67	−1075.44	3	PHE65, GLU66
CHEMBL3916717	−12.9	−8.97	−942.4	3	GLN138, GLN138, GLN138
CHEMBL370753	−14.3	−7.88	−1006.83	3	VAL139, VAL139, GLN138
CHEMBL1444	−11.2	−7.75	−1071.86	3	ASP131, GLN138, ARG130
CHEMBL2018096	−13.3	−8.14	−1008.49	6	THR128, VAL139, GLN138, LEU137, GLN138, ARG130
CHEMBL4594348	−11.5	−7.11	−1048.77	6	GLN138, ASP131, THR128, ARG130, ARG130
CHEMBL204656	−13.9	−7.7	−1063.36	2	LEU137, TYR129
CHEMBL518924	−11.5	−6.77	−1023.67	7	ASN133, ARG134, ARG134, GLY28, ARG127, THR32
CHEMBL2347655	−13.0	−7.96	−1051	6	ARG134, ARG134, ARG134, ARG134, GLY30, ASP50
CHEMBL70663	−11.1	−7.73	−1134.36	1	LEU137
CHEMBL1237	−13.0	−8.16	−1046.73	4	GLY28, LYS31, ARG130
CHEMBL286738	−11.8	−7.23	−1141.99	9	GLY28, ALA29, GLY30, LYS31, LYS31, LYS31, THR32, ARG34, ARG34
CHEMBL385517	−13.3	−7.19	−830.74	3	ALA89, GLU16, ARG111
CHEMBL2042273	−11.5	−7.77	−1040.65	3	VAL139, VAL139, GLN138
Doxycycline	−6.9	−7.1	−1066.59	4	ALA89, GLU16, LEU18, LYS88, ARG111

**Table 4:** Average values of simulated ligand-protein complexes at 100 nanoseconds.

Characteristics	CHEMBL1237	CHEMBL1237119	CHEMBL2018096	CHEMBL39167178	Doxycycline
RMSD (nm)	0.2927	0.3728	0.3282	0.3549	0.2850
RMSF (nm)	0.18	0.19	0.18	0.17	0.18
Rg (nm)	1.5911	1.5756	1.6203	1.5864	1.6143
SASA (nm^2^)	89.8092	87.5786	94.8024	90.6241	93.3669
H-bonds	142	139	137	134	139

The values represent average of each of the features of root mean square deviation (RMSD), root mean square fluctuation (RMSF), the radius of gyration (Rg), solvent-accessible surface area (SASA), and hydrogen bonds (H-bonds).

**Table 5:** Postsimulation binding-free calculation (MM-GBSA).

Name of complex	MM-GBSA (kcal·mol^−1^)	
	ΔGbind	ΔGbind range
Shikimate kinase_CHEMBL1237 complex	−47.50 ± 16.33	−63.83 to −31.19
Shikimate kinase_CHEMBL1237119 complex	−48.60 ± 14.39	−62.99 to −34.21
Shikimate kinase_CHEMBL2018096 complex	−32.55 ± 22.29	−54.84 to −10.27
Shikimate kinase_CHEMBL3916717 complex	−42.13 ± 10.01	−52.14 to −33.13
Shikimate kinase_Doxycycline complex	−33.50 ± 3.10	−36.61 to −30.40

## Data Availability

The datasets supporting the conclusions of this study are included within the article (and its additional files).
