# Supplementary material for: Targeting Shikimate Kinase Pathway of Acinetobacter baumannii: A Structure-Based Computational Approach to Identify Antibacterial Compounds
Source: J Trop Med. 2023 Mar 29;2023:6360187. doi: 10.1155/2023/6360187 (PMC10076115; doi:10.1155/2023/6360187)
Supplement: Supplementary Materials — Supplementary files are provided along with the manuscript. [file 6360187.f1.docx]

**Supplementary data**

**Supplementary table 1. Drug-like properties of the downloaded compounds.**

| Name | MW | AlogP | HBA | HBD | RB | PSA |
| --- | --- | --- | --- | --- | --- | --- |
| CHEMBL210618 | 356.3758 | 2.0144 | 6 | 2 | 7 | 119.49 |
| CHEMBL261131 | 489.5794 | 7.061 | 1 | 1 | 5 | 73.12 |
| CHEMBL235641 | 268.2938 | 0.6355 | 3 | 2 | 1 | 98.91 |
| CHEMBL97771 | 373.4046 | 5.0075 | 5 | 1 | 6 | 65.5 |
| CHEMBL2106793 | 281.3077 | -0.7377 | 5 | 2 | 8 | 89.55 |
| CHEMBL2105703 | 392.3652 | 3.708 | 1 | 0 | 3 | 21.06 |
| CHEMBL1317546 | 221.2524 | 1.1524 | 3 | 1 | 2 | 49.77 |
| CHEMBL2216863 | 421.4507 | 2.7996 | 5 | 2 | 4 | 129.62 |
| CHEMBL325166 | 558.6498 | 0.1031 | 5 | 3 | 12 | 194.37 |
| CHEMBL2105508 | 300.3573 | 1.3497 | 5 | 2 | 5 | 143.82 |
| CHEMBL9440 | 436.5267 | 2.3941 | 4 | 0 | 5 | 96.61 |
| CHEMBL3694252 | 269.3813 | 5.5565 | 2 | 1 | 4 | 33.12 |
| CHEMBL282731 | 474.3711 | 2.025 | 8 | 4 | 11 | 208.12 |
| CHEMBL4650521 | 421.9475 | 1.5347 | 4 | 2 | 3 | 154.5 |
| CHEMBL1201582 | 251.3263 | 3.6286 | 1 | 0 | 4 | 21.06 |
| CHEMBL3989670 | 368.4311 | 2.9752 | 5 | 1 | 4 | 51.56 |
| CHEMBL3544915 | 891.8027 | 5.2981 | 9 | 3 | 15 | 229.45 |
| CHEMBL459178 | 358.4363 | 4.2223 | 2 | 2 | 4 | 87.05 |
| CHEMBL4650223 | 453.9993 | 4.4387 | 5 | 3 | 7 | 49.77 |
| CHEMBL144620 | 217.1374 | -0.5544 | 1 | 3 | 2 | 108.02 |
| CHEMBL4757879 | 580.7231 | 6.479 | 5 | 2 | 8 | 100.44 |
| CHEMBL228792 | 215.2114 | 0.2262 | 4 | 2 | 3 | 79.63 |
| CHEMBL4533605 | 391.4001 | -1.0691 | 5 | 2 | 5 | 165.76 |
| CHEMBL2110630 | 312.4061 | 3.8015 | 3 | 1 | 8 | 54.34 |
| CHEMBL4594249 | 578.7222 | 2.8394 | 5 | 3 | 13 | 136.22 |
| CHEMBL4594621 | 304.3856 | 3.8269 | 2 | 2 | 3 | 45.15 |
| CHEMBL29835 | 251.3263 | 3.6286 | 1 | 0 | 4 | 21.06 |
| CHEMBL2107436 | 276.2021 | 3.4638 | 1 | 0 | 2 | 12.47 |
| CHEMBL2108699 | 468.489 | 0.421 | 8 | 1 | 5 | 170.18 |
| CHEMBL3639433 | 416.4114 | 2.8838 | 3 | 0 | 3 | 80.34 |
| CHEMBL2170601 | 340.7637 | 4.4831 | 2 | 2 | 5 | 87.14 |
| CHEMBL4298147 | 507.0172 | 5.6858 | 3 | 1 | 4 | 104.74 |
| CHEMBL257590 | 371.3773 | 2.9099 | 3 | 0 | 4 | 61.89 |
| CHEMBL3666062 | 488.3758 | 5.0407 | 3 | 2 | 7 | 70.56 |
| CHEMBL261932 | 410.9132 | 5.6742 | 1 | 0 | 8 | 79.67 |
| CHEMBL378225 | 578.7222 | 2.8394 | 5 | 3 | 13 | 136.22 |
| CHEMBL2108349 | 350.3282 | 3.4231 | 2 | 2 | 6 | 124.44 |
| CHEMBL114586 | 373.4079 | 2.3912 | 4 | 1 | 6 | 112.59 |
| CHEMBL3137358 | 771.9209 | 5.229 | 9 | 1 | 8 | 181.48 |
| CHEMBL64706 | 361.3956 | -0.0531 | 5 | 3 | 8 | 137.61 |
| CHEMBL3661407 | 315.3652 | 4.5382 | 1 | 0 | 5 | 41.96 |
| CHEMBL2104184 | 327.2488 | 3.8166 | 2 | 0 | 3 | 24.83 |
| CHEMBL179583 | 363.7971 | 4.1325 | 3 | 2 | 4 | 81 |
| CHEMBL23811 | 262.6486 | 2.4232 | 5 | 0 | 2 | 65.22 |
| CHEMBL3979920 | 344.3866 | 0.925 | 3 | 2 | 3 | 97.86 |
| CHEMBL301958 | 304.3856 | 3.8269 | 2 | 2 | 3 | 45.15 |
| CHEMBL457726 | 442.5958 | 6.6107 | 2 | 2 | 6 | 87.05 |
| CHEMBL3651711 | 433.4203 | 4.918 | 4 | 1 | 7 | 60.69 |
| CHEMBL3544920 | 456.937 | 4.4182 | 4 | 0 | 6 | 96.46 |
| CHEMBL2110886 | 364.5188 | 4.3281 | 2 | 2 | 10 | 77.76 |
| CHEMBL2108346 | 0 | 0 | 0 | 0 | 0 | 0 |
| CHEMBL3989664 | 576.665 | -0.1041 | 6 | 4 | 12 | 194.37 |
| CHEMBL2104330 | 258.271 | -0.2934 | 3 | 2 | 4 | 106.94 |
| CHEMBL2104528 | 144.2578 | 2.0068 | 0 | 2 | 6 | 38.05 |
| CHEMBL2104720 | 299.3675 | 2.2992 | 4 | 0 | 9 | 57.01 |
| CHEMBL4297220 | 486.2994 | 3.7618 | 5 | 3 | 6 | 103.37 |
| CHEMBL4297193 | 999.1683 | 5.6876 | 6 | 4 | 13 | 228.56 |
| CHEMBL2107322 | 616.9229 | 3.0195 | 6 | 3 | 11 | 151.49 |
| CHEMBL2105764 | 541.229 | 4.253 | 3 | 1 | 2 | 75.81 |
| CHEMBL2220475 | 508.4174 | 5.192 | 5 | 1 | 9 | 99.91 |
| CHEMBL2105739 | 453.5126 | 0.8868 | 4 | 2 | 7 | 173.96 |
| CHEMBL332003 | 488.9175 | 2.0574 | 7 | 1 | 8 | 108.25 |
| CHEMBL2103874 | 337.4404 | 1.112 | 4 | 2 | 5 | 99.36 |
| CHEMBL2111132 | 238.2082 | 1.8081 | 6 | 0 | 1 | 98.06 |
| CHEMBL2170592 | 350.3282 | 3.4231 | 2 | 2 | 6 | 124.44 |
| CHEMBL2107323 | 618.9387 | 2.8347 | 6 | 3 | 12 | 151.49 |
| CHEMBL1788400 | 399.2253 | 3.2735 | 3 | 1 | 8 | 92.91 |
| CHEMBL63055 | 255.2256 | 1.2269 | 2 | 0 | 2 | 74.68 |
| CHEMBL2107005 | 394.4602 | 4.2508 | 5 | 0 | 2 | 53.99 |
| CHEMBL76688 | 434.4629 | 2.2959 | 5 | 2 | 9 | 147.25 |
| CHEMBL2106980 | 289.3743 | 3.6601 | 1 | 0 | 2 | 28.16 |
| CHEMBL2170804 | 468.489 | 0.421 | 8 | 1 | 5 | 170.18 |
| CHEMBL3040926 | 219.3045 | -0.6891 | 2 | 1 | 7 | 124.5 |
| CHEMBL1788390 | 267.238 | 0.6995 | 4 | 0 | 6 | 104.33 |
| CHEMBL1788390 | 267.238 | 0.6995 | 4 | 0 | 6 | 104.33 |
| CHEMBL4297640 | 529.9183 | 2.8325 | 4 | 3 | 6 | 130.47 |
| CHEMBL2104499 | 513.0698 | 2.7538 | 4 | 0 | 11 | 149.48 |
| CHEMBL489079 | 197.2244 | 2.1616 | 0 | 0 | 4 | 26.02 |
| CHEMBL3544982 | 663.5869 | 7.5611 | 5 | 1 | 7 | 122.26 |
| CHEMBL1697741 | 478.623 | 4.9576 | 4 | 0 | 7 | 52.19 |
| CHEMBL2107736 | 481.9944 | 6.3055 | 3 | 1 | 6 | 112.58 |
| CHEMBL2105641 | 562.0354 | 5.3875 | 5 | 3 | 5 | 166.75 |
| CHEMBL497011 | 366.4567 | 2.3663 | 3 | 3 | 6 | 79.18 |
| CHEMBL4594279 | 527.6324 | 2.8235 | 5 | 1 | 9 | 126.44 |
| CHEMBL2218915 | 466.5411 | 4.6853 | 5 | 2 | 9 | 75.99 |
| CHEMBL1201405 | 470.5149 | 2.7034 | 3 | 0 | 10 | 125.4 |
| CHEMBL396298 | 563.6429 | 5.7481 | 5 | 2 | 8 | 92.89 |
| CHEMBL2106598 | 277.2074 | 0.5491 | 2 | 0 | 2 | 77.51 |
| CHEMBL2219410 | 358.7719 | 2.4726 | 2 | 0 | 5 | 82.65 |
| CHEMBL2105674 | 628.6188 | 6.4176 | 6 | 3 | 7 | 163.02 |
| CHEMBL2347651 | 613.1673 | 5.3204 | 4 | 0 | 9 | 143.97 |
| CHEMBL2146090 | 753.8131 | 4.2562 | 9 | 2 | 8 | 176.87 |
| CHEMBL34913 | 338.3971 | 3.041 | 4 | 1 | 3 | 94.57 |
| CHEMBL31965 | 485.9384 | 3.9337 | 5 | 2 | 9 | 88.61 |
| CHEMBL4298164 | 763.7637 | 3.8764 | 7 | 3 | 15 | 237.54 |
| CHEMBL4303303 | 569.6342 | 3.8719 | 8 | 1 | 10 | 145.44 |
| CHEMBL114715 | 438.5642 | 0.4307 | 3 | 2 | 12 | 160.64 |
| CHEMBL42126 | 381.2531 | 4.1622 | 4 | 1 | 5 | 60.45 |
| CHEMBL448864 | 102.0919 | -1.7879 | 2 | 1 | 0 | 64.35 |
| CHEMBL4597193 | 488.0189 | 4.5124 | 4 | 2 | 5 | 83.66 |
| CHEMBL522502 | 356.4204 | 4.457 | 3 | 2 | 6 | 73.06 |
| CHEMBL3545283 | 401.8402 | 3.7895 | 5 | 3 | 3 | 94.14 |
| CHEMBL150315 | 333.7447 | 4.3172 | 4 | 1 | 4 | 56.27 |
| CHEMBL433041 | 359.5056 | 5.3696 | 2 | 1 | 8 | 44.81 |
| CHEMBL78946 | 892.9921 | -1.3229 | 8 | 8 | 29 | 375.84 |
| CHEMBL1631216 | 450.3676 | 4.859 | 1 | 2 | 8 | 85.16 |
| CHEMBL544862 | 209.7151 | 1.7445 | 0 | 0 | 1 | 12.03 |
| CHEMBL288064 | 395.2182 | 3.2237 | 4 | 0 | 4 | 77.11 |
| CHEMBL84336 | 59.0903 | 0.6459 | 1 | 1 | 0 | 62.59 |
| CHEMBL1256842 | 354.3997 | 2.358 | 0 | 1 | 3 | 103.86 |
| CHEMBL4802251 | 473.5156 | 2.6158 | 4 | 2 | 9 | 102.48 |
| CHEMBL289556 | 408.5349 | 2.0587 | 3 | 2 | 5 | 150.81 |
| CHEMBL3661408 | 320.4048 | 4.5957 | 1 | 0 | 4 | 60.89 |
| CHEMBL4594454 | 395.8772 | 0.2536 | 5 | 2 | 2 | 70.68 |
| CHEMBL2105690 | 1047.535 | 10.288 | 6 | 2 | 17 | 170.42 |
| CHEMBL4650219 | 449.2991 | 2.3746 | 5 | 3 | 7 | 142.47 |
| CHEMBL3137325 | 696.2989 | 4.4453 | 5 | 1 | 6 | 112.57 |
| CHEMBL4596392 | 422.4405 | 1.0089 | 7 | 3 | 7 | 135.95 |
| CHEMBL3989448 | 505.6284 | 4.1975 | 5 | 1 | 6 | 88.61 |
| CHEMBL261237 | 335.3468 | 3.7057 | 1 | 2 | 4 | 101.13 |
| CHEMBL37858 | 385.3641 | 3.2925 | 1 | 0 | 3 | 74.57 |
| CHEMBL3989835 | 657.6632 | 3.8266 | 11 | 2 | 8 | 154.54 |
| CHEMBL273196 | 416.5172 | 0.0645 | 4 | 1 | 11 | 142.91 |
| CHEMBL4203190 | 385.9335 | 2.7592 | 2 | 1 | 7 | 70.83 |
| CHEMBL3655700 | 688.8379 | -1.4774 | 8 | 4 | 19 | 253.43 |
| CHEMBL342372 | 421.5105 | 3.3612 | 3 | 0 | 9 | 144.6 |
| CHEMBL4098877 | 379.4555 | 3.562 | 5 | 1 | 4 | 76.16 |
| CHEMBL1257753 | 398.9042 | 5.7328 | 2 | 1 | 3 | 41.05 |
| CHEMBL493936 | 543.6384 | -0.9428 | 6 | 4 | 11 | 222.94 |
| CHEMBL3039527 | 430.9311 | 1.6288 | 4 | 2 | 6 | 95.31 |
| CHEMBL4298160 | 561.6041 | 2.764 | 5 | 1 | 5 | 141.93 |
| CHEMBL4297651 | 478.6215 | 2.4267 | 6 | 0 | 9 | 76.3 |
| CHEMBL2105234 | 297.3914 | 1.5226 | 2 | 1 | 0 | 60.16 |
| CHEMBL51314 | 307.3432 | 3.4751 | 3 | 1 | 2 | 63.24 |
| CHEMBL207602 | 270.4507 | 6.4325 | 2 | 0 | 14 | 26.3 |
| CHEMBL1790497 | 930.186 | 4.6887 | 10 | 6 | 25 | 228.05 |
| CHEMBL3661415 | 195.1721 | 0.865 | 2 | 1 | 3 | 75.9 |
| CHEMBL153427 | 276.2878 | 2.182 | 6 | 2 | 4 | 92.54 |
| CHEMBL411491 | 395.2311 | 1.9147 | 4 | 3 | 3 | 122.03 |
| CHEMBL3991122 | 381.5509 | 5.4271 | 2 | 1 | 7 | 32.7 |
| CHEMBL507322 | 635.7338 | 1.4474 | 7 | 3 | 13 | 209.39 |
| CHEMBL272342 | 746.1409 | 10.747 | 2 | 1 | 14 | 96.78 |
| CHEMBL2219423 | 463.7841 | 3.6372 | 2 | 1 | 6 | 101.18 |
| CHEMBL579 | 438.5608 | 2.3766 | 3 | 0 | 8 | 157.54 |
| CHEMBL3545364 | 580.6537 | 0.5 | 8 | 2 | 5 | 157.03 |
| CHEMBL3545124 | 363.8617 | 1.3238 | 2 | 2 | 4 | 93.44 |
| CHEMBL269787 | 748.1137 | 9.7483 | 3 | 1 | 14 | 106.01 |
| CHEMBL304266 | 340.4129 | 4.3382 | 4 | 4 | 0 | 80.92 |
| CHEMBL481122 | 558.2684 | 2.5917 | 4 | 3 | 6 | 105.82 |
| CHEMBL1288786 | 236.272 | -0.6933 | 5 | 2 | 3 | 151.66 |
| CHEMBL32838 | 458.544 | 3.1825 | 5 | 0 | 11 | 97.89 |
| CHEMBL4297217 | 582.0704 | 4.5085 | 6 | 1 | 6 | 144.15 |
| CHEMBL3666063 | 380.8507 | 3.7006 | 3 | 2 | 3 | 109.14 |
| CHEMBL1189780 | 256.2997 | 2.14 | 1 | 0 | 3 | 55.12 |
| CHEMBL19611 | 388.4607 | 1.0831 | 4 | 5 | 9 | 123.32 |
| CHEMBL273910 | 449.2024 | 2.4084 | 4 | 1 | 2 | 83.55 |
| CHEMBL94507 | 652.1262 | -2.8676 | 22 | 2 | 12 | 395.22 |
| CHEMBL3989607 | 570.2073 | 3.4488 | 4 | 4 | 15 | 122.8 |
| CHEMBL313489 | 609.1531 | 8.0955 | 2 | 0 | 10 | 92.48 |
| CHEMBL2107578 | 350.4158 | 3.1824 | 4 | 0 | 4 | 51.56 |
| CHEMBL507614 | 167.1851 | 1.3665 | 0 | 1 | 1 | 81.33 |
| CHEMBL2106281 | 269.681 | 2.4548 | 5 | 0 | 6 | 61.56 |
| CHEMBL4587433 | 383.4243 | 0.9817 | 5 | 2 | 3 | 132.49 |
| CHEMBL3989942 | 644.8234 | 4.4748 | 6 | 0 | 5 | 152.68 |
| CHEMBL2107332 | 501.6214 | 8.3537 | 4 | 0 | 10 | 68.62 |
| CHEMBL304818 | 326.4293 | 5.4771 | 3 | 1 | 8 | 54.37 |
| CHEMBL499200 | 591.764 | 3.4105 | 5 | 2 | 12 | 159.98 |
| CHEMBL3661413 | 209.1986 | 1.2425 | 2 | 1 | 3 | 75.9 |
| CHEMBL261720 | 425.4362 | 2.8113 | 4 | 4 | 6 | 181.42 |
| CHEMBL1788387 | 502.2593 | 3.3224 | 4 | 0 | 14 | 59.08 |
| CHEMBL453766 | 593.7369 | 2.6678 | 6 | 2 | 12 | 169.21 |
| CHEMBL3643413 | 450.4574 | 2.6563 | 5 | 1 | 6 | 83.48 |
| CHEMBL3655685 | 549.6876 | 1.3452 | 5 | 3 | 10 | 189.66 |
| CHEMBL3655686 | 553.7194 | 0.8123 | 5 | 2 | 10 | 189.13 |
| CHEMBL85959 | 299.4106 | 4.4083 | 3 | 2 | 8 | 58.04 |
| CHEMBL2107829 | 299.8361 | 2.5564 | 2 | 1 | 6 | 55.48 |
| CHEMBL2107131 | 333.3789 | 2.7508 | 3 | 0 | 7 | 83.91 |
| CHEMBL4297515 | 1035.199 | 4.5519 | 15 | 2 | 22 | 279.13 |
| CHEMBL2106909 | 284.396 | 3.3984 | 2 | 0 | 9 | 27.05 |
| CHEMBL2107344 | 718.7547 | 7.5646 | 4 | 0 | 13 | 67.67 |
| CHEMBL2106429 | 630.6661 | 0.4867 | 9 | 5 | 6 | 221.56 |
| CHEMBL2104622 | 272.2991 | 1.5893 | 2 | 0 | 5 | 64.35 |
| CHEMBL3039536 | 591.1421 | -1.707 | 4 | 2 | 16 | 323.6 |
| CHEMBL2105775 | 742.432 | 3.1635 | 7 | 4 | 17 | 188.79 |
| CHEMBL2105388 | 382.4944 | 2.8679 | 3 | 0 | 10 | 95.94 |
| CHEMBL3989608 | 416.5106 | 2.9877 | 3 | 0 | 10 | 95.94 |
| CHEMBL2104960 | 302.3682 | 3.2953 | 3 | 1 | 7 | 56.25 |
| CHEMBL2105633 | 450.3749 | 0.5693 | 3 | 0 | 4 | 68.03 |
| CHEMBL4301163 | 629.2823 | 5.7334 | 6 | 2 | 8 | 133.74 |
| CHEMBL437526 | 523.5378 | 0.1324 | 8 | 2 | 6 | 147.24 |
| CHEMBL4297225 | 498.06 | 4.1234 | 5 | 2 | 4 | 103.69 |
| CHEMBL2105433 | 412.4622 | 2.001 | 5 | 1 | 9 | 150.88 |
| CHEMBL2107711 | 383.4855 | 5.1878 | 2 | 1 | 7 | 41.15 |
| CHEMBL3137334 | 579.6624 | 3.3268 | 8 | 4 | 7 | 170.81 |
| CHEMBL4651180 | 451.4685 | 3.8074 | 4 | 0 | 4 | 97.17 |
| CHEMBL3301596 | 328.6639 | 4.4609 | 0 | 0 | 1 | 26.02 |
| CHEMBL2105696 | 472.9645 | 2.4309 | 5 | 1 | 8 | 77.85 |
| CHEMBL1201391 | 436.5815 | 4.026 | 4 | 2 | 11 | 104.06 |
| CHEMBL2105742 | 618.8126 | 4.8416 | 5 | 2 | 10 | 106.17 |
| CHEMBL3655691 | 611.7554 | 0.5255 | 6 | 3 | 12 | 186.17 |
| CHEMBL2107809 | 379.386 | 2.2534 | 3 | 1 | 8 | 114.45 |
| CHEMBL536151 | 383.6729 | 4.8963 | 2 | 2 | 4 | 49.33 |
| CHEMBL2218880 | 253.3373 | 2.0398 | 3 | 1 | 3 | 55.4 |
| CHEMBL592374 | 228.1177 | 2.6907 | 0 | 0 | 1 | 12.03 |
| CHEMBL2104522 | 424.5988 | 4.2808 | 2 | 1 | 7 | 69.08 |
| CHEMBL2105716 | 403.273 | 3.5544 | 2 | 0 | 5 | 96.14 |
| CHEMBL2105752 | 409.5013 | 3.8778 | 4 | 1 | 7 | 83.67 |
| CHEMBL2105679 | 466.9433 | 4.3983 | 6 | 1 | 7 | 121.79 |
| CHEMBL2107579 | 325.4081 | 3.342 | 3 | 0 | 4 | 55.95 |
| CHEMBL4802253 | 446.4406 | 4.0092 | 2 | 0 | 7 | 64.15 |
| CHEMBL2107326 | 522.3922 | 3.5748 | 6 | 3 | 8 | 123.54 |
| CHEMBL3137329 | 326.3929 | 0.3823 | 3 | 4 | 3 | 104.03 |
| CHEMBL1788384 | 347.2854 | -0.1095 | 3 | 3 | 3 | 120.16 |
| CHEMBL2158055 | 561.7149 | 2.2641 | 4 | 2 | 9 | 110.85 |
| CHEMBL167055 | 265.2188 | 0.7982 | 4 | 2 | 4 | 117.74 |
| CHEMBL3989681 | 757.9148 | 3.8572 | 10 | 9 | 24 | 217.39 |
| CHEMBL2107774 | 327.2735 | 4.7837 | 0 | 1 | 5 | 49.33 |
| CHEMBL3707361 | 430.5372 | 3.4439 | 3 | 0 | 10 | 95.94 |
| CHEMBL331369 | 430.5372 | 3.4439 | 3 | 0 | 10 | 95.94 |
| CHEMBL350775 | 603.7052 | 5.202 | 9 | 0 | 16 | 117.15 |
| CHEMBL2107329 | 385.4402 | 2.5511 | 4 | 3 | 6 | 147.43 |
| CHEMBL417975 | 338.4666 | 1.05 | 4 | 1 | 4 | 151.33 |
| CHEMBL329012 | 375.4635 | 3.7499 | 3 | 1 | 5 | 58.37 |
| CHEMBL3655703 | 702.8214 | -2.142 | 9 | 4 | 19 | 247.72 |
| CHEMBL3833404 | 180.1574 | 1.2021 | 2 | 0 | 3 | 63.6 |
| CHEMBL10372 | 419.3771 | 3.8485 | 3 | 0 | 5 | 113.32 |
| CHEMBL4297665 | 391.4198 | 4.1312 | 6 | 2 | 3 | 74.73 |
| CHEMBL508303 | 2450.934 | -2.2158 | 36 | 16 | 120 | 777.77 |
| CHEMBL359570 | 375.4635 | 3.7499 | 3 | 1 | 5 | 58.37 |
| CHEMBL2103768 | 234.0793 | 2.084 | 2 | 1 | 2 | 40.54 |
| CHEMBL95058 | 572.1463 | -2.5878 | 19 | 2 | 10 | 338.88 |
| CHEMBL2107064 | 240.2573 | 1.5597 | 1 | 0 | 2 | 55.12 |
| CHEMBL2106751 | 583.5909 | 1.3324 | 7 | 2 | 14 | 201.57 |
| CHEMBL577 | 348.3936 | 1.539 | 1 | 0 | 8 | 106.94 |
| CHEMBL300841 | 448.5557 | 2.2576 | 2 | 0 | 9 | 160.48 |
| CHEMBL46423 | 421.6181 | 7.2023 | 1 | 2 | 7 | 44.37 |
| CHEMBL186537 | 43.028 | -1.5475 | 0 | 1 | 0 | 23.85 |
| CHEMBL1201236 | 226.2292 | 0.2051 | 2 | 4 | 4 | 115.81 |
| CHEMBL2111119 | 239.7411 | 3.5674 | 1 | 0 | 2 | 12.47 |
| CHEMBL2074730 | 563.6429 | 5.7481 | 5 | 2 | 8 | 92.89 |
| CHEMBL2111075 | 168.2114 | 1.1916 | 2 | 0 | 5 | 71.98 |
| CHEMBL4300558 | 365.4456 | 0.567 | 3 | 0 | 3 | 79.68 |
| CHEMBL25892 | 452.5062 | 2.3464 | 7 | 2 | 8 | 146 |
| CHEMBL2373151 | 138.9369 | 1.3235 | 3 | 3 | 1 | 60.69 |
| CHEMBL1233800 | 325.3651 | 3.1571 | 4 | 3 | 4 | 101.63 |
| CHEMBL142715 | 425.605 | 7.5063 | 1 | 0 | 7 | 12.47 |
| CHEMBL4298148 | 529.9183 | 2.8325 | 4 | 3 | 6 | 130.47 |
| CHEMBL2103797 | 22.9898 | 0 | 0 | 0 | 0 | 0 |
| CHEMBL581 | 435.4935 | 4.2246 | 2 | 0 | 9 | 104.72 |
| CHEMBL223824 | 536.6639 | 5.1683 | 3 | 4 | 8 | 116.05 |
| CHEMBL3661412 | 207.1828 | 0.9531 | 2 | 0 | 3 | 72.74 |
| CHEMBL520358 | 358.4363 | 4.2223 | 2 | 2 | 4 | 87.05 |
| CHEMBL3655687 | 633.8007 | 3.7612 | 6 | 3 | 14 | 163.06 |
| CHEMBL1243298 | 377.4794 | 4.8664 | 2 | 0 | 2 | 36.02 |
| CHEMBL1233897 | 381.3673 | 3.1581 | 2 | 1 | 5 | 45.23 |
| CHEMBL1192519 | 396.4364 | 2.7013 | 1 | 0 | 8 | 106.94 |
| CHEMBL600689 | 301.3801 | 3.9793 | 3 | 1 | 7 | 51.32 |
| CHEMBL4594378 | 414.7366 | 5.1491 | 4 | 2 | 7 | 75.62 |
| CHEMBL3931782 | 313.2947 | 0.0415 | 6 | 0 | 3 | 109.84 |
| CHEMBL134920 | 246.2254 | 0.7807 | 3 | 1 | 3 | 112.76 |
| CHEMBL113841 | 705.9711 | 3.2671 | 7 | 4 | 17 | 188.79 |
| CHEMBL3655695 | 645.8545 | 4.8292 | 5 | 3 | 13 | 153.83 |
| CHEMBL63440 | 342.4305 | 1.6118 | 5 | 2 | 11 | 97.03 |
| CHEMBL3655701 | 687.8499 | 0.0556 | 8 | 4 | 20 | 227.41 |
| CHEMBL3651708 | 347.4319 | 2.3332 | 5 | 0 | 4 | 79.67 |
| CHEMBL2219411 | 414.4598 | 2.8579 | 5 | 2 | 7 | 103.17 |
| CHEMBL2105677 | 542.0265 | 3.817 | 7 | 1 | 8 | 90.44 |
| CHEMBL75013 | 376.5344 | 5.2774 | 1 | 1 | 6 | 32.34 |
| CHEMBL2105197 | 398.5169 | 2.2786 | 4 | 1 | 8 | 129.08 |
| CHEMBL8706 | 272.1703 | 4.5357 | 1 | 1 | 7 | 12.47 |
| CHEMBL1090089 | 406.3833 | 2.2638 | 6 | 3 | 8 | 109.5 |
| CHEMBL3694253 | 298.4026 | 3.9544 | 1 | 1 | 4 | 62.27 |
| CHEMBL564131 | 401.8899 | 2.9697 | 4 | 2 | 7 | 99.81 |
| CHEMBL2105657 | 464.5864 | 4.1199 | 4 | 3 | 7 | 127.37 |
| CHEMBL518179 | 577.7375 | 3.033 | 5 | 2 | 12 | 159.98 |
| CHEMBL460810 | 561.7381 | 3.643 | 4 | 2 | 11 | 150.75 |
| CHEMBL2170434 | 374.3166 | 4.761 | 2 | 2 | 6 | 87.14 |
| CHEMBL2105855 | 634.8882 | 10.1907 | 5 | 2 | 14 | 88.1 |
| CHEMBL1082354 | 255.2736 | 1.5917 | 2 | 1 | 3 | 141.61 |
| CHEMBL4298156 | 468.5652 | 5.8876 | 3 | 1 | 9 | 59.07 |
| CHEMBL2104975 | 717.2799 | 7.0641 | 7 | 0 | 12 | 130 |
| CHEMBL69998 | 336.4027 | 2.297 | 5 | 0 | 5 | 78.05 |
| CHEMBL3582252 | 475.576 | 5.9512 | 3 | 0 | 9 | 68.23 |
| CHEMBL2219421 | 336.79 | 3.1504 | 2 | 0 | 5 | 79.82 |
| CHEMBL3039506 | 469.5365 | 6.8042 | 4 | 0 | 4 | 76.5 |
| CHEMBL4293433 | 449.6713 | 1.6874 | 3 | 2 | 4 | 72.88 |
| CHEMBL3969876 | 499.6455 | 1.9143 | 5 | 0 | 10 | 88.93 |
| CHEMBL1232829 | 741.448 | 5.2795 | 8 | 3 | 9 | 200.67 |
| CHEMBL2103790 | 416.4708 | 1.5959 | 5 | 2 | 4 | 102.42 |
| CHEMBL3640071 | 442.5512 | 1.1814 | 4 | 1 | 6 | 82.19 |
| CHEMBL2103843 | 519.617 | 1.2781 | 8 | 1 | 10 | 143.92 |
| CHEMBL185238 | 287.3186 | 2.7219 | 4 | 1 | 2 | 67.35 |
| CHEMBL307429 | 336.4027 | 2.297 | 5 | 0 | 5 | 78.05 |
| CHEMBL3644461 | 404.4007 | 2.5993 | 2 | 1 | 3 | 83.24 |
| CHEMBL3644465 | 351.4141 | 1.8095 | 3 | 0 | 2 | 80.59 |
| CHEMBL356066 | 379.4522 | 2.3972 | 3 | 4 | 9 | 88.59 |
| CHEMBL2111088 | 385.4402 | 2.5511 | 4 | 3 | 6 | 147.43 |
| CHEMBL461655 | 533.6849 | 2.7006 | 4 | 2 | 10 | 150.75 |
| CHEMBL2105653 | 463.3817 | 4.6103 | 6 | 2 | 7 | 101.39 |
| CHEMBL19215 | 403.5167 | 4.2163 | 2 | 1 | 5 | 46.5 |
| CHEMBL414 | 201.1998 | 0.1082 | 2 | 1 | 2 | 105.84 |
| CHEMBL1201600 | 163.1717 | -2.44 | 4 | 4 | 1 | 92.95 |
| CHEMBL2218882 | 322.3363 | 2.2021 | 4 | 1 | 6 | 74.73 |
| CHEMBL2107099 | 213.1735 | -0.2737 | 1 | 3 | 2 | 108.02 |
| CHEMBL16596 | 587.1713 | 8.7733 | 2 | 0 | 10 | 89.65 |
| CHEMBL2107228 | 408.4919 | 1.5903 | 6 | 4 | 10 | 129.65 |
| CHEMBL572964 | 98.0785 | -0.6578 | 2 | 0 | 0 | 82.98 |
| CHEMBL35084 | 515.6383 | 4.6217 | 5 | 1 | 13 | 111.16 |
| CHEMBL1788385 | 238.0806 | 2.9835 | 2 | 1 | 0 | 33.12 |
| CHEMBL3694254 | 316.441 | 3.9119 | 1 | 1 | 3 | 85.88 |
| CHEMBL2106094 | 251.3263 | 3.6286 | 1 | 0 | 4 | 21.06 |
| CHEMBL3039500 | 633.6882 | 4.1732 | 5 | 3 | 8 | 119.14 |
| CHEMBL3989734 | 453.5191 | -0.1764 | 8 | 1 | 8 | 230 |
| CHEMBL3990346 | 533.7463 | 3.5524 | 4 | 4 | 15 | 122.8 |
| CHEMBL69139 | 384.472 | 2.3133 | 4 | 0 | 6 | 74.24 |
| CHEMBL4541225 | 494.4867 | 2.226 | 4 | 0 | 7 | 132.36 |
| CHEMBL1788393 | 427.2785 | 4.0308 | 3 | 0 | 10 | 81.91 |
| CHEMBL187379 | 616.7471 | 4.4915 | 6 | 5 | 17 | 159.85 |
| CHEMBL1908306 | 469.7222 | 9.32 | 3 | 2 | 15 | 74.63 |
| CHEMBL355001 | 96.109 | -1.925 | 2 | 2 | 0 | 94.56 |
| CHEMBL10413 | 441.6356 | 4.8783 | 2 | 0 | 4 | 81.3 |
| CHEMBL13134 | 708.8888 | 4.0019 | 7 | 4 | 15 | 145.78 |
| CHEMBL3694251 | 261.3178 | 4.5431 | 2 | 1 | 2 | 33.12 |
| CHEMBL269026 | 1306.424 | 1.6901 | 13 | 9 | 32 | 433.62 |
| CHEMBL279786 | 477.6399 | 3.2462 | 4 | 4 | 12 | 161.07 |
| CHEMBL3426621 | 323.3857 | 0.7716 | 3 | 0 | 2 | 70.68 |
| CHEMBL3707269 | 380.3676 | 2.2733 | 3 | 2 | 4 | 78.84 |
| CHEMBL2104845 | 335.4427 | 5.5079 | 3 | 1 | 5 | 50.95 |
| CHEMBL3644463 | 411.4445 | 0.8259 | 3 | 1 | 4 | 115.47 |
| CHEMBL2170435 | 385.2147 | 4.5671 | 2 | 2 | 5 | 87.14 |
| CHEMBL155760 | 574.7055 | 7.0742 | 8 | 1 | 15 | 99.16 |
| CHEMBL290001 | 721.9904 | 4.5619 | 8 | 6 | 22 | 200.26 |
| CHEMBL4647810 | 375.427 | 2.1099 | 5 | 1 | 3 | 84.75 |
| CHEMBL1817841 | 121.092 | -0.3398 | 2 | 1 | 4 | 72.6 |
| CHEMBL188203 | 730.8895 | 5.7631 | 8 | 5 | 21 | 172.16 |
| CHEMBL1965985 | 325.3651 | 3.1571 | 4 | 3 | 4 | 101.63 |
| CHEMBL3651707 | 396.4364 | 3.4156 | 6 | 1 | 8 | 82.81 |
| CHEMBL3651709 | 428.9086 | 4.5139 | 4 | 2 | 8 | 72.72 |
| CHEMBL2107785 | 435.5736 | 3.3482 | 4 | 2 | 11 | 106.89 |
| CHEMBL499274 | 572.7209 | 2.4783 | 4 | 3 | 10 | 166.54 |
| CHEMBL2104968 | 413.794 | 4.0368 | 4 | 2 | 5 | 118.78 |
| CHEMBL116 | 505.6269 | 2.6238 | 6 | 3 | 12 | 139.57 |
| CHEMBL4297430 | 365.4456 | 0.567 | 3 | 0 | 3 | 79.68 |
| CHEMBL441738 | 1646.845 | -4.2342 | 19 | 19 | 51 | 642.98 |
| CHEMBL1765292 | 295.3325 | 3.132 | 1 | 0 | 4 | 57.61 |
| CHEMBL865 | 314.359 | 2.5235 | 4 | 1 | 3 | 94.57 |
| CHEMBL1502 | 383.3698 | 2.7221 | 5 | 1 | 7 | 117.62 |
| CHEMBL334966 | 776.3592 | 1.6805 | 15 | 3 | 16 | 335.94 |
| CHEMBL2220486 | 443.4944 | 3.1771 | 5 | 1 | 7 | 95.7 |
| CHEMBL3402762 | 492.5716 | 4.0378 | 6 | 0 | 7 | 96.93 |
| CHEMBL4437054 | 500.9217 | 4.7956 | 4 | 0 | 8 | 87.27 |
| CHEMBL3989516 | 578.4436 | 2.2469 | 10 | 2 | 10 | 202.19 |
| CHEMBL198362 | 435.8813 | 2.6071 | 5 | 1 | 5 | 116.42 |
| CHEMBL1614701 | 457.6814 | 3.1892 | 4 | 3 | 6 | 88.41 |
| CHEMBL4558324 | 554.6428 | 3.9854 | 6 | 2 | 10 | 109.67 |
| CHEMBL1873475 | 440.4971 | 3.4533 | 5 | 1 | 5 | 99.16 |
| CHEMBL56337 | 319.3986 | 3.4176 | 2 | 0 | 4 | 115 |
| CHEMBL68253 | 97.0937 | -1.2914 | 1 | 1 | 0 | 88.77 |
| CHEMBL48361 | 471.5111 | 2.2032 | 4 | 2 | 9 | 150.22 |
| CHEMBL608533 | 570.6371 | 5.0987 | 4 | 1 | 3 | 77.73 |
| CHEMBL4297865 | 487.5287 | 4.143 | 4 | 3 | 7 | 98.41 |
| CHEMBL1908371 | 1520.269 | -13.7759 | 24 | 0 | 35 | 760.84 |
| CHEMBL1278118 | 441.4555 | 4.4826 | 6 | 1 | 5 | 89.05 |
| CHEMBL4802161 | 446.5447 | 4.1341 | 5 | 1 | 5 | 101.8 |
| CHEMBL4065122 | 664.7965 | 4.025 | 6 | 2 | 7 | 120.99 |
| CHEMBL1618279 | 362.4432 | 5.8364 | 2 | 1 | 3 | 41.05 |
| CHEMBL1615025 | 504.2267 | 2.5684 | 5 | 3 | 5 | 109.38 |
| CHEMBL3989867 | 448.372 | 1.3722 | 4 | 1 | 3 | 103.7 |
| CHEMBL1171829 | 543.455 | 4.3069 | 5 | 0 | 7 | 88.19 |
| CHEMBL4297618 | 718.7149 | 6.3133 | 8 | 1 | 17 | 128.31 |
| CHEMBL607707 | 467.9231 | 4.3486 | 4 | 2 | 8 | 90.28 |
| CHEMBL1201162 | 324.44 | 0.5845 | 4 | 1 | 3 | 151.33 |
| CHEMBL3301600 | 423.4402 | 3.9838 | 5 | 3 | 10 | 97.4 |
| CHEMBL1201198 | 228.2101 | 1.3811 | 5 | 0 | 1 | 89.35 |
| CHEMBL281398 | 405.4928 | 3.5181 | 3 | 0 | 10 | 110.4 |
| CHEMBL2103772 | 385.4766 | 3.1288 | 5 | 1 | 11 | 97.77 |
| CHEMBL4227736 | 381.5078 | 4.9567 | 3 | 1 | 7 | 49.77 |
| CHEMBL4650361 | 405.468 | 4.0081 | 3 | 1 | 7 | 77.99 |
| CHEMBL3414626 | 562.7063 | 3.097 | 7 | 4 | 9 | 151.23 |
| CHEMBL3809489 | 506.6446 | 6.3743 | 4 | 2 | 4 | 97.78 |
| CHEMBL415049 | 587.5398 | 3.5071 | 9 | 3 | 15 | 184.63 |
| CHEMBL4594272 | 562.5607 | 5.3303 | 3 | 1 | 11 | 108.76 |
| CHEMBL2103855 | 546.9288 | 4.6391 | 4 | 1 | 9 | 142.17 |
| CHEMBL4297216 | 524.6781 | 5.1262 | 5 | 3 | 11 | 116.86 |
| CHEMBL4594271 | 562.5607 | 5.3303 | 3 | 1 | 11 | 108.76 |
| CHEMBL3125702 | 568.5522 | 6.161 | 3 | 0 | 9 | 100.13 |
| CHEMBL513818 | 568.13 | 3.365 | 4 | 2 | 10 | 150.75 |
| CHEMBL1650595 | 460.4505 | 4.8792 | 2 | 1 | 5 | 72.94 |
| CHEMBL4594290 | 389.2537 | 1.6394 | 3 | 2 | 7 | 133.91 |
| CHEMBL3414621 | 572.7376 | 4.0547 | 4 | 2 | 9 | 86.9 |
| CHEMBL1182714 | 464.8201 | 3.6591 | 4 | 1 | 9 | 76.89 |
| CHEMBL446785 | 976.9646 | -2.722 | 24 | 14 | 20 | 391.2 |
| CHEMBL408513 | 318.3477 | 1.6683 | 4 | 3 | 5 | 103.88 |
| CHEMBL2074689 | 527.6042 | 4.6991 | 3 | 1 | 6 | 48.83 |
| CHEMBL1232111 | 275.3444 | 4.7724 | 1 | 1 | 4 | 12.47 |
| CHEMBL3545413 | 562.5888 | 3.8086 | 5 | 2 | 8 | 99.17 |
| CHEMBL332750 | 370.4222 | 3.9508 | 4 | 0 | 7 | 109.78 |
| CHEMBL163 | 720.9442 | 5.664 | 7 | 4 | 18 | 202.26 |
| CHEMBL126159 | 452.4566 | 2.161 | 7 | 1 | 8 | 108.25 |
| CHEMBL157138 | 338.4466 | 2.8057 | 1 | 2 | 3 | 51.37 |
| CHEMBL2105662 | 693.7204 | 7.7908 | 2 | 2 | 12 | 61.44 |
| CHEMBL436 | 357.3475 | 3.958 | 1 | 0 | 6 | 81.86 |
| CHEMBL2103863 | 397.4244 | 2.238 | 5 | 3 | 8 | 104.04 |
| CHEMBL4297489 | 403.52 | 2.6048 | 2 | 2 | 5 | 76.18 |
| CHEMBL576982 | 560.6672 | 6.349 | 6 | 2 | 8 | 134.92 |
| CHEMBL1946170 | 482.8154 | 5.3261 | 4 | 3 | 6 | 92.35 |
| CHEMBL4650360 | 451.4685 | 3.8074 | 4 | 0 | 4 | 97.17 |
| CHEMBL1200862 | 195.2151 | 0.8929 | 1 | 1 | 3 | 83.55 |
| CHEMBL506871 | 244.2923 | 0.6219 | 2 | 2 | 2 | 83.8 |
| CHEMBL673 | 159.2276 | 3.0958 | 0 | 1 | 4 | 3.24 |
| CHEMBL3545123 | 1113.18 | 8.2373 | 10 | 4 | 17 | 199.58 |
| CHEMBL2017179 | 638.647 | 8.5054 | 3 | 0 | 9 | 87.38 |
| CHEMBL3936761 | 471.5509 | 3.2868 | 4 | 2 | 6 | 102.48 |
| CHEMBL1922235 | 474.5515 | 3.2975 | 4 | 3 | 6 | 94.14 |
| CHEMBL3188267 | 412.4191 | 2.8713 | 5 | 1 | 4 | 85.59 |
| CHEMBL4297647 | 897.0467 | 6.7753 | 12 | 3 | 11 | 216.74 |
| CHEMBL1631694 | 279.3149 | 2.4792 | 2 | 0 | 5 | 95.49 |
| CHEMBL4559134 | 525.6015 | 2.8895 | 7 | 1 | 8 | 112.56 |
| CHEMBL1439 | 299.2831 | -2.0736 | 5 | 0 | 3 | 133.67 |
| CHEMBL295392 | 391.4614 | 1.5387 | 5 | 4 | 8 | 116.76 |
| CHEMBL2106126 | 452.5427 | 3.5505 | 3 | 0 | 12 | 95.94 |
| CHEMBL2369717 | 1587.896 | 2.874 | 16 | 16 | 54 | 548.57 |
| CHEMBL42583 | 584.6822 | 0.658 | 5 | 4 | 17 | 233.6 |
| CHEMBL4298155 | 432.8791 | 2.0639 | 5 | 2 | 5 | 107.95 |
| CHEMBL1552586 | 403.8808 | 4.7826 | 2 | 2 | 3 | 124.49 |
| CHEMBL277592 | 365.3695 | 5.3132 | 3 | 0 | 5 | 59.59 |
| CHEMBL4298153 | 489.6441 | 4.8634 | 2 | 1 | 11 | 70.98 |
| CHEMBL4802241 | 383.4276 | -0.5792 | 6 | 1 | 5 | 128.94 |
| CHEMBL2104380 | 729.9263 | 1.8512 | 9 | 5 | 17 | 199.4 |
| CHEMBL2104854 | 422.606 | 6.1172 | 2 | 0 | 3 | 86.74 |
| CHEMBL4650367 | 441.4803 | -0.2308 | 6 | 3 | 12 | 117.36 |
| CHEMBL325441 | 439.3833 | 6.8139 | 2 | 0 | 7 | 68.53 |
| CHEMBL4594276 | 460.5315 | 2.4335 | 6 | 3 | 6 | 134.06 |
| CHEMBL182319 | 473.648 | 4.1479 | 2 | 1 | 11 | 98.74 |
| CHEMBL361727 | 622.8594 | 4.1749 | 7 | 5 | 19 | 171.16 |
| CHEMBL309962 | 313.3925 | 1.6669 | 3 | 2 | 4 | 141.94 |
| CHEMBL1516410 | 385.4766 | 3.1288 | 5 | 1 | 11 | 97.77 |
| CHEMBL2170427 | 324.3091 | 4.0242 | 2 | 2 | 5 | 87.14 |
| CHEMBL3989968 | 446.4439 | 4.3246 | 6 | 1 | 10 | 82.82 |
| CHEMBL3644455 | 426.4989 | 2.0465 | 3 | 1 | 4 | 92.61 |
| CHEMBL1929396 | 383.4475 | -0.7192 | 5 | 1 | 6 | 115.94 |
| CHEMBL539697 | 627.7332 | 5.6773 | 8 | 1 | 17 | 154.03 |
| CHEMBL2147777 | 426.5782 | 1.8581 | 3 | 0 | 4 | 81.94 |
| CHEMBL1096380 | 336.3877 | 1.3232 | 3 | 3 | 3 | 94.4 |
| CHEMBL3707347 | 348.4182 | 3.7789 | 2 | 0 | 4 | 99.28 |
| CHEMBL3137318 | 380.3509 | 2.0488 | 4 | 2 | 2 | 88.49 |
| CHEMBL276711 | 238.2845 | 2.3899 | 1 | 2 | 1 | 44.89 |
| CHEMBL1492500 | 295.4417 | 4.4665 | 0 | 0 | 3 | 28.54 |
| CHEMBL3989959 | 324.3109 | -1.447 | 5 | 1 | 6 | 159.88 |
| CHEMBL1628227 | 279.3761 | 3.9083 | 1 | 0 | 3 | 12.47 |
| CHEMBL512351 | 451.9055 | 3.4317 | 5 | 3 | 7 | 107.41 |
| CHEMBL471498 | 315.4067 | 2.9909 | 1 | 0 | 5 | 57.61 |
| CHEMBL4297582 | 495.6105 | 4.346 | 5 | 3 | 15 | 116.76 |
| CHEMBL119385 | 436.2621 | 6.4538 | 3 | 0 | 3 | 73.08 |
| CHEMBL396778 | 302.3434 | 2.447 | 2 | 1 | 7 | 64.35 |
| CHEMBL443684 | 974.6127 | 10.4952 | 6 | 2 | 17 | 170.42 |
| CHEMBL1201314 | 354.3617 | -1.1507 | 7 | 3 | 9 | 171.37 |
| CHEMBL4554795 | 282.314 | 1.0634 | 3 | 2 | 4 | 103.26 |
| CHEMBL193093 | 224.3026 | 1.0244 | 1 | 2 | 2 | 125.59 |
| CHEMBL21485 | 121.9296 | 1.7838 | 2 | 2 | 1 | 40.46 |
| CHEMBL2105776 | 428.5444 | 3.2338 | 3 | 0 | 8 | 128.11 |
| CHEMBL138225 | 399.5662 | 4.3909 | 1 | 1 | 3 | 66.4 |
| CHEMBL144613 | 361.3476 | 0.2062 | 4 | 2 | 3 | 174.83 |
| CHEMBL3651710 | 421.3665 | 5.5131 | 5 | 1 | 8 | 69.92 |
| CHEMBL4650217 | 502.5616 | 3.4906 | 5 | 0 | 5 | 114.62 |
| CHEMBL28527 | 385.3361 | 3.7829 | 3 | 1 | 4 | 86.36 |
| CHEMBL519123 | 321.33 | 1.8794 | 4 | 2 | 3 | 76.24 |
| CHEMBL3143751 | 633.8191 | 2.5403 | 7 | 5 | 19 | 188.95 |
| CHEMBL3666064 | 415.8436 | 5.2622 | 2 | 2 | 3 | 96.25 |
| CHEMBL4303198 | 405.3392 | 2.4413 | 3 | 2 | 7 | 97.44 |
| CHEMBL138029 | 449.5636 | 3.961 | 5 | 0 | 11 | 133.6 |
| CHEMBL2103833 | 396.4579 | 2.4032 | 2 | 3 | 5 | 77.23 |
| CHEMBL203665 | 540.6312 | 2.7585 | 7 | 2 | 8 | 147.06 |
| CHEMBL2105727 | 397.4244 | 2.238 | 5 | 3 | 8 | 104.04 |
| CHEMBL516786 | 568.13 | 3.365 | 4 | 2 | 10 | 150.75 |
| CHEMBL3661406 | 302.3664 | 4.1012 | 2 | 1 | 4 | 32.76 |
| CHEMBL3644459 | 379.4275 | 0.9913 | 3 | 2 | 3 | 126.33 |
| CHEMBL3644457 | 428.49 | 2.9865 | 2 | 0 | 4 | 72.38 |
| CHEMBL3661417 | 300.3505 | 4.391 | 2 | 0 | 4 | 29.6 |
| CHEMBL359891 | 640.7669 | 3.8122 | 8 | 5 | 19 | 172.16 |
| CHEMBL318779 | 401.5639 | 1.2403 | 5 | 3 | 13 | 129.67 |
| CHEMBL536282 | 295.3508 | 2.8387 | 1 | 1 | 2 | 82.64 |
| CHEMBL296588 | 685.8921 | 2.276 | 7 | 7 | 22 | 223.26 |
| CHEMBL430554 | 363.4082 | 0.61 | 1 | 0 | 9 | 132.96 |
| CHEMBL3143753 | 801.9674 | 5.3071 | 9 | 6 | 23 | 201.26 |
| CHEMBL4447328 | 431.487 | 2.7613 | 4 | 2 | 6 | 116.47 |
| CHEMBL4569451 | 266.2548 | 1.8075 | 3 | 0 | 3 | 80.9 |
| CHEMBL4298140 | 340.3797 | 2.4104 | 4 | 3 | 2 | 114.93 |
| CHEMBL1201368 | 340.4146 | 1.8119 | 1 | 0 | 7 | 106.94 |
| CHEMBL502124 | 588.0749 | 5.6238 | 4 | 3 | 9 | 102.49 |
| CHEMBL3392776 | 471.5243 | 5.1017 | 5 | 3 | 5 | 104.24 |
| CHEMBL3143750 | 591.7394 | 1.3761 | 7 | 5 | 17 | 188.95 |
| CHEMBL466588 | 568.13 | 3.365 | 4 | 2 | 10 | 150.75 |
| CHEMBL3644462 | 412.4987 | 3.1754 | 2 | 1 | 3 | 83.24 |
| CHEMBL3661418 | 274.3133 | 3.781 | 2 | 0 | 3 | 29.6 |
| CHEMBL409450 | 356.1735 | 2.9216 | 3 | 3 | 0 | 73.72 |
| CHEMBL3655690 | 610.7674 | 1.9483 | 6 | 3 | 12 | 174.14 |
| CHEMBL527026 | 539.4347 | 5.0467 | 4 | 2 | 7 | 94.69 |
| CHEMBL96862 | 332.2066 | -1.7484 | 10 | 2 | 4 | 169.86 |
| CHEMBL4298146 | 478.4227 | 3.9612 | 5 | 2 | 4 | 89.13 |
| CHEMBL4104957 | 561.9832 | 5.4642 | 3 | 1 | 7 | 113.6 |
| CHEMBL3661416 | 242.245 | 3.0781 | 2 | 0 | 3 | 29.6 |
| CHEMBL28636 | 397.3716 | 3.2776 | 4 | 1 | 5 | 95.59 |
| CHEMBL4594270 | 389.4869 | 3.746 | 3 | 1 | 5 | 82.7 |
| CHEMBL1162175 | 532.4431 | 1.812 | 11 | 2 | 8 | 188.99 |
| CHEMBL3661402 | 239.2692 | 3.0198 | 1 | 0 | 4 | 41.96 |
| CHEMBL3646118 | 314.3027 | 0.1417 | 6 | 1 | 3 | 106.75 |
| CHEMBL2397013 | 287.3153 | 1.9889 | 2 | 2 | 2 | 57.78 |
| CHEMBL3655698 | 609.7826 | 1.5405 | 6 | 3 | 12 | 192.21 |
| CHEMBL60550 | 620.8004 | 3.5268 | 8 | 4 | 15 | 171.6 |
| CHEMBL3640070 | 400.5112 | 2.4623 | 4 | 1 | 6 | 75.71 |
| CHEMBL3661411 | 316.393 | 4.5874 | 2 | 1 | 4 | 32.76 |
| CHEMBL2106525 | 327.2488 | 3.8166 | 2 | 0 | 3 | 24.83 |
| CHEMBL531048 | 210.1882 | 1.3642 | 4 | 0 | 0 | 59.92 |
| CHEMBL535834 | 429.9629 | 3.7148 | 3 | 1 | 8 | 87.6 |
| CHEMBL1877495 | 240.3003 | 2.5787 | 1 | 2 | 4 | 41.13 |
| CHEMBL1231461 | 80.9119 | -0.1036 | 0 | 0 | 0 | 0 |
| CHEMBL3186534 | 397.4723 | 3.5814 | 4 | 2 | 5 | 90.7 |
| CHEMBL453539 | 1513.783 | -4.2262 | 17 | 14 | 19 | 597.31 |
| CHEMBL4570801 | 462.9033 | 4.9293 | 4 | 1 | 3 | 69.56 |
| CHEMBL3675606 | 565.6373 | 2.4959 | 3 | 3 | 15 | 204.13 |
| CHEMBL3989427 | 381.5509 | 5.4271 | 2 | 1 | 7 | 32.7 |
| CHEMBL13608 | 396.4579 | 2.4032 | 2 | 3 | 5 | 77.23 |
| CHEMBL9298 | 223.2731 | 2.2261 | 2 | 0 | 2 | 41.61 |
| CHEMBL4297518 | 531.6675 | 3.2069 | 6 | 2 | 11 | 129.14 |
| CHEMBL3655697 | 605.7509 | 2.0734 | 6 | 4 | 12 | 192.74 |
| CHEMBL3666065 | 294.3047 | 2.2675 | 4 | 1 | 4 | 67.76 |
| CHEMBL2105901 | 284.396 | 2.7736 | 1 | 0 | 4 | 23.55 |
| CHEMBL3707394 | 342.3642 | 2.0842 | 3 | 1 | 5 | 58.64 |
| CHEMBL3644453 | 370.3926 | 1.0034 | 1 | 1 | 2 | 106.3 |
| CHEMBL3655692 | 633.8007 | 4.5844 | 6 | 4 | 14 | 160.07 |
| CHEMBL325695 | 443.5127 | 3.3811 | 7 | 1 | 11 | 116.23 |
| CHEMBL300337 | 656.8557 | 2.1503 | 7 | 6 | 18 | 191.69 |
| CHEMBL408983 | 1101.257 | -2.1484 | 11 | 7 | 24 | 387.32 |
| CHEMBL1733 | 410.4629 | 2.7362 | 1 | 0 | 8 | 106.94 |
| CHEMBL258844 | 551.6404 | 6.2836 | 4 | 2 | 5 | 95.49 |
| CHEMBL258004 | 293.3663 | 1.918 | 2 | 1 | 3 | 54.77 |
| CHEMBL84446 | 279.2239 | -0.2559 | 4 | 3 | 1 | 110.52 |
| CHEMBL2105502 | 367.4415 | 1.7636 | 4 | 2 | 5 | 68.54 |
| CHEMBL18 | 258.3173 | 1.4165 | 4 | 1 | 3 | 118.9 |
| CHEMBL51386 | 894.0861 | 0.6866 | 9 | 8 | 28 | 323.83 |
| CHEMBL38120 | 237.3395 | 3.6675 | 0 | 0 | 2 | 12.03 |
| CHEMBL1788368 | 237.3395 | 3.6675 | 0 | 0 | 2 | 12.03 |
| CHEMBL249097 | 335.3599 | 3.4039 | 4 | 4 | 5 | 113.16 |
| CHEMBL4594281 | 516.0587 | 4.0266 | 4 | 2 | 8 | 102.08 |
| CHEMBL3410535 | 414.3887 | 3.0158 | 3 | 0 | 4 | 145.87 |
| CHEMBL4299851 | 442.3956 | 0.3614 | 3 | 3 | 13 | 195.02 |
| CHEMBL1922282 | 443.258 | 3.9774 | 4 | 2 | 5 | 137.08 |
| CHEMBL3655696 | 631.8279 | 4.623 | 5 | 3 | 13 | 153.83 |
| CHEMBL161702 | 313.3478 | 2.7154 | 2 | 1 | 7 | 77.84 |
| CHEMBL224325 | 228.0747 | 3.3603 | 2 | 1 | 0 | 33.12 |
| CHEMBL404609 | 261.2996 | 1.9383 | 2 | 3 | 3 | 126.45 |
| CHEMBL185198 | 43.0247 | -1.8054 | 0 | 0 | 0 | 27.42 |
| CHEMBL2104331 | 659.81 | 4.3525 | 9 | 4 | 20 | 153.68 |
| CHEMBL1098285 | 416.5139 | 4.5014 | 4 | 0 | 11 | 66.84 |
| CHEMBL1199307 | 416.5139 | 4.5014 | 4 | 0 | 11 | 66.84 |
| CHEMBL1200739 | 34.0809 | -0.2072 | 0 | 0 | 0 | 0 |
| CHEMBL28196 | 281.349 | 3.6336 | 1 | 0 | 7 | 50.19 |
| CHEMBL1788402 | 351.1177 | 1.6345 | 3 | 1 | 1 | 95.87 |
| CHEMBL143687 | 745.765 | 4.7552 | 9 | 5 | 17 | 180.36 |
| CHEMBL2104578 | 389.4455 | 1.7667 | 2 | 0 | 7 | 110.18 |
| CHEMBL2104660 | 385.4766 | 3.1288 | 5 | 1 | 11 | 97.77 |
| CHEMBL3661410 | 314.3771 | 4.8772 | 2 | 0 | 4 | 29.6 |
| CHEMBL2105586 | 260.7188 | 2.7687 | 1 | 0 | 0 | 34.03 |
| CHEMBL2107466 | 337.3692 | 3.6781 | 3 | 0 | 6 | 79.55 |
| CHEMBL3984425 | 528.564 | 3.1117 | 5 | 2 | 5 | 125.65 |
| CHEMBL160571 | 546.6524 | 6.1895 | 8 | 2 | 14 | 107.95 |
| CHEMBL2170597 | 306.3187 | 3.8187 | 2 | 2 | 5 | 87.14 |
| CHEMBL2106020 | 218.0479 | 1.2628 | 2 | 2 | 2 | 55.48 |
| CHEMBL1200563 | 458.5423 | 3.5015 | 4 | 2 | 11 | 102.71 |
| CHEMBL2107833 | 553.5868 | 6.0323 | 6 | 3 | 7 | 123.06 |
| CHEMBL4594299 | 688.7284 | 6.5709 | 10 | 1 | 11 | 143.07 |
| CHEMBL2106694 | 197.2244 | 2.1616 | 0 | 0 | 4 | 26.02 |
| CHEMBL2364627 | 420.5257 | 5.4723 | 3 | 2 | 5 | 85.41 |
| CHEMBL122079 | 436.5912 | 5.3822 | 1 | 1 | 6 | 35.16 |
| CHEMBL435176 | 420.4595 | -0.0763 | 7 | 3 | 10 | 141.05 |
| CHEMBL70611 | 429.9629 | 3.7148 | 3 | 1 | 8 | 87.6 |
| CHEMBL496594 | 553.4613 | 6.0992 | 4 | 2 | 8 | 94.69 |
| CHEMBL493116 | 543.6384 | -1.0778 | 6 | 4 | 11 | 222.94 |
| CHEMBL405395 | 1209.737 | 1.3407 | 12 | 10 | 32 | 421.62 |
| CHEMBL4298173 | 372.4629 | 4.7341 | 3 | 1 | 0 | 50.28 |
| CHEMBL4297623 | 428.2913 | 1.5922 | 5 | 1 | 6 | 99.58 |
| CHEMBL160839 | 532.6258 | 5.6658 | 8 | 2 | 13 | 107.95 |
| CHEMBL1733373 | 224.2546 | 2.8726 | 2 | 0 | 3 | 29.6 |
| CHEMBL491510 | 322.396 | 4.0155 | 5 | 3 | 0 | 86.99 |
| CHEMBL4594286 | 571.5741 | 5.6001 | 8 | 2 | 13 | 160.12 |
| CHEMBL4594293 | 465.5065 | 2.9024 | 5 | 2 | 5 | 119.03 |
| CHEMBL3989930 | 486.5886 | 5.118 | 4 | 2 | 7 | 110.86 |
| CHEMBL237352 | 439.5288 | 4.5546 | 3 | 1 | 5 | 99.16 |
| CHEMBL257167 | 279.2901 | 2.1438 | 2 | 3 | 3 | 126.45 |
| CHEMBL356301 | 428.5246 | 0.7498 | 2 | 1 | 4 | 101.98 |
| CHEMBL468419 | 223.2731 | 2.2261 | 2 | 0 | 2 | 41.61 |
| CHEMBL2364622 | 443.5193 | 1.1931 | 4 | 3 | 6 | 140.74 |
| CHEMBL17331 | 360.3412 | 4.0148 | 2 | 1 | 4 | 84.71 |
| CHEMBL476186 | 484.9073 | 4.1866 | 4 | 2 | 5 | 92.67 |
| CHEMBL1908845 | 1529.34 | -7.6757 | 31 | 0 | 35 | 735.37 |
| CHEMBL296602 | 292.203 | 4.5401 | 0 | 0 | 2 | 12.03 |
| CHEMBL2218897 | 386.4995 | 5.0961 | 3 | 1 | 3 | 46.53 |
| CHEMBL1084955 | 369.4358 | 4.1182 | 2 | 2 | 3 | 124.49 |
| CHEMBL2104358 | 464.6395 | 4.2857 | 3 | 2 | 4 | 67.43 |
| CHEMBL267585 | 1233.372 | 1.5882 | 13 | 8 | 32 | 417.83 |
| CHEMBL3661403 | 244.3089 | 3.0773 | 1 | 0 | 3 | 60.89 |
| CHEMBL297884 | 774.9254 | 5.8628 | 8 | 3 | 10 | 209.55 |
| CHEMBL3990145 | 499.6455 | 1.9143 | 5 | 0 | 10 | 88.93 |
| CHEMBL3984441 | 556.5842 | 4.5346 | 6 | 2 | 8 | 107.61 |
| CHEMBL2106789 | 302.2788 | 1.4 | 4 | 1 | 2 | 100.9 |
| CHEMBL3989450 | 388.4574 | 2.3879 | 1 | 0 | 8 | 106.94 |
| CHEMBL447977 | 416.5967 | 6.6261 | 1 | 1 | 11 | 69.64 |
| CHEMBL259389 | 185.1854 | 0.9328 | 3 | 2 | 0 | 80.48 |
| CHEMBL324954 | 416.5172 | 0.0645 | 4 | 1 | 11 | 142.91 |
| CHEMBL223448 | 302.9501 | 3.2457 | 2 | 1 | 0 | 33.12 |
| CHEMBL2105038 | 270.3263 | 2.5145 | 2 | 1 | 4 | 47.72 |
| CHEMBL3655694 | 611.7554 | 0.479 | 6 | 4 | 13 | 194.96 |
| CHEMBL2107365 | 255.7405 | 2.3186 | 2 | 1 | 1 | 41.49 |
| CHEMBL1598450 | 304.7316 | 3.2836 | 3 | 1 | 3 | 72.68 |
| CHEMBL3644464 | 350.4293 | 1.5111 | 2 | 1 | 2 | 83.24 |
| CHEMBL3644454 | 386.392 | 0.7817 | 3 | 1 | 2 | 100.46 |
| CHEMBL468270 | 528.7099 | 1.5217 | 4 | 2 | 13 | 176.77 |
| CHEMBL3655702 | 659.7967 | -0.7838 | 8 | 4 | 18 | 227.41 |
| CHEMBL3989889 | 749.2936 | 7.0836 | 8 | 0 | 18 | 111.16 |
| CHEMBL4594291 | 389.2537 | 1.6394 | 3 | 2 | 7 | 133.91 |
| CHEMBL3655699 | 634.7457 | 1.3903 | 7 | 3 | 14 | 206.15 |
| CHEMBL51934 | 214.2631 | 1.6881 | 2 | 2 | 0 | 59.14 |
| CHEMBL206335 | 528.4616 | 3.5336 | 5 | 2 | 7 | 120.03 |
| CHEMBL2107586 | 375.505 | 5.2155 | 3 | 1 | 8 | 54.04 |
| CHEMBL364773 | 511.6529 | 4.2953 | 5 | 4 | 14 | 130.75 |
| CHEMBL558198 | 528.4616 | 3.5336 | 5 | 2 | 7 | 120.03 |
| CHEMBL143239 | 717.7119 | 4.2532 | 5 | 5 | 15 | 202.36 |
| CHEMBL54440 | 390.5131 | 3.9663 | 5 | 1 | 7 | 72.83 |
| CHEMBL3039499 | 910.0804 | 5.8801 | 10 | 1 | 10 | 201.79 |
| CHEMBL3040582 | 911.0883 | 5.2652 | 10 | 2 | 10 | 213.82 |
| CHEMBL48092 | 232.2783 | 2.2607 | 1 | 0 | 6 | 55.12 |
| CHEMBL80937 | 299.7979 | 3.093 | 1 | 1 | 2 | 42.82 |
| CHEMBL1788396 | 460.1457 | 2.025 | 4 | 6 | 7 | 139.12 |
| CHEMBL189358 | 690.8704 | 2.9216 | 8 | 7 | 20 | 218.05 |
| CHEMBL1290459 | 328.1641 | 4.7555 | 2 | 0 | 4 | 55.76 |
| CHEMBL461939 | 409.4021 | 5.1706 | 2 | 0 | 4 | 115 |
| CHEMBL1204135 | 338.4666 | 1.05 | 4 | 1 | 4 | 151.33 |
| CHEMBL485569 | 372.4629 | 4.7085 | 2 | 2 | 4 | 87.05 |
| CHEMBL2106052 | 128.2153 | 1.1208 | 0 | 2 | 2 | 38.05 |
| CHEMBL2364604 | 410.3935 | 2.3477 | 5 | 1 | 4 | 89.63 |
| CHEMBL2104954 | 574.1394 | 5.3982 | 4 | 1 | 7 | 107.54 |
| CHEMBL283656 | 283.3251 | 1.5831 | 1 | 0 | 5 | 60.05 |
| CHEMBL42908 | 559.6876 | 5.3143 | 4 | 3 | 15 | 98.74 |
| CHEMBL3039533 | 766.9034 | 4.1725 | 11 | 3 | 8 | 203.6 |
| CHEMBL459177 | 358.4363 | 4.2223 | 2 | 2 | 4 | 87.05 |
| CHEMBL4297219 | 561.4354 | 4.2897 | 5 | 2 | 6 | 122.47 |
| CHEMBL6259 | 399.3907 | 3.8284 | 1 | 0 | 3 | 65.78 |
| CHEMBL3651712 | 445.959 | 5.247 | 4 | 1 | 8 | 85.99 |
| CHEMBL2107625 | 393.4788 | 2.344 | 4 | 1 | 4 | 63.16 |
| CHEMBL1201365 | 388.4574 | 2.3879 | 1 | 0 | 8 | 106.94 |
| CHEMBL101309 | 501.721 | 8.2277 | 2 | 1 | 10 | 80.85 |
| CHEMBL115653 | 436.545 | 2.1712 | 5 | 2 | 7 | 110.26 |
| CHEMBL4298137 | 277.2544 | -0.4294 | 4 | 1 | 3 | 138.62 |
| CHEMBL560910 | 257.4718 | 3.2629 | 1 | 0 | 3 | 46.53 |
| CHEMBL3661409 | 316.393 | 4.5097 | 2 | 0 | 5 | 21.76 |
| CHEMBL3661405 | 244.2609 | 2.7883 | 2 | 1 | 3 | 32.76 |
| CHEMBL4297502 | 513.5445 | 3.0329 | 5 | 3 | 10 | 167.49 |
| CHEMBL2364611 | 369.4192 | 2.7302 | 4 | 1 | 3 | 86.69 |
| CHEMBL3989948 | 412.5267 | 3.7109 | 4 | 0 | 3 | 72.86 |
| CHEMBL3545213 | 457.5474 | 4.1829 | 6 | 0 | 6 | 112.4 |
| CHEMBL460785 | 163.1717 | -2.44 | 4 | 4 | 1 | 92.95 |
| CHEMBL1834657 | 560.4754 | 6.4405 | 5 | 2 | 8 | 95.09 |
| CHEMBL507361 | 482.193 | 2.5787 | 4 | 4 | 7 | 90.82 |
| CHEMBL3963485 | 466.5559 | 2.2473 | 5 | 2 | 6 | 135.77 |
| CHEMBL4084907 | 411.3816 | 1.8944 | 6 | 1 | 4 | 102.52 |
| CHEMBL91238 | 242.702 | 1.8437 | 2 | 2 | 5 | 50.36 |
| CHEMBL1221693 | 505.5192 | 3.4191 | 7 | 3 | 13 | 132.06 |
| CHEMBL1614707 | 468.4709 | 5.373 | 5 | 0 | 11 | 65.97 |
| CHEMBL2105682 | 548.0575 | 0.8018 | 6 | 3 | 6 | 164.87 |
| CHEMBL1163 | 704.8555 | 5.481 | 9 | 5 | 18 | 171.22 |
| CHEMBL3544988 | 393.4342 | 1.7218 | 4 | 1 | 5 | 124.09 |
| CHEMBL2103849 | 516.6561 | 3.1765 | 6 | 2 | 11 | 130.59 |
| CHEMBL36591 | 255.7405 | 2.6953 | 2 | 0 | 5 | 52.32 |
| CHEMBL3644587 | 317.3446 | 2.1537 | 3 | 2 | 2 | 77.67 |
| CHEMBL21731 | 277.4033 | 4.1163 | 0 | 0 | 4 | 12.03 |
| CHEMBL1171837 | 532.5595 | 4.7935 | 3 | 1 | 6 | 66.29 |
| CHEMBL2107832 | 431.2008 | 1.1712 | 4 | 4 | 6 | 94.48 |
| CHEMBL1201650 | 601.8629 | 2.7759 | 5 | 2 | 9 | 140.76 |
| CHEMBL464758 | 963.0852 | -2.3532 | 10 | 10 | 31 | 410.73 |
| CHEMBL4283683 | 447.5095 | 2.0133 | 4 | 2 | 6 | 120.39 |
| CHEMBL86304 | 268.7393 | 1.5289 | 2 | 1 | 4 | 41.57 |
| CHEMBL6437 | 264.3648 | 3.7063 | 0 | 0 | 0 | 6.48 |
| CHEMBL549 | 324.3919 | 3.7214 | 2 | 0 | 6 | 36.26 |
| CHEMBL1095032 | 479.9769 | 1.7568 | 5 | 2 | 5 | 115.4 |
| CHEMBL4297334 | 441.3972 | 0.3614 | 3 | 3 | 13 | 195.02 |
| CHEMBL248183 | 454.4461 | 5.3221 | 3 | 2 | 10 | 128.23 |
| CHEMBL3675607 | 436.5234 | 3.2752 | 2 | 2 | 10 | 137.73 |
| CHEMBL287257 | 322.229 | 4.6422 | 1 | 0 | 2 | 12.47 |
| CHEMBL3889654 | 428.4352 | 2.8897 | 4 | 2 | 3 | 86.52 |
| CHEMBL124660 | 562.703 | 5.6739 | 6 | 1 | 10 | 92.29 |
| CHEMBL511115 | 343.4168 | 4.1487 | 3 | 0 | 4 | 79.55 |
| CHEMBL3545235 | 500.9497 | 4.5521 | 6 | 1 | 7 | 77.97 |
| CHEMBL4297662 | 601.8629 | 2.7759 | 5 | 2 | 9 | 140.76 |
| CHEMBL24828 | 475.354 | 5.0901 | 4 | 1 | 6 | 59.51 |
| CHEMBL4297595 | 515.567 | 1.8132 | 7 | 2 | 14 | 190.77 |
| CHEMBL4297676 | 431.2008 | 1.1712 | 4 | 4 | 6 | 94.48 |
| CHEMBL2364621 | 447.5328 | 3.5623 | 5 | 1 | 5 | 105.04 |
| CHEMBL668 | 263.3767 | 4.0415 | 0 | 0 | 4 | 12.03 |
| CHEMBL1201863 | 369.3615 | 3.1089 | 3 | 1 | 6 | 87.08 |
| CHEMBL3622820 | 553.5141 | 1.3412 | 6 | 1 | 6 | 119.62 |
| CHEMBL1200374 | 296.4034 | 3.1129 | 2 | 0 | 0 | 34.14 |
| CHEMBL2396661 | 441.4705 | 3.7468 | 4 | 2 | 5 | 129.45 |
| CHEMBL4297610 | 354.7903 | 3.495 | 3 | 2 | 3 | 90.68 |
| CHEMBL4297601 | 601.5954 | 9.7665 | 3 | 0 | 8 | 38.77 |
| CHEMBL3621988 | 390.4103 | 2.0757 | 3 | 3 | 6 | 97.11 |
| CHEMBL93 | 236.2902 | 2.5613 | 2 | 2 | 2 | 94.8 |
| CHEMBL1201179 | 581.0575 | 5.954 | 6 | 1 | 11 | 114.73 |
| CHEMBL435128 | 451.3694 | 5.3012 | 4 | 1 | 8 | 108.33 |
| CHEMBL2105763 | 394.4701 | 1.7324 | 4 | 2 | 6 | 95.31 |
| CHEMBL4303214 | 393.3927 | 3.0242 | 7 | 1 | 5 | 95.71 |
| CHEMBL3545414 | 397.4723 | 3.5814 | 4 | 2 | 5 | 90.7 |
| CHEMBL3785197 | 722.598 | 8.4867 | 5 | 0 | 15 | 105.09 |
| CHEMBL1566 | 645.6048 | -6.8381 | 18 | 13 | 9 | 321.17 |
| CHEMBL1220 | 247.2715 | 0.3846 | 3 | 0 | 5 | 103.48 |
| CHEMBL88712 | 361.4336 | 5.149 | 2 | 0 | 6 | 59.42 |
| CHEMBL1207745 | 398.492 | 3.8916 | 3 | 2 | 9 | 86.99 |
| CHEMBL869 | 198.1362 | 1.1366 | 3 | 2 | 3 | 123.76 |
| CHEMBL3989926 | 629.6763 | 4.9386 | 6 | 2 | 12 | 142.71 |
| CHEMBL2218861 | 395.5593 | 2.5977 | 3 | 2 | 7 | 81.26 |
| CHEMBL1983268 | 560.6375 | 4.6218 | 3 | 3 | 7 | 85.52 |
| CHEMBL1200904 | 187.2808 | 3.7945 | 0 | 1 | 5 | 3.24 |
| CHEMBL408194 | 317.3844 | 3.0135 | 2 | 2 | 3 | 53.17 |
| CHEMBL1520 | 488.603 | 2.1446 | 6 | 1 | 8 | 121.8 |
| CHEMBL502 | 379.492 | 4.5697 | 3 | 0 | 6 | 38.77 |
| CHEMBL50588 | 480.6389 | 4.9455 | 4 | 0 | 7 | 52.19 |
| CHEMBL1231821 | 36.4609 | -0.1036 | 0 | 0 | 0 | 0 |
| CHEMBL834 | 235.0695 | -2.8932 | 5 | 1 | 4 | 180.93 |
| CHEMBL38458 | 397.537 | 3.2945 | 2 | 2 | 8 | 107.58 |
| CHEMBL4594440 | 401.4727 | 4.9141 | 1 | 1 | 6 | 74.07 |
| CHEMBL137 | 171.154 | -0.1016 | 2 | 1 | 3 | 81.19 |
| CHEMBL1200644 | 1498.184 | -18.9309 | 21 | 9 | 30 | 900.82 |
| CHEMBL1628504 | 366.4185 | 5.3056 | 4 | 2 | 6 | 97.42 |
| CHEMBL4297527 | 440.1846 | 1.1706 | 4 | 1 | 4 | 91.3 |
| CHEMBL1229 | 312.4045 | 1.0457 | 4 | 1 | 8 | 90.65 |
| CHEMBL1064 | 418.5662 | 4.635 | 5 | 1 | 7 | 72.83 |
| CHEMBL354541 | 693.7204 | 7.7908 | 2 | 2 | 12 | 61.44 |
| CHEMBL564829 | 460.5746 | 3.492 | 4 | 2 | 4 | 91.21 |
| CHEMBL3930624 | 472.3951 | 2.6443 | 5 | 1 | 4 | 96.77 |
| CHEMBL3601411 | 588.7386 | 4.2378 | 8 | 2 | 4 | 156.7 |
| CHEMBL434090 | 546.6524 | 6.2017 | 8 | 1 | 13 | 99.16 |
| CHEMBL188255 | 654.7935 | 4.2747 | 8 | 5 | 19 | 172.16 |
| CHEMBL2104947 | 448.5341 | 5.3087 | 5 | 0 | 9 | 90.82 |
| CHEMBL1084866 | 429.8982 | 4.5005 | 3 | 2 | 6 | 70.56 |
| CHEMBL213934 | 344.5126 | 3.7268 | 3 | 3 | 10 | 106.23 |
| CHEMBL2105796 | 228.6324 | 0.5437 | 3 | 3 | 3 | 78.43 |
| CHEMBL189141 | 936.0168 | -2.745 | 9 | 8 | 31 | 404.94 |
| CHEMBL186052 | 696.8732 | 5.4389 | 8 | 5 | 21 | 172.16 |
| CHEMBL73151 | 340.4625 | 2.8532 | 1 | 2 | 3 | 51.37 |
| CHEMBL24955 | 249.7359 | 2.8668 | 1 | 0 | 0 | 20.31 |
| CHEMBL265019 | 1233.372 | 1.6557 | 13 | 8 | 32 | 417.83 |
| CHEMBL4780754 | 551.6404 | 6.2836 | 4 | 2 | 5 | 95.49 |
| CHEMBL409038 | 322.7484 | 3.9926 | 4 | 1 | 2 | 77.83 |
| CHEMBL3644452 | 354.3932 | 1.7975 | 2 | 1 | 2 | 83.24 |
| CHEMBL3644458 | 414.4931 | 1.3727 | 4 | 0 | 3 | 114.9 |
| CHEMBL3655689 | 607.7237 | 1.7717 | 7 | 3 | 12 | 188.72 |
| CHEMBL3655693 | 622.735 | 1.5279 | 6 | 2 | 13 | 185.54 |
| CHEMBL2370886 | 1668.902 | -3.6081 | 17 | 16 | 58 | 702.83 |
| CHEMBL3039507 | 424.9382 | 3.7341 | 6 | 3 | 6 | 104.45 |
| CHEMBL4594275 | 460.5315 | 2.4335 | 6 | 3 | 6 | 134.06 |
| CHEMBL1084617 | 436.5035 | 2.5345 | 5 | 1 | 8 | 77.85 |
| CHEMBL468894 | 534.673 | 1.55 | 5 | 2 | 10 | 163.64 |
| CHEMBL4297378 | 276.2465 | -1.1072 | 3 | 1 | 3 | 141.45 |
| CHEMBL488025 | 372.41 | 3.7099 | 2 | 0 | 4 | 79.82 |
| CHEMBL1744447 | 480.5297 | 1.7239 | 6 | 2 | 10 | 152.13 |
| CHEMBL572284 | 402.4409 | 1.5054 | 6 | 2 | 9 | 112.27 |
| CHEMBL1144 | 424.5277 | 2.1316 | 5 | 3 | 11 | 124.29 |
| CHEMBL3301627 | 1145.049 | 6.2314 | 10 | 6 | 29 | 234.76 |
| CHEMBL402548 | 474.5548 | 2.0066 | 4 | 2 | 6 | 93.8 |
| CHEMBL2059028 | 749.2936 | 7.0836 | 8 | 0 | 18 | 111.16 |
| CHEMBL204021 | 666.7711 | 8.3044 | 3 | 0 | 14 | 83.74 |
| CHEMBL4650214 | 576.6832 | 6.4587 | 5 | 1 | 8 | 84.23 |
| CHEMBL2103847 | 406.4727 | 2.3814 | 6 | 4 | 10 | 124.96 |
| CHEMBL458875 | 260.219 | 0.1708 | 5 | 3 | 2 | 104.55 |
| CHEMBL4802151 | 1281.848 | 11.3804 | 10 | 2 | 25 | 285.21 |
| CHEMBL2216859 | 440.5401 | 3.0488 | 3 | 2 | 5 | 76.98 |
| CHEMBL125 | 407.568 | 4.8664 | 3 | 0 | 20 | 68.4 |
| CHEMBL4297063 | 392.4294 | 2.3986 | 2 | 3 | 2 | 94.04 |
| CHEMBL1206690 | 370.4222 | 3.2118 | 5 | 1 | 5 | 97.65 |
| CHEMBL4297624 | 351.4157 | 1.1614 | 4 | 0 | 6 | 82.43 |
| CHEMBL4297529 | 637.6638 | -5.7871 | 8 | 6 | 18 | 344.91 |
| CHEMBL515606 | 417.4986 | 2.3665 | 4 | 0 | 9 | 99.18 |
| CHEMBL1241348 | 869.8209 | 5.9759 | 9 | 3 | 15 | 226.62 |
| CHEMBL1229517 | 489.9221 | 4.3433 | 4 | 2 | 7 | 100.3 |
| CHEMBL601719 | 450.3367 | 3.2261 | 3 | 1 | 5 | 77.99 |
| CHEMBL3989406 | 348.3936 | 1.539 | 1 | 0 | 8 | 106.94 |
| CHEMBL325424 | 425.5189 | 6.5806 | 1 | 0 | 6 | 59.3 |
| CHEMBL3905910 | 469.8382 | 4.7318 | 5 | 3 | 4 | 94.14 |
| CHEMBL3545363 | 838.8653 | 3.9432 | 11 | 3 | 7 | 203.6 |
| CHEMBL1200534 | 498.5681 | 3.3032 | 5 | 0 | 12 | 114.4 |
| CHEMBL1213165 | 418.3708 | 1.0258 | 4 | 1 | 3 | 103.7 |
| CHEMBL4471306 | 580.8249 | 0.6495 | 10 | 3 | 9 | 216.11 |
| CHEMBL188944 | 435.557 | 2.8069 | 5 | 4 | 12 | 130.75 |
| CHEMBL3544911 | 365.3892 | 1.5908 | 6 | 2 | 8 | 134.76 |
| CHEMBL2051970 | 329.4549 | 2.9602 | 3 | 0 | 8 | 99.98 |
| CHEMBL434492 | 399.4785 | 1.7371 | 3 | 1 | 11 | 122.16 |
| CHEMBL280728 | 366.3344 | 3.614 | 3 | 0 | 9 | 71.78 |
| CHEMBL1983865 | 323.4072 | 3.8651 | 5 | 1 | 8 | 89.93 |
| CHEMBL3644451 | 336.4027 | 1.592 | 2 | 1 | 2 | 83.24 |
| CHEMBL1201387 | 402.484 | 2.8441 | 1 | 0 | 8 | 106.94 |
| CHEMBL259388 | 459.5782 | 5.4208 | 2 | 0 | 5 | 66.37 |
| CHEMBL460378 | 563.7109 | 2.6842 | 5 | 2 | 11 | 159.98 |
| CHEMBL1697842 | 286.3655 | 4.4048 | 1 | 0 | 5 | 46.53 |
| CHEMBL3644456 | 432.4836 | 1.5782 | 4 | 0 | 3 | 114.9 |
| CHEMBL277689 | 484.995 | 4.6576 | 3 | 1 | 11 | 104.74 |
| CHEMBL132586 | 482.572 | 3.4788 | 6 | 3 | 14 | 126.49 |
| CHEMBL466585 | 550.7121 | 2.7647 | 4 | 1 | 11 | 136.13 |
| CHEMBL3652780 | 294.308 | 2.0674 | 2 | 0 | 3 | 83.84 |
| CHEMBL94567 | 412.1865 | -2.0282 | 13 | 2 | 6 | 226.2 |
| CHEMBL365195 | 764.0702 | 5.7261 | 8 | 6 | 24 | 200.26 |
| CHEMBL184041 | 559.5133 | 8.8605 | 1 | 0 | 6 | 74.77 |
| CHEMBL4536936 | 263.3122 | 2.7984 | 4 | 1 | 4 | 80.7 |
| CHEMBL3143754 | 619.7925 | 2.2885 | 7 | 5 | 19 | 188.95 |
| CHEMBL206468 | 147.1723 | -1.9016 | 3 | 3 | 1 | 72.72 |
| CHEMBL523865 | 573.6644 | 0.2127 | 7 | 3 | 12 | 209.39 |
| CHEMBL185745 | 526.6245 | 2.5406 | 6 | 5 | 15 | 159.85 |
| CHEMBL363028 | 682.8467 | 5.1871 | 8 | 5 | 21 | 172.16 |
| CHEMBL3661401 | 226.2705 | 2.5828 | 2 | 1 | 3 | 32.76 |
| CHEMBL3661414 | 150.1745 | 0.9706 | 2 | 1 | 2 | 32.76 |
| CHEMBL269455 | 286.233 | 2.399 | 2 | 2 | 0 | 58.2 |
| CHEMBL3545187 | 442.3179 | 2.8482 | 4 | 2 | 7 | 84.73 |
| CHEMBL3143748 | 725.8714 | 3.8187 | 9 | 6 | 21 | 201.26 |
| CHEMBL3422109 | 387.3947 | 2.5028 | 6 | 2 | 8 | 135.65 |
| CHEMBL1200831 | 466.614 | 2.9764 | 5 | 0 | 10 | 146.54 |
| CHEMBL264004 | 739.9626 | 3.4837 | 9 | 6 | 21 | 217.48 |
| CHEMBL186528 | 463.6101 | 3.7193 | 5 | 4 | 14 | 130.75 |
| CHEMBL70620 | 371.4749 | 0.0319 | 3 | 3 | 9 | 185.62 |
| CHEMBL3644460 | 361.4122 | 1.8994 | 3 | 1 | 3 | 107.03 |
| CHEMBL154580 | 182.2179 | 3.0692 | 1 | 0 | 2 | 17.07 |
| CHEMBL2170440 | 331.3281 | 3.6976 | 3 | 2 | 6 | 110.93 |
| CHEMBL2368199 | 431.4986 | 5.5192 | 4 | 1 | 5 | 59.42 |
| CHEMBL359790 | 421.5304 | 2.3444 | 5 | 4 | 12 | 130.75 |
| CHEMBL73193 | 526.4921 | 3.6883 | 6 | 2 | 9 | 157.59 |
| CHEMBL187568 | 563.6862 | 1.6916 | 7 | 6 | 16 | 188.95 |
| CHEMBL269499 | 1191.292 | 0.3565 | 13 | 8 | 30 | 417.83 |
| CHEMBL4650323 | 455.5084 | 4.8038 | 4 | 1 | 5 | 95.38 |
| CHEMBL4297277 | 319.3986 | 3.4176 | 2 | 0 | 4 | 115 |
| CHEMBL2403318 | 653.5682 | 6.4972 | 4 | 1 | 7 | 114.93 |
| CHEMBL2141296 | 361.0287 | 2.9739 | 4 | 4 | 7 | 98.66 |
| CHEMBL73622 | 280.1389 | 1.0169 | 2 | 0 | 1 | 82.91 |
| CHEMBL7679 | 391.1912 | 3.6282 | 2 | 0 | 4 | 72.19 |
| CHEMBL457725 | 386.4894 | 5.1947 | 2 | 2 | 4 | 87.05 |
| CHEMBL1256351 | 199.6342 | 1.513 | 0 | 0 | 3 | 63.32 |
| CHEMBL188916 | 582.7308 | 4.1673 | 6 | 5 | 17 | 159.85 |
| CHEMBL49004 | 577.7128 | 0.9136 | 7 | 5 | 17 | 188.95 |
| CHEMBL1113 | 313.7814 | 3.4216 | 2 | 0 | 1 | 36.86 |
| CHEMBL2035187 | 472.5787 | 4.547 | 5 | 1 | 4 | 68.74 |
| CHEMBL3987016 | 459.4658 | 3.3976 | 4 | 2 | 6 | 101.57 |
| CHEMBL4297463 | 518.5711 | 4.0623 | 3 | 2 | 8 | 79.36 |
| CHEMBL4583691 | 502.4856 | 4.0666 | 6 | 2 | 8 | 96.81 |
| CHEMBL4297389 | 421.4359 | 4.6207 | 3 | 2 | 6 | 60.77 |
| CHEMBL3989939 | 428.4352 | 2.8897 | 4 | 2 | 3 | 86.52 |
| CHEMBL3989870 | 463.552 | 3.655 | 6 | 1 | 7 | 132.38 |
| CHEMBL3039596 | 562.6546 | 5.5665 | 5 | 0 | 15 | 122.85 |
| CHEMBL1201087 | 451.6043 | 2.715 | 2 | 2 | 8 | 71.68 |
| CHEMBL4594405 | 488.0189 | 4.5124 | 4 | 2 | 5 | 83.66 |
| CHEMBL115 | 613.7895 | 3.0824 | 5 | 4 | 12 | 118.03 |
| CHEMBL293743 | 314.311 | 3.9613 | 3 | 2 | 4 | 75.79 |
| CHEMBL2103775 | 406.4958 | 1.7081 | 4 | 1 | 9 | 129.08 |
| CHEMBL3982723 | 472.4662 | 1.6058 | 5 | 2 | 5 | 135.94 |
| CHEMBL4116008 | 445.5385 | 1.452 | 5 | 3 | 8 | 141.93 |
| CHEMBL1232461 | 423.8954 | 3.3711 | 5 | 1 | 5 | 81.4 |
| CHEMBL184 | 225.2046 | -0.8071 | 6 | 3 | 4 | 119.31 |
| CHEMBL2105759 | 371.4169 | -0.7201 | 6 | 1 | 5 | 128.94 |
| CHEMBL1967878 | 451.5397 | 3.1018 | 3 | 3 | 6 | 99.41 |
| CHEMBL1237119 | 390.5131 | 4.41 | 3 | 2 | 10 | 86.99 |
| CHEMBL539378 | 584.7067 | 6.7554 | 5 | 0 | 8 | 69.48 |
| CHEMBL308954 | 435.2767 | 5.4982 | 5 | 2 | 4 | 120.64 |
| CHEMBL1201011 | 438.5161 | 3.336 | 3 | 0 | 10 | 95.94 |
| CHEMBL2142592 | 512.502 | 4.5616 | 4 | 0 | 5 | 67.15 |
| CHEMBL2107819 | 508.995 | 1.6989 | 6 | 3 | 8 | 140.06 |
| CHEMBL322241 | 604.7397 | 3.5778 | 6 | 5 | 14 | 166.75 |
| CHEMBL2104406 | 603.7052 | 5.202 | 9 | 0 | 16 | 117.15 |
| CHEMBL410414 | 674.7102 | 7.552 | 3 | 2 | 10 | 83.44 |
| CHEMBL4297641 | 450.939 | 2.1352 | 5 | 2 | 5 | 110.69 |
| CHEMBL187249 | 540.6511 | 3.0031 | 6 | 5 | 15 | 159.85 |
| CHEMBL2110732 | 469.939 | 4.9518 | 4 | 2 | 7 | 79.38 |
| CHEMBL1976040 | 500.5954 | 3.7565 | 5 | 2 | 7 | 104.34 |
| CHEMBL4117187 | 478.4026 | 4.0162 | 3 | 0 | 5 | 63.16 |
| CHEMBL2103749 | 2180.285 | -7.792 | 22 | 19 | 67 | 901.57 |
| CHEMBL4297203 | 241.0951 | -1.0099 | 3 | 2 | 4 | 84.83 |
| CHEMBL1200944 | 232.2337 | -1.3736 | 3 | 0 | 1 | 102.96 |
| CHEMBL175691 | 366.4185 | 5.3056 | 4 | 2 | 6 | 97.42 |
| CHEMBL4297435 | 464.3616 | 2.5771 | 4 | 0 | 7 | 90.45 |
| CHEMBL3948730 | 571.5492 | 6.6329 | 6 | 1 | 6 | 109.06 |
| CHEMBL3360203 | 541.0218 | 4.0183 | 6 | 3 | 8 | 156.71 |
| CHEMBL3833385 | 929.0967 | 6.7821 | 10 | 4 | 14 | 229.93 |
| CHEMBL3904602 | 474.6012 | 3.9056 | 4 | 2 | 4 | 91.21 |
| CHEMBL3121849 | 762.8964 | 5.4975 | 8 | 4 | 12 | 174.64 |
| CHEMBL39541 | 785.0909 | 4.6365 | 7 | 2 | 21 | 161.65 |
| CHEMBL224060 | 324.7676 | 2.7372 | 4 | 0 | 3 | 61.42 |
| CHEMBL289228 | 489.3958 | 5.5516 | 2 | 0 | 4 | 65.84 |
| CHEMBL1166 | 508.6341 | 1.241 | 4 | 3 | 10 | 186.09 |
| CHEMBL2001019 | 372.3833 | 0.8602 | 7 | 1 | 5 | 107.43 |
| CHEMBL148674 | 380.3939 | 2.9312 | 3 | 1 | 8 | 111.62 |
| CHEMBL223228 | 315.675 | 4.8543 | 2 | 1 | 2 | 38.33 |
| CHEMBL85943 | 247.8376 | -1.6348 | 0 | 0 | 0 | 80.26 |
| CHEMBL2103743 | 312.3696 | 0.9045 | 4 | 1 | 3 | 88.91 |
| CHEMBL545315 | 485.9384 | 3.9337 | 5 | 2 | 9 | 88.61 |
| CHEMBL36503 | 440.4703 | 2.1576 | 4 | 0 | 13 | 139.97 |
| CHEMBL217899 | 339.4578 | 4.01 | 3 | 0 | 3 | 76.77 |
| CHEMBL4297588 | 740.929 | 6.3309 | 6 | 4 | 17 | 208.05 |
| CHEMBL188956 | 591.7394 | 2.5346 | 7 | 6 | 17 | 188.95 |
| CHEMBL99701 | 377.3917 | 1.407 | 2 | 0 | 8 | 127.25 |
| CHEMBL3655688 | 617.8013 | 4.3712 | 5 | 3 | 13 | 153.83 |
| CHEMBL3037892 | 1743.881 | -6.6172 | 20 | 13 | 50 | 776.47 |
| CHEMBL265127 | 1283.387 | 1.645 | 14 | 9 | 32 | 438.06 |
| CHEMBL2106994 | 477.6383 | 4.439 | 4 | 0 | 7 | 54.37 |
| CHEMBL2105735 | 748.2858 | 3.7625 | 10 | 3 | 14 | 190.71 |
| CHEMBL129732 | 597.6609 | 3.3007 | 7 | 4 | 16 | 151.51 |
| CHEMBL266672 | 1219.345 | 1.1995 | 13 | 8 | 31 | 417.83 |
| CHEMBL2103882 | 369.4159 | 2.7776 | 2 | 2 | 2 | 66.89 |
| CHEMBL2105801 | 259.2988 | -0.4922 | 5 | 3 | 4 | 90.23 |
| CHEMBL2220442 | 411.4659 | 4.0219 | 2 | 2 | 8 | 82.69 |
| CHEMBL1770916 | 489.6441 | 4.8634 | 2 | 1 | 11 | 70.98 |
| CHEMBL4594445 | 410.4101 | 4.0011 | 3 | 0 | 6 | 68.71 |
| CHEMBL4297477 | 389.4024 | 1.1404 | 4 | 1 | 4 | 79.18 |
| CHEMBL2103868 | 501.5057 | 4.3428 | 6 | 2 | 8 | 98.78 |
| CHEMBL1689063 | 265.2437 | -0.7012 | 4 | 1 | 3 | 138.62 |
| CHEMBL463981 | 391.7381 | 2.7453 | 3 | 2 | 7 | 103.02 |
| CHEMBL590799 | 398.4125 | 1.3829 | 6 | 1 | 10 | 134.81 |
| CHEMBL3545368 | 349.4262 | 2.6833 | 2 | 3 | 7 | 77.15 |
| CHEMBL3989970 | 415.4248 | 1.3718 | 6 | 2 | 6 | 115.02 |
| CHEMBL2387080 | 460.5315 | 1.4064 | 6 | 1 | 5 | 118.67 |
| CHEMBL1200734 | 583.5909 | 1.3324 | 7 | 2 | 14 | 201.57 |
| CHEMBL1879463 | 469.5365 | 6.8042 | 4 | 0 | 4 | 76.5 |
| CHEMBL3989866 | 449.3799 | 2.05 | 5 | 2 | 3 | 100.87 |
| CHEMBL4297182 | 536.5348 | 3.5886 | 6 | 1 | 3 | 104.03 |
| CHEMBL1174 | 831.9619 | -2.4999 | 8 | 8 | 11 | 374.49 |
| CHEMBL65067 | 481.5854 | 4.0386 | 4 | 1 | 8 | 65.9 |
| CHEMBL2107825 | 476.4659 | 1.39 | 8 | 2 | 12 | 153.29 |
| CHEMBL478858 | 416.5967 | 6.6261 | 1 | 1 | 11 | 69.64 |
| CHEMBL1947204 | 448.8767 | 5.6284 | 4 | 2 | 7 | 76.14 |
| CHEMBL218490 | 324.44 | 0.5845 | 4 | 1 | 3 | 151.33 |
| CHEMBL833 | 263.7857 | 3.6657 | 0 | 0 | 2 | 31.48 |
| CHEMBL4297661 | 601.8629 | 2.7759 | 5 | 2 | 9 | 140.76 |
| CHEMBL1200340 | 312.4045 | 1.0457 | 4 | 1 | 8 | 90.65 |
| CHEMBL4298172 | 373.4476 | 2.0833 | 4 | 1 | 4 | 66.32 |
| CHEMBL398435 | 522.568 | 2.7062 | 8 | 4 | 10 | 163.74 |
| CHEMBL4650366 | 383.4607 | 3.627 | 4 | 0 | 5 | 73.75 |
| CHEMBL4650314 | 332.308 | -0.7277 | 4 | 2 | 6 | 117.86 |
| CHEMBL1508 | 324.3919 | 3.7214 | 2 | 0 | 6 | 36.26 |
| CHEMBL503 | 404.5396 | 4.2181 | 5 | 1 | 7 | 72.83 |
| CHEMBL4297583 | 387.38 | 2.5582 | 4 | 1 | 3 | 67.45 |
| CHEMBL110094 | 291.4299 | 4.7738 | 0 | 0 | 3 | 3.24 |
| CHEMBL4297520 | 516.3021 | 3.9225 | 6 | 2 | 6 | 118.91 |
| CHEMBL506110 | 594.6571 | -0.236 | 10 | 1 | 9 | 216.16 |
| CHEMBL2338801 | 284.2338 | 3.0216 | 3 | 2 | 4 | 65.98 |
| CHEMBL3545432 | 517.1216 | 2.9544 | 5 | 2 | 11 | 168.33 |
| CHEMBL3907479 | 356.3806 | 2.4343 | 3 | 3 | 3 | 105.38 |
| CHEMBL1277072 | 468.5206 | 1.5805 | 4 | 3 | 5 | 97.9 |
| CHEMBL1173655 | 485.9384 | 3.8134 | 5 | 2 | 8 | 88.61 |
| CHEMBL3622821 | 380.3676 | 2.2733 | 3 | 2 | 4 | 78.84 |
| CHEMBL274654 | 310.3471 | 2.4853 | 1 | 2 | 4 | 82.19 |
| CHEMBL319111 | 391.4644 | 4.9216 | 3 | 0 | 5 | 46.09 |
| CHEMBL343448 | 540.6958 | 1.6898 | 6 | 4 | 2 | 193.3 |
| CHEMBL4650328 | 281.2727 | 1.1542 | 6 | 3 | 2 | 136.72 |
| CHEMBL2338329 | 352.3407 | 2.2559 | 4 | 2 | 5 | 108.75 |
| CHEMBL98 | 264.3202 | 1.9802 | 3 | 3 | 8 | 78.43 |
| CHEMBL1852688 | 560.4754 | 6.4405 | 5 | 2 | 8 | 95.09 |
| CHEMBL922 | 501.4705 | 1.9022 | 11 | 1 | 15 | 176.79 |
| CHEMBL2103852 | 417.2566 | 2.0844 | 4 | 3 | 3 | 129.54 |
| CHEMBL218650 | 534.4844 | 6.2732 | 5 | 0 | 9 | 101.12 |
| CHEMBL1161632 | 62.0248 | 0.1623 | 0 | 0 | 0 | 57.53 |
| CHEMBL3301599 | 409.5212 | 3.2713 | 3 | 2 | 4 | 67.25 |
| CHEMBL449158 | 905.0326 | 3.9331 | 17 | 4 | 13 | 240.11 |
| CHEMBL1957287 | 517.401 | 6.4983 | 4 | 0 | 6 | 56.49 |
| CHEMBL3989690 | 328.2766 | 4.225 | 1 | 0 | 4 | 12.47 |
| CHEMBL1580 | 268.2691 | -1.5346 | 6 | 3 | 2 | 112.13 |
| CHEMBL3833368 | 323.3641 | 2.5806 | 1 | 2 | 3 | 56.92 |
| CHEMBL3137320 | 380.3509 | 2.0488 | 4 | 2 | 2 | 88.49 |
| CHEMBL189 | 211.2194 | 0.9055 | 3 | 1 | 1 | 69.54 |
| CHEMBL4802154 | 593.1211 | 5.7514 | 5 | 2 | 8 | 113.45 |
| CHEMBL4650321 | 427.495 | 3.9169 | 4 | 1 | 6 | 85.52 |
| CHEMBL2107821 | 263.3752 | 2.66 | 2 | 1 | 6 | 55.48 |
| CHEMBL3655081 | 323.4138 | 0.494 | 4 | 2 | 6 | 99.36 |
| CHEMBL258734 | 731.8312 | 3.6413 | 9 | 3 | 8 | 188.9 |
| CHEMBL377300 | 370.3776 | 3.1057 | 5 | 2 | 5 | 85.19 |
| CHEMBL1352 | 63.0128 | 0.0727 | 1 | 1 | 0 | 63.37 |
| CHEMBL3182314 | 433.4549 | 1.6181 | 5 | 2 | 9 | 150.08 |
| CHEMBL1097279 | 772.3274 | -1.0307 | 11 | 3 | 16 | 347.26 |
| CHEMBL231813 | 679.8493 | 2.5452 | 8 | 4 | 14 | 179.56 |
| CHEMBL4594436 | 413.4039 | 2.6705 | 3 | 1 | 6 | 147.88 |
| CHEMBL296913 | 369.4143 | 3.8267 | 5 | 0 | 5 | 85.62 |
| CHEMBL265502 | 1297.28 | 5.2401 | 11 | 6 | 16 | 534.03 |
| CHEMBL4521078 | 438.4779 | 3.9902 | 4 | 0 | 5 | 94.04 |
| CHEMBL3586404 | 397.4708 | 1.6661 | 6 | 2 | 4 | 100.47 |
| CHEMBL17879 | 674.9553 | 6.1469 | 4 | 1 | 13 | 78.46 |
| CHEMBL2387408 | 546.6755 | 3.8254 | 3 | 0 | 13 | 150.41 |
| CHEMBL435224 | 645.1396 | 3.6153 | 7 | 0 | 12 | 131.91 |
| CHEMBL346977 | 391.5507 | 3.5076 | 2 | 0 | 5 | 77.54 |
| CHEMBL538867 | 504.768 | 4.3566 | 3 | 1 | 2 | 75.81 |
| CHEMBL3707350 | 562.7063 | 2.6241 | 5 | 2 | 9 | 120.16 |
| CHEMBL227875 | 359.2574 | 4.1221 | 4 | 0 | 6 | 88.65 |
| CHEMBL525610 | 4117.715 | -16.9682 | 48 | 47 | 146 | 1803.25 |
| CHEMBL589390 | 319.4433 | 3.9152 | 1 | 0 | 3 | 21.06 |
| CHEMBL1092581 | 446.4918 | 2.6668 | 5 | 2 | 7 | 108.23 |
| CHEMBL3544977 | 1001.264 | 11.5678 | 8 | 4 | 12 | 174.64 |
| CHEMBL490672 | 503.6358 | 4.7389 | 7 | 1 | 8 | 103.02 |
| CHEMBL4297668 | 460.5746 | 3.492 | 4 | 2 | 4 | 91.21 |
| CHEMBL35033 | 302.3682 | 3.7654 | 3 | 1 | 6 | 64.09 |
| CHEMBL3544985 | 492.5667 | 4.7914 | 6 | 1 | 6 | 115.74 |
| CHEMBL3286580 | 398.3697 | 4.2439 | 1 | 1 | 9 | 37.05 |
| CHEMBL4129286 | 545.4519 | 1.6689 | 10 | 4 | 11 | 188.22 |
| CHEMBL103667 | 527.6572 | 6.7989 | 4 | 2 | 8 | 80.65 |
| CHEMBL729 | 628.8008 | 5.6455 | 5 | 4 | 15 | 120 |
| CHEMBL2103839 | 990.2061 | 6.5497 | 14 | 2 | 8 | 211.31 |
| CHEMBL270515 | 413.2751 | 2.8434 | 6 | 5 | 9 | 131.78 |
| CHEMBL3935857 | 363.37 | 1.0919 | 5 | 1 | 2 | 99.41 |
| CHEMBL2087361 | 391.4198 | 4.1312 | 6 | 2 | 3 | 74.73 |
| CHEMBL4594260 | 572.7376 | 4.0547 | 4 | 2 | 9 | 86.9 |
| CHEMBL476960 | 267.2762 | -4.0822 | 7 | 7 | 5 | 153.64 |
| CHEMBL160 | 1202.611 | 4.3339 | 12 | 5 | 15 | 278.8 |
| CHEMBL719 | 500.6222 | 2.6074 | 7 | 3 | 17 | 146.05 |
| CHEMBL2104306 | 324.3737 | 3.0639 | 4 | 2 | 7 | 69.73 |
| CHEMBL493982 | 492.5817 | 5.3493 | 5 | 1 | 6 | 77.52 |
| CHEMBL572878 | 464.5864 | 4.1199 | 4 | 3 | 7 | 127.37 |
| CHEMBL3977543 | 478.9291 | 4.8998 | 4 | 3 | 4 | 134.06 |
| CHEMBL1214827 | 409.5212 | 3.2713 | 3 | 2 | 4 | 67.25 |
| CHEMBL3989843 | 133.1903 | 1.1936 | 0 | 0 | 1 | 26.02 |
| CHEMBL3545155 | 466.3305 | 4.6997 | 3 | 3 | 5 | 94.2 |
| CHEMBL371405 | 313.7766 | 1.2968 | 4 | 2 | 4 | 75.63 |
| CHEMBL444172 | 527.6042 | 4.6991 | 3 | 1 | 6 | 48.83 |
| CHEMBL522892 | 392.4294 | 2.3986 | 2 | 3 | 2 | 94.04 |
| CHEMBL122 | 314.3557 | 2.8715 | 4 | 0 | 3 | 68.82 |
| CHEMBL2107830 | 450.9093 | 1.8399 | 7 | 4 | 6 | 108.61 |
| CHEMBL2408045 | 473.9277 | 3.4878 | 5 | 2 | 7 | 88.61 |
| CHEMBL3137309 | 868.4392 | 7.3306 | 6 | 3 | 13 | 180.41 |
| CHEMBL279785 | 331.4079 | 0.214 | 5 | 5 | 8 | 127.76 |
| CHEMBL12856 | 187.198 | -0.0026 | 2 | 2 | 1 | 71.77 |
| CHEMBL3545312 | 762.8964 | 5.4975 | 8 | 4 | 12 | 174.64 |
| CHEMBL3828074 | 588.6988 | 4.4691 | 5 | 1 | 7 | 133 |
| CHEMBL480 | 369.3615 | 3.4266 | 3 | 1 | 6 | 99.16 |
| CHEMBL1963681 | 483.9506 | 1.7747 | 6 | 3 | 8 | 128.62 |
| CHEMBL1442422 | 295.3788 | 2.8853 | 1 | 0 | 3 | 26.79 |
| CHEMBL182 | 255.2306 | -1.3178 | 7 | 4 | 5 | 139.54 |
| CHEMBL2064032 | 345.3912 | 2.4559 | 3 | 0 | 5 | 72.37 |
| CHEMBL228814 | 517.0616 | 5.7596 | 3 | 0 | 9 | 81.22 |
| CHEMBL1091644 | 421.4937 | 3.9572 | 4 | 2 | 3 | 89.85 |
| CHEMBL3407547 | 569.626 | 4.0903 | 7 | 0 | 9 | 163.26 |
| CHEMBL932 | 504.6256 | 2.4892 | 8 | 4 | 12 | 145.44 |
| CHEMBL2107762 | 283.3433 | 4.3203 | 1 | 0 | 4 | 21.06 |
| CHEMBL1519 | 430.5372 | 3.4439 | 3 | 0 | 10 | 95.94 |
| CHEMBL3039515 | 695.8884 | 6.402 | 5 | 2 | 16 | 175.79 |
| CHEMBL2218894 | 410.458 | 3.3441 | 2 | 2 | 8 | 85.52 |
| CHEMBL1230609 | 632.6538 | 4.4371 | 7 | 2 | 12 | 111.25 |
| CHEMBL760 | 256.0881 | 1.9651 | 2 | 0 | 0 | 44.7 |
| CHEMBL3126842 | 659.838 | 4.5489 | 5 | 1 | 6 | 112.57 |
| CHEMBL2105762 | 398.4275 | 0.628 | 4 | 0 | 3 | 98.83 |
| CHEMBL459505 | 377.5058 | 4.9583 | 3 | 1 | 7 | 83.87 |
| CHEMBL415 | 314.8523 | 5.0523 | 0 | 0 | 4 | 6.48 |
| CHEMBL3707219 | 323.341 | 2.4829 | 6 | 2 | 11 | 101.93 |
| CHEMBL288441 | 530.4462 | 4.8833 | 5 | 1 | 9 | 82.88 |
| CHEMBL4650327 | 425.4048 | 3.1459 | 3 | 0 | 5 | 141.56 |
| CHEMBL1735 | 613.7895 | 3.0824 | 5 | 4 | 12 | 118.03 |
| CHEMBL3545065 | 327.4008 | 1.4274 | 2 | 2 | 4 | 93.44 |
| CHEMBL2105684 | 457.6814 | 3.1892 | 4 | 3 | 6 | 88.41 |
| CHEMBL152 | 279.187 | -0.9867 | 6 | 2 | 6 | 157.71 |
| CHEMBL3187723 | 441.2268 | 2.7303 | 4 | 3 | 6 | 88.41 |
| CHEMBL2107817 | 264.2358 | -1.379 | 3 | 1 | 3 | 141.45 |
| CHEMBL2105729 | 519.5624 | 5.3325 | 5 | 2 | 6 | 147.48 |
| CHEMBL2158051 | 561.7149 | 2.2641 | 4 | 2 | 9 | 110.85 |
| CHEMBL110 | 260.2487 | 0.8202 | 2 | 1 | 5 | 90.06 |
| CHEMBL4594400 | 459.4838 | 0.6505 | 7 | 0 | 4 | 126.69 |
| CHEMBL644 | 294.4338 | 4.8366 | 0 | 0 | 4 | 6.48 |
| CHEMBL571987 | 310.3504 | 2.6869 | 5 | 1 | 6 | 94.86 |
| CHEMBL3707409 | 553.5141 | 1.3412 | 6 | 1 | 6 | 119.62 |
| CHEMBL4303323 | 401.3964 | 3.1756 | 5 | 2 | 4 | 101.38 |
| CHEMBL1323 | 547.6636 | 2.8264 | 7 | 3 | 12 | 148.8 |
| CHEMBL2105709 | 560.6672 | 6.349 | 6 | 2 | 8 | 134.92 |
| CHEMBL2105261 | 310.4299 | 4.2842 | 2 | 1 | 1 | 34.14 |
| CHEMBL2079130 | 345.3912 | 2.4559 | 3 | 0 | 5 | 72.37 |
| CHEMBL126224 | 284.439 | 4.7357 | 0 | 0 | 4 | 8.17 |
| CHEMBL4216467 | 510.3582 | 5.866 | 3 | 3 | 5 | 88.05 |
| CHEMBL553 | 393.4357 | 4.3126 | 6 | 2 | 10 | 74.73 |
| CHEMBL1289926 | 386.4695 | 3.946 | 3 | 2 | 5 | 95.97 |
| CHEMBL4297258 | 568.5933 | 5.2774 | 5 | 2 | 12 | 100.44 |
| CHEMBL85164 | 398.4125 | 1.3829 | 6 | 1 | 10 | 134.81 |
| CHEMBL4209157 | 478.4227 | 3.9612 | 5 | 2 | 4 | 89.13 |
| CHEMBL25 | 180.1574 | 1.2021 | 2 | 0 | 3 | 63.6 |
| CHEMBL3545062 | 883.0019 | 5.5115 | 10 | 4 | 13 | 193.1 |
| CHEMBL49642 | 389.8343 | 3.8697 | 3 | 1 | 5 | 63.99 |
| CHEMBL225071 | 458.4876 | 1.8792 | 3 | 2 | 9 | 180.93 |
| CHEMBL799 | 369.4607 | 3.8675 | 5 | 1 | 7 | 81.93 |
| CHEMBL2105737 | 485.4981 | 6.7353 | 4 | 1 | 6 | 63.69 |
| CHEMBL518520 | 443.4084 | 0.2399 | 7 | 2 | 6 | 155.07 |
| CHEMBL305666 | 518.561 | 2.8292 | 7 | 1 | 3 | 106.36 |
| CHEMBL2105733 | 323.3641 | 2.5806 | 1 | 2 | 3 | 56.92 |
| CHEMBL1173055 | 323.3641 | 2.5806 | 1 | 2 | 3 | 56.92 |
| CHEMBL25263 | 340.3716 | 0.2266 | 2 | 0 | 7 | 115.22 |
| CHEMBL1922660 | 637.8307 | 8.5002 | 4 | 0 | 13 | 111.77 |
| CHEMBL3137312 | 493.5747 | 4.6878 | 5 | 2 | 6 | 118.64 |
| CHEMBL4594298 | 688.7284 | 6.5709 | 10 | 1 | 11 | 143.07 |
| CHEMBL1339 | 488.603 | 2.7222 | 7 | 1 | 8 | 122.06 |
| CHEMBL450895 | 926.0949 | 0.2812 | 15 | 10 | 3 | 319.61 |
| CHEMBL225072 | 427.4106 | 0.6607 | 3 | 4 | 9 | 191.26 |
| CHEMBL2107358 | 317.3844 | 3.0135 | 2 | 2 | 3 | 53.17 |
| CHEMBL363387 | 376.3523 | 4.173 | 1 | 0 | 4 | 83.36 |
| CHEMBL4650338 | 489.4734 | 3.996 | 4 | 2 | 5 | 106.24 |
| CHEMBL521686 | 434.4628 | 2.3931 | 4 | 1 | 4 | 86.37 |
| CHEMBL1664 | 585.6068 | 2.688 | 9 | 2 | 14 | 195.91 |
| CHEMBL3039503 | 445.6147 | 5.9055 | 2 | 1 | 5 | 106.08 |
| CHEMBL180022 | 557.0427 | 4.7747 | 6 | 2 | 11 | 112.4 |
| CHEMBL4297598 | 410.9116 | 6.125 | 2 | 1 | 4 | 41.99 |
| CHEMBL1168 | 416.5106 | 2.9877 | 3 | 0 | 10 | 95.94 |
| CHEMBL713 | 277.2792 | -1.3654 | 5 | 4 | 2 | 130.05 |
| CHEMBL2110588 | 404.5429 | 3.8087 | 4 | 2 | 11 | 71.03 |
| CHEMBL3039504 | 539.6248 | 3.0932 | 4 | 2 | 8 | 94.22 |
| CHEMBL4065616 | 602.576 | 2.3641 | 11 | 4 | 13 | 213.88 |
| CHEMBL777 | 199.1608 | -1.4465 | 3 | 1 | 2 | 87.07 |
| CHEMBL2364624 | 455.4722 | 5.9926 | 2 | 1 | 7 | 75.11 |
| CHEMBL3121539 | 543.7146 | 5.1596 | 4 | 1 | 8 | 124.54 |
| CHEMBL1083390 | 353.4381 | 2.7025 | 3 | 0 | 3 | 70.68 |
| CHEMBL4594446 | 358.1966 | 3.3363 | 4 | 1 | 1 | 83.06 |
| CHEMBL3989869 | 571.5492 | 6.6329 | 6 | 1 | 6 | 109.06 |
| CHEMBL2103871 | 517.9156 | 5.2512 | 5 | 1 | 6 | 108.76 |
| CHEMBL217092 | 542.0265 | 3.817 | 7 | 1 | 8 | 90.44 |
| CHEMBL1200969 | 528.5297 | 5.5973 | 2 | 2 | 4 | 58.2 |
| CHEMBL3099695 | 227.237 | 1.9754 | 2 | 0 | 2 | 41.61 |
| CHEMBL3545185 | 443.306 | 1.6976 | 5 | 2 | 7 | 97.62 |
| CHEMBL3894860 | 446.548 | 3.1793 | 4 | 2 | 3 | 91.21 |
| CHEMBL2105708 | 532.5595 | 4.7935 | 3 | 1 | 6 | 66.29 |
| CHEMBL2048484 | 444.5157 | 3.3562 | 5 | 4 | 5 | 118.39 |
| CHEMBL1139 | 352.4651 | 2.6827 | 3 | 2 | 10 | 86.99 |
| CHEMBL1161681 | 47.0134 | 0.2812 | 1 | 1 | 0 | 49.66 |
| CHEMBL1789941 | 306.365 | 1.7999 | 4 | 1 | 4 | 83.18 |
| CHEMBL3545110 | 434.5373 | 2.7183 | 4 | 1 | 5 | 91.21 |
| CHEMBL2095208 | 776.0227 | 5.4517 | 7 | 3 | 20 | 194.5 |
| CHEMBL2013174 | 910.5174 | 6.07 | 9 | 3 | 18 | 222.02 |
| CHEMBL1206245 | 474.4187 | 2.2688 | 10 | 0 | 10 | 156.01 |
| CHEMBL1089318 | 413.1691 | 0.8825 | 5 | 2 | 3 | 149.46 |
| CHEMBL166863 | 361.3873 | 2.2293 | 4 | 1 | 3 | 94.84 |
| CHEMBL1771 | 321.8217 | 3.5082 | 2 | 0 | 4 | 57.78 |
| CHEMBL468895 | 534.673 | 1.55 | 5 | 2 | 10 | 163.64 |
| CHEMBL3661404 | 240.297 | 2.9913 | 2 | 0 | 4 | 21.76 |
| CHEMBL187614 | 721.9904 | 4.5619 | 8 | 6 | 22 | 200.26 |
| CHEMBL115794 | 243.2498 | 1.8602 | 2 | 1 | 1 | 41.49 |
| CHEMBL1908332 | 459.6645 | 4.1049 | 3 | 1 | 3 | 69.72 |
| CHEMBL348609 | 560.679 | 6.5505 | 8 | 1 | 14 | 99.16 |
| CHEMBL451887 | 719.9099 | 3.6594 | 7 | 4 | 20 | 158.47 |
| CHEMBL1029 | 219.278 | -0.5754 | 4 | 4 | 4 | 84.16 |
| CHEMBL272980 | 396.4445 | 2.7989 | 4 | 3 | 6 | 105.82 |
| CHEMBL1694 | 424.4895 | 3.3011 | 3 | 0 | 10 | 95.94 |
| CHEMBL1615369 | 627.7332 | 5.6773 | 8 | 1 | 17 | 154.03 |
| CHEMBL3143749 | 753.9246 | 4.7311 | 9 | 6 | 23 | 201.26 |
| CHEMBL185777 | 753.9892 | 3.5461 | 9 | 6 | 22 | 217.48 |
| CHEMBL953 | 305.286 | 1.6081 | 4 | 2 | 5 | 127.7 |
| CHEMBL3989967 | 472.4926 | 4.2721 | 7 | 2 | 8 | 111.67 |
| CHEMBL76222 | 499.6671 | 1.6979 | 5 | 4 | 11 | 166.72 |
| CHEMBL809 | 306.2296 | 4.9963 | 0 | 0 | 2 | 12.03 |
| CHEMBL1201127 | 547.6636 | 2.8264 | 7 | 3 | 12 | 148.8 |
| CHEMBL4297615 | 432.8791 | 2.0639 | 5 | 2 | 5 | 107.95 |
| CHEMBL1096979 | 257.2432 | -1.6309 | 5 | 6 | 5 | 148.07 |
| CHEMBL1503 | 345.4161 | 2.889 | 4 | 1 | 5 | 108.39 |
| CHEMBL246600 | 334.3637 | 2.8259 | 6 | 2 | 7 | 77.38 |
| CHEMBL75094 | 423.5064 | 1.3481 | 7 | 2 | 5 | 142.51 |
| CHEMBL1330792 | 275.0903 | 0.1475 | 4 | 4 | 2 | 106.86 |
| CHEMBL3187812 | 432.5331 | 2.563 | 4 | 1 | 6 | 129.08 |
| CHEMBL4535757 | 560.5944 | 6.6424 | 6 | 1 | 5 | 104.45 |
| CHEMBL4130229 | 453.9196 | 5.8035 | 3 | 2 | 4 | 71.09 |
| CHEMBL273264 | 347.3706 | 2.1608 | 4 | 2 | 6 | 138.07 |
| CHEMBL1467 | 136.1115 | -0.837 | 3 | 2 | 0 | 74.43 |
| CHEMBL1115 | 181.2117 | 1.564 | 2 | 0 | 2 | 33.42 |
| CHEMBL4650443 | 424.3819 | 2.4254 | 5 | 0 | 4 | 79.24 |
| CHEMBL2111084 | 433.4996 | 3.5977 | 5 | 1 | 5 | 93.45 |
| CHEMBL271068 | 400.453 | 3.9993 | 4 | 1 | 5 | 116.02 |
| CHEMBL1908391 | 498.6424 | 4.2931 | 3 | 2 | 7 | 101.63 |
| CHEMBL2105741 | 615.3948 | 5.8093 | 4 | 2 | 5 | 107.13 |
| CHEMBL4459585 | 383.4093 | 0.3746 | 6 | 0 | 4 | 113.93 |
| CHEMBL1928262 | 519.617 | 1.2781 | 8 | 1 | 10 | 143.92 |
| CHEMBL4296717 | 349.4048 | 1.3406 | 4 | 2 | 6 | 100.02 |
| CHEMBL3989908 | 473.375 | 4.8785 | 6 | 3 | 8 | 108.74 |
| CHEMBL4650343 | 402.4954 | 2.7139 | 4 | 3 | 6 | 105.55 |
| CHEMBL206834 | 576.6153 | 4.6258 | 5 | 2 | 9 | 99.17 |
| CHEMBL4650319 | 585.6966 | 4.9187 | 6 | 2 | 13 | 113.85 |
| CHEMBL2103842 | 466.9433 | 4.3983 | 6 | 1 | 7 | 121.79 |
| CHEMBL2110666 | 373.3716 | 2.597 | 2 | 0 | 5 | 70.12 |
| CHEMBL3989958 | 582.9609 | 3.0076 | 6 | 1 | 8 | 119.29 |
| CHEMBL3916243 | 367.4001 | 2.3478 | 5 | 1 | 3 | 129.46 |
| CHEMBL1236682 | 572.3372 | 2.8277 | 5 | 4 | 9 | 116.27 |
| CHEMBL4206119 | 629.8105 | 5.3756 | 7 | 2 | 12 | 163.01 |
| CHEMBL4297187 | 468.5652 | 5.8876 | 3 | 1 | 9 | 59.07 |
| CHEMBL2105987 | 298.4192 | 3.5036 | 2 | 0 | 0 | 34.14 |
| CHEMBL3989914 | 619.7046 | 6.316 | 5 | 2 | 13 | 157.73 |
| CHEMBL3707235 | 489.363 | 1.2908 | 4 | 0 | 6 | 92.42 |
| CHEMBL1236962 | 505.496 | 3.7086 | 7 | 1 | 6 | 115.34 |
| CHEMBL488 | 232.2783 | 1.2851 | 2 | 2 | 2 | 72.19 |
| CHEMBL552212 | 434.534 | 4.4139 | 5 | 3 | 9 | 101.06 |
| CHEMBL4650272 | 446.548 | 3.1793 | 4 | 2 | 3 | 91.21 |
| CHEMBL3039517 | 436.303 | 2.5283 | 5 | 2 | 5 | 97.4 |
| CHEMBL2177390 | 457.9962 | 3.1047 | 4 | 1 | 6 | 81.59 |
| CHEMBL2107357 | 373.4509 | 2.8513 | 3 | 3 | 5 | 78.94 |
| CHEMBL4297421 | 420.4378 | 4.2538 | 6 | 5 | 4 | 151.15 |
| CHEMBL1835207 | 356.4387 | 3.7809 | 3 | 1 | 5 | 82.7 |
| CHEMBL3989715 | 499.6142 | 1.9296 | 7 | 3 | 17 | 148.88 |
| CHEMBL4571518 | 423.2964 | 2.5973 | 4 | 2 | 6 | 88.07 |
| CHEMBL483254 | 349.4262 | 2.6833 | 2 | 3 | 7 | 77.15 |
| CHEMBL4204869 | 437.4387 | 2.0378 | 6 | 1 | 4 | 127.79 |
| CHEMBL3590106 | 433.331 | 3.7536 | 3 | 4 | 7 | 90.04 |
| CHEMBL4594253 | 344.3866 | 0.925 | 3 | 2 | 3 | 97.86 |
| CHEMBL3989921 | 557.0427 | 4.7747 | 6 | 2 | 11 | 112.4 |
| CHEMBL812 | 181.2117 | 1.564 | 2 | 0 | 2 | 33.42 |
| CHEMBL99 | 302.3682 | 2.6401 | 3 | 2 | 6 | 69.64 |
| CHEMBL563 | 244.2609 | 3.6556 | 0 | 0 | 3 | 37.3 |
| CHEMBL1201772 | 373.4411 | 3.8515 | 3 | 0 | 6 | 74.85 |
| CHEMBL3989931 | 473.375 | 4.8785 | 6 | 3 | 8 | 108.74 |
| CHEMBL220491 | 383.5072 | -0.3329 | 5 | 1 | 7 | 163.8 |
| CHEMBL304087 | 452.4599 | 2.857 | 7 | 3 | 8 | 123.95 |
| CHEMBL2079588 | 409.8257 | 3.6393 | 6 | 2 | 6 | 102.17 |
| CHEMBL1980297 | 488.5366 | 3.6288 | 4 | 4 | 6 | 146.33 |
| CHEMBL1201202 | 1508.263 | -12.1529 | 29 | 9 | 30 | 872.52 |
| CHEMBL3989922 | 320.3883 | 2.1165 | 2 | 1 | 3 | 72.94 |
| CHEMBL3544983 | 491.3853 | 5.9375 | 4 | 1 | 6 | 59.51 |
| CHEMBL3301615 | 209.2863 | 2.2703 | 0 | 0 | 1 | 12.03 |
| CHEMBL885 | 247.2467 | 0.8547 | 5 | 2 | 2 | 115.67 |
| CHEMBL371064 | 411.5404 | 2.7484 | 3 | 2 | 7 | 88.47 |
| CHEMBL404 | 300.2911 | -1.3958 | 5 | 0 | 3 | 130.84 |
| CHEMBL511099 | 173.2542 | 1.8481 | 0 | 0 | 1 | 12.03 |
| CHEMBL924 | 272.0896 | -1.4928 | 6 | 1 | 4 | 172.73 |
| CHEMBL2110581 | 6610.071 | -17.3388 | 173 | 45 | 122 | 3329.32 |
| CHEMBL4594449 | 647.6319 | 1.912 | 5 | 5 | 15 | 239.32 |
| CHEMBL3702854 | 665.7597 | 3.8505 | 7 | 1 | 7 | 138.66 |
| CHEMBL4435170 | 425.4167 | 1.0089 | 7 | 3 | 7 | 135.95 |
| CHEMBL4594292 | 492.5716 | 4.0378 | 6 | 0 | 7 | 96.93 |
| CHEMBL3545068 | 480.5196 | 0.4103 | 8 | 1 | 8 | 139.79 |
| CHEMBL603469 | 439.4626 | 2.9157 | 4 | 3 | 1 | 88.65 |
| CHEMBL1324 | 273.2408 | 3.08 | 3 | 2 | 3 | 100.67 |
| CHEMBL766 | 358.453 | 0.9691 | 2 | 1 | 11 | 155.02 |
| CHEMBL1515 | 114.1688 | 1.183 | 0 | 1 | 0 | 52.81 |
| CHEMBL3707348 | 465.5065 | 2.9024 | 5 | 2 | 5 | 119.03 |
| CHEMBL1950289 | 448.4415 | 3.2703 | 5 | 3 | 5 | 97.12 |
| CHEMBL4594420 | 495.4284 | 4.394 | 3 | 4 | 4 | 98.91 |
| CHEMBL4650317 | 526.3944 | 3.702 | 5 | 1 | 7 | 125.41 |
| CHEMBL3622533 | 508.5529 | 2.1932 | 9 | 2 | 7 | 166.96 |
| CHEMBL183419 | 27.0253 | 0.5675 | 1 | 0 | 0 | 23.79 |
| CHEMBL3301622 | 552.7115 | 2.3751 | 5 | 3 | 9 | 121.11 |
| CHEMBL4297374 | 618.5265 | 3.0978 | 5 | 3 | 4 | 135.44 |
| CHEMBL4292264 | 618.5265 | 3.0978 | 5 | 3 | 4 | 135.44 |
| CHEMBL84 | 421.4458 | 2.8187 | 6 | 2 | 3 | 104.89 |
| CHEMBL31634 | 333.1353 | 0.2014 | 5 | 3 | 3 | 104.55 |
| CHEMBL783 | 317.4226 | 3.9562 | 1 | 1 | 6 | 66.4 |
| CHEMBL3545311 | 584.0924 | 4.1869 | 3 | 2 | 8 | 95.67 |
| CHEMBL2105719 | 469.939 | 4.9518 | 4 | 2 | 7 | 79.38 |
| CHEMBL221959 | 312.3696 | 0.9045 | 4 | 1 | 3 | 88.91 |
| CHEMBL1614644 | 296.4067 | 2.1367 | 0 | 0 | 4 | 26.3 |
| CHEMBL238804 | 496.6217 | 3.999 | 6 | 1 | 12 | 109.87 |
| CHEMBL3137315 | 652.5603 | 5.8194 | 4 | 1 | 7 | 117.76 |
| CHEMBL4297584 | 415.4197 | 4.3099 | 5 | 2 | 5 | 96.7 |
| CHEMBL4567446 | 502.5616 | 3.4906 | 5 | 0 | 5 | 114.62 |
| CHEMBL3545308 | 555.5515 | 4.2306 | 5 | 3 | 9 | 111.72 |
| CHEMBL779 | 389.404 | 1.8048 | 4 | 1 | 1 | 74.87 |
| CHEMBL939 | 446.9024 | 4.206 | 5 | 1 | 8 | 68.74 |
| CHEMBL4297507 | 310.3537 | 0.1911 | 4 | 1 | 3 | 88.91 |
| CHEMBL3989947 | 481.6221 | 6.5908 | 7 | 0 | 22 | 95.28 |
| CHEMBL1090771 | 520.885 | 3.2416 | 6 | 1 | 10 | 127.77 |
| CHEMBL838 | 424.4895 | 3.3011 | 3 | 0 | 10 | 95.94 |
| CHEMBL14762 | 354.4493 | 3.1839 | 4 | 3 | 8 | 87.89 |
| CHEMBL427340 | 568.7043 | 3.9155 | 6 | 5 | 17 | 159.85 |
| CHEMBL435966 | 226.2325 | 0.0034 | 2 | 0 | 4 | 73.43 |
| CHEMBL19 | 236.272 | -0.2287 | 4 | 1 | 1 | 144.03 |
| CHEMBL114 | 670.8408 | 3.6358 | 6 | 5 | 13 | 166.75 |
| CHEMBL2103828 | 523.945 | 4.7726 | 5 | 4 | 5 | 178.78 |
| CHEMBL1770248 | 436.8827 | 2.1033 | 7 | 4 | 6 | 108.61 |
| CHEMBL2103798 | 450.5053 | 4.3853 | 5 | 1 | 8 | 72.5 |
| CHEMBL231779 | 459.4971 | 2.5005 | 5 | 1 | 5 | 110.76 |
| CHEMBL266349 | 429.5126 | 0.5153 | 3 | 2 | 9 | 148.61 |
| CHEMBL3916717 | 445.4921 | 3.8751 | 5 | 1 | 6 | 90.52 |
| CHEMBL2103880 | 409.5013 | 3.8778 | 4 | 1 | 7 | 83.67 |
| CHEMBL46618 | 446.4983 | 0.0248 | 4 | 2 | 9 | 132.21 |
| CHEMBL325041 | 384.2372 | 1.8125 | 6 | 4 | 9 | 124.44 |
| CHEMBL3410450 | 532.0581 | 2.1237 | 5 | 1 | 5 | 134.83 |
| CHEMBL467058 | 472.5325 | 0.1289 | 7 | 3 | 16 | 193.29 |
| CHEMBL1201740 | 529.5158 | 4.9019 | 5 | 2 | 7 | 97.62 |
| CHEMBL4802152 | 478.4508 | 3.5064 | 5 | 1 | 4 | 104.19 |
| CHEMBL3143752 | 767.9511 | 4.9829 | 9 | 6 | 23 | 201.26 |
| CHEMBL4448462 | 376.4732 | -0.1138 | 4 | 0 | 3 | 104.98 |
| CHEMBL275231 | 662.8173 | 2.0786 | 8 | 7 | 19 | 218.05 |
| CHEMBL189134 | 477.6367 | 3.9711 | 5 | 4 | 14 | 130.75 |
| CHEMBL3143747 | 711.8448 | 3.3562 | 9 | 6 | 21 | 201.26 |
| CHEMBL590754 | 292.3303 | 2.7748 | 5 | 1 | 6 | 83.14 |
| CHEMBL362324 | 640.8316 | 3.0967 | 8 | 5 | 18 | 188.38 |
| CHEMBL2103936 | 600.6484 | 6.8632 | 5 | 0 | 12 | 104.77 |
| CHEMBL1164729 | 316.3748 | 3.3981 | 3 | 0 | 5 | 111.45 |
| CHEMBL2105720 | 404.281 | 4.2322 | 2 | 0 | 5 | 93.31 |
| CHEMBL4285417 | 412.5086 | 1.2799 | 4 | 2 | 4 | 116.23 |
| CHEMBL2219422 | 427.3232 | 3.7408 | 2 | 1 | 6 | 101.18 |
| CHEMBL1308 | 82.1038 | 0.2896 | 1 | 1 | 0 | 28.68 |
| CHEMBL374731 | 242.2286 | -0.0029 | 5 | 3 | 2 | 104.55 |
| CHEMBL370753 | 363.4959 | 3.3612 | 1 | 1 | 6 | 29.95 |
| CHEMBL3707393 | 227.237 | 1.9754 | 2 | 0 | 2 | 41.61 |
| CHEMBL72 | 266.3807 | 3.852 | 0 | 0 | 4 | 15.27 |
| CHEMBL4297190 | 480.5825 | 3.3414 | 5 | 3 | 10 | 120.62 |
| CHEMBL2338397 | 464.4376 | 1.3978 | 5 | 3 | 8 | 111.63 |
| CHEMBL405963 | 1267.388 | 1.9124 | 13 | 8 | 32 | 417.83 |
| CHEMBL423875 | 347.384 | 0.9235 | 7 | 0 | 6 | 115.43 |
| CHEMBL3298910 | 557.0064 | 8.0851 | 3 | 1 | 8 | 59.39 |
| CHEMBL3137317 | 485.4981 | 6.7353 | 4 | 1 | 6 | 63.69 |
| CHEMBL3982690 | 437.4931 | 3.3892 | 6 | 2 | 5 | 100.72 |
| CHEMBL192 | 474.5764 | 2.1252 | 6 | 1 | 7 | 121.8 |
| CHEMBL2336325 | 462.5441 | 3.3698 | 6 | 1 | 4 | 92.71 |
| CHEMBL18116 | 207.2258 | 2.2136 | 3 | 1 | 2 | 49.77 |
| CHEMBL1159823 | 1041.176 | -0.7839 | 15 | 8 | 18 | 409.49 |
| CHEMBL2105279 | 338.4036 | 3.4474 | 3 | 2 | 5 | 68.18 |
| CHEMBL571546 | 431.5268 | 3.2627 | 4 | 1 | 9 | 63.69 |
| CHEMBL222559 | 602.6643 | 6.9261 | 6 | 2 | 12 | 113.97 |
| CHEMBL690 | 423.5198 | 1.4538 | 5 | 3 | 11 | 127.12 |
| CHEMBL3039513 | 392.3783 | 2.1403 | 4 | 3 | 7 | 95.59 |
| CHEMBL1851943 | 358.4778 | 3.5332 | 3 | 2 | 10 | 70.39 |
| CHEMBL363255 | 979.0813 | -0.8797 | 8 | 7 | 33 | 399.15 |
| CHEMBL2005186 | 452.5078 | 4.1801 | 5 | 3 | 7 | 104.82 |
| CHEMBL4297423 | 540.1232 | 3.3679 | 4 | 1 | 7 | 106.89 |
| CHEMBL1678 | 379.492 | 4.5697 | 3 | 0 | 6 | 38.77 |
| CHEMBL1079742 | 393.4357 | 4.3126 | 6 | 2 | 10 | 74.73 |
| CHEMBL4072833 | 429.5142 | 3.815 | 4 | 2 | 7 | 93.37 |
| CHEMBL4297497 | 446.4406 | 4.0092 | 2 | 0 | 7 | 64.15 |
| CHEMBL4802130 | 452.5078 | 4.1801 | 5 | 3 | 7 | 104.82 |
| CHEMBL3545376 | 446.5447 | 3.4727 | 5 | 0 | 9 | 77.33 |
| CHEMBL227529 | 339.3916 | 2.7106 | 3 | 0 | 3 | 97.05 |
| CHEMBL376359 | 339.3916 | 2.7106 | 3 | 0 | 3 | 97.05 |
| CHEMBL4594251 | 453.5323 | 3.9008 | 4 | 1 | 8 | 94.31 |
| CHEMBL4442620 | 430.4445 | 3.6218 | 4 | 3 | 8 | 116.57 |
| CHEMBL3989973 | 417.8148 | 4.2043 | 3 | 2 | 6 | 66.49 |
| CHEMBL1401 | 307.282 | 1.6335 | 4 | 1 | 5 | 139.67 |
| CHEMBL1278146 | 410.4845 | 2.7799 | 2 | 2 | 6 | 68.44 |
| CHEMBL3991932 | 556.6305 | 6.552 | 5 | 3 | 9 | 106.23 |
| CHEMBL249856 | 248.3009 | 2.4468 | 2 | 2 | 3 | 91.02 |
| CHEMBL3301612 | 540.0107 | 3.369 | 7 | 3 | 10 | 148.51 |
| CHEMBL3545154 | 491.3422 | 4.9429 | 5 | 1 | 6 | 76.58 |
| CHEMBL2103840 | 396.486 | 0.2206 | 3 | 2 | 7 | 93.15 |
| CHEMBL3301595 | 292.203 | 4.5645 | 0 | 0 | 1 | 26.02 |
| CHEMBL3039502 | 416.863 | 4.161 | 4 | 2 | 4 | 88.49 |
| CHEMBL3317857 | 297.1351 | 1.368 | 3 | 2 | 5 | 124.1 |
| CHEMBL853 | 211.2178 | 0.248 | 4 | 2 | 2 | 90.37 |
| CHEMBL3137303 | 472.4028 | 0.9132 | 8 | 0 | 10 | 161.67 |
| CHEMBL1332032 | 248.1554 | 2.1444 | 2 | 0 | 4 | 63.6 |
| CHEMBL166 | 398.3387 | 0.7731 | 4 | 4 | 4 | 173.57 |
| CHEMBL4297596 | 487.3848 | 5.6676 | 4 | 0 | 4 | 116.44 |
| CHEMBL592445 | 615.7258 | 3.47 | 7 | 2 | 7 | 128.29 |
| CHEMBL2374220 | 888.9999 | 6.9016 | 8 | 4 | 12 | 174.64 |
| CHEMBL887 | 171.2383 | 3.1195 | 0 | 1 | 3 | 12.03 |
| CHEMBL2103810 | 529.6483 | 2.3402 | 7 | 2 | 17 | 162.12 |
| CHEMBL4594441 | 395.387 | 5.3993 | 5 | 3 | 5 | 105.07 |
| CHEMBL3989553 | 347.3706 | 2.1608 | 4 | 2 | 6 | 138.07 |
| CHEMBL3989868 | 480.5212 | 4.2602 | 7 | 1 | 6 | 111.37 |
| CHEMBL255863 | 529.5158 | 4.9019 | 5 | 2 | 7 | 97.62 |
| CHEMBL2104981 | 735.805 | 8.4848 | 3 | 0 | 13 | 80.08 |
| CHEMBL4081711 | 361.3675 | 1.478 | 5 | 2 | 6 | 103.54 |
| CHEMBL515387 | 366.4089 | 0.1712 | 3 | 0 | 3 | 64.6 |
| CHEMBL4297642 | 534.6068 | 3.0393 | 5 | 2 | 13 | 142.06 |
| CHEMBL1615993 | 365.2497 | 3.2115 | 6 | 1 | 4 | 118.66 |
| CHEMBL3639788 | 571.5741 | 5.6001 | 8 | 2 | 13 | 160.12 |
| CHEMBL1477 | 459.5503 | 4.1793 | 4 | 2 | 11 | 99.88 |
| CHEMBL1086218 | 1214.622 | 4.7569 | 12 | 4 | 15 | 275.64 |
| CHEMBL2105721 | 415.4149 | 1.9887 | 7 | 2 | 5 | 110.11 |
| CHEMBL1823872 | 1637.878 | -4.1389 | 17 | 18 | 26 | 663.83 |
| CHEMBL4297589 | 618.6184 | 2.09 | 9 | 1 | 12 | 184.54 |
| CHEMBL1255891 | 707.9638 | 4.0001 | 7 | 4 | 15 | 179.23 |
| CHEMBL3218576 | 480.5196 | 0.4103 | 8 | 1 | 8 | 139.79 |
| CHEMBL3334567 | 345.3613 | 0.5286 | 6 | 0 | 3 | 92.13 |
| CHEMBL1205 | 567.7824 | 5.2596 | 4 | 4 | 10 | 127.2 |
| CHEMBL4297524 | 530.5038 | 4.1798 | 6 | 2 | 7 | 110.51 |
| CHEMBL2364625 | 454.4642 | 5.3148 | 2 | 1 | 7 | 77.94 |
| CHEMBL2107823 | 469.9423 | 3.7973 | 4 | 2 | 6 | 83.89 |
| CHEMBL4297504 | 499.6389 | 3.9221 | 6 | 0 | 12 | 73.06 |
| CHEMBL313006 | 389.5945 | 7.0256 | 2 | 1 | 9 | 71.47 |
| CHEMBL1540 | 253.2578 | -0.9169 | 6 | 4 | 5 | 130.31 |
| CHEMBL1328913 | 294.4338 | 4.3897 | 0 | 0 | 4 | 6.48 |
| CHEMBL1201168 | 231.2505 | 1.1841 | 3 | 2 | 4 | 67.16 |
| CHEMBL4650485 | 479.4275 | 3.0878 | 4 | 3 | 8 | 125.26 |
| CHEMBL59356 | 318.3659 | 4.1087 | 2 | 2 | 4 | 94.8 |
| CHEMBL2103825 | 401.5821 | 9.1773 | 4 | 0 | 16 | 52.33 |
| CHEMBL541388 | 291.3687 | 0.1886 | 3 | 0 | 2 | 70.68 |
| CHEMBL429910 | 408.8726 | 2.4099 | 6 | 4 | 6 | 99.38 |
| CHEMBL2105015 | 308.7649 | 2.7829 | 2 | 1 | 3 | 46.5 |
| CHEMBL389433 | 308.7649 | 2.7829 | 2 | 1 | 3 | 46.5 |
| CHEMBL2360464 | 425.3948 | -0.6949 | 3 | 4 | 9 | 196.92 |
| CHEMBL636 | 250.3367 | 3.1239 | 2 | 0 | 5 | 32.78 |
| CHEMBL294951 | 295.4417 | 4.1114 | 0 | 0 | 0 | 31.48 |
| CHEMBL490 | 329.3654 | 3.2298 | 3 | 0 | 4 | 39.72 |
| CHEMBL4297405 | 519.5178 | 2.6862 | 5 | 4 | 8 | 186.27 |
| CHEMBL1289494 | 454.8631 | 4.942 | 7 | 2 | 6 | 107.74 |
| CHEMBL479527 | 600.4733 | 8.4905 | 4 | 0 | 10 | 59.08 |
| CHEMBL2403238 | 405.3522 | 1.6412 | 5 | 2 | 3 | 100.87 |
| CHEMBL1206232 | 396.3283 | 3.2034 | 8 | 0 | 8 | 113.49 |
| CHEMBL1380 | 286.3323 | 0.6328 | 4 | 3 | 4 | 101.88 |
| CHEMBL4514636 | 503.3778 | 4.1231 | 6 | 2 | 7 | 94.6 |
| CHEMBL3301625 | 423.4402 | 3.9838 | 5 | 3 | 10 | 97.4 |
| CHEMBL273575 | 238.3275 | 2.5832 | 0 | 1 | 1 | 29.26 |
| CHEMBL4112930 | 298.3149 | 1.9658 | 2 | 2 | 0 | 64.78 |
| CHEMBL1538 | 519.4428 | 1.9261 | 13 | 1 | 17 | 195.25 |
| CHEMBL814 | 318.3346 | 2.5517 | 3 | 0 | 10 | 56.84 |
| CHEMBL4297648 | 482.8451 | 2.593 | 4 | 1 | 5 | 90.53 |
| CHEMBL57 | 266.2979 | 2.299 | 3 | 1 | 1 | 58.12 |
| CHEMBL1230165 | 349.7705 | 4.1348 | 2 | 1 | 3 | 75.11 |
| CHEMBL629 | 277.4033 | 4.772 | 0 | 0 | 3 | 3.24 |
| CHEMBL497 | 305.4996 | 2.9915 | 2 | 1 | 0 | 33.12 |
| CHEMBL2017974 | 410.3935 | 2.3477 | 5 | 1 | 4 | 89.63 |
| CHEMBL1922094 | 498.6011 | 0.9255 | 7 | 2 | 5 | 162.07 |
| CHEMBL859 | 152.1109 | -0.69 | 3 | 3 | 0 | 94.4 |
| CHEMBL3545366 | 270.2899 | 1.2143 | 4 | 2 | 2 | 102.48 |
| CHEMBL972 | 187.2808 | 3.7945 | 0 | 1 | 5 | 3.24 |
| CHEMBL2103879 | 364.3978 | 4.3759 | 4 | 3 | 3 | 96.07 |
| CHEMBL1650443 | 357.3821 | 2.9161 | 3 | 0 | 3 | 97.05 |
| CHEMBL419213 | 405.4879 | 1.4598 | 1 | 0 | 12 | 132.96 |
| CHEMBL2303621 | 738.875 | 4.7706 | 8 | 4 | 13 | 174.64 |
| CHEMBL3989937 | 412.4191 | 2.8713 | 5 | 1 | 4 | 85.59 |
| CHEMBL1078685 | 248.2428 | 0.762 | 5 | 1 | 3 | 91.14 |
| CHEMBL3301621 | 209.2863 | 2.2703 | 0 | 0 | 1 | 12.03 |
| CHEMBL1170047 | 292.0307 | 1.2734 | 1 | 1 | 2 | 86.23 |
| CHEMBL4297522 | 487.4991 | 3.3633 | 5 | 1 | 6 | 83.16 |
| CHEMBL2105659 | 394.4205 | 3.1822 | 5 | 1 | 9 | 100.62 |
| CHEMBL67279 | 214.0698 | 0.373 | 3 | 2 | 3 | 86.79 |
| CHEMBL1450 | 366.8375 | 5.2945 | 3 | 1 | 2 | 54.37 |
| CHEMBL4297629 | 481.9066 | 4.2918 | 7 | 1 | 5 | 93.49 |
| CHEMBL599872 | 755.9205 | 4.6469 | 9 | 3 | 7 | 188.9 |
| CHEMBL431 | 466.614 | 2.9764 | 5 | 0 | 10 | 146.54 |
| CHEMBL788 | 354.0985 | 0.0891 | 5 | 3 | 2 | 104.55 |
| CHEMBL338975 | 275.3461 | 2.6723 | 2 | 1 | 2 | 44.81 |
| CHEMBL3260567 | 399.4236 | 2.8004 | 4 | 2 | 5 | 84.31 |
| CHEMBL4069704 | 590.0363 | 6.064 | 5 | 1 | 9 | 102.6 |
| CHEMBL4650336 | 467.3439 | 7.0063 | 3 | 1 | 4 | 54.56 |
| CHEMBL2103860 | 572.6167 | 7.1927 | 3 | 1 | 9 | 58.64 |
| CHEMBL3545064 | 214.0698 | 0.373 | 3 | 2 | 3 | 86.79 |
| CHEMBL3693786 | 467.9479 | 4.5566 | 5 | 3 | 11 | 107.45 |
| CHEMBL142703 | 303.3993 | 0.1799 | 3 | 1 | 4 | 76.36 |
| CHEMBL110458 | 163.1717 | -2.44 | 4 | 4 | 1 | 92.95 |
| CHEMBL218394 | 519.6767 | 1.8408 | 5 | 4 | 10 | 150.7 |
| CHEMBL1743259 | 377.9115 | 3.9202 | 2 | 0 | 7 | 46.3 |
| CHEMBL481611 | 525.5588 | 3.9788 | 4 | 1 | 11 | 107.44 |
| CHEMBL504535 | 369.4772 | 3.7888 | 3 | 0 | 5 | 62.83 |
| CHEMBL1201057 | 357.4451 | 0.2913 | 2 | 1 | 11 | 157.85 |
| CHEMBL2107355 | 163.1717 | -2.44 | 4 | 4 | 1 | 92.95 |
| CHEMBL1232767 | 20.0063 | -0.1036 | 0 | 0 | 0 | 0 |
| CHEMBL880 | 321.3318 | -0.4331 | 7 | 1 | 9 | 122.22 |
| CHEMBL1201182 | 1030.287 | 5.6197 | 16 | 4 | 11 | 241.96 |
| CHEMBL3617964 | 545.5335 | 3.1802 | 6 | 1 | 7 | 124.33 |
| CHEMBL637 | 277.4018 | 3.0221 | 2 | 1 | 5 | 32.7 |
| CHEMBL27759 | 376.4085 | 2.266 | 4 | 3 | 7 | 106.34 |
| CHEMBL4297458 | 495.6153 | 4.9559 | 5 | 1 | 5 | 77.99 |
| CHEMBL1709 | 306.2296 | 4.9963 | 0 | 0 | 2 | 12.03 |
| CHEMBL1269025 | 548.0575 | 0.8018 | 6 | 3 | 6 | 164.87 |
| CHEMBL3348963 | 574.9819 | 5.2389 | 6 | 1 | 11 | 131.17 |
| CHEMBL17 | 305.1588 | 0.5064 | 4 | 2 | 2 | 137.08 |
| CHEMBL2103884 | 532.6092 | -0.3865 | 8 | 3 | 14 | 176.49 |
| CHEMBL4297636 | 571.7049 | 3.7808 | 7 | 3 | 16 | 146.05 |
| CHEMBL502835 | 539.6248 | 3.0932 | 4 | 2 | 8 | 94.22 |
| CHEMBL4297674 | 370.4206 | 2.6196 | 2 | 3 | 4 | 91.22 |
| CHEMBL520733 | 361.4354 | 0.6323 | 4 | 3 | 5 | 98.74 |
| CHEMBL2180602 | 424.5111 | 3.2775 | 2 | 3 | 6 | 77.23 |
| CHEMBL3545063 | 499.6073 | 4.337 | 4 | 2 | 10 | 87.55 |
| CHEMBL1201174 | 407.3136 | 1.9304 | 3 | 0 | 5 | 77.04 |
| CHEMBL1422 | 407.3136 | 1.9304 | 3 | 0 | 5 | 77.04 |
| CHEMBL3112741 | 348.3754 | -0.889 | 4 | 1 | 4 | 136.66 |
| CHEMBL4802156 | 586.7292 | 3.994 | 4 | 1 | 6 | 119.19 |
| CHEMBL1233528 | 618.8126 | 4.8416 | 5 | 2 | 10 | 106.17 |
| CHEMBL189963 | 447.5328 | 3.5623 | 5 | 1 | 5 | 105.04 |
| CHEMBL411907 | 525.5901 | 1.57 | 8 | 1 | 9 | 119.44 |
| CHEMBL1444 | 285.3027 | 2.065 | 4 | 0 | 4 | 78.29 |
| CHEMBL2364614 | 366.4583 | 4.3623 | 2 | 1 | 2 | 55.04 |
| CHEMBL522038 | 473.5652 | 1.1024 | 6 | 2 | 11 | 146.35 |
| CHEMBL2110585 | 529.6483 | 2.3402 | 7 | 2 | 17 | 162.12 |
| CHEMBL578 | 376.4467 | 2.1388 | 3 | 0 | 10 | 95.94 |
| CHEMBL2364628 | 433.5029 | 3.8922 | 5 | 3 | 11 | 107.45 |
| CHEMBL1560 | 217.2853 | 0.6422 | 1 | 1 | 3 | 96.41 |
| CHEMBL2325741 | 428.9152 | 0.7097 | 4 | 3 | 6 | 120.16 |
| CHEMBL3989891 | 252.2448 | 2.0852 | 2 | 0 | 3 | 66.16 |
| CHEMBL4208229 | 449.8384 | 3.8654 | 5 | 3 | 6 | 103.37 |
| CHEMBL2105671 | 147.1723 | -1.9016 | 3 | 3 | 1 | 72.72 |
| CHEMBL4297587 | 439.4826 | 2.3623 | 3 | 3 | 3 | 97.54 |
| CHEMBL514800 | 460.5002 | 1.509 | 7 | 1 | 8 | 127.46 |
| CHEMBL1200759 | 256.0881 | 1.9651 | 2 | 0 | 0 | 44.7 |
| CHEMBL1083385 | 321.8217 | 3.5082 | 2 | 0 | 4 | 57.78 |
| CHEMBL252164 | 465.5414 | 3.9732 | 6 | 3 | 7 | 108.06 |
| CHEMBL4594350 | 604.1174 | 5.3284 | 5 | 0 | 7 | 88.83 |
| CHEMBL334830 | 420.1893 | 1.7048 | 4 | 1 | 2 | 88.48 |
| CHEMBL2103830 | 580.4595 | 3.6025 | 12 | 2 | 10 | 196.53 |
| CHEMBL1201142 | 171.2383 | 3.1195 | 0 | 1 | 3 | 12.03 |
| CHEMBL4297638 | 603.5366 | 2.4199 | 6 | 1 | 8 | 133.54 |
| CHEMBL4297163 | 454.4806 | 4.3319 | 5 | 1 | 5 | 108.27 |
| CHEMBL3137301 | 411.4908 | 3.7333 | 3 | 1 | 12 | 92.7 |
| CHEMBL3286830 | 406.413 | 2.0124 | 5 | 1 | 0 | 110.06 |
| CHEMBL4802155 | 336.3877 | 2.1384 | 4 | 1 | 7 | 86.2 |
| CHEMBL3663929 | 562.7063 | 2.6241 | 5 | 2 | 9 | 120.16 |
| CHEMBL1561 | 207.2243 | -2.4435 | 5 | 5 | 3 | 104.39 |
| CHEMBL2105804 | 531.6872 | 6.7721 | 3 | 2 | 8 | 67.15 |
| CHEMBL1257015 | 376.4085 | 1.0956 | 5 | 2 | 7 | 93.21 |
| CHEMBL52939 | 429.506 | 4.055 | 4 | 0 | 14 | 85.3 |
| CHEMBL3674570 | 461.5991 | 4.227 | 5 | 2 | 4 | 103.69 |
| CHEMBL1293 | 536.8726 | 11.9986 | 0 | 0 | 10 | 0 |
| CHEMBL41 | 309.3261 | 4.0333 | 1 | 0 | 7 | 21.26 |
| CHEMBL498847 | 185.1805 | 0.2759 | 2 | 1 | 3 | 81.19 |
| CHEMBL139367 | 328.4072 | -0.1266 | 3 | 3 | 8 | 148.53 |
| CHEMBL2018096 | 404.4518 | 2.26 | 5 | 4 | 4 | 118.39 |
| CHEMBL160038 | 273.2408 | 2.6286 | 3 | 2 | 4 | 100.67 |
| CHEMBL4071161 | 454.4806 | 4.3319 | 5 | 1 | 5 | 108.27 |
| CHEMBL1200814 | 221.2375 | -0.7139 | 5 | 2 | 2 | 125.64 |
| CHEMBL1201773 | 373.4411 | 3.8515 | 3 | 0 | 6 | 74.85 |
| CHEMBL237500 | 472.5422 | 3.4831 | 5 | 0 | 5 | 116.86 |
| CHEMBL1213492 | 421.4889 | 4.3186 | 4 | 3 | 9 | 90.9 |
| CHEMBL1229211 | 419.3788 | 1.7036 | 5 | 2 | 3 | 100.87 |
| CHEMBL2316582 | 532.5595 | 4.4472 | 3 | 2 | 6 | 77.15 |
| CHEMBL175247 | 495.7348 | 8.528 | 5 | 1 | 23 | 81.7 |
| CHEMBL364635 | 707.9638 | 4.4995 | 8 | 6 | 21 | 200.26 |
| CHEMBL121810 | 381.4681 | 5.3151 | 3 | 1 | 4 | 54.04 |
| CHEMBL87708 | 418.9584 | 6.3082 | 1 | 0 | 7 | 23.55 |
| CHEMBL1287853 | 524.6781 | 5.1262 | 5 | 3 | 11 | 116.86 |
| CHEMBL1201753 | 421.4608 | 3.9286 | 3 | 2 | 8 | 90.65 |
| CHEMBL503749 | 2111.421 | -8.1694 | 33 | 15 | 100 | 722.37 |
| CHEMBL403 | 233.2416 | -0.6958 | 3 | 0 | 1 | 100.13 |
| CHEMBL1241855 | 451.4901 | 2.0808 | 6 | 1 | 11 | 128.77 |
| CHEMBL3137308 | 326.3929 | 0.3823 | 3 | 4 | 3 | 104.03 |
| CHEMBL4297627 | 380.4187 | 1.9678 | 4 | 1 | 0 | 75.94 |
| CHEMBL1236524 | 265.2685 | -2.0944 | 5 | 5 | 2 | 140.31 |
| CHEMBL3391662 | 765.8771 | 3.1067 | 10 | 3 | 7 | 198.03 |
| CHEMBL3989650 | 240.3434 | 2.554 | 0 | 0 | 7 | 24.06 |
| CHEMBL1421 | 488.0055 | 3.3995 | 5 | 3 | 7 | 134.75 |
| CHEMBL1188395 | 295.3508 | 2.8387 | 1 | 1 | 2 | 82.64 |
| CHEMBL4297181 | 406.4775 | 3.3222 | 5 | 1 | 5 | 82.17 |
| CHEMBL4297066 | 404.5429 | 3.8087 | 4 | 2 | 11 | 71.03 |
| CHEMBL3137336 | 429.248 | 3.3881 | 3 | 1 | 6 | 86.08 |
| CHEMBL477772 | 437.518 | 3.379 | 5 | 2 | 5 | 127.41 |
| CHEMBL107747 | 378.4857 | 2.0701 | 2 | 2 | 6 | 125.51 |
| CHEMBL445 | 263.3767 | 4.2361 | 0 | 0 | 3 | 12.03 |
| CHEMBL2364626 | 420.5257 | 5.4723 | 3 | 2 | 5 | 85.41 |
| CHEMBL3039522 | 806.9408 | 2.3228 | 6 | 6 | 15 | 194.92 |
| CHEMBL3707349 | 539.691 | 4.5795 | 3 | 3 | 9 | 118.36 |
| CHEMBL3265032 | 411.4591 | 2.8162 | 4 | 2 | 4 | 83.89 |
| CHEMBL4297377 | 628.2743 | 6.3483 | 6 | 1 | 8 | 121.71 |
| CHEMBL2063090 | 766.9034 | 4.1725 | 11 | 3 | 8 | 203.6 |
| CHEMBL270995 | 441.4555 | 3.0074 | 6 | 1 | 8 | 117.28 |
| CHEMBL494 | 360.4871 | 4.1192 | 2 | 2 | 9 | 77.76 |
| CHEMBL1165 | 498.5681 | 3.3032 | 5 | 0 | 12 | 114.4 |
| CHEMBL4297511 | 368.5142 | -1.3312 | 2 | 0 | 11 | 228.14 |
| CHEMBL4541964 | 405.7975 | 6.2753 | 1 | 0 | 6 | 50.19 |
| CHEMBL4297593 | 496.8916 | 2.0593 | 6 | 3 | 10 | 123.98 |
| CHEMBL870 | 249.096 | -1.3044 | 5 | 1 | 5 | 180.93 |
| CHEMBL591118 | 377.453 | 0.7765 | 3 | 0 | 4 | 68.03 |
| CHEMBL521851 | 513.6356 | 1.7324 | 6 | 1 | 5 | 144.17 |
| CHEMBL197672 | 569.5021 | 2.8521 | 5 | 3 | 12 | 150.9 |
| CHEMBL4594348 | 297.3517 | 1.1878 | 2 | 2 | 3 | 79.19 |
| CHEMBL204656 | 447.8838 | 5.2305 | 3 | 1 | 7 | 88.76 |
| CHEMBL141 | 229.2562 | 0.6492 | 5 | 2 | 2 | 115.67 |
| CHEMBL1738797 | 482.6166 | 4.825 | 3 | 1 | 3 | 72.36 |
| CHEMBL2107779 | 744.8961 | 0.7446 | 10 | 6 | 23 | 355.24 |
| CHEMBL1256841 | 298.3397 | 0.3586 | 3 | 3 | 7 | 83.12 |
| CHEMBL4297644 | 341.4307 | 1.8685 | 3 | 2 | 2 | 105.91 |
| CHEMBL3931971 | 586.7028 | 3.7668 | 5 | 3 | 12 | 211.58 |
| CHEMBL4303201 | 407.4375 | 3.3264 | 4 | 1 | 6 | 82.39 |
| CHEMBL4297619 | 340.3333 | 0.9732 | 4 | 2 | 6 | 117.34 |
| CHEMBL4446378 | 613.207 | 6.3267 | 5 | 1 | 1 | 93.32 |
| CHEMBL2013119 | 450.4822 | 1.403 | 6 | 1 | 11 | 131.6 |
| CHEMBL2104969 | 745.2487 | 10.3605 | 2 | 1 | 15 | 96.78 |
| CHEMBL4650334 | 406.4264 | 2.2058 | 3 | 2 | 5 | 81.75 |
| CHEMBL991 | 224.2133 | 0.72 | 4 | 2 | 2 | 84.32 |
| CHEMBL4073443 | 340.3797 | 2.4104 | 4 | 3 | 2 | 114.93 |
| CHEMBL2105661 | 511.701 | 3.6025 | 6 | 1 | 11 | 153.65 |
| CHEMBL496978 | 531.4256 | 1.8303 | 1 | 2 | 14 | 165.06 |
| CHEMBL2364607 | 531.31 | 3.7016 | 2 | 2 | 4 | 64.6 |
| CHEMBL2146883 | 531.31 | 3.7016 | 2 | 2 | 4 | 64.6 |
| CHEMBL1581 | 368.4678 | 2.4117 | 3 | 0 | 9 | 95.94 |
| CHEMBL225304 | 74.0851 | -1.6338 | 1 | 1 | 0 | 90.42 |
| CHEMBL3301603 | 552.7115 | 2.3751 | 5 | 3 | 9 | 121.11 |
| CHEMBL393220 | 557.6319 | 4.7624 | 3 | 3 | 12 | 114.62 |
| CHEMBL3301601 | 409.4104 | 1.3144 | 5 | 2 | 3 | 123.63 |
| CHEMBL3989972 | 428.4185 | 3.6772 | 5 | 0 | 5 | 79.76 |
| CHEMBL1449676 | 219.6256 | 0.5923 | 2 | 1 | 4 | 81.19 |
| CHEMBL1818442 | 228.1177 | 2.6907 | 0 | 0 | 1 | 12.03 |
| CHEMBL501849 | 749.9391 | 4.6141 | 9 | 2 | 8 | 193.51 |
| CHEMBL1592 | 438.5161 | 3.336 | 3 | 0 | 10 | 95.94 |
| CHEMBL2105728 | 443.5408 | 3.1504 | 4 | 0 | 5 | 78.43 |
| CHEMBL2105756 | 454.8631 | 4.942 | 7 | 2 | 6 | 107.74 |
| CHEMBL4802239 | 585.6966 | 4.9187 | 6 | 2 | 13 | 113.85 |
| CHEMBL2105754 | 357.3821 | 2.9161 | 3 | 0 | 3 | 97.05 |
| CHEMBL3989910 | 299.7085 | 3.9646 | 2 | 1 | 2 | 70.42 |
| CHEMBL483158 | 518.9235 | 5.929 | 5 | 1 | 6 | 105.93 |
| CHEMBL4466233 | 452.5493 | 0.695 | 5 | 3 | 7 | 110.69 |
| CHEMBL3545369 | 438.2328 | 0.7972 | 7 | 4 | 8 | 176.14 |
| CHEMBL2105685 | 447.5095 | 4.7793 | 5 | 1 | 5 | 107.98 |
| CHEMBL331378 | 429.5523 | 3.9116 | 3 | 0 | 8 | 125.28 |
| CHEMBL4249337 | 483.361 | 2.6851 | 5 | 4 | 4 | 136.1 |
| CHEMBL1518 | 170.2321 | 2.1999 | 1 | 2 | 2 | 80.74 |
| CHEMBL562318 | 392.4525 | 4.2925 | 4 | 0 | 5 | 52.83 |
| CHEMBL2110627 | 476.6088 | 2.8574 | 4 | 0 | 11 | 149.48 |
| CHEMBL1201320 | 345.4161 | 2.5713 | 4 | 1 | 5 | 96.31 |
| CHEMBL1908360 | 958.2244 | 5.7448 | 14 | 3 | 9 | 204.66 |
| CHEMBL193240 | 403.2075 | 4.4022 | 4 | 1 | 7 | 60.45 |
| CHEMBL222813 | 332.3098 | -3.6066 | 6 | 5 | 7 | 198.22 |
| CHEMBL4298138 | 355.3662 | 1.9169 | 4 | 2 | 0 | 81.07 |
| CHEMBL554 | 581.0575 | 5.954 | 6 | 1 | 11 | 114.73 |
| CHEMBL53538 | 504.6024 | 1.5858 | 7 | 2 | 9 | 142.03 |
| CHEMBL1738758 | 532.5182 | 4.3772 | 6 | 3 | 8 | 134.66 |
| CHEMBL269732 | 804.0182 | 4.552 | 12 | 3 | 7 | 178.36 |
| CHEMBL1800807 | 637.5084 | 9.8678 | 3 | 0 | 9 | 38.77 |
| CHEMBL4204794 | 498.558 | 2.332 | 5 | 0 | 5 | 106.81 |
| CHEMBL2146086 | 377.3933 | 2.7329 | 5 | 2 | 6 | 89.23 |
| CHEMBL1201834 | 253.3207 | 2.09 | 2 | 2 | 4 | 94.9 |
| CHEMBL289351 | 394.3124 | 1.8478 | 6 | 0 | 8 | 119.15 |
| CHEMBL4297534 | 374.439 | 2.6971 | 3 | 3 | 3 | 96.84 |
| CHEMBL1200343 | 368.4678 | 2.4117 | 3 | 0 | 9 | 95.94 |
| CHEMBL1551724 | 258.2313 | -2.7094 | 6 | 5 | 3 | 156.85 |
| CHEMBL2216870 | 415.423 | 3.6993 | 5 | 2 | 5 | 101.38 |
| CHEMBL1090 | 267.2413 | -2.0174 | 7 | 4 | 2 | 139.54 |
| CHEMBL4113131 | 561.4354 | 4.2897 | 5 | 2 | 6 | 122.47 |
| CHEMBL3900409 | 420.4611 | 2.5363 | 5 | 1 | 4 | 109.29 |
| CHEMBL518924 | 349.4726 | 2.8628 | 2 | 1 | 7 | 70.83 |
| CHEMBL36255 | 332.3478 | 3.2158 | 6 | 2 | 6 | 77.38 |
| CHEMBL584 | 567.7824 | 5.2596 | 4 | 4 | 10 | 127.2 |
| CHEMBL4297594 | 253.3207 | 2.09 | 2 | 2 | 4 | 94.9 |
| CHEMBL2347655 | 420.4761 | 4.5218 | 4 | 0 | 6 | 96.46 |
| CHEMBL2403108 | 558.1351 | 6.4825 | 5 | 2 | 9 | 113.62 |
| CHEMBL2108681 | 377.3933 | 2.7329 | 5 | 2 | 6 | 89.23 |
| CHEMBL1201398 | 341.8098 | 2.4268 | 0 | 1 | 4 | 116.64 |
| CHEMBL3183184 | 290.3972 | 5.0467 | 3 | 0 | 10 | 35.53 |
| CHEMBL255066 | 395.8771 | 4.294 | 5 | 0 | 10 | 68.98 |
| CHEMBL542 | 137.136 | 0.6878 | 0 | 1 | 1 | 63.32 |
| CHEMBL3989794 | 534.6432 | 5.0417 | 4 | 1 | 12 | 113.01 |
| CHEMBL4218271 | 287.1205 | -1.5907 | 3 | 2 | 6 | 150.11 |
| CHEMBL3545097 | 309.3259 | 1.416 | 5 | 2 | 2 | 121.67 |
| CHEMBL1944698 | 372.4629 | 4.7341 | 3 | 1 | 0 | 50.28 |
| CHEMBL3545215 | 432.5214 | 2.9984 | 4 | 1 | 6 | 85.8 |
| CHEMBL107360 | 304.4287 | 4.723 | 0 | 0 | 1 | 6.48 |
| CHEMBL3137305 | 510.4927 | 1.7795 | 7 | 3 | 9 | 150.48 |
| CHEMBL2105695 | 574.9819 | 5.2389 | 6 | 1 | 11 | 131.17 |
| CHEMBL348475 | 646.7315 | 5.5731 | 7 | 2 | 11 | 111.25 |
| CHEMBL2163631 | 377.3933 | 2.7329 | 5 | 2 | 6 | 89.23 |
| CHEMBL2105643 | 396.3283 | 3.2034 | 8 | 0 | 8 | 113.49 |
| CHEMBL1399 | 293.3663 | 2.2812 | 4 | 0 | 5 | 78.29 |
| CHEMBL2419346 | 442.5776 | 3.8095 | 4 | 1 | 8 | 115.76 |
| CHEMBL1201831 | 396.3283 | 3.2034 | 8 | 0 | 8 | 113.49 |
| CHEMBL2104962 | 373.3716 | 2.597 | 2 | 0 | 5 | 70.12 |
| CHEMBL485980 | 469.3856 | 1.1481 | 11 | 1 | 8 | 173.8 |
| CHEMBL4297611 | 389.4869 | 3.746 | 3 | 1 | 5 | 82.7 |
| CHEMBL1201733 | 437.518 | 3.379 | 5 | 2 | 5 | 127.41 |
| CHEMBL3287255 | 366.4584 | 1.252 | 5 | 3 | 8 | 160.69 |
| CHEMBL1076263 | 560.6176 | 2.1233 | 7 | 2 | 5 | 162 |
| CHEMBL2108350 | 287.1205 | -1.5907 | 3 | 2 | 6 | 150.11 |
| CHEMBL3304485 | 1145.049 | 6.2314 | 10 | 6 | 29 | 234.76 |
| CHEMBL1200678 | 704.8555 | 5.481 | 9 | 5 | 18 | 171.22 |
| CHEMBL3545051 | 304.4287 | 4.723 | 0 | 0 | 1 | 6.48 |
| CHEMBL1078178 | 414.4598 | 2.8579 | 5 | 2 | 7 | 103.17 |
| CHEMBL2218919 | 1214.622 | 4.3994 | 12 | 5 | 16 | 278.8 |
| CHEMBL416146 | 358.8419 | 3.4671 | 4 | 0 | 3 | 68.3 |
| CHEMBL4297866 | 487.5287 | 4.143 | 4 | 3 | 7 | 98.41 |
| CHEMBL572881 | 373.4509 | 2.8513 | 3 | 3 | 5 | 78.94 |
| CHEMBL2108163 | 359.4243 | 2.9821 | 3 | 1 | 4 | 64.74 |
| CHEMBL3301576 | 447.5095 | 4.7793 | 5 | 1 | 5 | 107.98 |
| CHEMBL70663 | 329.3984 | 5.5518 | 3 | 2 | 4 | 73.63 |
| CHEMBL3701238 | 418.4485 | 1.8707 | 6 | 1 | 5 | 108.39 |
| CHEMBL118 | 381.3722 | 4.0636 | 3 | 1 | 4 | 86.36 |
| CHEMBL830 | 182.1685 | -0.7667 | 0 | 0 | 5 | 89.34 |
| CHEMBL1233550 | 127.9124 | -0.1036 | 0 | 0 | 0 | 0 |
| CHEMBL481 | 586.678 | 5.4769 | 7 | 1 | 5 | 114.2 |
| CHEMBL4297639 | 505.5456 | 4.1123 | 5 | 3 | 6 | 112.24 |
| CHEMBL279115 | 616.825 | 5.7835 | 8 | 3 | 17 | 130.36 |
| CHEMBL3707320 | 482.6166 | 4.825 | 3 | 1 | 3 | 72.36 |
| CHEMBL3545059 | 551.7583 | 3.2958 | 6 | 3 | 19 | 146.13 |
| CHEMBL1639 | 551.7583 | 3.2958 | 6 | 3 | 19 | 146.13 |
| CHEMBL3989909 | 450.4574 | 2.6563 | 5 | 1 | 6 | 83.48 |
| CHEMBL4778541 | 359.4243 | 2.9821 | 3 | 1 | 4 | 64.74 |
| CHEMBL1779710 | 401.4232 | 1.2304 | 3 | 1 | 7 | 84.66 |
| CHEMBL2022968 | 516.0587 | 4.0266 | 4 | 2 | 8 | 102.08 |
| CHEMBL4650333 | 469.535 | 3.8747 | 6 | 2 | 8 | 98.26 |
| CHEMBL1237 | 405.4879 | 1.4598 | 1 | 0 | 12 | 132.96 |
| CHEMBL2103851 | 447.5095 | 4.7793 | 5 | 1 | 5 | 107.98 |
| CHEMBL101253 | 346.8129 | 4.7078 | 3 | 1 | 4 | 50.7 |
| CHEMBL3301606 | 389.4503 | 2.6521 | 4 | 1 | 4 | 80.12 |
| CHEMBL466246 | 346.3364 | -3.1981 | 6 | 4 | 8 | 187.22 |
| CHEMBL542103 | 421.6181 | 7.2023 | 1 | 2 | 7 | 44.37 |
| CHEMBL1204948 | 253.2527 | 2.763 | 2 | 0 | 3 | 63.33 |
| CHEMBL3786343 | 486.5886 | 5.118 | 4 | 2 | 7 | 110.86 |
| CHEMBL1349 | 324.3357 | -0.64 | 6 | 2 | 8 | 151.14 |
| CHEMBL92401 | 179.219 | 0.1934 | 2 | 2 | 3 | 54.02 |
| CHEMBL1088752 | 383.4591 | 3.6894 | 3 | 2 | 6 | 71.09 |
| CHEMBL496748 | 535.3301 | 1.8303 | 1 | 2 | 14 | 165.06 |
| CHEMBL2106480 | 488.603 | 2.1446 | 6 | 1 | 8 | 121.8 |
| CHEMBL4802192 | 359.4243 | 2.9821 | 3 | 1 | 4 | 64.74 |
| CHEMBL1231160 | 443.5193 | 1.1931 | 4 | 3 | 6 | 140.74 |
| CHEMBL55 | 340.4195 | 2.5952 | 4 | 2 | 10 | 118.2 |
| CHEMBL1615209 | 359.4426 | 2.033 | 4 | 1 | 8 | 96.31 |
| CHEMBL317094 | 405.4449 | 2.0068 | 4 | 0 | 10 | 116.25 |
| CHEMBL2110816 | 293.4458 | 5.3413 | 0 | 0 | 4 | 3.24 |
| CHEMBL535 | 398.4738 | 2.6398 | 2 | 3 | 7 | 77.23 |
| CHEMBL3646221 | 306.7011 | 1.6875 | 3 | 2 | 4 | 99.52 |
| CHEMBL4297222 | 495.0163 | 3.5538 | 4 | 1 | 6 | 83.36 |
| CHEMBL3787344 | 495.0163 | 3.5538 | 4 | 1 | 6 | 83.36 |
| CHEMBL2023898 | 738.875 | 4.7706 | 8 | 4 | 13 | 174.64 |
| CHEMBL3137330 | 404.3442 | 0.9634 | 4 | 1 | 3 | 103.7 |
| CHEMBL2105902 | 370.2471 | 4.1492 | 3 | 0 | 4 | 51.75 |
| CHEMBL254316 | 444.4163 | 0.9177 | 8 | 3 | 6 | 152.24 |
| CHEMBL1737 | 474.5764 | 2.1252 | 6 | 1 | 7 | 121.8 |
| CHEMBL4297188 | 432.5197 | 4.4614 | 3 | 1 | 4 | 95.64 |
| CHEMBL3707222 | 805.9544 | 4.5572 | 10 | 5 | 14 | 238.02 |
| CHEMBL3039558 | 450.5053 | 4.3853 | 5 | 1 | 8 | 72.5 |
| CHEMBL404108 | 293.7206 | 4.3754 | 0 | 1 | 4 | 49.33 |
| CHEMBL2105750 | 799.9746 | 4.4254 | 9 | 2 | 14 | 172.69 |
| CHEMBL3833371 | 545.9071 | 2.5673 | 9 | 3 | 11 | 167.99 |
| CHEMBL418995 | 337.4553 | 4.9748 | 0 | 0 | 8 | 49.33 |
| CHEMBL4650215 | 407.3714 | 1.7202 | 5 | 2 | 5 | 111.71 |
| CHEMBL1138 | 409.4252 | 4.6077 | 3 | 2 | 6 | 60.77 |
| CHEMBL4650989 | 405.468 | 4.0081 | 3 | 1 | 7 | 77.99 |
| CHEMBL282042 | 670.8408 | 3.6358 | 6 | 5 | 13 | 166.75 |
| CHEMBL4163691 | 470.523 | 4.5598 | 5 | 2 | 7 | 95.76 |
| CHEMBL4298158 | 532.5182 | 4.3772 | 6 | 3 | 8 | 134.66 |
| CHEMBL491473 | 450.5053 | 4.3853 | 5 | 1 | 8 | 72.5 |
| CHEMBL1922673 | 636.8228 | 7.8224 | 4 | 0 | 13 | 114.6 |
| CHEMBL1738757 | 553.5868 | 6.0323 | 6 | 3 | 7 | 123.06 |
| CHEMBL1082508 | 362.4185 | 3.4298 | 3 | 2 | 4 | 97.64 |
| CHEMBL3581647 | 491.9925 | 4.5187 | 5 | 2 | 4 | 120.64 |
| CHEMBL4594287 | 477.5554 | 5.3135 | 4 | 2 | 6 | 112.87 |
| CHEMBL1460 | 236.2273 | -0.7108 | 5 | 2 | 2 | 93.03 |
| CHEMBL286738 | 309.3376 | 2.6871 | 3 | 1 | 2 | 107.98 |
| CHEMBL3544986 | 368.4678 | 2.4117 | 3 | 0 | 9 | 95.94 |
| CHEMBL266497 | 236.1992 | 0.7862 | 3 | 2 | 0 | 67.43 |
| CHEMBL4279047 | 414.7366 | 5.1491 | 4 | 2 | 7 | 75.62 |
| CHEMBL1496 | 481.5376 | 2.4017 | 6 | 2 | 10 | 149.3 |
| CHEMBL4297604 | 481.5424 | 4.1875 | 3 | 0 | 8 | 79.04 |
| CHEMBL2105891 | 275.3461 | 2.6723 | 2 | 1 | 2 | 44.81 |
| CHEMBL94 | 275.3461 | 2.6723 | 2 | 1 | 2 | 44.81 |
| CHEMBL1201728 | 263.3752 | 2.7711 | 2 | 2 | 4 | 43.7 |
| CHEMBL1118 | 263.3752 | 2.7711 | 2 | 2 | 4 | 43.7 |
| CHEMBL589583 | 452.5427 | 3.5505 | 3 | 0 | 12 | 95.94 |
| CHEMBL3659477 | 336.3877 | 3.4356 | 3 | 2 | 5 | 70.25 |
| CHEMBL54126 | 223.2915 | 1.0359 | 2 | 0 | 3 | 29.54 |
| CHEMBL3301605 | 348.3754 | -0.889 | 4 | 1 | 4 | 136.66 |
| CHEMBL3353410 | 499.6073 | 4.337 | 4 | 2 | 10 | 87.55 |
| CHEMBL493 | 654.5945 | 4.2646 | 5 | 3 | 5 | 118.21 |
| CHEMBL385517 | 315.41 | 0.2755 | 3 | 1 | 2 | 90.35 |
| CHEMBL4127030 | 516.1581 | -1.2396 | 15 | 3 | 8 | 293.8 |
| CHEMBL2043437 | 374.439 | 2.6971 | 3 | 3 | 3 | 96.84 |
| CHEMBL7568 | 399.9568 | 5.6661 | 2 | 1 | 9 | 37.39 |
| CHEMBL3039525 | 633.6882 | 4.1732 | 5 | 3 | 8 | 119.14 |
| CHEMBL3544964 | 440.858 | 3.7735 | 5 | 2 | 6 | 97.86 |
| CHEMBL1086997 | 219.278 | -0.5754 | 4 | 4 | 4 | 84.16 |
| CHEMBL37744 | 317.2236 | 3.4478 | 1 | 0 | 4 | 16.13 |
| CHEMBL4439321 | 387.4312 | 3.1592 | 4 | 2 | 6 | 109.23 |
| CHEMBL1219 | 359.4426 | 2.3507 | 4 | 1 | 8 | 108.39 |
| CHEMBL2103838 | 456.4172 | 4.794 | 5 | 3 | 6 | 100.27 |
| CHEMBL290960 | 287.2923 | 1.9239 | 4 | 0 | 3 | 114.4 |
| CHEMBL4594382 | 314.3015 | 1.7999 | 4 | 1 | 4 | 83.18 |
| CHEMBL4594381 | 314.3015 | 1.7999 | 4 | 1 | 4 | 83.18 |
| CHEMBL3127326 | 894.1091 | 7.4924 | 8 | 4 | 16 | 178.72 |
| CHEMBL298734 | 638.8216 | 5.765 | 3 | 1 | 3 | 79.53 |
| CHEMBL710 | 372.5441 | 3.0674 | 2 | 2 | 2 | 58.2 |
| CHEMBL1201743 | 315.41 | 0.2755 | 3 | 1 | 2 | 90.35 |
| CHEMBL363392 | 674.9553 | 6.1469 | 4 | 1 | 13 | 78.46 |
| CHEMBL1172928 | 255.7405 | 2.3186 | 2 | 1 | 1 | 41.49 |
| CHEMBL1795071 | 306.365 | 1.7999 | 4 | 1 | 4 | 83.18 |
| CHEMBL11662 | 228.2466 | 1.2898 | 1 | 0 | 4 | 55.12 |
| CHEMBL3544978 | 487.4378 | 2.0082 | 8 | 2 | 1 | 143.31 |
| CHEMBL119 | 369.4176 | 2.787 | 4 | 3 | 6 | 117.54 |
| CHEMBL1642 | 493.6027 | 4.2205 | 4 | 2 | 7 | 86.28 |
| CHEMBL4650225 | 765.4438 | 7.2248 | 5 | 1 | 3 | 99.8 |
| CHEMBL1089 | 136.1943 | 0.8367 | 0 | 2 | 3 | 38.05 |
| CHEMBL3989402 | 328.4072 | -0.1266 | 3 | 3 | 8 | 148.53 |
| CHEMBL3545365 | 510.4645 | 3.8697 | 5 | 3 | 8 | 109.42 |
| CHEMBL4297675 | 520.9991 | 4.4715 | 8 | 0 | 16 | 97.36 |
| CHEMBL1094636 | 320.3883 | 2.1165 | 2 | 1 | 3 | 72.94 |
| CHEMBL4582651 | 533.6005 | 2.9203 | 7 | 3 | 8 | 135.53 |
| CHEMBL126648 | 532.5263 | 4.1353 | 7 | 3 | 10 | 170.11 |
| CHEMBL4298151 | 432.5197 | 4.4614 | 3 | 1 | 4 | 95.64 |
| CHEMBL1259059 | 529.4525 | 2.3027 | 9 | 3 | 11 | 167.99 |
| CHEMBL674 | 284.3514 | 0.4459 | 2 | 1 | 6 | 101.65 |
| CHEMBL1336 | 464.8249 | 5.1206 | 4 | 3 | 6 | 92.35 |
| CHEMBL1683590 | 729.8966 | 0.8283 | 11 | 1 | 4 | 146.39 |
| CHEMBL1683544 | 729.8966 | 0.8283 | 11 | 1 | 4 | 146.39 |
| CHEMBL2105704 | 426.853 | 3.4682 | 5 | 3 | 6 | 115.57 |
| CHEMBL3301602 | 425.504 | 1.9955 | 5 | 1 | 5 | 105.57 |
| CHEMBL3301607 | 425.504 | 1.9955 | 5 | 1 | 5 | 105.57 |
| CHEMBL1201066 | 277.4018 | 3.0221 | 2 | 1 | 5 | 32.7 |
| CHEMBL18786 | 340.4824 | 3.7187 | 1 | 1 | 8 | 57.64 |
| CHEMBL277100 | 417.3811 | 3.8755 | 1 | 0 | 3 | 74.57 |
| CHEMBL3989923 | 420.4528 | 3.2508 | 3 | 2 | 8 | 93.48 |
| CHEMBL1237061 | 420.4528 | 3.2508 | 3 | 2 | 8 | 93.48 |
| CHEMBL129 | 267.2413 | -0.9865 | 4 | 2 | 3 | 96.68 |
| CHEMBL4297694 | 340.4824 | 3.7187 | 1 | 1 | 8 | 57.64 |
| CHEMBL223360 | 375.3989 | 4.7833 | 2 | 4 | 3 | 95.83 |
| CHEMBL14249 | 507.181 | -2.5128 | 16 | 3 | 8 | 308.56 |
| CHEMBL4296719 | 241.0951 | -1.0099 | 3 | 2 | 4 | 84.83 |
| CHEMBL31601 | 630.8383 | 3.0332 | 7 | 5 | 16 | 169.86 |
| CHEMBL20 | 222.2454 | -1.3602 | 5 | 2 | 2 | 151.66 |
| CHEMBL2028663 | 519.5624 | 5.3325 | 5 | 2 | 6 | 147.48 |
| CHEMBL1272 | 452.5857 | 5.2818 | 2 | 1 | 10 | 78.87 |
| CHEMBL2364608 | 425.7491 | 3.8059 | 5 | 1 | 5 | 105.7 |
| CHEMBL514201 | 513.0035 | 3.9814 | 3 | 0 | 5 | 65.86 |
| CHEMBL473417 | 421.2971 | 4.2658 | 4 | 1 | 4 | 84.51 |
| CHEMBL4594250 | 453.5323 | 3.9008 | 4 | 1 | 8 | 94.31 |
| CHEMBL2105712 | 485.9384 | 3.8134 | 5 | 2 | 8 | 88.61 |
| CHEMBL11 | 280.4073 | 4.3879 | 0 | 0 | 4 | 6.48 |
| CHEMBL3301610 | 506.5934 | 4.9145 | 4 | 1 | 7 | 75 |
| CHEMBL3707266 | 434.5373 | 2.7183 | 4 | 1 | 5 | 91.21 |
| CHEMBL3989962 | 427.4106 | 0.6607 | 3 | 4 | 9 | 191.26 |
| CHEMBL734 | 75.0666 | -0.8448 | 2 | 2 | 0 | 49.33 |
| CHEMBL2105697 | 279.3364 | 3.6253 | 3 | 0 | 3 | 38.25 |
| CHEMBL3301570 | 75.0666 | -0.8448 | 2 | 2 | 0 | 49.33 |
| CHEMBL206815 | 414.4964 | 0.9731 | 7 | 3 | 6 | 149.85 |
| CHEMBL1567 | 398.4738 | 2.6398 | 2 | 3 | 7 | 77.23 |
| CHEMBL435381 | 381.8091 | 3.0035 | 4 | 1 | 4 | 95.59 |
| CHEMBL3317856 | 448.5175 | 1.3536 | 5 | 0 | 5 | 94.28 |
| CHEMBL1200659 | 376.4467 | 2.1388 | 3 | 0 | 10 | 95.94 |
| CHEMBL3039519 | 885.064 | 6.8594 | 8 | 4 | 13 | 231.12 |
| CHEMBL3813842 | 382.4197 | 0.4513 | 7 | 1 | 2 | 117.1 |
| CHEMBL2042273 | 379.8578 | 3.8177 | 4 | 0 | 6 | 84.08 |
| CHEMBL3899477 | 635.8232 | 4.1358 | 5 | 4 | 10 | 135.37 |
| CHEMBL4297366 | 427.5365 | 3.0172 | 4 | 3 | 13 | 90.9 |
| CHEMBL4594353 | 420.3387 | 5.3263 | 3 | 2 | 4 | 81.65 |
| CHEMBL1801250 | 394.4023 | 1.1027 | 5 | 2 | 5 | 103.27 |
| CHEMBL3039514 | 882.0171 | 7.0564 | 9 | 4 | 13 | 188.8 |
| CHEMBL1487 | 558.6398 | 5.4402 | 3 | 3 | 12 | 111.79 |
| CHEMBL2105717 | 501.5057 | 4.3428 | 6 | 2 | 8 | 98.78 |
| CHEMBL2110737 | 259.2988 | -0.4922 | 5 | 3 | 4 | 90.23 |
| CHEMBL3545307 | 552.5309 | 4.4935 | 5 | 2 | 6 | 106.83 |
| CHEMBL4446357 | 471.5213 | 1.4672 | 6 | 2 | 5 | 125.8 |
| CHEMBL593 | 456.5611 | 1.9169 | 4 | 3 | 6 | 118.81 |
| CHEMBL3039598 | 563.6625 | 6.2443 | 5 | 0 | 15 | 120.02 |
| CHEMBL3137331 | 510.4927 | 1.7795 | 7 | 3 | 9 | 150.48 |
| CHEMBL2103875 | 615.3948 | 5.8093 | 4 | 2 | 5 | 107.13 |
| CHEMBL3813873 | 417.8148 | 4.2043 | 3 | 2 | 6 | 66.49 |
| CHEMBL4802163 | 527.6605 | 3.7228 | 4 | 3 | 6 | 104.38 |
| CHEMBL3707372 | 861.0065 | 5.9879 | 11 | 3 | 9 | 203.6 |
| CHEMBL261244 | 530.4272 | 0.4564 | 9 | 2 | 8 | 194.65 |
| CHEMBL1200485 | 464.8249 | 5.1206 | 4 | 3 | 6 | 92.35 |
| CHEMBL38380 | 291.3687 | 0.1886 | 3 | 0 | 2 | 70.68 |
| CHEMBL1289601 | 426.853 | 3.4682 | 5 | 3 | 6 | 115.57 |
| CHEMBL4298167 | 425.504 | 1.9955 | 5 | 1 | 5 | 105.57 |
| CHEMBL3989920 | 420.4528 | 3.2508 | 3 | 2 | 8 | 93.48 |
| CHEMBL2105595 | 308.7649 | 2.7829 | 2 | 1 | 3 | 46.5 |
| CHEMBL320775 | 284.3363 | 2.3134 | 3 | 2 | 2 | 109.96 |
| CHEMBL215645 | 250.3367 | 3.1239 | 2 | 0 | 5 | 32.78 |
| CHEMBL492399 | 279.3364 | 3.6253 | 3 | 0 | 3 | 38.25 |
| CHEMBL79280 | 371.255 | 3.5343 | 3 | 1 | 4 | 63.78 |
| CHEMBL941 | 493.6027 | 4.2205 | 4 | 2 | 7 | 86.28 |
| CHEMBL3707221 | 804.9465 | 3.8794 | 9 | 5 | 14 | 240.85 |
| CHEMBL1199480 | 550.9043 | 11.5862 | 0 | 0 | 25 | 34.58 |
| CHEMBL211471 | 223.2915 | 1.0359 | 2 | 0 | 3 | 29.54 |
| CHEMBL278020 | 223.2915 | 1.0359 | 2 | 0 | 3 | 29.54 |
| CHEMBL2103745 | 315.41 | 0.2755 | 3 | 1 | 2 | 90.35 |
| CHEMBL206031 | 703.8227 | 3.6573 | 11 | 2 | 15 | 191.6 |
| CHEMBL871 | 206.0282 | -0.8018 | 5 | 1 | 2 | 154.91 |
| CHEMBL916 | 440.5966 | 3.4271 | 3 | 1 | 14 | 113.11 |
| CHEMBL997 | 319.2289 | -0.1406 | 5 | 1 | 9 | 158.15 |
| CHEMBL3989861 | 263.3752 | 2.7711 | 2 | 2 | 4 | 43.7 |
| CHEMBL1988530 | 422.5614 | 3.0366 | 6 | 0 | 10 | 127.02 |
| CHEMBL69941 | 386.6535 | 7.3002 | 1 | 0 | 5 | 17.07 |
| CHEMBL1235782 | 520.6549 | 3.7421 | 8 | 2 | 4 | 103.68 |
| CHEMBL1223851 | 328.316 | 3.5514 | 6 | 1 | 4 | 78.13 |
| CHEMBL560724 | 314.2895 | 3.3004 | 6 | 2 | 3 | 89.13 |
| CHEMBL464877 | 262.301 | 0.9488 | 4 | 2 | 0 | 66.76 |
| CHEMBL1801926 | 295.2927 | 1.985 | 3 | 1 | 4 | 88.24 |
| CHEMBL562222 | 368.4263 | 1.1032 | 4 | 1 | 2 | 67.87 |
| CHEMBL1449034 | 368.4263 | 1.1032 | 4 | 1 | 2 | 67.87 |
| CHEMBL454310 | 502.6892 | 4.6802 | 6 | 0 | 13 | 127.02 |
| CHEMBL572434 | 379.535 | 5.5063 | 2 | 1 | 8 | 42.23 |
| CHEMBL399873 | 442.7168 | 6.2586 | 2 | 2 | 1 | 40.46 |
| CHEMBL182992 | 312.3166 | 3.8188 | 5 | 0 | 4 | 57.9 |
| CHEMBL52676 | 298.2901 | 3.5678 | 5 | 1 | 3 | 68.9 |
| CHEMBL96172 | 491.6831 | 4.7083 | 6 | 2 | 2 | 124.96 |
| CHEMBL319648 | 477.6566 | 4.2619 | 6 | 2 | 2 | 124.96 |
| CHEMBL453818 | 489.6673 | 4.2637 | 6 | 2 | 2 | 124.96 |
| CHEMBL511398 | 475.6407 | 3.8468 | 6 | 2 | 2 | 124.96 |
| CHEMBL1580 | 268.2691 | -1.2567 | 6 | 3 | 2 | 112.13 |
| CHEMBL436605 | 520.572 | 3.7305 | 9 | 3 | 10 | 157.33 |
| CHEMBL453642 | 438.5128 | 5.814 | 6 | 3 | 7 | 96.22 |
| CHEMBL388661 | 472.5275 | 4.0606 | 8 | 5 | 8 | 136.68 |
| CHEMBL445279 | 284.2635 | 2.442 | 5 | 1 | 0 | 57.15 |
| CHEMBL380909 | 246.348 | 1.2877 | 1 | 0 | 0 | 23.55 |
| CHEMBL3590537 | 264.3633 | 0.6683 | 2 | 1 | 0 | 43.78 |
| CHEMBL455667 | 456.5281 | 4.7128 | 7 | 4 | 8 | 116.45 |
| CHEMBL377725 | 246.348 | 1.2877 | 1 | 0 | 0 | 23.55 |
| CHEMBL509486 | 426.5021 | 4.7292 | 6 | 4 | 7 | 107.22 |
| CHEMBL454647 | 442.5015 | 4.4618 | 7 | 5 | 7 | 127.45 |
| CHEMBL3590540 | 244.3321 | 2.3176 | 1 | 0 | 0 | 25.24 |
| CHEMBL470762 | 432.5928 | 4.5215 | 5 | 1 | 8 | 68.29 |
| CHEMBL2336751 | 522.5416 | 0.568 | 11 | 6 | 10 | 167.53 |
| CHEMBL253590 | 360.401 | 2.4724 | 6 | 3 | 7 | 88.38 |
| CHEMBL497442 | 416.5503 | 3.5871 | 5 | 1 | 9 | 72.83 |
| CHEMBL511651 | 400.5509 | 5.1331 | 4 | 1 | 7 | 59.06 |
| CHEMBL472640 | 418.5662 | 4.113 | 5 | 2 | 7 | 79.29 |
| CHEMBL37537 | 154.1201 | 0.8996 | 2 | 2 | 1 | 77.76 |
| CHEMBL513197 | 302.451 | 4.8819 | 0 | 0 | 2 | 37.3 |
| CHEMBL195296 | 300.4351 | 4.9868 | 2 | 1 | 1 | 37.3 |
| CHEMBL1834675 | 346.4174 | 2.271 | 5 | 2 | 1 | 83.83 |
| CHEMBL61673 | 222.1941 | 1.4746 | 3 | 1 | 1 | 83.83 |
| CHEMBL1277661 | 302.451 | 4.8767 | 2 | 2 | 1 | 40.46 |
| CHEMBL454320 | 330.2889 | 3.033 | 7 | 3 | 3 | 109.36 |
| CHEMBL234316 | 478.4029 | 0.8776 | 12 | 7 | 5 | 199.51 |
| CHEMBL250450 | 464.3763 | 0.6266 | 12 | 8 | 4 | 210.51 |
| CHEMBL517713 | 475.6407 | 3.8173 | 6 | 2 | 2 | 124.96 |
| CHEMBL511141 | 477.5904 | 2.5872 | 8 | 2 | 2 | 122.39 |
| CHEMBL459816 | 493.656 | 3.43 | 7 | 3 | 2 | 145.19 |
| CHEMBL460241 | 489.6673 | 4.5258 | 6 | 2 | 2 | 124.96 |
| CHEMBL471687 | 475.6175 | 4.1501 | 7 | 2 | 2 | 109.86 |
| CHEMBL1253219 | 253.8762 | 1.1222 | 2 | 0 | 0 | 26.3 |
| CHEMBL3187351 | 148.2895 | 2.8751 | 0 | 0 | 5 | 50.6 |
| CHEMBL170458 | 114.2086 | 2.0288 | 1 | 0 | 4 | 25.3 |
| CHEMBL477 | 267.2413 | -2.0174 | 7 | 4 | 2 | 139.54 |
| CHEMBL18602 | 86.1323 | 1.3968 | 1 | 0 | 3 | 17.07 |
| CHEMBL1080997 | 152.2334 | 3.0837 | 1 | 0 | 4 | 17.07 |
| CHEMBL366603 | 146.2736 | 2.6194 | 0 | 0 | 5 | 50.6 |
| CHEMBL1347061 | 94.199 | 1.3858 | 0 | 0 | 1 | 50.6 |
| CHEMBL123040 | 178.3386 | 3.21 | 0 | 0 | 6 | 75.9 |
| CHEMBL25719 | 154.2493 | 2.9341 | 1 | 1 | 4 | 20.23 |
| CHEMBL234926 | 58.0791 | 0.2588 | 1 | 1 | 1 | 20.23 |
| CHEMBL468037 | 142.1094 | 0.2127 | 2 | 1 | 2 | 70.67 |
| CHEMBL2105350 | 268.4778 | 6.9129 | 1 | 1 | 15 | 20.23 |
| CHEMBL150 | 286.2363 | 2.7984 | 6 | 4 | 1 | 111.13 |
| CHEMBL521394 | 396.3469 | 3.0662 | 8 | 2 | 3 | 103.68 |
| CHEMBL1778157 | 442.4154 | 1.8958 | 9 | 1 | 5 | 109.75 |
| CHEMBL1950045 | 154.1632 | 1.0119 | 3 | 3 | 2 | 60.69 |
| CHEMBL28941 | 400.3787 | 1.8607 | 8 | 2 | 3 | 103.68 |
| CHEMBL95972 | 400.3787 | 2.6896 | 8 | 2 | 3 | 103.68 |
| CHEMBL487214 | 410.3735 | 3.3172 | 8 | 1 | 4 | 92.68 |
| CHEMBL329250 | 402.3946 | 1.8088 | 8 | 3 | 4 | 114.68 |
| CHEMBL82303 | 412.3894 | 2.4944 | 8 | 0 | 4 | 89.52 |
| CHEMBL61 | 414.4053 | 2.1117 | 8 | 1 | 4 | 92.68 |
| CHEMBL1778160 | 412.3894 | 2.4944 | 8 | 0 | 4 | 89.52 |
| CHEMBL53566 | 138.1638 | 1.2793 | 2 | 2 | 2 | 40.46 |
| CHEMBL283120 | 414.4053 | 2.1117 | 8 | 1 | 4 | 92.68 |
| CHEMBL99342 | 388.368 | 1.5578 | 8 | 4 | 3 | 125.68 |
| CHEMBL63970 | 398.4059 | 3.208 | 7 | 0 | 4 | 72.45 |
| CHEMBL3349967 | 400.3787 | 1.8607 | 8 | 2 | 3 | 103.68 |
| CHEMBL458847 | 314.2895 | 2.4256 | 6 | 1 | 1 | 66.38 |
| CHEMBL2407387 | 348.4333 | 1.095 | 5 | 3 | 0 | 86.99 |
| CHEMBL521396 | 448.5061 | 0.7625 | 8 | 2 | 5 | 119.36 |
| CHEMBL1414 | 198.1727 | 1.1342 | 3 | 1 | 3 | 75.99 |
| CHEMBL583912 | 290.2681 | 1.9202 | 6 | 5 | 1 | 110.38 |
| CHEMBL253582 | 342.2965 | -4.3105 | 11 | 8 | 5 | 189.53 |
| CHEMBL362378 | 322.3545 | 3.8479 | 4 | 2 | 3 | 66.76 |
| CHEMBL451168 | 272.2528 | 2.2977 | 5 | 3 | 1 | 86.99 |
| CHEMBL254051 | 272.2528 | 1.9129 | 5 | 3 | 1 | 86.99 |
| CHEMBL311498 | 290.2681 | 1.9202 | 6 | 5 | 1 | 110.38 |
| CHEMBL66 | 304.2516 | 1.3781 | 7 | 5 | 1 | 127.45 |
| CHEMBL126121 | 300.2629 | 3.0494 | 6 | 3 | 2 | 100.13 |
| CHEMBL125743 | 306.2675 | 1.6528 | 7 | 6 | 1 | 130.61 |
| CHEMBL582103 | 254.2375 | 1.7935 | 4 | 1 | 1 | 63.6 |
| CHEMBL1923074 | 346.3298 | -0.9791 | 9 | 5 | 6 | 138.07 |
| CHEMBL575808 | 342.3426 | 3.8024 | 6 | 0 | 5 | 67.13 |
| CHEMBL31574 | 286.2363 | 2.7984 | 6 | 4 | 1 | 111.13 |
| CHEMBL460618 | 308.371 | 4.9171 | 3 | 2 | 5 | 57.53 |
| CHEMBL170405 | 302.2357 | 2.531 | 7 | 5 | 1 | 131.36 |
| CHEMBL522251 | 484.3644 | -0.031 | 14 | 9 | 7 | 243.9 |
| CHEMBL445740 | 204.3511 | 4.7533 | 0 | 0 | 0 | 0 |

| **Supplementary table 2.** **Pharmacokinetic properties of the selected drug-like molecules.** | | | | | | | | | | | | | | | | | | | | | | | | | | | | | | | | | | |
| --- | --- | --- | --- | --- | --- | --- | --- | --- | --- | --- | --- | --- | --- | --- | --- | --- | --- | --- | --- | --- | --- | --- | --- | --- | --- | --- | --- | --- | --- | --- | --- | --- | --- | --- |
| ChEMBL | **SMILES** | MOL_WEIGHT | LOGP | #ROTATABLE_BONDS | #ACCEPTORS | #DONORS | SURFACE_AREA | Water solubility | Caco2 permeability | Intestinal absorption (human) | Skin Permeability | P-gp substrate | P gp-I inhibitor | P gp-II inhibitor | VDss (human) | Fraction unbound (human) | BBB permeability | CNS permeability | CYP2D6 substrate | CYP3A4 substrate | CYP1A2 inhibitior | CYP2C19 inhibitior | CYP2C9 inhibitior | CYP2D6 inhibitior | CYP2C3A4 inhibitior | Total Clearance | Renal OCT2 substrate | AMES toxicity | Max. tolerated dose (human) | hERG I inhibitor | hERG II inhibitor | Oral Rat Acute Toxicity (LD50) | Oral Rat Chronic Toxicity (LOAEL) | Hepatotoxicity |
| CHEMBL2106793 | COCCNC(=O)c1ccnc(C(=O)NCCOC)c1 | 281.312 | -0.166 | 8 | 5 | 2 | 117.22 | -2.722 | 0.99 | 82.896 | -3.251 | No | No | No | -0.514 | 0.584 | -0.706 | -3.22 | No | No | No | No | No | No | No | 0.977 | No | No | 0.927 | No | No | 2.211 | 1.485 | Yes |
| CHEMBL235641 | O=C1Nc2ccc3ncsc3c2/C1=C/c1c[nH]cn1 | 268.301 | 2.5121 | 1 | 4 | 2 | 111.57 | -2.864 | 0.798 | 85.088 | -2.735 | Yes | No | No | 0.728 | 0.018 | -0.901 | -2.278 | No | Yes | Yes | No | No | Yes | No | 1.276 | Yes | Yes | 0.179 | No | Yes | 2.703 | 2.309 | Yes |
| CHEMBL2105703 | Cc1ccc2c(c1)c1c(n2CCc2ccc(C)nc2)CCN(C)C1.Cl.Cl | 392.374 | 4.72734 | 3 | 3 | 0 | 167.789 | -4.102 | 1.035 | 93.061 | -2.613 | Yes | Yes | Yes | 1.47 | 0.228 | 0.715 | -1.855 | Yes | Yes | Yes | No | No | Yes | Yes | 1.466 | No | Yes | -0.389 | No | Yes | 2.704 | 0.912 | No |
| CHEMBL1317546 | CC(=O)c1ccc(N2CCOCC2)cc1O | 221.256 | 1.4314 | 2 | 4 | 1 | 94.444 | -2.613 | 1.193 | 96.466 | -3.09 | No | No | No | 0.19 | 0.479 | -0.159 | -2.886 | No | No | Yes | No | No | No | No | 0.467 | No | No | 0.189 | No | No | 1.858 | 1.471 | No |
| CHEMBL2216863 | Cc1csc2nc([C@H](C)Nc3ncnc4[nH]cnc34)c(-c3cccc(F)c3)c(=O)n12 | 421.461 | 3.70992 | 4 | 8 | 2 | 173.056 | -2.895 | 1.077 | 88.546 | -2.735 | Yes | No | No | -0.211 | 0.352 | -1.876 | -3.827 | No | No | No | No | Yes | No | Yes | 0.586 | Yes | Yes | 0.472 | No | Yes | 2.513 | 2.443 | Yes |
| CHEMBL2105508 | CCOc1nsnc1NS(=O)(=O)c1ccc(N)cc1 | 300.365 | 1.3198 | 5 | 7 | 2 | 114.344 | -2.537 | 0.745 | 74.667 | -2.744 | Yes | No | No | -0.09 | 0.264 | -0.403 | -3.169 | No | No | No | No | No | No | No | 0.065 | No | No | 0.8 | No | No | 1.964 | 1.764 | Yes |
| CHEMBL9440 | CN(Cc1ccc(S(=O)(=O)N2CCOCC2)cc1)c1ccc2c3c(cccc13)C(N)=N2 | 436.537 | 2.8475 | 5 | 6 | 1 | 181.342 | -4.472 | 1.172 | 97.05 | -2.749 | Yes | Yes | Yes | 0.067 | 0.007 | -0.759 | -2.411 | No | Yes | Yes | Yes | Yes | No | No | 0.744 | No | Yes | -0.3 | No | Yes | 2.124 | 1.74 | Yes |
| CHEMBL3694252 | CCCCCC1CCCc2c1nc1ccccc1c2O | 269.388 | 4.9406 | 4 | 2 | 1 | 120.406 | -4.565 | 1.64 | 94.22 | -2.748 | Yes | No | No | 0.795 | 0 | 0.232 | -1.559 | No | Yes | Yes | Yes | Yes | No | No | 0.959 | No | No | 0.35 | No | Yes | 2.145 | 2.352 | Yes |
| CHEMBL4650521 | C[C@@H]1OCC2(CCN(c3cnc(Sc4ccnc(N)c4Cl)c(N)n3)CC2)[C@@H]1N | 421.958 | 2.1732 | 3 | 9 | 3 | 172.74 | -3.434 | 0.41 | 85.716 | -2.741 | Yes | No | No | 0.908 | 0.51 | -1.126 | -3.199 | No | No | No | No | No | No | No | 0.642 | No | No | 0.128 | No | Yes | 2.664 | 1.227 | Yes |
| CHEMBL3989670 | O.c1cnc2c(c1)C(Cc1ccncc1)(Cc1ccncc1)c1cccnc1-2 | 368.44 | 3.1938 | 4 | 4 | 0 | 163.051 | -3.642 | 0.938 | 100 | -2.703 | No | No | Yes | 0.512 | 0.376 | -0.558 | -2.414 | No | Yes | Yes | Yes | Yes | No | Yes | 0.318 | No | No | -0.075 | No | Yes | 2.377 | 0.533 | Yes |
| CHEMBL459178 | COc1cc(-c2ccccc2)cc([C@H](C)C#Cc2c(C)nc(N)nc2N)c1 | 358.445 | 3.78022 | 3 | 5 | 2 | 158.967 | -4.474 | 0.997 | 95.754 | -2.758 | Yes | Yes | Yes | -0.424 | 0 | 0.093 | -2.075 | No | Yes | Yes | Yes | Yes | No | Yes | 0.161 | No | Yes | -0.154 | No | Yes | 2.742 | 2.64 | Yes |
| CHEMBL4650223 | Cc1ccc(C(=O)Oc2ccc(C(CN(C)C)C3(O)CCCCC3)cc2)cc1.Cl.O.O | 454.007 | 3.32702 | 6 | 4 | 1 | 190.806 | -4.172 | 0.878 | 81.721 | -2.923 | Yes | Yes | Yes | 0.46 | 0.075 | -0.612 | -2.479 | No | Yes | No | No | No | No | Yes | 0.994 | No | No | -0.168 | No | Yes | 2.766 | 1.484 | Yes |
| CHEMBL144620 | NP(N)(=O)NC(=O)c1ccc(F)cc1 | 217.14 | 0.581 | 2 | 2 | 3 | 79.811 | -2.141 | -0.073 | 68.918 | -3.258 | Yes | No | No | -0.924 | 0.39 | -0.982 | -3.433 | No | No | No | No | No | No | No | -0.336 | No | No | 1.137 | No | No | 2.34 | 1.635 | No |
| CHEMBL228792 | c1coc(CNc2ncnc3[nH]cnc23)c1 | 215.216 | 1.558 | 3 | 5 | 2 | 90.747 | -2.586 | 1.202 | 96.169 | -2.735 | Yes | No | No | -0.265 | 0.562 | -1.246 | -3.864 | No | No | Yes | No | No | No | No | 1.054 | No | Yes | 0.886 | No | No | 2.376 | 1.042 | Yes |
| CHEMBL2110630 | COc1cccc(OC(C)CNC(=N)Cc2cccc(C)c2)c1 | 312.413 | 3.58049 | 7 | 3 | 2 | 137.505 | -4.113 | 1.476 | 91.386 | -2.831 | Yes | Yes | No | 0.07 | 0.077 | -0.031 | -2.25 | Yes | No | Yes | Yes | Yes | No | No | 0.556 | No | Yes | 0.037 | No | Yes | 2.632 | 1.18 | Yes |
| CHEMBL29835 | CCCN(c1ccncc1)n1ccc2ccccc21 | 251.333 | 3.716 | 4 | 3 | 0 | 112.634 | -3.528 | 1.692 | 97.269 | -2.255 | Yes | No | No | 0.381 | 0.262 | 0.32 | -1.69 | No | Yes | Yes | Yes | No | No | No | 0.469 | No | Yes | -0.329 | No | No | 2.098 | 0.48 | Yes |
| CHEMBL3639433 | N[C@H]1C[C@@H](N2Cc3cnc(C(F)(F)F)nc3C2)CSC1c1cc(F)ccc1F | 416.419 | 3.6633 | 2 | 5 | 1 | 161.886 | -4.662 | 1.356 | 88.954 | -3.347 | Yes | Yes | No | 0.855 | 0.265 | 0.08 | -2.058 | No | Yes | No | No | No | No | Yes | 0.435 | No | No | -0.698 | No | Yes | 3.148 | 1.537 | Yes |
| CHEMBL2170601 | O=C(O)c1ccc(Nc2nccc(Nc3ccccc3Cl)n2)cc1 | 340.77 | 4.3154 | 5 | 5 | 3 | 142.709 | -3.494 | 0.76 | 89.38 | -2.728 | Yes | No | No | -0.225 | 0.148 | -1.293 | -2.169 | No | No | Yes | No | No | No | No | -0.035 | No | No | 0.835 | No | No | 2.218 | 1.3 | No |
| CHEMBL257590 | O=S(=O)(c1ccccc1)N1CCN(c2ncccc2C(F)(F)F)CC1 | 371.384 | 2.6113 | 3 | 4 | 0 | 143.304 | -4.59 | 1.394 | 95.855 | -2.947 | No | No | No | -0.503 | 0.109 | 0.019 | -2.908 | No | Yes | Yes | Yes | Yes | No | No | 0.488 | No | No | -0.126 | No | No | 2.675 | 1.42 | Yes |
| CHEMBL114586 | N=C(N)c1ccc2cc(OC(=O)c3ccc(NC4=NCCN4)cc3)ccc2c1 | 373.416 | 2.71417 | 4 | 6 | 4 | 161.284 | -3.197 | 0.819 | 80.217 | -2.735 | Yes | Yes | Yes | 1.327 | 0.066 | -0.522 | -2.375 | No | Yes | Yes | Yes | No | No | No | 0.72 | No | No | -0.262 | No | Yes | 2.57 | 2.511 | Yes |
| CHEMBL64706 | CCOC(=O)CCNC(=O)N[C@H]1CCN(c2ccc(C(=N)N)cc2)C1=O | 361.402 | 0.32837 | 7 | 5 | 4 | 150.923 | -3.63 | 0.191 | 58.737 | -3.074 | Yes | No | No | -0.535 | 0.496 | -1.106 | -3.549 | No | No | No | No | No | No | No | 0.696 | No | No | 0.588 | No | No | 2.343 | 0.941 | Yes |
| CHEMBL2104184 | CO/N=C/[C@@H]1[C@@H](c2ccc(Cl)c(Cl)c2)C[C@@H]2CC[C@H]1N2C | 327.255 | 4.1919 | 3 | 3 | 0 | 135.398 | -3.413 | 1.54 | 90.528 | -2.416 | No | No | No | 1.143 | 0.42 | 0.44 | -2.649 | No | Yes | Yes | No | No | Yes | No | 0.532 | No | No | -0.071 | No | No | 3.372 | 0.988 | No |
| CHEMBL179583 | NC(=O)c1ccc2[nH]c(-c3ccc(Oc4ccc(Cl)cc4)cc3)nc2c1 | 363.804 | 4.7745 | 4 | 3 | 2 | 153.939 | -2.909 | 1.012 | 80.068 | -2.735 | Yes | Yes | Yes | 0.105 | 0.232 | -0.703 | -1.929 | No | Yes | Yes | Yes | Yes | No | Yes | 0.8 | Yes | Yes | 0.121 | No | Yes | 2.286 | 1.483 | Yes |
| CHEMBL23811 | COC(=O)c1nc2cc(Cl)c3cccnc3c2o1 | 262.652 | 2.816 | 1 | 5 | 0 | 106.337 | -3.142 | 1.292 | 97.873 | -2.717 | No | No | No | -0.39 | 0.218 | 0.35 | -2.984 | No | No | Yes | Yes | No | No | No | 0.322 | No | Yes | 0.419 | No | No | 2.628 | 0.757 | Yes |
| CHEMBL3979920 | Cn1cc(-c2nc(N[C@@H]3CCCC[C@@H]3N)c(F)c3c2C(=O)NC3)cn1 | 344.394 | 1.5464 | 3 | 6 | 3 | 144.355 | -3.067 | 0.583 | 76.556 | -2.845 | Yes | No | No | 0.963 | 0.522 | -1.022 | -3.08 | No | No | No | No | No | No | No | 0.848 | No | No | -0.261 | No | No | 2.434 | 1.551 | Yes |
| CHEMBL301958 | OC1CCCc2nc3ccccc3c(NCc3ccccc3)c21 | 304.393 | 4.2166 | 3 | 3 | 2 | 135.553 | -4.435 | 1.361 | 89.659 | -2.807 | Yes | No | Yes | 0.108 | 0 | 0.448 | -1.511 | No | Yes | Yes | Yes | Yes | No | Yes | 0.272 | No | No | -0.058 | No | Yes | 2.213 | 1.201 | Yes |
| CHEMBL3651711 | COc1cc(-n2ccc(-c3ccc(C(F)(F)F)cc3)cc2=O)ccc1OCC(C)(C)O | 433.426 | 4.6816 | 6 | 5 | 1 | 176.844 | -5.438 | 1.268 | 95.773 | -2.677 | Yes | Yes | Yes | 0.294 | 0.205 | -0.248 | -2.078 | No | Yes | Yes | Yes | Yes | No | Yes | 0.135 | No | No | 0.274 | No | No | 2.445 | 1.454 | Yes |
| CHEMBL3544920 | CON(C)C(=O)N1N=C(c2cc(F)ccc2F)S[C@@]1(CCCN)c1ccccc1.Cl | 456.946 | 4.3021 | 6 | 5 | 1 | 183.964 | -5.655 | 0.697 | 91.948 | -2.861 | No | Yes | Yes | 0.454 | 0.075 | -1.041 | -2.277 | No | Yes | No | No | No | Yes | Yes | 0.26 | Yes | No | -0.274 | No | Yes | 2.822 | 0.83 | Yes |
| CHEMBL2110886 | CCCCC[C@H](O)/C=C/[C@H]1[C@H](O)C[C@]2(C)C/C(=C\CCCC(=O)O)C[C@H]12 | 364.526 | 4.4622 | 10 | 3 | 3 | 157.556 | -3.357 | 0.751 | 94.839 | -2.734 | No | No | Yes | -0.972 | 0.137 | -0.735 | -2.407 | No | No | No | No | No | No | No | 1.367 | No | No | -1.786 | No | No | 3.343 | 2.297 | No |
| CHEMBL2104330 | CN(CC(=O)NO)C(=O)[C@@H]1CCCC[C@@H]1C(=O)O | 258.274 | -0.1588 | 4 | 4 | 3 | 104.72 | -2.022 | -0.227 | 32.249 | -2.735 | Yes | No | No | -1.711 | 0.571 | -0.846 | -3.422 | No | No | No | No | No | No | No | 0.346 | No | No | 0.586 | No | No | 1.958 | 2.685 | No |
| CHEMBL2104528 | CCCCCCC(C)NN | 144.262 | 1.8086 | 6 | 2 | 2 | 64.154 | -2.208 | 1.308 | 91.644 | -2.222 | No | No | No | 0.011 | 0.538 | 0.073 | -2.546 | No | No | No | No | No | No | No | 1.764 | No | No | 0.87 | No | No | 2.282 | 1.516 | Yes |
| CHEMBL2104720 | CCCCCC(/C=C/n1ccnc1)OC(=O)c1cccnc1 | 299.374 | 3.5547 | 8 | 5 | 0 | 130.217 | -2.646 | 1.504 | 95.507 | -2.829 | No | No | No | -0.285 | 0.422 | -0.19 | -2.86 | No | No | Yes | No | Yes | No | Yes | 0.981 | No | Yes | 0.436 | No | No | 2.57 | 1.153 | Yes |
| CHEMBL4297220 | Cl.O=C(Nc1ccc(OC(F)(F)Cl)cc1)c1cnc(N2CC[C@@H](O)C2)c(-c2cc[nH]n2)c1 | 486.306 | 3.8847 | 6 | 6 | 3 | 192.513 | -3.878 | 0.548 | 88.87 | -2.75 | Yes | Yes | Yes | -0.078 | 0.106 | -1.795 | -2.803 | No | Yes | No | Yes | Yes | No | Yes | 0 | No | Yes | 0.06 | No | Yes | 2.896 | 1.77 | Yes |
| CHEMBL4297193 | CC(C)N1CCN(Cc2cnc(-c3cc(-c4cccc5[nH]ccc45)cc4[nH]ncc34)o2)CC1.CC(C)N1CCN(Cc2cnc(-c3cc(-c4cccc5[nH]ccc45)cc4[nH]ncc34)o2)CC1.O=C(O)CCC(=O)O | 999.19 | 9.7202 | 13 | 12 | 6 | 426.934 | -2.892 | -1.353 | 51.741 | -2.735 | Yes | No | Yes | -0.091 | 0.378 | -2.874 | -3.357 | Yes | Yes | No | No | No | No | No | -0.034 | No | No | 0.438 | No | No | 2.482 | 3.267 | Yes |
| CHEMBL2107322 | CNCCCOc1cc(F)c(-c2c(Cl)nc3ncnn3c2N[C@@H](C)C(F)(F)F)c(F)c1.O.O.O=C(O)/C=C/C(=O)O | 616.928 | 2.1363 | 10 | 9 | 4 | 232.782 | -3.076 | -0.305 | 20.745 | -2.735 | Yes | No | No | -0.128 | 0.161 | -2.427 | -3.573 | No | No | No | No | No | No | No | -0.672 | No | No | 0.863 | No | No | 2.266 | 1.372 | Yes |
| CHEMBL2105739 | CNS(=O)(=O)C[C@H]1CC[C@H](N(C)c2[nH]cnc3nccc2-3)CC1.O=C(O)/C=C\C(=O)O | 453.521 | 1.1655 | 7 | 7 | 4 | 180.153 | -2.598 | -0.547 | 15.554 | -2.735 | No | No | No | -1.091 | 0.487 | -1.813 | -3.763 | No | No | No | No | No | No | No | 0.799 | No | No | 0.844 | No | No | 1.892 | 1.995 | Yes |
| CHEMBL332003 | COc1cc2c3c(n(CCCNCCO)c(=O)c2cc1OC)-c1cc2c(cc1C3=O)OCO2.Cl | 488.924 | 2.3526 | 8 | 9 | 2 | 201.147 | -4.437 | 0.712 | 100 | -2.756 | Yes | Yes | Yes | 0.505 | 0.166 | -1.212 | -3.613 | No | Yes | No | No | No | No | Yes | 1.448 | Yes | No | 0.285 | No | Yes | 2.518 | 1.831 | No |
| CHEMBL2103874 | CNS(=O)(=O)C[C@H]1CC[C@H](N(C)c2ncnc3[nH]ccc23)CC1 | 337.449 | 1.5021 | 5 | 5 | 2 | 136.111 | -2.719 | 1.268 | 92.035 | -3.255 | No | No | No | 0.01 | 0.454 | -1.127 | -3.544 | No | No | No | No | No | No | No | 0.675 | No | No | -0.378 | No | No | 2.424 | 0.419 | Yes |
| CHEMBL2111132 | c1ccc2c(c1)cc(-c1nnn[nH]1)c1nnnn12 | 238.214 | 0.4576 | 1 | 7 | 1 | 99.626 | -2.66 | 0.74 | 68.76 | -2.735 | No | No | No | 0.254 | 0.199 | -1.505 | -3.935 | No | No | Yes | No | No | No | No | 0.296 | No | No | 0.466 | No | No | 2.363 | 3.362 | Yes |
| CHEMBL2170592 | O=C(O)c1ccc(Nc2nccc(Nc3ccccc3C(=O)O)n2)cc1 | 350.334 | 3.3602 | 6 | 6 | 4 | 147.726 | -3.14 | 0.575 | 45.611 | -2.735 | Yes | No | No | -1.494 | 0.134 | -1.432 | -3.288 | No | No | No | No | No | No | No | 0.001 | No | No | 1.28 | No | No | 2.531 | 2.851 | Yes |
| CHEMBL2107323 | CNCCCOc1cc(F)c(-c2c(Cl)nc3ncnn3c2N[C@@H](C)C(F)(F)F)c(F)c1.O.O.O=C(O)CCC(=O)O | 618.944 | 2.3603 | 11 | 9 | 4 | 233.471 | -3.076 | -0.318 | 21.523 | -2.735 | Yes | No | No | -0.101 | 0.162 | -2.488 | -3.586 | No | No | No | No | No | No | No | -0.411 | No | No | 0.86 | No | No | 2.258 | 1.351 | Yes |
| CHEMBL1788400 | O=C(C(Cl)Cl)N(CCO)Cc1ccc(Oc2ccc([N+](=O)[O-])cc2)cc1 | 399.23 | 3.5117 | 8 | 5 | 1 | 159.402 | -5.568 | 1.06 | 83.235 | -2.727 | Yes | Yes | Yes | 0.002 | 0 | -0.849 | -2.622 | No | Yes | Yes | Yes | Yes | No | Yes | 0.24 | No | Yes | -0.27 | No | No | 2.18 | 2.177 | Yes |
| CHEMBL63055 | O=C(O)CN1C(=O)c2cccc3cccc(c23)C1=O | 255.229 | 1.5204 | 2 | 3 | 1 | 107.606 | -3.007 | -0.275 | 94.918 | -2.755 | Yes | No | No | -0.855 | 0.122 | -0.001 | -2.532 | No | Yes | No | No | No | No | No | 0.206 | No | No | -0.033 | No | No | 2.076 | 1.229 | No |
| CHEMBL2107005 | COc1cccc2c1O[C@H]1[C@@H](C2)C(=O)[C@H]2Cc3cccc(OC)c3O[C@H]2C1(C)C | 394.467 | 3.8523 | 2 | 5 | 0 | 170.466 | -4.985 | 1.146 | 100 | -2.772 | No | Yes | Yes | -0.098 | 0 | -0.479 | -1.815 | No | Yes | Yes | Yes | Yes | No | Yes | -0.08 | No | No | 0.353 | No | No | 2.594 | 1.789 | No |
| CHEMBL76688 | CC(C)(C)C(=O)Oc1ccc(S(=O)(=O)Nc2ccccc2C(=O)NCC(=O)O)cc1 | 434.47 | 2.2533 | 7 | 6 | 3 | 173.661 | -4.069 | -0.212 | 47.586 | -2.735 | Yes | No | No | -1.19 | 0 | -1.151 | -3.5 | No | No | No | No | No | No | No | 0.187 | No | No | -0.101 | No | No | 2.227 | 2.687 | Yes |
| CHEMBL2106980 | c1ccc(-c2nc(N3CCNCC3)cc3ccccc23)cc1 | 289.382 | 3.3114 | 2 | 3 | 1 | 130.153 | -3.682 | 0.942 | 95.605 | -2.554 | Yes | No | Yes | 1.049 | 0.197 | 0.418 | -1.465 | No | Yes | Yes | No | No | Yes | Yes | 0.705 | Yes | Yes | 0.122 | No | Yes | 2.634 | 0.473 | Yes |
| CHEMBL2170804 | CS(=O)(=O)O.OCCn1cc(-c2cnc3nnn(Cc4ccc5ncccc5c4)c3n2)cn1 | 468.499 | 1.1776 | 5 | 11 | 2 | 187.859 | -2.861 | -0.027 | 39.385 | -2.735 | No | No | No | -0.303 | 0.233 | -1.562 | -3.66 | No | Yes | No | No | No | No | No | 0.496 | No | No | 0.615 | No | No | 2.286 | 2.794 | Yes |
| CHEMBL3040926 | CC(=N)NCCSC[C@](C)(N)C(=O)O | 219.31 | 0.10837 | 6 | 4 | 4 | 87.948 | -0.875 | -0.396 | 38.851 | -2.735 | No | No | No | -0.746 | 0.795 | -1 | -3.571 | No | No | No | No | No | No | No | 0.495 | No | No | 1.566 | No | No | 1.824 | 1.638 | No |
| CHEMBL1788390 | CCOC(=O)/C(=C/c1ncc([N+](=O)[O-])n1C)C(C)=O | 267.241 | 0.8638 | 5 | 7 | 0 | 108.39 | -2.927 | 0.276 | 80.302 | -2.735 | Yes | No | No | -0.724 | 0.527 | -0.979 | -3.038 | No | No | No | No | No | No | No | 0.362 | No | Yes | 0.34 | No | No | 1.987 | 1.583 | Yes |
| CHEMBL2104499 | CCOC(=O)[C@H](CCc1ccccc1)N[C@H]1CS[C@H](c2cccs2)CN(CC(=O)O)C1=O.Cl | 513.081 | 3.3937 | 10 | 7 | 2 | 207.846 | -3.178 | -0.068 | 50.49 | -2.735 | Yes | No | No | -0.208 | 0.442 | -1.096 | -2.901 | No | Yes | No | No | No | No | No | 0.903 | No | No | -0.259 | No | No | 2.878 | 2.163 | Yes |
| CHEMBL489079 | NC/C(=C/F)CCc1ccc(F)cc1 | 197.228 | 2.5704 | 4 | 1 | 1 | 82.237 | -2.239 | 1.241 | 93.904 | -2.305 | No | No | No | 0.613 | 0.361 | 0.33 | -2.03 | No | No | Yes | No | No | Yes | No | 0.866 | No | No | 0.952 | No | No | 2.03 | 2.064 | Yes |
| CHEMBL497011 | Cc1cccc(C)c1CNc1cc(C(=O)NCCO)cn2c(C)c(C)nc12 | 366.465 | 2.90218 | 6 | 5 | 3 | 158.801 | -2.534 | 0.925 | 93.303 | -2.735 | Yes | No | No | 0.87 | 0.368 | -0.748 | -2.952 | No | Yes | Yes | No | No | Yes | No | 1.087 | Yes | Yes | 0.339 | No | Yes | 2.775 | 1.257 | No |
| CHEMBL4594279 | CS(=O)(=O)O.O=C(Cc1ccc(-c2ccc(OCCN3CCOCC3)cc2)cn1)NCc1ccccc1 | 527.643 | 2.8225 | 9 | 7 | 2 | 217.742 | -3.343 | 0.31 | 47.007 | -2.735 | Yes | No | Yes | 0.526 | 0.197 | -1.199 | -3.26 | No | Yes | No | No | No | No | No | 0.782 | No | No | 0.28 | No | No | 2.674 | 2.253 | Yes |
| CHEMBL2218915 | CCOC(=O)CC(O)CC(O)/C=C/C1=C(c2ccc(F)cc2)c2ccccc2OC12CCCC2 | 466.549 | 4.9541 | 8 | 5 | 2 | 199.079 | -5.565 | 0.737 | 96.825 | -2.699 | Yes | Yes | Yes | -0.261 | 0.108 | 0.097 | -2.721 | No | Yes | No | Yes | No | No | Yes | 0.769 | No | No | 0.038 | No | Yes | 4.22 | 0.996 | Yes |
| CHEMBL1201405 | COc1cc2c(cc1OC)CN(C(=O)[C@H](C)N[C@@H](CCc1ccccc1)C(=O)O)[C@H](C(=O)O)C2 | 470.522 | 2.1058 | 10 | 6 | 3 | 197.837 | -2.852 | 0.014 | 26.707 | -2.735 | Yes | No | No | -1.855 | 0.202 | -0.982 | -3.407 | No | No | No | No | No | No | No | 0.684 | No | No | 0.902 | No | No | 2.251 | 2.876 | Yes |
| CHEMBL2219410 | O=C([O-])c1ccc(/C=C/S(=O)(=O)Cc2ccc(Cl)cc2)cc1.[Na+] | 358.778 | -0.7068 | 5 | 4 | 0 | 161.556 | -3.559 | -0.002 | 70.057 | -2.667 | No | No | No | -0.95 | 0.011 | -0.688 | -3.085 | No | Yes | No | No | No | No | No | -1.027 | No | No | 0.157 | No | No | 2.107 | 2.118 | Yes |
| CHEMBL2105674 | Cc1cc(F)ccc1-c1nc(NC(CO)CO)nc2c1ccc(=O)n2-c1c(F)cccc1F.Cc1ccc(S(=O)(=O)O)cc1 | 628.629 | 4.18024 | 7 | 9 | 4 | 249.409 | -2.945 | -0.405 | 50.293 | -2.735 | No | No | No | -0.952 | 0.273 | -2.545 | -4.156 | No | Yes | No | No | Yes | No | No | -0.364 | No | No | 0.817 | No | No | 2.777 | 2.722 | Yes |
| CHEMBL34913 | Cc1nc(C2CCCCC2)c(-c2ccc(S(N)(=O)=O)c(F)c2)o1 | 338.404 | 3.48422 | 3 | 4 | 1 | 134.317 | -4.661 | 1.062 | 93.324 | -2.942 | No | No | No | 0.014 | 0.066 | -0.692 | -2.182 | No | Yes | Yes | Yes | Yes | No | No | 1.104 | No | Yes | 0.219 | No | No | 2.707 | 1.215 | Yes |
| CHEMBL31965 | C=CC(=O)Nc1cc2c(Nc3ccc(F)c(Cl)c3)ncnc2cc1OCCCN1CCOCC1 | 485.947 | 4.3915 | 9 | 7 | 2 | 201.28 | -4.541 | 0.845 | 92.935 | -2.743 | Yes | Yes | Yes | 1.355 | 0.15 | -1.536 | -3.286 | No | Yes | No | No | No | No | Yes | 0.893 | Yes | No | 0.118 | No | Yes | 2.746 | 1.216 | Yes |
| CHEMBL42126 | COc1ccc(C(=O)Nc2c(Cl)cncc2Cl)cc1OC1CCCC1 | 381.259 | 4.9707 | 5 | 4 | 1 | 155.802 | -5.116 | 1.193 | 89.8 | -3.036 | No | No | No | -0.017 | 0.053 | 0.007 | -2.028 | No | Yes | Yes | Yes | Yes | No | Yes | 0.255 | No | No | 0.392 | No | Yes | 2.898 | 0.94 | Yes |
| CHEMBL4597193 | Cc1cc(C)c(CNC(=O)c2cc(Cl)c3c(c2C)O[C@@](C)([C@H]2CC[C@H](N(C)C)CC2)O3)c(=O)[nH]1 | 488.028 | 4.49136 | 5 | 5 | 2 | 205.334 | -4.647 | 0.992 | 90.79 | -3.04 | Yes | Yes | Yes | 0.799 | 0.134 | -1.16 | -2.898 | No | Yes | No | No | No | No | Yes | 0.673 | No | No | -0.258 | No | Yes | 2.672 | 1.163 | Yes |
| CHEMBL522502 | Nc1ccc2ncnc(NCCc3ccc(Oc4ccccc4)cc3)c2c1 | 356.429 | 4.6589 | 6 | 5 | 2 | 157.4 | -4.798 | 1.057 | 90.696 | -2.736 | Yes | Yes | Yes | -0.575 | 0.055 | -0.044 | -2.011 | No | Yes | Yes | Yes | Yes | No | Yes | 0.456 | No | Yes | 0.429 | No | Yes | 2.651 | 0.944 | Yes |
| CHEMBL3545283 | CN1CCC(c2c(O)cc(O)c3c(=O)cc(-c4ccccc4Cl)oc23)C1CO | 401.846 | 3.3046 | 3 | 6 | 3 | 165.766 | -4.021 | 0.876 | 82.657 | -2.746 | Yes | Yes | Yes | 0.686 | 0.109 | -1.218 | -2.377 | No | Yes | Yes | No | No | No | Yes | 0.525 | No | No | 0.489 | No | Yes | 2.731 | 2.388 | Yes |
| CHEMBL150315 | COc1cc2ncnc(Nc3ccc(Cl)cc3F)c2cc1OC | 333.75 | 4.1831 | 4 | 5 | 1 | 136.585 | -4.431 | 1.349 | 93.05 | -2.754 | No | Yes | Yes | -0.186 | 0.117 | -0.149 | -2.17 | No | No | Yes | Yes | Yes | No | Yes | 0.458 | No | No | 0.707 | No | Yes | 2.726 | 0.845 | Yes |
| CHEMBL433041 | CCCCCCCNC(=O)Oc1ccc2c(c1)[C@]1(C)CCN(C)[C@@H]1N2C | 359.514 | 4.1145 | 7 | 4 | 1 | 157.238 | -3.874 | 1.154 | 86.656 | -3.04 | Yes | Yes | Yes | 1.068 | 0.182 | 0.341 | -2.067 | No | Yes | No | No | No | Yes | No | 0.912 | Yes | No | -0.294 | No | No | 3.208 | 0.834 | Yes |
| CHEMBL288064 | Ic1cccc(CSc2nnc(-c3ccncc3)o2)c1 | 395.225 | 4.0285 | 4 | 5 | 0 | 133.717 | -3.601 | 1.395 | 95.888 | -2.593 | No | Yes | No | 0.06 | 0.103 | 0.223 | -2.124 | No | Yes | Yes | Yes | Yes | Yes | Yes | -0.324 | No | No | 0.116 | No | No | 2.396 | 0.619 | No |
| CHEMBL84336 | N#CS | 59.093 | 0.39728 | 0 | 2 | 1 | 24.287 | 0.099 | 1.346 | 100 | -2.907 | Yes | No | No | -0.107 | 0.773 | -0.071 | -2.59 | No | No | No | No | No | No | No | 0.213 | No | No | 1.309 | No | No | 2.604 | 1.37 | No |
| CHEMBL1256842 | CN1Cc2c(N)cccc2C(c2ccccc2)C1.O=C(O)/C=C\C(=O)O | 354.406 | 2.5579 | 3 | 4 | 3 | 151.73 | -2.983 | 0.528 | 33.729 | -2.735 | Yes | No | No | -1.128 | 0.267 | -0.691 | -2.243 | No | No | No | No | No | No | No | 0.348 | No | No | 0.484 | No | No | 2.213 | 1.381 | Yes |
| CHEMBL4802251 | CN(C)CC[C@H](NC(=O)c1cc2c(cnn2C(C)(C)C)cc1Oc1ccc(F)cc1F)C(N)=O | 473.524 | 3.3972 | 8 | 6 | 2 | 196.209 | -5.562 | 0.81 | 85.416 | -2.738 | Yes | Yes | Yes | -0.172 | 0.13 | -1.456 | -3.281 | No | Yes | No | Yes | Yes | No | Yes | 0.416 | No | No | 0.21 | No | Yes | 2.565 | 2.735 | Yes |
| CHEMBL289556 | O=C(N[C@H]1CCS[C@H]2CCC[C@@H](C(=O)O)N2C1=O)[C@@H](S)Cc1ccccc1 | 408.545 | 1.941 | 5 | 5 | 3 | 168.031 | -3.595 | 0.901 | 89.569 | -2.735 | Yes | No | No | -0.803 | 0.382 | -1.028 | -2.854 | No | No | No | No | No | No | No | 0.058 | No | No | -0.07 | No | No | 2.035 | 1.044 | Yes |
| CHEMBL3661408 | [O-][S+](c1ccccc1)C1OC1c1ccc(-c2ccccc2)cc1 | 320.413 | 4.5588 | 4 | 2 | 0 | 138.478 | -6.273 | 1.596 | 95.98 | -2.701 | Yes | Yes | Yes | 0.289 | 0.07 | 0.256 | -1.188 | No | Yes | Yes | Yes | Yes | No | No | 0.367 | No | No | 0.484 | No | Yes | 2.093 | 0.292 | Yes |
| CHEMBL4594454 | C[C@H]1CNCCCN1S(=O)(=O)c1cccc2cncc(F)c12.Cl.O.O | 395.884 | 0.5188 | 2 | 4 | 1 | 151.867 | -3.001 | 0.764 | 73.271 | -2.919 | No | No | No | -0.203 | 0.284 | -1.159 | -3.106 | No | Yes | No | No | No | No | No | 0.504 | No | No | -0.082 | No | No | 2.692 | 1.334 | Yes |
| CHEMBL4650219 | NC(CO)(CO)CO.O=C(O)C1=Cc2cc(OC(F)(F)F)ccc2OC1C(F)(F)F | 449.3 | 1.0373 | 5 | 7 | 5 | 164.29 | -3.015 | -0.473 | 27.505 | -2.735 | Yes | No | No | -1.645 | 0.518 | -1.993 | -4.424 | No | No | No | No | No | No | No | 0.141 | No | No | 0.936 | No | No | 2.347 | 2.282 | No |
| CHEMBL4596392 | CNC(=O)c1nnc(NC(=O)C2CC2)cc1Nc1cccc(-c2ncn(C)n2)c1OC | 422.449 | 1.7324 | 7 | 9 | 3 | 177.78 | -3.608 | 0.161 | 83.391 | -2.735 | Yes | Yes | No | -0.06 | 0.045 | -1.499 | -3.607 | No | No | No | No | No | No | No | -0.12 | No | No | 0.423 | No | Yes | 2.383 | 1.746 | Yes |
| CHEMBL261237 | N#C/C(=C(/N)Sc1ccc(N)cc1)c1ccccc1C(F)(F)F | 335.354 | 4.23078 | 3 | 4 | 2 | 134.951 | -3.706 | 0.715 | 87.825 | -2.79 | Yes | No | No | 0.006 | 0.133 | -0.122 | -1.685 | No | Yes | Yes | Yes | Yes | No | Yes | -0.037 | Yes | Yes | 0.336 | No | Yes | 2.813 | 1.386 | No |
| CHEMBL37858 | O=C(O)c1cn(-c2ccc(F)cc2)c2cc(N3CCNCC3)c(F)cc2c1=O | 385.37 | 2.3767 | 3 | 5 | 2 | 158.177 | -3.928 | 0.964 | 95.224 | -2.735 | Yes | No | No | 0.396 | 0.277 | -0.614 | -2.471 | No | Yes | Yes | No | No | No | No | 0.118 | No | No | 0.173 | No | Yes | 2.576 | 1.323 | Yes |
| CHEMBL273196 | CN[C@H](Cc1ccccc1)C(=O)N1CCC[C@H]1C(=O)N[C@H](C=O)CCCN=C(N)N | 416.526 | -0.4546 | 11 | 5 | 4 | 176.819 | -2.698 | 0.53 | 49.678 | -2.736 | Yes | No | No | 0.405 | 0.544 | -0.711 | -3.878 | No | Yes | No | No | No | No | No | 0.886 | No | No | 0.026 | No | No | 2.678 | 2.99 | Yes |
| CHEMBL4203190 | Cl.NCCCCN(Cc1nc2ccccc2[nH]1)[C@H]1CCCc2cccnc21 | 385.943 | 3.9982 | 7 | 4 | 2 | 166.35 | -2.778 | 1.055 | 84.249 | -2.735 | Yes | Yes | Yes | 0.042 | 0.099 | -0.985 | -2.431 | No | Yes | Yes | No | Yes | Yes | Yes | 1.736 | Yes | Yes | 0.203 | No | Yes | 2.567 | 0.591 | Yes |
| CHEMBL342372 | C[C@H](N[C@@H](CCc1ccccc1)C(=O)O)C(=O)N1N=C(C(C)(C)C)S[C@H]1C(=O)O | 421.519 | 2.3962 | 8 | 6 | 3 | 173.906 | -2.779 | 0.885 | 26.875 | -2.735 | No | No | No | -2.079 | 0.345 | -1.049 | -2.849 | No | No | No | No | No | No | No | -0.142 | No | No | 0.393 | No | No | 1.98 | 2.251 | Yes |
| CHEMBL4098877 | C[C@H]1CN(c2cc(-c3n[nH]c4ccc(OC5(C)CC5)cc34)ncn2)C[C@@H](C)O1 | 379.464 | 3.5648 | 4 | 6 | 1 | 163.631 | -3.317 | 1.243 | 94.917 | -2.814 | Yes | Yes | No | 0.663 | 0.156 | -0.439 | -3.26 | No | Yes | Yes | Yes | No | No | Yes | 0.638 | No | No | -0.277 | No | Yes | 3.212 | 1.484 | Yes |
| CHEMBL3039527 | Cl.Cn1cc(CNCC2CCN(c3ncc(C(=O)NO)cn3)CC2)c2ccccc21 | 430.94 | 2.5153 | 6 | 7 | 3 | 181.23 | -3.715 | 0.754 | 74.814 | -2.93 | Yes | Yes | Yes | 0.5 | 0.24 | -1.133 | -2.76 | No | Yes | No | No | No | No | Yes | 0.882 | No | No | 0.195 | No | Yes | 3.03 | 2.02 | Yes |
| CHEMBL4298160 | Cc1ccc(S(=O)(=O)O)cc1.Cn1cc(Nc2nccc(N3C[C@@H]4CC[C@H](C3)N4C(=O)[C@@H]3CC3(F)F)n2)cn1 | 561.615 | 3.03022 | 5 | 9 | 2 | 224.151 | -3.559 | 1.17 | 54.552 | -2.735 | Yes | No | No | -0.654 | 0.237 | -1.695 | -3.166 | No | No | No | No | No | No | No | -0.165 | No | No | 1.001 | No | No | 2.785 | 1.52 | Yes |
| CHEMBL4297651 | CO[C@H]1[C@H]([C@@]2(C)O[C@@H]2CC=C(C)C)[C@]2(CC[C@H]1OC(=O)N1CC(CCN3CCOCC3)C1)CO2 | 478.63 | 2.8535 | 8 | 7 | 0 | 203.393 | -3.741 | 1.213 | 95.461 | -3.057 | Yes | Yes | No | 0.306 | 0.417 | -0.681 | -3.075 | No | Yes | No | No | No | No | No | 1.004 | No | No | -0.9 | No | Yes | 3.01 | 1.332 | No |
| CHEMBL2105234 | C[C@]12C=CC(=O)C(N)=C1C=C[C@@H]1[C@@H]2CC[C@]2(C)C(=O)CC[C@@H]12 | 297.398 | 2.9258 | 0 | 3 | 1 | 130.879 | -4.296 | 1.36 | 97.899 | -3.44 | No | No | No | 0.218 | 0.208 | -0.049 | -2.791 | No | Yes | No | No | No | No | No | 0.049 | Yes | No | -0.547 | No | No | 2.269 | 1.617 | No |
| CHEMBL51314 | CC(C)(C)C(=O)Nc1ccc2c(c1)C(=O)C(=O)c1ccccc1-2 | 307.349 | 3.7172 | 1 | 3 | 1 | 134.072 | -4.596 | 1.189 | 96.642 | -2.78 | Yes | No | No | -0.097 | 0 | -0.213 | -1.801 | No | Yes | Yes | Yes | Yes | No | No | 0.081 | No | Yes | 0.149 | No | Yes | 2.391 | 2.187 | Yes |
| CHEMBL3661415 | O=[N+]([O-])c1ccc(C2OC2CO)cc1 | 195.174 | 1.027 | 3 | 4 | 1 | 80.081 | -2.433 | 0.51 | 76.272 | -2.92 | Yes | No | No | -0.124 | 0.391 | -0.226 | -2.581 | No | No | No | No | No | No | No | 0.507 | No | Yes | 0.614 | No | No | 1.824 | 1.696 | No |
| CHEMBL153427 | CCOC(=O)c1c(C)cc2c(CO)nnc(O)c2c1C | 276.292 | 1.62124 | 3 | 6 | 2 | 115.574 | -2.696 | 0.624 | 80.088 | -2.787 | Yes | No | No | -0.075 | 0.309 | -0.468 | -3.071 | No | No | No | No | No | No | No | 0.774 | No | No | 1.049 | No | No | 2.195 | 2.017 | Yes |
| CHEMBL411491 | NS(=O)(=O)c1ccc(N/N=C2\C(=O)Nc3ccc(Br)cc32)cc1 | 395.238 | 1.8648 | 3 | 5 | 3 | 140.307 | -3.37 | 0.696 | 78.181 | -2.911 | Yes | No | No | -0.436 | 0.046 | -0.888 | -2.544 | No | Yes | Yes | No | No | No | No | -0.306 | No | No | 0.088 | No | No | 1.692 | 2.248 | Yes |
| CHEMBL2219423 | Cl.Cn1ncc(Cl)c1-c1cc(C(=O)N[C@H](CN)Cc2cccc(F)c2)sc1Cl | 463.793 | 4.3161 | 6 | 5 | 2 | 181.594 | -5.103 | 1.069 | 88.789 | -2.789 | Yes | Yes | Yes | 0.615 | 0.075 | -0.734 | -2.188 | No | Yes | No | No | No | No | Yes | 0.92 | Yes | No | 0.146 | No | Yes | 2.072 | 2.089 | Yes |
| CHEMBL579 | C[C@H](N[C@@H](CCc1ccccc1)C(=O)O)C(=O)N1CC2(C[C@H]1C(=O)O)SCCS2 | 438.571 | 1.9123 | 8 | 6 | 3 | 178.158 | -2.815 | 0.656 | 25.745 | -2.735 | Yes | No | No | -2.149 | 0.328 | -1.017 | -2.965 | No | No | No | No | No | No | No | 0.294 | No | No | 0.525 | No | No | 1.932 | 2.978 | Yes |
| CHEMBL3545364 | CS(=O)(=O)O.O.O.O=c1ccc2ncc(=O)n3c2n1C[C@H]3CN1CCC(NCc2cc3c(cn2)OCCC3)CC1 | 580.664 | -1.0583 | 5 | 11 | 2 | 230.538 | -2.762 | -0.282 | 33.83 | -2.735 | Yes | No | No | -0.039 | 0.451 | -1.573 | -3.432 | No | Yes | No | No | No | No | No | 0.733 | No | No | 0.616 | No | No | 1.974 | -0.011 | Yes |
| CHEMBL3545124 | CN(C)C(C(=O)Nc1ccc2c(=O)[nH]ccc2c1)c1ccsc1.Cl | 363.87 | 3.2528 | 4 | 4 | 2 | 149.276 | -4.047 | 1.144 | 88.833 | -2.773 | Yes | Yes | Yes | 0.62 | 0.059 | 0.046 | -2.205 | No | Yes | Yes | Yes | No | No | No | 1.269 | No | No | 0.221 | No | Yes | 2.279 | 1.479 | Yes |
| CHEMBL304266 | CC1(C)CC2(CC(C)(C)c3cc(O)c(O)cc32)c2cc(O)c(O)cc21 | 340.419 | 4.1577 | 0 | 4 | 4 | 146.938 | -3.198 | 0.932 | 96.652 | -2.735 | Yes | No | No | 0.116 | 0.104 | -0.827 | -1.937 | No | No | Yes | Yes | Yes | No | No | 0.109 | No | No | 0.143 | No | Yes | 1.904 | 1.802 | No |
| CHEMBL481122 | Br.Br.Nc1ccccc1NC(=O)c1ccc(CNc2nccc(-c3cccnc3)n2)cc1 | 558.278 | 5.141 | 6 | 6 | 3 | 206.618 | -3.703 | 1.103 | 88.094 | -2.738 | Yes | Yes | Yes | 0.446 | 0.053 | -1.492 | -2.164 | No | Yes | Yes | Yes | Yes | No | Yes | -0.421 | No | No | 0.282 | No | Yes | 2.826 | 0.572 | Yes |
| CHEMBL1288786 | CCC(=O)Nc1nnc(S(N)(=O)=O)s1 | 236.278 | -0.466 | 3 | 6 | 2 | 84.386 | -2.742 | -0.053 | 59.712 | -2.888 | No | No | No | -0.567 | 0.615 | -0.624 | -3.252 | No | No | No | No | No | No | No | 0.011 | No | No | 1.357 | No | No | 2.618 | 1.928 | Yes |
| CHEMBL32838 | CO[C@H]1[C@H]([C@@]2(C)O[C@@H]2CC=C(C)C)[C@]2(CC[C@H]1OC(=O)/C=C/C=C/C=C/C=C/C(=O)O)CO2 | 458.551 | 3.9154 | 10 | 6 | 1 | 194.969 | -3.694 | 0.833 | 74.505 | -2.735 | No | No | Yes | -0.794 | 0.132 | -0.868 | -2.739 | No | Yes | No | No | No | No | No | 1.135 | No | No | -0.299 | No | No | 2.119 | 1.38 | No |
| CHEMBL4297217 | CCNC(=O)C[C@@H]1N=C(c2ccc(Cl)cc2)c2cc(OC)ccc2-n2c(C)nnc21.O=S(=O)(O)c1ccccc1 | 582.082 | 4.58932 | 6 | 8 | 2 | 237.001 | -3.628 | 0.351 | 72.393 | -2.735 | Yes | No | No | -0.735 | 0.147 | -1.225 | -3.257 | No | Yes | No | No | Yes | No | No | 0.02 | No | No | 0.811 | No | No | 2.601 | 0.666 | Yes |
| CHEMBL3666063 | Nc1c(C(=O)Nc2cccc(Cl)c2)sc2nc(-c3ccncc3)ccc12 | 380.86 | 4.8462 | 3 | 5 | 2 | 158.026 | -4.055 | 1.059 | 91.903 | -2.745 | Yes | Yes | Yes | 0.347 | 0.123 | -0.512 | -1.94 | No | Yes | Yes | Yes | Yes | No | Yes | -0.096 | No | Yes | 0.131 | No | Yes | 2.924 | 1.033 | Yes |
| CHEMBL273910 | O=C1CC2(C(=O)N1)C(=O)N(Cc1ccc(Br)cc1F)C(=O)c1ccc(F)cc12 | 449.207 | 2.1942 | 2 | 4 | 1 | 165.186 | -4.654 | 1.282 | 95.435 | -2.764 | Yes | Yes | No | -0.687 | 0.058 | -0.248 | -3.166 | No | Yes | No | Yes | No | No | Yes | -0.298 | No | No | 0.105 | No | No | 2.339 | 1.439 | Yes |
| CHEMBL2107578 | c1cnc2c(c1)C(Cc1ccncc1)(Cc1ccncc1)c1cccnc1-2 | 350.425 | 4.0185 | 4 | 4 | 0 | 157.57 | -3.89 | 1.019 | 100 | -2.695 | No | No | Yes | 0.624 | 0.385 | 0.335 | -2.191 | No | Yes | Yes | Yes | Yes | Yes | Yes | 0.308 | No | Yes | -0.032 | No | Yes | 2.313 | 1.039 | Yes |
| CHEMBL507614 | O=C(O)c1cc2sccc2[nH]1 | 167.189 | 1.9276 | 1 | 2 | 2 | 66.283 | -2.743 | 1.243 | 90.057 | -2.48 | No | No | No | -0.347 | 0.413 | 0.199 | -2.906 | No | No | No | No | No | No | No | 0.347 | No | No | 0.363 | No | No | 2.521 | 1.331 | No |
| CHEMBL2106281 | CCOCCOC(=O)c1nc2cc(Cl)ccc2o1 | 269.684 | 2.6745 | 5 | 5 | 0 | 108.644 | -3.202 | 1.356 | 95.679 | -2.707 | No | No | No | -0.323 | 0.221 | 0.349 | -2.993 | No | No | Yes | No | No | No | No | 0.293 | No | No | 1.282 | No | No | 2.788 | 0.971 | Yes |
| CHEMBL4587433 | O=C1NC(=O)/C(=C\c2c[nH]c(=O)c(-c3ccc(N4CCNCC4)nc3)c2)S1 | 383.433 | 1.1704 | 3 | 7 | 3 | 158.613 | -4.076 | -0.115 | 79.538 | -3.277 | Yes | No | No | -0.32 | 0.484 | -0.899 | -3.055 | No | Yes | Yes | No | No | No | No | 0.562 | No | Yes | 0.145 | No | No | 2.698 | 1.16 | Yes |
| CHEMBL3989942 | CC#Cc1cncc(-c2ccc3c(c2)[C@@]2(N=C(C)C(N)=N2)[C@]2(CC[C@H](OC)CC2)C3)c1.CC1(C)[C@@H]2CC[C@@]1(CS(=O)(=O)O)C(=O)C2 | 644.838 | 5.5056 | 4 | 8 | 2 | 271.842 | -3.513 | -0.409 | 64.817 | -2.735 | Yes | No | No | -0.383 | 0.055 | -1.056 | -2.743 | No | Yes | No | No | No | No | No | -0.344 | No | No | 0.539 | No | No | 3.068 | 1.614 | Yes |
| CHEMBL304818 | CC1=C(C)C(=O)C(CCCCC#CCCCC#CCO)=C(C)C1=O | 326.436 | 3.5209 | 6 | 3 | 1 | 144.769 | -4.506 | 1.376 | 97.36 | -2.867 | No | Yes | No | -0.251 | 0.1 | -0.128 | -2.196 | No | Yes | Yes | Yes | Yes | No | Yes | 1.678 | No | No | -0.815 | No | No | 1.588 | 1.974 | No |
| CHEMBL3661413 | CC(O)C1OC1c1ccc([N+](=O)[O-])cc1 | 209.201 | 1.4155 | 3 | 4 | 1 | 86.446 | -2.481 | 0.109 | 92.909 | -2.904 | No | No | No | -0.011 | 0.217 | -0.301 | -2.482 | No | Yes | Yes | No | No | No | No | 0.534 | No | Yes | 0.387 | No | No | 1.906 | 2.057 | No |
| CHEMBL261720 | Nc1nc(Nc2ccc(S(N)(=O)=O)cc2)nn1C(=S)Nc1c(F)cccc1F | 425.446 | 1.7746 | 4 | 8 | 4 | 162.968 | -3.857 | 0.458 | 73.522 | -2.738 | Yes | No | No | -0.458 | 0.062 | -1.592 | -3.52 | No | Yes | No | No | No | No | No | -0.645 | No | No | 0.746 | No | Yes | 2.129 | 1.399 | Yes |
| CHEMBL3643413 | CCC(=O)N1CC[C@H](Nc2ncnc3c2CN(c2cnc(OC)c(C(F)(F)F)c2)CC3)C1 | 450.465 | 2.8845 | 5 | 7 | 1 | 183.356 | -4.538 | 1.322 | 94.229 | -3.087 | No | No | No | -0.523 | 0.176 | -1.209 | -3.204 | No | Yes | No | No | No | No | No | 0.91 | No | No | -0.114 | No | No | 2.979 | 1.354 | Yes |
| CHEMBL85959 | Cc1nc(NCCCCCCc2ccccc2)nc(C)c1O | 299.418 | 4.01404 | 8 | 4 | 2 | 132.191 | -4.04 | 1.408 | 91.334 | -2.861 | Yes | Yes | No | 0.525 | 0.149 | 0.068 | -2.3 | No | Yes | Yes | Yes | Yes | No | No | 1.231 | No | No | 0.4 | No | Yes | 2.051 | 1.452 | Yes |
| CHEMBL2107829 | Cl.NCC[C@@H](O)c1cccc(OCC2CCCCC2)c1 | 299.842 | 3.4497 | 6 | 3 | 2 | 127.476 | -3.65 | 1.397 | 89.9 | -3.15 | No | No | No | 0.724 | 0.206 | -0.034 | -2.186 | No | Yes | Yes | No | No | Yes | No | 1.37 | No | No | -0.48 | No | Yes | 2.648 | 2.02 | No |
| CHEMBL2107131 | CCOC(=O)[C@H](C)C[C@@H](C)C(=O)N1c2ccccc2C[C@H]1C(=O)O | 333.384 | 2.2543 | 6 | 4 | 1 | 140.957 | -3.128 | 1.21 | 98.139 | -2.733 | No | No | No | -1.085 | 0.338 | -0.568 | -2.671 | No | No | No | No | No | No | No | 0.551 | No | No | 0.949 | No | No | 2.154 | 1.948 | No |
| CHEMBL2106909 | CCCCCC(/C=C/n1ccnc1)OCc1ccccc1 | 284.403 | 4.5195 | 9 | 3 | 0 | 126.836 | -2.796 | 1.904 | 93.05 | -2.752 | Yes | No | No | 0.523 | 0.166 | 0.516 | -1.68 | No | No | Yes | Yes | Yes | Yes | Yes | 1.217 | Yes | No | 0.815 | No | Yes | 2.426 | 2.122 | No |
| CHEMBL2106429 | CN(Cc1ccc(S(=O)(=O)N2CCOCC2)cc1)c1ccc2c3c(cccc13)C(=N)N2.O=C(O)[C@H]1O[C@@H](O)[C@H](O)[C@@H](O)[C@@H]1O | 630.676 | 0.11887 | 6 | 11 | 7 | 252.778 | -2.875 | -1.036 | 0 | -2.735 | Yes | No | No | -1.027 | 0.208 | -2.369 | -4.532 | No | No | No | No | No | No | No | 0.417 | No | No | 0.304 | No | No | 2.944 | 4.528 | Yes |
| CHEMBL2104622 | O=C(O)C1CCc2cc(OCCn3ccnc3)ccc21 | 272.304 | 2.0765 | 5 | 4 | 1 | 116.38 | -3.049 | 1.081 | 96.166 | -2.736 | Yes | No | No | -0.298 | 0.322 | -0.317 | -2.628 | No | No | No | No | No | No | No | 0.854 | Yes | No | 0.205 | No | No | 2.271 | 1.194 | Yes |
| CHEMBL3039536 | CC(=N)NCCSC[C@](C)(N)C(=O)O.CC(=N)NCCSC[C@](C)(N)C(=O)O.Cl.O=C(O)/C=C\C(=O)O | 591.153 | 0.35034 | 14 | 10 | 10 | 230.368 | -2.79 | -0.995 | 0 | -2.735 | No | No | No | -0.633 | 0.605 | -2.731 | -4.585 | No | No | No | No | No | No | No | -0.343 | No | No | 0.607 | No | No | 2.306 | 1.615 | Yes |
| CHEMBL2105388 | CCCC[C@H](N[C@@H](C)C(=O)N1[C@H](C(=O)OCC)C[C@@H]2CCCC[C@@H]21)C(=O)O | 382.501 | 2.3307 | 9 | 5 | 2 | 161.364 | -2.12 | 0.719 | 46.449 | -2.735 | Yes | No | No | -0.794 | 0.625 | -1.125 | -3.49 | No | No | No | No | No | No | No | 0.743 | No | No | 0.433 | No | No | 1.695 | 2.091 | Yes |
| CHEMBL3989608 | CCOC(=O)[C@H](CCc1ccccc1)N[C@@H](C)C(=O)N1C2CCC(CC2)[C@H]1C(=O)O | 416.518 | 2.3832 | 9 | 5 | 2 | 177.326 | -2.71 | 0.185 | 55.234 | -2.735 | Yes | No | No | -0.229 | 0.688 | -0.693 | -3.068 | No | Yes | No | No | No | No | No | 0.955 | No | No | 0.109 | No | No | 2.132 | 2.006 | Yes |
| CHEMBL2104960 | COc1ccc([C@H](C)Cn2cc[nH]c2=O)cc1OCC1CC1 | 302.374 | 2.7775 | 7 | 4 | 1 | 129.43 | -2.665 | 1.431 | 91.512 | -2.735 | Yes | Yes | No | 0.295 | 0.206 | -0.103 | -2.542 | No | Yes | Yes | Yes | No | Yes | No | 1.176 | Yes | Yes | 0.131 | No | Yes | 3.005 | 1.38 | No |
| CHEMBL2105633 | COc1cc2c(cc1OC)[C@@H]1C[C@H](N)[C@@H](N3C[C@@H](CF)CC3=O)CN1CC2.Cl.Cl | 450.382 | 2.3642 | 4 | 5 | 1 | 183.243 | -2.677 | 0.845 | 91.003 | -2.761 | Yes | No | No | 0.178 | 0.57 | -1.268 | -2.82 | Yes | Yes | No | No | No | No | No | 1.164 | No | No | -0.271 | No | Yes | 3.266 | 0.673 | Yes |
| CHEMBL2105433 | N=C(N)Nc1ccc(C(=O)Oc2ccc(SCCN3C(=O)CCC3=O)cc2)cc1 | 412.471 | 2.45227 | 7 | 6 | 3 | 171.458 | -3.524 | 1.078 | 70.79 | -2.768 | Yes | Yes | No | 1.182 | 0.16 | -1.315 | -2.924 | No | Yes | No | No | No | No | No | 0.868 | No | Yes | -0.182 | No | Yes | 2.222 | 1.699 | Yes |
| CHEMBL3137334 | CCNC(=O)c1noc(-c2cc(C(C)C)c(O)cc2O)c1-c1ccc(CN2CCOCC2)cc1.CS(=O)(=O)O.O | 579.672 | 2.8044 | 7 | 9 | 4 | 233.173 | -3.431 | -0.761 | 37.755 | -2.735 | Yes | No | Yes | -0.197 | 0.126 | -1.706 | -3.833 | No | Yes | No | No | No | No | No | 0.469 | No | No | 0.356 | No | Yes | 2.241 | 1.777 | Yes |
| CHEMBL4651180 | COc1ccc(-c2c(-c3ccc(C#N)c(F)c3)nc(N3CCC(N)CC3)n(C)c2=O)cc1F | 451.477 | 3.20028 | 4 | 7 | 1 | 189.207 | -4.47 | 0.476 | 90.961 | -2.75 | Yes | Yes | Yes | 0.679 | 0.225 | -1.193 | -2.394 | No | Yes | No | No | No | No | No | 0.142 | Yes | No | -0.126 | No | Yes | 2.732 | 1.042 | Yes |
| CHEMBL3301596 | Cl.N[C@@H]1CC[C@@H](c2ccc(Cl)c(Cl)c2)c2ccccc21 | 328.67 | 5.3407 | 1 | 1 | 1 | 135.042 | -5.615 | 1.413 | 91.613 | -2.224 | No | Yes | No | 1.528 | 0.027 | 0.102 | -1.585 | No | Yes | Yes | Yes | No | Yes | No | 1.199 | No | No | 0.113 | No | Yes | 2.696 | 0.669 | No |
| CHEMBL2105696 | COc1cccc(C(=O)Nc2ccc(OCCN3CCOCC3)c(-c3ccnn3C)c2)c1.Cl | 472.973 | 3.4808 | 8 | 7 | 1 | 199.396 | -4.629 | 1.235 | 93.587 | -2.78 | Yes | Yes | Yes | 0.624 | 0.049 | -0.741 | -2.816 | No | Yes | No | No | Yes | No | Yes | 0.969 | Yes | No | 0.142 | No | Yes | 2.765 | 0.601 | Yes |
| CHEMBL1201391 | CCC(C)(C)C(=O)O[C@H]1C[C@@H](C)C=C2C=C[C@H](C)[C@H](CC[C@@H](O)C[C@@H](O)CC(=O)O)[C@H]21 | 436.589 | 4.1057 | 10 | 5 | 3 | 185.926 | -2.912 | 0.621 | 49.089 | -2.735 | Yes | No | No | -0.849 | 0.276 | -1.279 | -3.306 | No | No | No | No | No | No | No | 1.01 | No | No | 0.283 | No | No | 2.207 | 2.521 | No |
| CHEMBL2107809 | CCc1c(C(=O)C(N)=O)c2c(OCC(=O)[O-])cccc2n1Cc1ccccc1.[Na+] | 402.382 | -1.9473 | 8 | 6 | 1 | 189.29 | -3.796 | 0.216 | 43.87 | -2.733 | Yes | No | No | -0.702 | 0.18 | -0.574 | -3.842 | No | Yes | No | No | No | No | No | 1.193 | No | No | 0.266 | No | No | 2.097 | 1.352 | Yes |
| CHEMBL2218880 | CC1CC(OC(=O)[C@@H]2CCC(=O)N2)CC(C)(C)C1 | 253.342 | 2.023 | 2 | 3 | 1 | 108.458 | -2.644 | 1.259 | 95.853 | -3.909 | No | No | No | 0.117 | 0.553 | 0.203 | -2.906 | No | No | No | No | No | No | No | 1.013 | No | No | 0.451 | No | No | 2.374 | 2.159 | No |
| CHEMBL2104522 | CC(C)Sc1ccc([C@H](O)[C@@H](C)N2CCN(C(=O)/C=C/c3ccccc3)CC2)cc1 | 424.61 | 4.4666 | 7 | 4 | 1 | 184.136 | -4.852 | 1.307 | 93.298 | -2.818 | Yes | Yes | Yes | 0.703 | 0 | -0.082 | -1.069 | Yes | Yes | No | Yes | No | Yes | Yes | 0.784 | Yes | No | -0.453 | No | Yes | 3.089 | 1.692 | Yes |
| CHEMBL2105716 | O=C([O-])CSc1nnc(Br)n1-c1ccc(C2CC2)c2ccccc12.[Na+] | 426.271 | -0.0936 | 5 | 6 | 0 | 179.176 | -3.63 | 1.257 | 73.367 | -2.715 | No | No | No | -0.327 | 0.145 | -0.926 | -3.107 | No | Yes | No | No | No | No | No | -0.967 | No | No | -0.312 | No | No | 2.713 | 0.357 | Yes |
| CHEMBL2105752 | CN(C)CCCOc1ccc2[nH]nc(S(=O)(=O)c3cccc4ccccc34)c2c1.Cl | 445.972 | 4.3012 | 7 | 5 | 1 | 181.726 | -4.321 | 1.174 | 91.16 | -2.735 | Yes | Yes | Yes | -0.335 | 0.095 | -1.285 | -2.484 | No | Yes | Yes | Yes | Yes | No | Yes | 0.798 | No | Yes | 0.214 | No | Yes | 2.851 | 1.413 | Yes |
| CHEMBL2107579 | CCCn1c(=O)c2ccccc2n2c(CN3CCCCC3)nnc12 | 325.416 | 2.4401 | 4 | 6 | 0 | 140.269 | -2.641 | 1.238 | 96.287 | -2.822 | Yes | No | No | 0.994 | 0.437 | -0.158 | -2.901 | No | Yes | Yes | No | No | No | No | 0.912 | No | No | -0.519 | No | Yes | 2.726 | 1.385 | Yes |
| CHEMBL4802253 | Cl.NCc1ccc2c(c1)nc(CN1C(=O)C3(CC3)c3ccc(F)cc31)n2CCCC(F)(F)F | 482.909 | 4.9768 | 6 | 4 | 1 | 193.523 | -2.902 | 1.412 | 80.605 | -2.735 | Yes | Yes | Yes | -0.637 | 0.26 | -0.279 | -1.983 | No | Yes | No | Yes | Yes | No | Yes | 1.496 | Yes | Yes | -0.271 | No | Yes | 2.289 | 0.745 | No |
| CHEMBL3137329 | Br.NC(=O)c1cnc2[nH]ccc2c1N[C@H]1[C@H]2CC3C[C@@H]1C[C@](O)(C3)C2 | 407.312 | 2.5912 | 3 | 4 | 4 | 155.897 | -2.844 | 0.881 | 86.19 | -2.816 | Yes | No | No | 0.415 | 0.365 | -1.131 | -4.275 | No | Yes | No | No | No | No | No | -0.244 | No | No | -0.551 | No | No | 2.565 | 1.209 | Yes |
| CHEMBL167055 | CC(=O)C(=Cc1cc(O)c(O)c([N+](=O)[O-])c1)C(C)=O | 265.221 | 1.5674 | 4 | 6 | 2 | 107.496 | -2.047 | 0.077 | 82.628 | -2.758 | Yes | No | No | -0.405 | 0.205 | -0.512 | -2.733 | No | No | No | No | No | No | No | 0.697 | No | No | 0.26 | No | No | 2.569 | 2.158 | No |
| CHEMBL2107774 | CCc1ccc(Nc2c(F)c(F)cc(F)c2F)c(CC(=O)O)c1 | 327.277 | 4.1761 | 5 | 2 | 2 | 129.116 | -3.594 | 1.377 | 92.849 | -2.735 | Yes | No | No | -0.71 | 0.15 | 0.344 | -2.813 | No | No | No | No | Yes | No | No | -0.32 | No | No | 0.891 | No | No | 2.555 | 2.586 | No |
| CHEMBL3707361 | CCOC(=O)[C@H](CCc1ccccc1)N[C@@H](C)C(=O)N1[C@H](C(=O)O)C[C@@H]2CCCC[C@@H]21.Cl | 467.006 | 3.1951 | 9 | 5 | 2 | 195.844 | -2.852 | 0.19 | 50.865 | -2.735 | Yes | No | No | -0.277 | 0.629 | -0.853 | -2.855 | No | Yes | No | No | No | No | No | 1.015 | No | No | 0.005 | No | No | 2.148 | 1.956 | Yes |
| CHEMBL331369 | CCOC(=O)[C@H](CCc1ccccc1)N[C@@H](C)C(=O)N1[C@H](C(=O)O)C[C@@H]2CCCC[C@@H]21 | 430.545 | 2.7733 | 9 | 5 | 2 | 183.691 | -2.766 | 0.228 | 51.11 | -2.735 | Yes | No | No | -0.221 | 0.652 | -0.718 | -2.921 | No | Yes | No | No | No | No | No | 0.707 | No | No | 0.014 | No | No | 2.108 | 2.013 | Yes |
| CHEMBL2107329 | COC(=O)Nc1nc2cc(Sc3ccc(NC(=O)[C@H](C)N)cc3)ccc2[nH]1.Cl | 421.91 | 3.5999 | 5 | 6 | 4 | 171.576 | -3.17 | -0.027 | 68.082 | -2.735 | Yes | Yes | No | 0.475 | 0.168 | -1.298 | -2.654 | No | No | No | No | No | No | No | 1.057 | No | No | 0.491 | No | No | 2.064 | 2.099 | No |
| CHEMBL417975 | CC(C)CN[C@H]1CCS(=O)(=O)c2sc(S(N)(=O)=O)cc21 | 338.476 | 0.8596 | 4 | 6 | 2 | 122.896 | -2.197 | 0.425 | 85.1 | -3.352 | No | No | No | -0.043 | 0.472 | -0.882 | -3.143 | No | No | No | No | No | No | No | 0.029 | No | No | 0.752 | No | No | 2.75 | 1.835 | Yes |
| CHEMBL4297665 | C#Cc1cccc(Nc2ncnc3cc4c(cc23)OCCOCCOCCO4)c1.Cl | 427.888 | 3.5809 | 2 | 7 | 1 | 180.68 | -4.543 | 1.489 | 94.409 | -2.774 | No | Yes | Yes | -0.151 | 0.016 | -0.586 | -2.781 | No | Yes | Yes | Yes | No | No | Yes | 0.458 | No | No | 0.019 | No | Yes | 2.667 | 1.031 | Yes |
| CHEMBL359570 | O=C1Cc2cc3c(CCC4CCN(Cc5ccccc5)CC4)noc3cc2N1 | 375.472 | 4.1672 | 5 | 4 | 1 | 164.244 | -4.4 | 0.936 | 90.482 | -2.825 | Yes | Yes | Yes | 1.355 | 0.064 | 0.063 | -1.978 | Yes | Yes | Yes | No | No | Yes | Yes | 1.254 | Yes | No | -0.426 | No | Yes | 2.663 | 1.742 | Yes |
| CHEMBL2103768 | CN(C(=O)C(Cl)Cl)c1ccc(O)cc1 | 234.082 | 2.1587 | 2 | 2 | 1 | 91.848 | -2.896 | 1.146 | 93.409 | -2.589 | No | No | No | -0.137 | 0.392 | -0.008 | -2.098 | No | No | Yes | No | No | No | No | 0.075 | No | Yes | 0.918 | No | No | 2.114 | 1.557 | No |
| CHEMBL2107064 | O=C(O)c1ccc2c(c1)C=C(n1ccnc1)CC2 | 240.262 | 2.5256 | 2 | 3 | 1 | 104.212 | -2.874 | 1.243 | 91.633 | -2.739 | Yes | No | No | -0.427 | 0.336 | 0.177 | -2.83 | No | No | Yes | No | No | No | No | 0.654 | No | Yes | 0.266 | No | No | 2.326 | 1.875 | No |
| CHEMBL577 | C[C@H](N[C@@H](CCc1ccccc1)C(=O)O)C(=O)N1CCC[C@H]1C(=O)O | 348.399 | 1.1261 | 8 | 4 | 3 | 146.188 | -2.598 | 0.235 | 21.221 | -2.735 | Yes | No | No | -2.214 | 0.473 | -0.927 | -3.306 | No | No | No | No | No | No | No | 0.459 | No | No | 0.541 | No | No | 1.794 | 2.913 | Yes |
| CHEMBL300841 | O=C(O)CN1C[C@@H](c2cccs2)SC[C@H](N[C@@H](CCc2ccccc2)C(=O)O)C1=O | 448.566 | 2.4934 | 9 | 6 | 3 | 182.643 | -2.949 | 0.63 | 32.888 | -2.735 | Yes | No | No | -1.544 | 0.318 | -1.282 | -2.993 | No | Yes | No | No | No | No | No | 0.203 | No | No | 0.295 | No | No | 2.293 | 2.051 | Yes |
| CHEMBL186537 | [N-]=[N+]=N | 43.029 | 0.87547 | 0 | 1 | 1 | 17.298 | 0.483 | 1.187 | 91.255 | -3.342 | Yes | No | No | 0.078 | 0.832 | -0.519 | -2.949 | No | No | No | No | No | No | No | 0.367 | No | Yes | 1.317 | No | No | 3.037 | 1.84 | No |
| CHEMBL1201236 | C[C@@](Cc1ccc(O)c(O)c1)(NN)C(=O)O | 226.232 | -0.0531 | 4 | 5 | 5 | 92.295 | -2.54 | -0.2 | 45.172 | -2.735 | Yes | No | No | -1.251 | 0.577 | -1.149 | -4.274 | No | No | Yes | No | No | No | No | 0.535 | No | Yes | 1.268 | No | No | 2.52 | 3.298 | Yes |
| CHEMBL2111119 | COc1ccc(Cl)c2c1CCCC2N(C)C | 239.746 | 3.2876 | 2 | 2 | 0 | 102.157 | -2.677 | 1.306 | 90.023 | -2.277 | No | No | No | 0.902 | 0.638 | 0.705 | -2.511 | No | No | Yes | No | No | Yes | No | 0.722 | No | No | 0.098 | No | No | 3.106 | 0.939 | No |
| CHEMBL2111075 | CCCCCOS(=O)(=O)O | 168.214 | 0.996 | 5 | 3 | 1 | 61.15 | -0.878 | 0.797 | 90.684 | -2.735 | No | No | No | -0.671 | 0.696 | -0.239 | -3.01 | No | No | No | No | No | No | No | 0.461 | No | No | 1.143 | No | No | 1.94 | 0.615 | No |
| CHEMBL4300558 | CN(C)C(=O)N1C[C@H]2C[C@](C)(NCC(=O)N3C[C@@H](F)C[C@H]3C#N)C[C@H]2C1 | 365.453 | 0.82068 | 3 | 4 | 1 | 153.636 | -3.638 | 0.628 | 72.134 | -3.409 | No | No | No | 0.141 | 0.632 | -0.699 | -3.167 | No | Yes | No | No | No | No | No | 0.293 | No | No | 0.148 | No | No | 2.686 | 0.871 | Yes |
| CHEMBL25892 | CC(C)C(NC(=O)Cn1c(-c2ccccc2)ncc(N)c1=O)C(=O)c1nnc(C(C)(C)C)o1 | 452.515 | 2.1967 | 7 | 9 | 2 | 190.786 | -4.107 | 0.116 | 73.302 | -2.741 | Yes | Yes | Yes | -0.292 | 0 | -0.987 | -3.479 | No | Yes | No | No | No | No | Yes | 0.429 | No | No | 0.186 | No | Yes | 2.169 | 2.027 | Yes |
| CHEMBL1233800 | CCOC(=O)Nc1cc2c(c(N)n1)N=C(c1ccccc1)[C@H](C)N2 | 325.372 | 3.167 | 3 | 6 | 3 | 139.502 | -3.913 | 0.472 | 87.397 | -2.806 | Yes | No | No | 0.3 | 0.244 | -0.839 | -2.349 | No | Yes | No | No | No | No | No | -0.144 | No | No | 0.004 | No | No | 2.57 | 0.502 | Yes |
| CHEMBL3661412 | CC(=O)C1OC1c1ccc([N+](=O)[O-])cc1 | 207.185 | 1.6237 | 3 | 4 | 0 | 85.813 | -2.833 | 0.113 | 95.435 | -2.789 | No | No | No | 0.033 | 0.21 | -0.315 | -2.432 | No | No | Yes | No | No | No | No | 0.509 | No | Yes | 0.652 | No | No | 1.951 | 2.043 | No |
| CHEMBL520358 | COc1cc(-c2ccccc2)cc(C(C)C#Cc2c(C)nc(N)nc2N)c1 | 358.445 | 3.78022 | 3 | 5 | 2 | 158.967 | -4.474 | 0.997 | 95.754 | -2.758 | Yes | Yes | Yes | -0.424 | 0 | 0.093 | -2.075 | No | Yes | Yes | Yes | Yes | No | Yes | 0.161 | No | Yes | -0.154 | No | Yes | 2.742 | 2.64 | Yes |
| CHEMBL1243298 | CN1CC[C@@]2(C)c3cc(OC(=O)N4CCc5ccccc5C4)ccc3N(C)[C@@H]12 | 377.488 | 3.6128 | 1 | 4 | 0 | 166.04 | -4.179 | 1.296 | 92.714 | -3.089 | Yes | Yes | No | 0.941 | 0.058 | 0.615 | -1.268 | No | Yes | No | No | No | Yes | Yes | 0.66 | Yes | No | -0.579 | No | Yes | 2.664 | 0.935 | Yes |
| CHEMBL1233897 | O=C(NC1CCN(CC(F)(F)F)CC1)c1ccc(-c2cccc(F)c2)nc1 | 381.373 | 3.6442 | 4 | 3 | 1 | 153.755 | -4.569 | 1.232 | 91.897 | -2.889 | Yes | No | No | 0.699 | 0.239 | 0.44 | -1.753 | No | Yes | No | No | No | Yes | No | 0.417 | No | No | -0.551 | No | Yes | 2.668 | 0.565 | Yes |
| CHEMBL1192519 | O=C(O)CN1C(=O)[C@@H](N[C@@H](CCc2ccccc2)C(=O)O)CCc2ccccc21 | 396.443 | 2.0946 | 8 | 4 | 3 | 168.515 | -2.992 | 1.125 | 38.295 | -2.735 | Yes | No | No | -1.615 | 0.226 | -0.784 | -2.903 | No | No | No | No | No | No | No | 0.328 | No | No | 0.343 | No | No | 2.117 | 1.368 | Yes |
| CHEMBL600689 | CCOC(=O)c1[nH]c(C)c(Cc2ccc(OCC)cc2)c1C | 301.386 | 3.79774 | 6 | 3 | 1 | 131.006 | -4.223 | 1.382 | 93.634 | -3.033 | No | Yes | No | 0.284 | 0.091 | 0.208 | -2.253 | No | Yes | Yes | Yes | Yes | No | Yes | 0.851 | No | No | 0.519 | No | No | 2.22 | 1.972 | Yes |
| CHEMBL4594378 | C[C@@H](Nc1nc(N[C@H](C)C(F)(F)F)nc(-c2cccc(Cl)n2)n1)C(F)(F)F.C[C@@H](Nc1nc(N[C@H](C)C(F)(F)F)nc(-c2cccc(Cl)n2)n1)C(F)(F)F.O.O=C(O)CC(O)(CC(=O)O)C(=O)O | 1039.62 | 6.5516 | 15 | 16 | 8 | 382.875 | -2.891 | -1.075 | 0 | -2.735 | Yes | No | No | -0.696 | 0.343 | -5.062 | -4.73 | No | Yes | No | No | No | No | No | -0.047 | No | No | 0.396 | No | No | 2.465 | 1.936 | Yes |
| CHEMBL3931782 | [Na+].[O-]c1c(-n2ccnn2)cnn1-c1cc(N2CCOCC2)ncn1 | 336.291 | -3.8428 | 3 | 10 | 0 | 158.922 | -2.732 | 0.679 | 63.612 | -2.782 | No | No | No | -0.985 | 0.369 | -1.532 | -3.333 | No | Yes | Yes | No | No | No | No | 1.321 | No | No | -0.426 | No | No | 2.034 | 0.92 | Yes |
| CHEMBL134920 | Cn1c([N+](=O)[O-])cnc1/C=C/c1ccnc(N)n1 | 246.23 | 0.8709 | 3 | 7 | 1 | 101.965 | -2.337 | -0.065 | 82.285 | -2.74 | No | No | No | 0.167 | 0.496 | -0.778 | -3.042 | No | No | No | No | No | No | No | 0.416 | No | Yes | 0.838 | No | No | 2.009 | 1.451 | Yes |
| CHEMBL63440 | CCOC(=O)[C@H]1O[C@@H]1C(=O)N[C@@H](CC(C)C)C(=O)NCCC(C)C | 342.436 | 1.0101 | 10 | 5 | 2 | 143.384 | -2.344 | 0.027 | 65.955 | -2.986 | Yes | No | No | -0.344 | 0.412 | -0.24 | -3.196 | No | No | No | No | No | No | No | 1.476 | No | No | 0.345 | No | No | 2.363 | 1.892 | No |
| CHEMBL3651708 | COc1ccc(/C=C2\SC(N3CCN(C)CC3)=NC2=O)cc1OC | 347.44 | 1.9215 | 3 | 6 | 0 | 145.671 | -3.416 | 1.31 | 93.778 | -3.146 | Yes | No | No | 0.599 | 0.301 | 0.029 | -2.47 | No | Yes | No | No | No | No | No | 0.504 | No | Yes | -0.266 | No | Yes | 2.578 | 1.373 | Yes |
| CHEMBL2219411 | Cl.Cl.N#CCNC(=O)c1ccc(-c2ccnc(Nc3ccc(N4CCOCC4)cc3)n2)cc1 | 487.391 | 3.82078 | 6 | 7 | 2 | 204.367 | -4.51 | 0.776 | 95.077 | -2.768 | Yes | Yes | Yes | 0.148 | 0.098 | -0.731 | -2.436 | No | Yes | Yes | Yes | No | No | Yes | 0.003 | No | No | 0.311 | No | Yes | 3.051 | 0.458 | Yes |
| CHEMBL2105197 | C[C@H](CSC(=O)[C@@H](C)NC(=O)C1CCCCC1)C(=O)N1CCC[C@H]1C(=O)O | 398.525 | 2.0429 | 7 | 5 | 2 | 164.17 | -2.872 | 0.854 | 42.132 | -2.735 | Yes | No | No | -1.048 | 0.409 | -0.854 | -2.887 | No | No | No | No | No | No | No | 0.148 | No | No | 0.289 | No | No | 2.003 | 1.758 | Yes |
| CHEMBL8706 | C#CCN(C)CCCOc1ccc(Cl)cc1Cl | 272.175 | 3.3273 | 6 | 2 | 0 | 112.465 | -3.406 | 1.335 | 92.291 | -2.211 | No | No | No | 0.972 | 0.348 | 0.739 | -1.908 | No | No | Yes | No | No | Yes | No | 0.816 | No | No | 0.704 | No | No | 2.859 | 0.936 | No |
| CHEMBL1090089 | Cn1c(=O)c(Oc2ccc(F)cc2F)cc2cnc(NC(CCO)CCO)nc21 | 406.389 | 1.9443 | 8 | 8 | 3 | 163.973 | -3.88 | 0.594 | 79.544 | -2.736 | Yes | Yes | No | -0.163 | 0.167 | -1.661 | -4.067 | No | No | No | No | No | No | No | 0.544 | No | No | 0.763 | No | Yes | 2.623 | 2.941 | Yes |
| CHEMBL3694253 | CCCNC(=O)c1ccc(-n2c(C)cc3ccccc32)s1 | 298.411 | 4.14022 | 4 | 3 | 1 | 127.766 | -4.544 | 1.18 | 92.337 | -2.472 | No | Yes | Yes | 0.502 | 0.124 | 0.28 | -1.413 | No | Yes | Yes | Yes | Yes | No | No | 0.2 | Yes | Yes | 0.191 | No | Yes | 2.536 | 0.867 | Yes |
| CHEMBL564131 | Cn1nc(C(C)(C)C)cc1C(=O)N[C@@H](Cc1cccc(Cl)c1)C(=O)NCC#N | 401.898 | 2.35188 | 6 | 5 | 2 | 169.229 | -4.617 | 1.003 | 76.468 | -2.992 | Yes | Yes | Yes | -0.191 | 0.116 | -0.211 | -2.559 | No | Yes | No | Yes | No | No | Yes | 0.037 | No | No | 0.19 | No | No | 1.934 | 1.645 | No |
| CHEMBL2105657 | C[C@H](O)C(=O)O.Cc1cc(Nc2cc(N3CCN(C)CC3)nc(Sc3ccc(NC(=O)C4CC4)cc3)n2)n[nH]1 | 554.677 | 2.95502 | 8 | 10 | 5 | 230.775 | -2.945 | -1.142 | 39.206 | -2.735 | Yes | No | No | 0.575 | 0.427 | -2.165 | -3.777 | Yes | Yes | No | No | No | No | No | 0.358 | No | No | 0.443 | No | No | 2.589 | 2.335 | Yes |
| CHEMBL2170434 | O=C(O)c1ccc(Nc2nccc(Nc3ccccc3C(F)(F)F)n2)cc1 | 374.322 | 4.6808 | 5 | 5 | 3 | 151.267 | -3.6 | 0.816 | 87.555 | -2.729 | Yes | No | No | -0.24 | 0.133 | -1.408 | -2.126 | No | No | Yes | No | No | No | No | -0.34 | No | No | 0.866 | No | No | 2.156 | 1.246 | No |
| CHEMBL1082354 | O=C(Nc1ncc([N+](=O)[O-])s1)c1cccs1 | 255.28 | 2.3651 | 3 | 6 | 1 | 97.718 | -3.328 | 0.73 | 88.205 | -2.793 | Yes | No | No | -0.545 | 0.213 | -0.805 | -2.339 | No | No | Yes | No | No | No | No | 0.024 | No | Yes | 0.214 | No | No | 2.746 | 1.443 | Yes |
| CHEMBL2219421 | O=C(O)c1ccc(/C=C/S(=O)(=O)Cc2ccc(Cl)cc2)cc1 | 336.796 | 3.6239 | 5 | 3 | 1 | 133.143 | -4.38 | 1.086 | 91.625 | -2.75 | No | No | No | -0.9 | 0.006 | -0.532 | -2.211 | No | Yes | No | No | No | No | No | -0.072 | No | No | 0.18 | No | No | 2.365 | 1.5 | No |
| CHEMBL4293433 | C=CC(=O)N1CCN(C(=O)CNc2cc(I)c(Cl)cc2O)CC1 | 449.676 | 1.9189 | 4 | 4 | 2 | 152.772 | -4.431 | 1.003 | 93.114 | -3.259 | Yes | No | No | 0.012 | 0.446 | -1.012 | -3.053 | No | No | No | No | No | No | No | 0.28 | No | No | 0.285 | No | No | 1.783 | 1.388 | No |
| CHEMBL3969876 | COCCCCn1c(C(=O)N(CC(C)C)[C@@H]2CNC[C@H](C(=O)N3CCOCC3)C2)nc2ccccc21.Cl | 536.117 | 2.8198 | 10 | 7 | 1 | 225.812 | -3.386 | 1.017 | 87.06 | -2.735 | Yes | Yes | No | 0.396 | 0.27 | -0.825 | -3.07 | No | Yes | No | No | No | No | Yes | 1.085 | Yes | Yes | 0.162 | No | Yes | 2.346 | 0.451 | Yes |
| CHEMBL2103790 | COC(=O)N1CC2(CC2)C[C@H](C(=O)NO)[C@H]1C(=O)N1CCN(c2ccccc2)CC1 | 416.478 | 1.0777 | 3 | 6 | 2 | 175.064 | -3.599 | 0.85 | 69.247 | -3.101 | Yes | No | No | -0.432 | 0.278 | -0.537 | -2.817 | No | No | No | No | No | No | No | 0.217 | No | Yes | -0.172 | No | No | 2.311 | 2.99 | Yes |
| CHEMBL3640071 | CC(C)C[C@H](NC(=O)c1ccc(N2CCN(C)CC2)cc1)C(=O)N1CC[C@H]2OCC(=O)[C@H]21 | 442.56 | 1.1517 | 6 | 6 | 1 | 189.41 | -3.241 | 0.757 | 74.479 | -3.643 | Yes | No | No | 0.279 | 0.355 | -0.705 | -3.394 | No | Yes | No | No | No | No | No | 0.749 | No | No | -0.454 | No | Yes | 2.671 | 1.66 | Yes |
| CHEMBL185238 | Cc1cccc(-c2n[nH]cc2-c2ccc3ncccc3n2)n1 | 287.326 | 3.39032 | 2 | 4 | 1 | 126.648 | -3.002 | 1.247 | 99.646 | -2.742 | Yes | No | No | 0.623 | 0.32 | 0.91 | -3.205 | No | Yes | Yes | No | No | No | No | 0.745 | No | No | 0.137 | No | Yes | 2.749 | 1.645 | Yes |
| CHEMBL3644461 | N[C@H]1C[C@@H](N2Cc3nc(C(F)(F)F)[nH]c3C2)CSC1c1cc(F)ccc1F | 404.408 | 3.5964 | 2 | 4 | 2 | 155.876 | -2.664 | 1.094 | 85.262 | -2.735 | Yes | No | No | -0.068 | 0.432 | 0.186 | -2.135 | No | Yes | No | No | No | Yes | No | 0.49 | No | Yes | -0.036 | No | Yes | 2.509 | 0.649 | Yes |
| CHEMBL3644465 | Cc1nc2c(o1)CN([C@H]1CSC(c3cc(F)ccc3F)[C@@H](N)C1)C2 | 351.422 | 3.15092 | 2 | 5 | 1 | 142.964 | -2.58 | 1.309 | 90.237 | -2.854 | Yes | No | No | 1.183 | 0.655 | 0.348 | -2.091 | No | Yes | No | No | No | Yes | No | 0.689 | No | No | -0.719 | No | Yes | 3.499 | 0.485 | Yes |
| CHEMBL356066 | O=C(/C=C/c1ccc(CN(CCO)CCc2c[nH]c3ccccc23)cc1)NO | 379.46 | 2.7235 | 9 | 4 | 4 | 163.623 | -3.354 | 0.806 | 92.33 | -2.737 | Yes | No | Yes | 0.891 | 0.214 | -1.019 | -2.774 | Yes | Yes | Yes | Yes | No | Yes | Yes | 1.29 | No | No | 0.429 | No | Yes | 2.59 | 3.294 | Yes |
| CHEMBL2111088 | COC(=O)Nc1nc2cc(Sc3ccc(NC(=O)[C@H](C)N)cc3)ccc2[nH]1 | 385.449 | 3.1781 | 5 | 6 | 4 | 159.423 | -3.178 | 0.012 | 68.326 | -2.735 | Yes | No | No | 0.48 | 0.169 | -1.163 | -2.72 | No | No | Yes | No | No | No | No | 0.748 | No | Yes | 0.496 | No | No | 2.059 | 2.135 | No |
| CHEMBL2105653 | CC[C@](O)(c1nnc(NCc2ccc3c(-c4ccc(F)cc4)cc(=O)oc3c2)o1)C(F)(F)F | 463.387 | 4.7541 | 6 | 7 | 2 | 183.227 | -3.71 | 0.574 | 93.619 | -2.737 | Yes | Yes | Yes | 0.206 | 0.147 | -0.778 | -2.4 | No | Yes | No | Yes | Yes | No | Yes | 0.18 | No | Yes | 0.49 | No | Yes | 3.023 | 0.397 | Yes |
| CHEMBL19215 | CN1C[C@H](CNC(=O)OCc2ccccc2)C[C@@H]2c3cccc4c3c(cn4C)C[C@H]21 | 403.526 | 4.0647 | 4 | 4 | 1 | 177.177 | -4.442 | 1.095 | 92.802 | -2.781 | Yes | Yes | Yes | 0.99 | 0 | 0.495 | -0.957 | No | Yes | Yes | No | No | Yes | Yes | 0.733 | No | No | -0.185 | No | Yes | 3.051 | 0.686 | Yes |
| CHEMBL414 | NS(=O)(=O)c1ccc(C(=O)O)cc1 | 201.203 | 0.0322 | 2 | 3 | 2 | 74.853 | -2.591 | 0.085 | 61.024 | -2.732 | No | No | No | -1.241 | 0.491 | -0.499 | -3.09 | No | No | No | No | No | No | No | 0.961 | No | No | 1.403 | No | No | 1.704 | 2.641 | No |
| CHEMBL2218882 | N#Cc1ccc(C(C(O)Cc2ccc(F)cc2)n2cncn2)cc1 | 322.343 | 2.48188 | 5 | 5 | 1 | 138.185 | -3.841 | 1.023 | 98.125 | -2.805 | No | No | No | -0.08 | 0.132 | -0.409 | -2.496 | No | Yes | Yes | Yes | Yes | No | No | 0.714 | No | No | 0.607 | No | Yes | 2.652 | 1.588 | Yes |
| CHEMBL2107099 | Cc1ccccc1C(=O)NP(N)(N)=O | 213.177 | 0.75032 | 2 | 2 | 3 | 82.011 | -2.585 | -0.127 | 71.461 | -2.894 | No | No | No | -0.401 | 0.547 | -0.814 | -2.717 | No | No | No | No | No | No | No | -0.222 | No | No | 1.246 | No | No | 2.239 | 1.795 | No |
| CHEMBL2107228 | CO[C@H](C(=O)NO)[C@@H](CC(C)C)C(=O)N[C@H](C(=O)Nc1ccccn1)C(C)(C)C | 408.499 | 1.7337 | 9 | 6 | 4 | 171.121 | -2.916 | 0.218 | 47.515 | -2.778 | Yes | No | No | -0.73 | 0.262 | -1.148 | -3.36 | No | No | No | No | No | No | No | 1.49 | No | No | 0.548 | No | Yes | 2.443 | 2.682 | Yes |
| CHEMBL572964 | O=S(=O)(O)O | 98.079 | -0.6528 | 0 | 2 | 2 | 29.006 | 0.372 | 0.436 | 84.415 | -2.735 | No | No | No | -1.065 | 0.771 | -0.235 | -3.484 | No | No | No | No | No | No | No | 0.702 | No | No | 1.424 | No | No | 1.462 | 2.084 | No |
| CHEMBL1788385 | Cc1cc(Br)c(O)c2ncccc12 | 238.084 | 3.01132 | 0 | 2 | 1 | 84.359 | -3.448 | 1.289 | 94.259 | -2.305 | No | No | No | 0.297 | 0.325 | 0.544 | -2.157 | No | No | Yes | No | No | No | No | 0.045 | No | Yes | 0.95 | No | No | 2.497 | 2.352 | No |
| CHEMBL3694254 | O=C(NC1CC1)c1ccc(N2CCSc3ccccc32)s1 | 316.451 | 3.8841 | 3 | 4 | 1 | 131.901 | -4.604 | 1.764 | 91.408 | -2.71 | Yes | Yes | No | 0.484 | 0.086 | 0.185 | -1.418 | Yes | Yes | Yes | Yes | Yes | No | No | 0.186 | Yes | Yes | -0.288 | No | Yes | 2.905 | 1.153 | No |
| CHEMBL2106094 | CCCN(c1ccncc1)n1ccc2ccccc21.Cl | 287.794 | 4.1378 | 4 | 3 | 0 | 124.787 | -4.098 | 1.702 | 96.231 | -2.314 | Yes | No | No | 0.4 | 0.237 | 0.277 | -1.676 | No | Yes | Yes | Yes | Yes | No | No | 0.1 | Yes | Yes | -0.35 | No | Yes | 2.161 | 0.326 | Yes |
| CHEMBL69139 | CN(C(=O)CCCOc1ccc2c(c1)CN1CC(=O)NC1=N2)C1CCCCC1 | 384.48 | 2.5696 | 6 | 5 | 1 | 165.289 | -2.977 | 0.904 | 85.734 | -2.735 | Yes | No | No | 0.335 | 0.347 | -0.565 | -2.705 | No | No | No | No | No | No | No | 1.115 | Yes | No | 0.215 | No | No | 3.072 | 0.842 | Yes |
| CHEMBL4541225 | COC(=O)C1=C(CN2CC(F)(F)C[C@H]2C(=O)O)NC(c2nccs2)=N[C@@]1(C)c1ccc(F)cc1 | 494.495 | 2.7687 | 6 | 8 | 2 | 196.324 | -3.214 | 0.511 | 68.608 | -2.735 | No | No | No | -1.153 | 0.291 | -1.416 | -3.658 | No | Yes | No | No | No | No | No | 0.109 | No | No | 0.571 | No | No | 2.245 | 2.059 | Yes |
| CHEMBL1788393 | CCOCCN(Cc1ccc(Oc2ccc([N+](=O)[O-])cc2)cc1)C(=O)C(Cl)Cl | 427.284 | 4.5559 | 10 | 5 | 0 | 172.451 | -6.555 | 0.487 | 90.491 | -2.726 | Yes | Yes | Yes | 0.153 | 0 | -0.891 | -2.519 | No | Yes | No | Yes | Yes | No | Yes | 0.304 | No | Yes | -0.022 | No | No | 2.356 | 2.053 | Yes |
| CHEMBL355001 | NS(N)(=O)=O | 96.111 | -1.8514 | 0 | 2 | 2 | 29.95 | 0.945 | 0.647 | 81.214 | -3.945 | No | No | No | -0.523 | 0.897 | -0.303 | -3.148 | No | No | No | No | No | No | No | 0.854 | No | Yes | 1.587 | No | No | 2.551 | 0.408 | No |
| CHEMBL19611 | CNC(=O)[C@H](Cc1c[nH]c2ccccc12)NC(=O)[C@@H](CC(=O)NO)CC(C)C | 388.47 | 4 | 5 | 123.32 | 1.5 |  |  |  |  |  |  |  |  |  |  |  |  |  |  |  |  |  |  |  |  |  |  |  |  |  |  |  |  |
| CHEMBL3694251 | Cc1ccccc1/C=C/c1cc(O)c2ccccc2n1 | 261.324 | 4.41922 | 2 | 2 | 1 | 117.589 | -4.416 | 1.338 | 90.975 | -2.695 | Yes | No | No | 0.254 | 0.095 | 0.704 | -0.931 | No | Yes | Yes | Yes | Yes | No | No | 0.709 | No | Yes | 0.432 | No | Yes | 2.12 | 2.172 | Yes |
| CHEMBL279786 | CNC(=O)[C@H](Cc1ccccc1)NC(=O)[C@H](CC(C)C)[C@H](CSc1cccs1)C(=O)NO | 477.652 | 3.0976 | 12 | 6 | 4 | 196.879 | -4.362 | -0.479 | 62.78 | -2.761 | Yes | Yes | Yes | -0.507 | 0.037 | -0.972 | -3.045 | No | Yes | No | Yes | No | No | Yes | 0.175 | No | No | -0.416 | No | No | 2.835 | 1.976 | Yes |
| CHEMBL3426621 | C[C@H]1CNCCCN1S(=O)(=O)c1cccc2cncc(F)c12 | 323.393 | 1.7464 | 2 | 4 | 1 | 128.753 | -2.994 | 1.224 | 96.686 | -2.964 | No | No | No | 0.211 | 0.325 | 0.134 | -2.956 | No | Yes | No | No | No | No | No | 0.186 | No | No | 0.227 | No | No | 3.044 | 1.265 | Yes |
| CHEMBL3707269 | CC[C@@H]1CN(C(=O)NCC(F)(F)F)C[C@@H]1c1cnc2cnc3[nH]ccc3n12.O.O.O.O.O=C(O)C(O)C(O)C(=O)O | 602.52 | -2.5135 | 6 | 8 | 6 | 228.015 | -2.891 | -1.181 | 0 | -2.735 | Yes | No | No | -0.698 | 0.427 | -2.662 | -5.801 | No | No | No | No | No | No | No | -0.24 | No | No | 0.403 | No | No | 2.471 | 3.143 | Yes |
| CHEMBL3644463 | NC(=O)Cn1cc2c(n1)CN([C@H]1CSC(c3cc(F)cc(F)c3F)[C@@H](N)C1)C2 | 411.453 | 1.6754 | 4 | 6 | 2 | 163.589 | -2.421 | 0.42 | 89.402 | -2.742 | Yes | No | No | 0.798 | 0.671 | -0.938 | -3.094 | No | No | No | No | No | No | No | 0.654 | No | No | -0.607 | No | No | 2.801 | 0.651 | Yes |
| CHEMBL2170435 | O=C(O)c1ccc(Nc2nccc(Nc3ccccc3Br)n2)cc1 | 385.221 | 4.4245 | 5 | 5 | 3 | 146.273 | -3.511 | 0.757 | 89.313 | -2.728 | Yes | No | No | -0.209 | 0.146 | -1.314 | -2.146 | No | No | Yes | No | No | No | No | -0.276 | No | No | 0.832 | No | No | 2.223 | 1.284 | Yes |
| CHEMBL4647810 | C[C@@H]1COCCN1c1cc(-c2ccnn2C)c2ccnc(-c3ccn[nH]3)c2n1 | 375.436 | 2.6455 | 3 | 7 | 1 | 161.728 | -3.182 | 0.997 | 100 | -2.74 | Yes | No | No | 0.574 | 0.326 | -1.015 | -3.53 | No | Yes | Yes | No | No | No | No | 0.479 | No | No | 0.074 | No | Yes | 2.795 | 1.238 | Yes |
| CHEMBL1817841 | O=[N+]([O-])OCCCO | 121.092 | -0.4229 | 4 | 4 | 1 | 45.984 | 0.061 | 0.756 | 82.851 | -3.515 | No | No | No | -0.432 | 0.714 | -0.363 | -3.069 | No | No | No | No | No | No | No | 0.811 | No | Yes | 1.131 | No | No | 2.461 | 0.475 | No |
| CHEMBL1965985 | CCOC(=O)Nc1cc2c(c(N)n1)N=C(c1ccccc1)[C@H](C)N2.O=S(=O)(O)CCO | 451.505 | 2.0335 | 5 | 9 | 5 | 179.661 | -3.187 | -0.744 | 37.432 | -2.735 | Yes | No | No | -1.268 | 0.326 | -1.563 | -3.711 | No | No | No | No | No | No | No | -0.505 | No | No | 1.04 | No | No | 2.111 | 2.218 | Yes |
| CHEMBL3651707 | COc1cc(-n2ccc(COc3ccccn3)cc2=O)ccc1OCC(C)(C)O | 396.443 | 2.9698 | 8 | 7 | 1 | 168.681 | -3.627 | 1.097 | 99.662 | -2.749 | No | Yes | No | -0.01 | 0.203 | -1.046 | -2.975 | No | Yes | Yes | Yes | No | No | Yes | 0.726 | No | No | -0.048 | No | No | 2.416 | 0.885 | Yes |
| CHEMBL3651709 | COc1cc(-n2ccc(CNc3ccc(Cl)cc3)cc2=O)ccc1OCC(C)(C)O | 428.916 | 4.2613 | 8 | 6 | 2 | 180.201 | -5.144 | 0.729 | 93.272 | -2.797 | Yes | Yes | Yes | 0.212 | 0.14 | -0.567 | -2.319 | No | Yes | Yes | Yes | Yes | No | Yes | -0.102 | No | No | 0.436 | No | Yes | 2.751 | 1.765 | Yes |
| CHEMBL2107785 | CCC(C)(C)C(=O)O[C@H]1C[C@@H](C)C=C2C=C[C@H](C)[C@H](CC[C@@H](O)C[C@@H](O)CC(=O)[O-])[C@H]21.CCC(C)(C)C(=O)O[C@H]1C[C@@H](C)C=C2C=C[C@H](C)[C@H](CC[C@@H](O)C[C@@H](O)CC(=O)[O-])[C@H]21.[Ca+2] | 911.24 | 5.1612 | 20 | 12 | 4 | 406.6 | -2.888 | -0.247 | 0 | -2.735 | Yes | No | No | -0.559 | 0.364 | -1.937 | -3.287 | No | Yes | No | No | No | No | No | 0.708 | No | No | 0.371 | No | No | 2.461 | 2.052 | No |
| CHEMBL2104968 | CO[P@](=O)(c1cc(C)cc(/C=C/C#N)c1)c1c(C(N)=O)[nH]c2ccc(Cl)cc12 | 413.801 | 3.6407 | 5 | 4 | 2 | 166.566 | -5.309 | 1.039 | 88.642 | -2.75 | Yes | Yes | Yes | -0.583 | 0 | -0.496 | -2.413 | No | Yes | No | Yes | Yes | No | Yes | -0.322 | No | Yes | -0.247 | No | Yes | 2.358 | 1.419 | Yes |
| CHEMBL4297430 | CN(C)C(=O)N1C[C@H]2C[C@](C)(NCC(=O)N3C[C@@H](F)C[C@H]3C#N)C[C@H]2C1.Cc1ccc(S(=O)(=O)O)cc1 | 537.658 | 2.0624 | 4 | 6 | 2 | 217.693 | -3.382 | 0.575 | 34.189 | -2.735 | Yes | No | No | -0.366 | 0.508 | -1.206 | -3.21 | No | No | No | No | No | No | No | 0.064 | No | No | 0.85 | No | No | 1.731 | 0.885 | Yes |
| CHEMBL1765292 | CCC(C(=O)O)c1ccc(N2Cc3ccccc3C2=O)cc1 | 295.338 | 3.4252 | 4 | 2 | 1 | 128.549 | -4.25 | 1.302 | 100 | -2.724 | No | No | No | -0.621 | 0.002 | -0.144 | -2.127 | No | Yes | No | No | No | No | No | 0.208 | No | No | 0.862 | No | No | 3.007 | 2.21 | Yes |
| CHEMBL865 | Cc1onc(-c2ccccc2)c1-c1ccc(S(N)(=O)=O)cc1 | 314.366 | 2.96442 | 3 | 4 | 1 | 127.977 | -3.844 | 1.265 | 94.173 | -2.715 | No | No | Yes | -0.365 | 0.183 | -0.62 | -2.19 | No | Yes | Yes | Yes | Yes | No | No | 0.838 | No | No | 0.523 | No | Yes | 2.149 | 0.329 | Yes |
| CHEMBL1502 | COc1ccnc(C[S+]([O-])c2nc3cc(OC(F)F)ccc3[nH]2)c1OC | 383.376 | 2.8843 | 7 | 6 | 1 | 150.044 | -2.99 | 0.914 | 79.479 | -2.735 | Yes | No | No | -0.086 | 0.225 | -0.98 | -3.472 | No | No | Yes | Yes | No | No | No | 0.716 | Yes | Yes | 0.089 | No | No | 1.872 | 1.443 | Yes |
| CHEMBL2220486 | CNC(=O)c1cccc2cc(Oc3ccnc4cc(OCC5(N)CC5)c(OC)cc34)ccc12 | 443.503 | 4.4186 | 7 | 6 | 2 | 191.012 | -4.081 | 0.977 | 100 | -2.735 | Yes | Yes | Yes | 0.181 | 0.252 | -0.462 | -2.439 | No | Yes | No | Yes | Yes | No | Yes | 1.087 | Yes | No | 0.542 | No | Yes | 2.622 | 1.063 | No |
| CHEMBL3402762 | CN1CCC(COc2cnc(-c3cccc(Cn4nc(-c5cccc(C#N)c5)ccc4=O)c3)nc2)CC1 | 492.583 | 4.00788 | 7 | 8 | 0 | 215.366 | -3.676 | 0.59 | 87.772 | -2.736 | No | Yes | Yes | 1.103 | 0.216 | -1.217 | -2.466 | No | Yes | No | No | Yes | No | Yes | 0.494 | Yes | No | 0.473 | No | Yes | 2.817 | 1.287 | Yes |
| CHEMBL198362 | O=C(NC[C@H]1CN(c2ccc(N3CCOCC3=O)cc2)C(=O)O1)c1ccc(Cl)s1 | 435.889 | 2.5199 | 5 | 6 | 1 | 175.486 | -4.973 | 1.109 | 93.806 | -3.448 | Yes | Yes | Yes | -0.438 | 0.086 | -0.934 | -2.596 | No | Yes | No | Yes | No | No | Yes | 0.298 | No | No | -0.439 | No | Yes | 2.907 | 1.963 | Yes |
| CHEMBL1614701 | Cn1cnc2c(F)c(Nc3ccc(Br)cc3Cl)c(C(=O)NOCCO)cc21 | 457.687 | 3.5256 | 6 | 6 | 3 | 166.728 | -3.369 | 0.989 | 90.212 | -2.735 | Yes | Yes | No | 0.712 | 0.215 | -1.481 | -2.536 | No | No | Yes | No | Yes | No | Yes | 0.738 | No | Yes | 0.349 | No | Yes | 2.4 | 2.141 | No |
| CHEMBL1873475 | C=CC(=O)N1CCC[C@@H](n2nc(-c3ccc(Oc4ccccc4)cc3)c3c(N)ncnc32)C1 | 440.507 | 4.2173 | 5 | 7 | 1 | 190.873 | -3.59 | 0.764 | 93.452 | -2.735 | Yes | Yes | Yes | 0.131 | 0.105 | -0.863 | -2.475 | No | Yes | No | Yes | Yes | No | Yes | 0.584 | No | No | 0.272 | No | Yes | 2.944 | 1.558 | Yes |
| CHEMBL56337 | CC(/C=C1\SC(=S)N(CC(=O)O)C1=O)=C\c1ccccc1 | 319.407 | 2.9187 | 4 | 4 | 1 | 131.69 | -3.386 | 1.336 | 90.773 | -2.735 | No | No | No | -0.859 | 0.287 | 0.127 | -2.814 | No | No | No | No | No | No | No | 0.108 | No | No | 0.698 | No | No | 2.317 | 1.335 | No |
| CHEMBL68253 | NS(=O)(=O)O | 97.095 | -1.2521 | 0 | 2 | 2 | 29.478 | 0.43 | 0.538 | 82.411 | -2.735 | No | No | No | -0.809 | 0.828 | -0.244 | -3.125 | No | No | No | No | No | No | No | 0.832 | No | No | 1.405 | No | No | 1.715 | 0.463 | No |
| CHEMBL48361 | Cn1c(CNc2ccc(C(=N)N)cc2)nc2cc(C(=O)N(CCC(=O)O)c3ccccn3)ccc21 | 471.521 | 2.98597 | 9 | 7 | 4 | 201.092 | -2.954 | -0.44 | 57.219 | -2.735 | Yes | No | No | 0.652 | 0.163 | -1.37 | -3.139 | No | Yes | No | No | Yes | No | No | 0.552 | No | No | 0.211 | No | No | 2.451 | 1.766 | Yes |
| CHEMBL4297865 | C=CC(=O)Nc1cccc(Oc2nc(Nc3ccc(N4CCN(C)CC4)c(F)c3)nc3[nH]ccc23)c1 | 487.539 | 4.5092 | 7 | 7 | 3 | 207.16 | -3.483 | 1.04 | 89.035 | -2.735 | Yes | Yes | Yes | 0.788 | 0 | -1.497 | -2.783 | No | Yes | Yes | Yes | Yes | No | Yes | 0.592 | No | No | 0.856 | No | Yes | 2.766 | 2.817 | Yes |
| CHEMBL1278118 | COc1cc(OC)cc(N2CCN(C(=O)Nc3nc4cc(F)ccc4nc3OC)CC2)c1 | 441.463 | 3.1488 | 5 | 7 | 1 | 184.26 | -4.456 | 1.262 | 94.491 | -2.74 | Yes | Yes | Yes | -0.331 | 0.093 | -1.018 | -3.374 | No | Yes | No | No | Yes | No | Yes | 0.545 | No | No | 0.623 | No | Yes | 2.503 | 1.763 | Yes |
| CHEMBL4802161 | CC(=O)c1c(C)c2cnc(Nc3ccc(C4CCNCC4)cn3)nc2n(C2CCCC2)c1=O | 446.555 | 4.02322 | 5 | 8 | 2 | 192.414 | -3.664 | 1.332 | 94.64 | -2.88 | Yes | Yes | No | 0.853 | 0.302 | -0.911 | -3.024 | No | Yes | No | No | No | No | Yes | 0.786 | No | No | -0.766 | No | Yes | 2.977 | 1.37 | Yes |
| CHEMBL3989867 | O=C(NCc1c(F)cc(F)cc1F)c1cn2c(c([O-])c1=O)C(=O)N1[C@H]3CC[C@H](C3)O[C@@H]1C2.[Na+] | 471.367 | -1.9936 | 3 | 6 | 1 | 207.162 | -3.197 | 1.19 | 66 | -3.09 | Yes | No | No | -0.535 | 0.291 | -1.089 | -4.06 | No | Yes | No | No | No | No | No | 0.872 | No | No | -0.201 | No | No | 2.277 | 1.322 | Yes |
| CHEMBL1201162 | CCN[C@H]1C[C@H](C)S(=O)(=O)c2sc(S(N)(=O)=O)cc21.Cl | 360.91 | 1.0338 | 3 | 6 | 2 | 128.685 | -2.422 | 0.39 | 84.621 | -3.346 | No | No | No | -0.122 | 0.439 | -1.088 | -3.169 | No | No | No | No | No | No | No | 0.555 | No | No | 0.526 | No | No | 2.955 | 1.676 | Yes |
| CHEMBL3301600 | C=CC(=O)Nc1cccc(Nc2nc(Nc3ccc(OCCOC)cc3)ncc2F)c1.O=S(=O)(O)c1ccccc1 | 581.626 | 5.1859 | 11 | 9 | 4 | 236.38 | -3.132 | -0.226 | 55.46 | -2.735 | Yes | No | No | -0.383 | 0.021 | -2.073 | -3.551 | No | Yes | No | No | No | No | No | -0.632 | No | No | 0.989 | No | No | 2.238 | 2.097 | Yes |
| CHEMBL1201198 | Cc1cccn2c(=O)c(-c3nnn[nH]3)cnc12 | 228.215 | 0.18302 | 1 | 6 | 1 | 95.137 | -2.501 | 1.257 | 94.074 | -2.735 | No | No | No | 0.448 | 0.362 | -1.249 | -4.2 | No | No | Yes | No | No | No | No | 0.656 | No | No | 0.652 | No | No | 2.175 | 2.775 | Yes |
| CHEMBL281398 | CC(C)(C)/N=C(\NC#N)Nc1cccc(/C(=C\CCCC(=O)O)c2cccnc2)c1 | 405.502 | 4.40528 | 7 | 4 | 3 | 176.741 | -3.052 | 0.662 | 60.676 | -2.735 | Yes | No | Yes | 1.252 | 0.197 | -0.862 | -2.295 | No | Yes | No | No | No | No | No | -0.031 | No | No | 0.128 | No | No | 2.439 | 1.547 | Yes |
| CHEMBL2103772 | CC(=O)SCC(Cc1ccccc1)C(=O)NCC(=O)OCc1ccccc1 | 385.485 | 2.9846 | 9 | 5 | 1 | 163.044 | -5.082 | 0.982 | 94.112 | -2.734 | No | Yes | Yes | -0.179 | 0 | -0.533 | -2.677 | No | Yes | Yes | Yes | Yes | Yes | Yes | 0.075 | No | No | 0.465 | No | Yes | 2.038 | 1.585 | No |
| CHEMBL4227736 | Cc1ccc(C(=O)Oc2ccc(C(CN(C)C)C3(O)CCCCC3)cc2)cc1 | 381.516 | 4.55462 | 6 | 4 | 1 | 167.691 | -4.341 | 0.911 | 91.784 | -2.948 | Yes | Yes | Yes | 0.844 | 0.084 | 0.134 | -2.1 | No | Yes | No | No | No | Yes | No | 0.748 | No | No | 0.219 | No | Yes | 2.934 | 1.18 | Yes |
| CHEMBL4650361 | C[C@@H](N)COc1ccc(-c2cnc3ccc(N[C@H](C)c4cccc(F)c4)nn23)cc1.O=C(O)CCCCC(=O)O | 551.619 | 5.1505 | 12 | 8 | 4 | 231.172 | -2.895 | -0.651 | 41.962 | -2.735 | Yes | No | Yes | -0.172 | 0.13 | -1.99 | -3.418 | No | No | No | No | Yes | No | No | 0.396 | No | No | 0.432 | No | No | 2.446 | 1.591 | Yes |
| CHEMBL1182714 | CNCCCOc1cc(F)c(-c2c(Cl)nc3ncnn3c2N[C@@H](C)C(F)(F)F)c(F)c1 | 464.826 | 4.0739 | 8 | 7 | 2 | 178.133 | -3.534 | 1.213 | 90.141 | -2.738 | Yes | Yes | No | 0.739 | 0.196 | -0.87 | -3.191 | No | Yes | No | No | Yes | No | Yes | 0.024 | No | No | 0.27 | No | Yes | 2.453 | 1.022 | Yes |
| CHEMBL408513 | O=C(/C=C/c1cccc(S(=O)(=O)Nc2ccccc2)c1)NO | 318.354 | 2.006 | 5 | 4 | 3 | 127.664 | -3.605 | 0.128 | 76.558 | -2.807 | Yes | No | No | -0.39 | 0.011 | -0.813 | -2.69 | No | Yes | Yes | No | No | No | No | 0.511 | No | No | 0.325 | No | Yes | 2.852 | 2.58 | No |
| CHEMBL1232111 | C#CCN(C)CC1=Cc2ccccc2Oc2ccccc21 | 275.351 | 3.8979 | 3 | 2 | 0 | 125.22 | -4.068 | 1.467 | 97.416 | -2.49 | Yes | Yes | No | 1.62 | 0.076 | 0.773 | -1.296 | No | Yes | No | No | No | Yes | No | 0.801 | Yes | No | 0.229 | No | Yes | 2.964 | 0.959 | Yes |
| CHEMBL332750 | CCOc1ccc(-c2nc(-c3cccc(C(=O)O)n3)cs2)cc1OCC | 370.43 | 4.3677 | 7 | 6 | 1 | 154.633 | -3.557 | 1.137 | 94.559 | -2.734 | No | No | No | -0.254 | 0.253 | -1.01 | -3.004 | No | No | No | No | No | No | No | 0.345 | No | No | 0.619 | No | No | 2.073 | 0.835 | Yes |
| CHEMBL126159 | COc1cc2c3c(n(CCCNCCO)c(=O)c2cc1OC)-c1cc2c(cc1C3=O)OCO2 | 452.463 | 1.9308 | 8 | 9 | 2 | 188.994 | -4.214 | 0.703 | 100 | -2.758 | Yes | Yes | Yes | 0.504 | 0.176 | -1.064 | -3.644 | No | Yes | No | No | No | No | Yes | 1.13 | Yes | No | 0.272 | No | Yes | 2.468 | 1.888 | No |
| CHEMBL157138 | CCN(CC)C(=O)N[C@H]1C=C2c3cccc4[nH]cc(c34)C[C@H]2N(C)C1 | 338.455 | 2.8414 | 3 | 2 | 2 | 148.231 | -3.092 | 0.906 | 90.477 | -2.762 | Yes | No | No | 1.629 | 0.6 | 0.185 | -2.208 | No | Yes | No | No | No | Yes | No | 0.862 | No | No | -0.84 | No | Yes | 3.103 | 0.55 | Yes |
| CHEMBL2103863 | CN(C)Cc1c(C(=O)NCCOc2ccc(C(=O)NO)cc2)oc2ccccc12 | 397.431 | 2.4222 | 8 | 6 | 3 | 167.564 | -4.586 | 1.152 | 74.885 | -2.783 | Yes | Yes | No | 0.167 | 0.077 | -0.979 | -2.973 | No | Yes | No | No | No | No | No | 0.658 | No | No | 0.088 | No | Yes | 2.698 | 2.034 | Yes |
| CHEMBL4297489 | Cc1nn(C)cc1CN1CCC(c2ccc(C(=O)Nc3ccccc3N)cc2)CC1 | 403.53 | 3.94262 | 5 | 5 | 2 | 177.243 | -3.774 | 1.09 | 91.2 | -2.753 | Yes | Yes | Yes | 0.859 | 0.1 | -0.475 | -2.028 | No | Yes | Yes | Yes | Yes | Yes | Yes | 0.958 | Yes | No | -0.168 | No | Yes | 2.653 | 1.921 | Yes |
| CHEMBL4650360 | COc1ccc(-c2c(-c3ccc(C#N)c(F)c3)nc(N3CCC(N)CC3)n(C)c2=O)cc1F.O=S(=O)(O)c1ccccc1 | 609.655 | 4.13358 | 5 | 9 | 2 | 246.899 | -3.771 | -0.231 | 59.819 | -2.735 | Yes | No | Yes | -0.626 | 0.222 | -1.455 | -3.291 | No | Yes | No | No | No | No | No | 0.064 | No | No | 0.881 | No | No | 2.915 | 0.726 | Yes |
| CHEMBL506871 | C[C@]1(c2nc3cccc(C(N)=O)c3[nH]2)CCCN1 | 244.298 | 1.2604 | 2 | 3 | 3 | 104.777 | -2.848 | 0.192 | 83.705 | -2.735 | Yes | No | No | 0.294 | 0.349 | -0.82 | -3.755 | No | No | Yes | No | No | No | No | 1.189 | Yes | Yes | 0.337 | No | No | 3.067 | 1.285 | Yes |
| CHEMBL673 | C#CCN(C)Cc1ccccc1 | 159.232 | 1.7516 | 3 | 1 | 0 | 74.015 | -1.522 | 1.538 | 94.83 | -1.727 | No | No | No | 0.99 | 0.455 | 0.784 | -1.602 | Yes | No | No | No | No | Yes | No | 0.935 | Yes | No | 0.514 | No | No | 2.418 | 1.583 | No |
| CHEMBL3936761 | C=CC(=O)N1CCC([C@@H]2CCNc3c(C(N)=O)c(-c4ccc(Oc5ccccc5)cc4)nn32)CC1 | 471.561 | 4.2226 | 6 | 6 | 2 | 203.917 | -5.206 | 0.599 | 93.23 | -2.803 | Yes | Yes | Yes | 0.331 | 0.04 | -0.947 | -2.359 | No | Yes | Yes | Yes | Yes | No | Yes | 0.434 | No | No | 0.053 | No | Yes | 2.879 | 0.681 | Yes |
| CHEMBL1922235 | COc1ccc(C(=O)Nc2cccc(O)c2NC(=O)c2ccc(N3CCCN(C)CC3)cc2)cc1 | 474.561 | 4.0473 | 6 | 6 | 3 | 204.674 | -4.178 | 1.052 | 85.426 | -2.735 | Yes | Yes | Yes | 0.426 | 0.001 | -0.867 | -2.352 | No | Yes | No | Yes | Yes | No | Yes | 0.677 | No | No | 0.24 | No | Yes | 2.33 | 2.331 | Yes |
| CHEMBL3188267 | CNC(=O)c1ccc(-c2cnc3ncc(Cc4ccc5ncccc5c4)n3n2)cc1F | 412.428 | 3.429 | 4 | 6 | 1 | 176.164 | -2.904 | 1.433 | 96.373 | -2.735 | Yes | Yes | Yes | -0.364 | 0.28 | -1.154 | -3.35 | No | Yes | Yes | Yes | Yes | Yes | Yes | 0.715 | Yes | No | 0.331 | No | Yes | 2.488 | 0.88 | Yes |
| CHEMBL1631694 | CSc1ccc(OCc2ncc([N+](=O)[O-])n2C)cc1 | 279.321 | 2.6292 | 5 | 6 | 0 | 114.112 | -2.993 | 0.932 | 91.001 | -2.738 | Yes | No | No | 0.368 | 0.292 | -0.874 | -2.386 | No | No | Yes | Yes | No | No | No | 0.701 | Yes | Yes | 0.772 | No | No | 2.028 | 1.541 | No |
| CHEMBL295392 | CNC(=O)[C@H](Cc1ccc(OC)cc1)NC(=O)C1(CC(=O)NO)CCCCC1 | 391.468 | 1.3145 | 8 | 5 | 4 | 164.531 | -2.914 | 0.407 | 63.443 | -2.797 | Yes | Yes | No | -0.606 | 0.235 | -0.807 | -3.007 | No | No | No | No | No | No | No | 1.424 | No | No | -0.315 | No | Yes | 2.642 | 1.629 | Yes |
| CHEMBL2106126 | CCOC(=O)[C@H](CCc1ccccc1)N[C@@H](C)C(=O)N(CC(=O)O)C1Cc2ccccc2C1.Cl | 489.012 | 3.0314 | 11 | 5 | 2 | 206.447 | -3.231 | 0.012 | 49.104 | -2.735 | Yes | No | No | -0.168 | 0.372 | -0.726 | -3.029 | No | Yes | No | No | No | No | No | 1.211 | No | No | -0.267 | No | No | 2.199 | 2.03 | Yes |
| CHEMBL4298155 | CCOc1c([C@H](C)n2nc(C)c3c(N)ncnc32)cc(Cl)c(F)c1[C@@H]1CNC(=O)C1.Cl | 469.348 | 3.54272 | 5 | 7 | 2 | 189.282 | -3.992 | 0.768 | 89.301 | -2.741 | Yes | Yes | No | 0.143 | 0.158 | -1.333 | -3.12 | No | Yes | No | No | Yes | No | Yes | 0.148 | No | No | 0.166 | No | Yes | 2.397 | 1.226 | Yes |
| CHEMBL4298153 | Br.Br.CCC[C@H](N[C@H]1CCc2cc(F)cc(F)c2C1)C(=O)Nc1cn(C(C)(C)CNCC(C)(C)C)cn1 | 651.479 | 5.9422 | 10 | 5 | 3 | 240.858 | -3.069 | 0.735 | 83.157 | -2.735 | Yes | Yes | No | 0.139 | 0.317 | -1.472 | -2.351 | No | Yes | No | No | No | Yes | Yes | 1.291 | No | No | 0.244 | No | Yes | 2.609 | 1.003 | No |
| CHEMBL4802241 | N#CCC1(n2cc(-c3ncnc4[nH]ccc34)cn2)CN(S(=O)(=O)C2CC2)C1 | 383.437 | 1.23818 | 5 | 7 | 1 | 155.247 | -2.542 | 0.976 | 84.897 | -2.808 | Yes | No | No | 0.439 | 0.246 | -0.756 | -3.499 | No | Yes | No | No | No | No | No | 0.848 | No | No | -0.302 | No | Yes | 2.245 | 1.863 | Yes |
| CHEMBL4650367 | CC(=O)N[C@@H](Cc1c[nH]c2ccccc12)C(=O)N[C@@H](CCC(=O)C=[N+]=[N-])C(=O)OC(C)C | 441.488 | 1.3014 | 11 | 5 | 3 | 184.873 | -3.551 | -0.047 | 64.848 | -2.753 | Yes | Yes | No | 0.471 | 0.298 | -1.082 | -3.286 | No | No | No | No | No | No | Yes | 1.038 | No | No | -0.231 | No | Yes | 2.246 | 1.819 | Yes |
| CHEMBL4594276 | Cl.N#CCC1CCN(c2nc(Nc3ccc(N4CCC(O)CC4)cc3)c3c(=O)[nH]ncc3n2)CC1 | 497.003 | 2.96968 | 5 | 9 | 3 | 208.956 | -3.324 | 1.06 | 73.985 | -2.74 | Yes | Yes | Yes | 0.455 | 0.034 | -1.676 | -2.835 | No | Yes | No | No | Yes | No | Yes | -0.17 | No | No | 0.324 | No | Yes | 2.904 | 2.272 | Yes |
| CHEMBL182319 | CN[C@H](C(=O)N[C@H](C(=O)N(C)[C@H](/C=C(\C)C(=O)O)C(C)C)C(C)(C)C)C(C)(C)c1ccccc1 | 473.658 | 3.597 | 10 | 4 | 3 | 204.544 | -3.638 | 0.804 | 48.532 | -2.735 | Yes | No | No | -0.549 | 0.198 | -0.588 | -2.374 | No | Yes | No | No | No | No | No | -0.126 | No | No | -0.404 | No | No | 3.062 | 1.05 | No |
| CHEMBL309962 | O=C(O)[C@@H]1CS[C@H](c2ccccc2O)N1C(=O)CCS | 313.4 | 1.7393 | 4 | 5 | 3 | 125.93 | -2.653 | 0.947 | 93.17 | -2.745 | Yes | No | No | -0.371 | 0.507 | -0.953 | -2.755 | No | No | No | No | No | No | No | 0.266 | No | No | -0.046 | No | No | 2.034 | 1.377 | No |
| CHEMBL1516410 | CC(=O)SC[C@@H](Cc1ccccc1)C(=O)NCC(=O)OCc1ccccc1 | 385.485 | 2.9846 | 9 | 5 | 1 | 163.044 | -5.082 | 0.982 | 94.112 | -2.734 | No | Yes | Yes | -0.179 | 0 | -0.533 | -2.677 | No | Yes | Yes | Yes | Yes | Yes | Yes | 0.075 | No | No | 0.465 | No | Yes | 2.038 | 1.585 | No |
| CHEMBL2170427 | O=C(O)c1ccc(Nc2nccc(Nc3ccccc3F)n2)cc1 | 324.315 | 3.8011 | 5 | 5 | 3 | 136.571 | -3.475 | 0.818 | 90.317 | -2.731 | Yes | No | No | -0.269 | 0.136 | -1.332 | -2.333 | No | No | Yes | No | No | No | No | -0.192 | No | No | 0.916 | No | No | 2.159 | 1.141 | No |
| CHEMBL3644455 | CC(C)(O)Cn1cc2c(n1)CN([C@H]1CSC(c3cc(F)cc(F)c3F)[C@@H](N)C1)C2 | 426.508 | 2.961 | 4 | 6 | 2 | 171.612 | -2.619 | 1.077 | 89.805 | -2.741 | Yes | No | No | 0.947 | 0.657 | 0.325 | -3.008 | No | No | No | No | No | No | No | 0.655 | No | No | -0.669 | No | No | 2.997 | 0.291 | Yes |
| CHEMBL1929396 | Cc1cc2ncc(C(=O)NCC(C)(C)NCC(=O)N3CCC[C@H]3C#N)cn2n1 | 383.456 | 0.6503 | 6 | 7 | 2 | 163.271 | -3.661 | 0.159 | 68.256 | -2.99 | Yes | No | No | 0.19 | 0.507 | -0.918 | -3.204 | No | No | No | No | No | No | No | 0.871 | No | No | 0.049 | No | No | 2.403 | 1.474 | Yes |
| CHEMBL2147777 | Cc1cc(N2CCN([C@@H]3CN[C@H](C(=O)N4CCSC4)C3)CC2)n(-c2ccccc2)n1 | 426.59 | 1.56612 | 4 | 7 | 1 | 181.936 | -3.092 | 1.03 | 97.465 | -2.887 | Yes | No | No | 1.366 | 0.408 | 0.174 | -2.61 | No | Yes | No | No | No | No | No | 0.621 | No | No | -0.93 | No | Yes | 2.43 | 1.206 | Yes |
| CHEMBL1096380 | CC(C)(C)c1[nH]cnc1/C=c1\[nH]c(=O)/c(=C/c2ccccc2)[nH]c1=O | 336.395 | 0.7414 | 2 | 3 | 3 | 143.547 | -2.819 | -0.249 | 75.2 | -2.735 | Yes | No | No | 0.219 | 0.009 | -0.614 | -2.593 | No | No | Yes | No | No | Yes | No | 0.437 | Yes | Yes | 0.346 | No | Yes | 2.626 | 1.489 | Yes |
| CHEMBL3707347 | CC(C)(Sc1ccncc1-c1ccc(C#N)c2ccccc12)C(=O)O | 348.427 | 4.72878 | 4 | 4 | 1 | 149.686 | -2.756 | 0.6 | 100 | -2.735 | Yes | No | No | -2.018 | 0.102 | -0.438 | -1.857 | No | No | No | No | Yes | No | No | 0.219 | No | No | 0.778 | No | No | 2.218 | 1.363 | Yes |
| CHEMBL3137318 | Cc1ccc(S(=O)(=O)O)cc1.Cn1ncnc1[C@H]1c2n[nH]c(=O)c3cc(F)cc(c23)N[C@@H]1c1ccc(F)cc1 | 552.563 | 3.87022 | 3 | 8 | 3 | 220.65 | -2.898 | -0.473 | 54.928 | -2.735 | No | No | No | -0.773 | 0.302 | -2.052 | -3.832 | No | No | No | No | No | No | No | -0.212 | No | No | 0.487 | No | No | 2.42 | 2.455 | Yes |
| CHEMBL276711 | Cc1cc(C)c(/C=C2\C(=O)Nc3ccccc32)[nH]1 | 238.29 | 3.12424 | 1 | 1 | 2 | 105.538 | -3.57 | 1.334 | 93.854 | -3.303 | Yes | No | No | 0.491 | 0.174 | 0.317 | -1.746 | No | Yes | Yes | Yes | No | No | No | 0.341 | No | No | -0.054 | No | No | 2.312 | 1.355 | No |
| CHEMBL1492500 | CN(C)CC/C=C1\c2ccccc2CSc2ccccc21 | 295.451 | 4.6757 | 3 | 2 | 0 | 131.231 | -4.689 | 1.458 | 93.117 | -2.141 | Yes | Yes | Yes | 0.77 | 0.124 | 0.904 | -1.371 | Yes | Yes | No | No | No | Yes | No | 0.726 | Yes | No | 0.612 | No | No | 1.858 | 1.254 | Yes |
| CHEMBL3989959 | NCCONC(=O)[C@@H]1CC[C@@H]2CN1C(=O)N2OS(=O)(=O)O | 324.315 | -2.0041 | 6 | 7 | 3 | 120.352 | -2.217 | -0.527 | 5.974 | -2.735 | Yes | No | No | -0.625 | 0.861 | -1.222 | -3.497 | No | No | No | No | No | No | No | 0.708 | No | No | 1.366 | No | No | 1.607 | 2.657 | No |
| CHEMBL1628227 | CN(C)CCC=C1c2ccccc2COc2ccccc21 | 279.383 | 3.9624 | 3 | 2 | 0 | 126.221 | -4.49 | 1.458 | 98.188 | -2.463 | Yes | Yes | No | 1.456 | 0.041 | 0.81 | -1.271 | No | Yes | Yes | No | No | Yes | No | 0.648 | No | No | 0.149 | No | Yes | 2.894 | 1.213 | Yes |
| CHEMBL512351 | COc1ccc(NC(=O)c2ccc(C(=N)N(C)C)cc2)c(C(=O)Nc2ccc(Cl)cn2)c1 | 451.914 | 4.13517 | 6 | 5 | 3 | 190.24 | -4.152 | 1.381 | 78.615 | -2.744 | Yes | Yes | Yes | -0.079 | 0 | -1.417 | -2.47 | No | Yes | No | Yes | Yes | No | Yes | 0.26 | No | No | 0.465 | No | Yes | 2.541 | 1.3 | Yes |
| CHEMBL471498 | O=C(O)[C@H](CC(=O)N1C[C@H]2CCCC[C@H]2C1)Cc1ccccc1 | 315.413 | 2.9686 | 5 | 2 | 1 | 137.041 | -3.681 | 1.381 | 93.826 | -2.755 | Yes | No | No | -0.485 | 0.102 | 0.064 | -2.163 | No | Yes | No | No | No | No | No | 0.347 | No | No | -1.322 | No | No | 2.906 | 1.712 | No |
| CHEMBL4297582 | O=C(CCCCCCC(=O)Nc1ccc(CN[C@H](C(=O)OC2CCCC2)c2ccccc2)cc1)NO | 495.62 | 4.7878 | 14 | 6 | 4 | 212.318 | -4.637 | 0.437 | 76.933 | -2.746 | Yes | Yes | Yes | 0.533 | 0.106 | -1.227 | -2.98 | No | Yes | No | Yes | No | Yes | Yes | 1.363 | No | No | -0.698 | No | Yes | 2.735 | 2.467 | Yes |
| CHEMBL396778 | C[C@H](NCc1ccc(OCc2cccc(F)c2)cc1)C(N)=O | 302.349 | 2.3681 | 7 | 3 | 2 | 128.644 | -3.36 | 0.987 | 94.034 | -2.881 | Yes | No | No | 0.519 | 0.134 | -0.26 | -2.707 | No | Yes | Yes | Yes | No | No | No | 1.014 | No | Yes | 0.493 | No | Yes | 2.531 | 1.931 | No |
| CHEMBL1201314 | CC(C)[C@H](N)C(=O)OCC(CO)OCn1cnc2c(=O)[nH]c(N)nc21 | 354.367 | -1.4367 | 8 | 10 | 4 | 142.5 | -2.602 | 0.154 | 58.788 | -2.735 | Yes | No | No | -0.254 | 0.747 | -1.67 | -4.06 | No | No | No | No | No | No | No | 0.814 | No | No | 0.089 | No | No | 2.345 | 2.177 | Yes |
| CHEMBL4554795 | COc1nc(N[C@H]2CC[C@H](N)CC2)c(F)cc1C(N)=O | 282.319 | 1.01 | 4 | 5 | 3 | 116.235 | -2.993 | 0.122 | 72.083 | -3.058 | No | No | No | 0.562 | 0.666 | -0.832 | -3.176 | No | No | No | No | No | No | No | 0.771 | No | No | 0.245 | No | No | 1.869 | 1.618 | Yes |
| CHEMBL193093 | NC(=O)c1sc(-c2ccsc2)cc1N | 224.31 | 2.1577 | 2 | 4 | 2 | 88.975 | -2.583 | 0.85 | 88.694 | -2.903 | Yes | No | No | 0.183 | 0.165 | -0.176 | -2.257 | No | No | Yes | No | No | No | No | -0.051 | No | Yes | 0.337 | No | No | 2.434 | 1.312 | No |
| CHEMBL2105776 | C[C@H](CSC(=O)c1ccccc1)C(=O)N1C[C@@H](Sc2ccccc2)C[C@H]1C(=O)[O-].C[C@H](CSC(=O)c1ccccc1)C(=O)N1C[C@@H](Sc2ccccc2)C[C@H]1C(=O)[O-].[Ca+2] | 897.188 | 5.0348 | 14 | 12 | 0 | 391.581 | -2.934 | 0.048 | 28.568 | -2.735 | Yes | No | No | -1.345 | 0.104 | -1.218 | -3.051 | No | Yes | No | No | No | No | No | 0.26 | No | No | 0.481 | No | No | 2.485 | 0.655 | No |
| CHEMBL144613 | CC(=O)Nc1cc(S(=O)(=O)O)cc2cc(S(=O)(=O)O)cc(O)c12 | 361.353 | 0.9972 | 3 | 6 | 4 | 130.606 | -2.895 | -0.425 | 17.785 | -2.735 | Yes | No | No | 0.001 | 0.31 | -1.321 | -3.769 | No | No | No | No | No | No | No | 0.603 | No | No | 0.829 | No | No | 2.361 | 2.695 | Yes |
| CHEMBL3651710 | COc1cc(-n2ccc(-c3ccc(OC(F)(F)F)cc3)cc2=O)ccc1OCCO | 421.371 | 3.7828 | 7 | 6 | 1 | 169.228 | -4.917 | 1.022 | 96.461 | -2.71 | Yes | Yes | Yes | 0.091 | 0.218 | -1.225 | -2.496 | No | Yes | Yes | Yes | Yes | No | Yes | 0.222 | No | No | 0.409 | No | No | 2.433 | 0.665 | Yes |
| CHEMBL519123 | O=C(Nc1ccc(-c2cn[nH]c2)cc1)C1COc2ccccc2O1 | 321.336 | 2.8552 | 3 | 4 | 2 | 137.97 | -3.64 | 0.933 | 90.077 | -2.803 | Yes | No | No | -0.291 | 0.021 | -0.984 | -2.246 | Yes | Yes | Yes | Yes | Yes | No | Yes | 0.082 | No | No | -0.021 | No | Yes | 2.359 | 1.536 | Yes |
| CHEMBL4303198 | O=C(CCCS)NC1CCN(C(=O)COc2ccc(Cl)cc2Cl)CC1 | 405.347 | 3.1894 | 7 | 4 | 2 | 162.948 | -4.451 | 1.2 | 87.298 | -3.43 | Yes | No | No | -0.27 | 0.174 | 0.053 | -2.707 | No | Yes | No | No | No | No | No | 0.041 | No | No | 0.519 | No | Yes | 3.269 | 1.267 | No |
| CHEMBL138029 | CCOC(=O)[C@H](CCc1ccccc1)N[C@@H](C)C(=O)N1N=C(C(C)(C)C)S[C@H]1C(=O)O | 449.573 | 2.8747 | 9 | 7 | 2 | 186.955 | -3.081 | 0.098 | 49.644 | -2.735 | Yes | No | No | -0.401 | 0.59 | -0.726 | -2.86 | No | Yes | No | No | No | No | No | 0.179 | No | No | 0.117 | No | No | 2.332 | 2.054 | Yes |
| CHEMBL2103833 | Cc1[nH]c(/C=C2\C(=O)Nc3ccc(F)cc32)c(C)c1C(=O)NCCN1CCCC1.O=P(O)(O)O | 494.46 | 2.16034 | 5 | 4 | 6 | 195.662 | -3.205 | 0.219 | 59.862 | -2.739 | Yes | No | No | -0.286 | 0.238 | -1.803 | -3.765 | No | Yes | No | No | No | No | No | 0.886 | No | No | 0.078 | No | Yes | 2.255 | 2.949 | Yes |
| CHEMBL2105727 | CN(C)Cc1c(C(=O)NCCOc2ccc(C(=O)NO)cc2)oc2ccccc12.Cl | 433.892 | 2.844 | 8 | 6 | 3 | 179.717 | -4.808 | 0.777 | 74.64 | -2.776 | Yes | Yes | No | 0.143 | 0.06 | -1.114 | -2.906 | No | Yes | No | Yes | No | No | Yes | 0.948 | No | No | 0.071 | No | Yes | 2.714 | 1.999 | Yes |
| CHEMBL3661406 | OC(c1ccccc1)C1OC1c1ccc(-c2ccccc2)cc1 | 302.373 | 4.5271 | 4 | 2 | 1 | 135.542 | -5.982 | 1.71 | 93.825 | -2.701 | Yes | Yes | Yes | 0.243 | 0.077 | 0.296 | -1.475 | No | Yes | Yes | Yes | Yes | No | No | 0.119 | No | No | 0.529 | No | Yes | 2.082 | 2.092 | Yes |
| CHEMBL3644459 | NC(=O)c1[nH]nc2c1CN([C@H]1CSC(c3cc(F)ccc3F)[C@@H](N)C1)C2 | 379.436 | 1.6765 | 3 | 5 | 3 | 152.849 | -2.556 | 0.19 | 70.155 | -2.735 | Yes | No | No | 0.915 | 0.587 | -1.048 | -2.876 | No | No | No | No | No | No | No | 0.582 | No | No | -0.097 | No | No | 2.874 | 1.269 | Yes |
| CHEMBL3644457 | CC(C)(F)Cn1cc2c(n1)CN([C@H]1CSC(c3cc(F)cc(F)c3F)[C@@H](N)C1)C2 | 428.499 | 3.9382 | 4 | 5 | 1 | 170.983 | -2.808 | 1.178 | 89.181 | -2.743 | Yes | No | No | 0.941 | 0.619 | 0.543 | -2.09 | No | No | No | No | No | No | Yes | 0.615 | No | No | -0.686 | No | Yes | 3.199 | 0.124 | Yes |
| CHEMBL3661417 | O=C(c1ccccc1)C1OC1c1ccc(-c2ccccc2)cc1 | 300.357 | 4.6764 | 4 | 2 | 0 | 134.91 | -6.322 | 1.725 | 96.484 | -2.7 | Yes | Yes | Yes | 0.24 | 0.077 | 0.45 | -1.207 | No | Yes | Yes | Yes | Yes | No | No | 0.102 | No | No | 0.514 | No | Yes | 2.066 | 2.088 | Yes |
| CHEMBL318779 | CSCC[C@@H](C=O)NC(=O)[C@H](CC(C)C)NC(=O)[C@H](CC(C)C)NC(C)=O | 401.573 | 1.5049 | 13 | 5 | 3 | 166.727 | -2.859 | -0.036 | 55.349 | -2.946 | Yes | Yes | No | -0.585 | 0.377 | -0.877 | -3.445 | No | No | No | No | No | No | No | 0.247 | No | No | 0.205 | No | No | 2.649 | 1.041 | No |
| CHEMBL536282 | Cl.NCc1cnc(S)n1[C@H]1CCc2c(F)cc(F)cc2C1 | 331.819 | 3.0605 | 2 | 4 | 2 | 132.925 | -2.591 | 1.281 | 90.259 | -2.762 | Yes | No | No | 0.274 | 0.516 | 0.134 | -1.999 | No | No | Yes | No | No | No | No | 1.35 | No | No | -0.043 | No | No | 2.73 | 1.427 | Yes |
| CHEMBL430554 | NCCCC[C@H](N[C@H]1CCc2ccccc2N(CC(=O)O)C1=O)C(=O)O | 363.414 | 0.5908 | 9 | 5 | 4 | 151.527 | -2.725 | 0.225 | 17.673 | -2.735 | Yes | No | No | -0.667 | 0.779 | -1.024 | -3.539 | No | No | No | No | No | No | No | 0.416 | No | No | 0.542 | No | No | 2.101 | 2.283 | Yes |
| CHEMBL4447328 | C=CC(=O)N1CCC[C@@H](n2nc(-c3ccc(Oc4ccccc4)cc3)c(C(N)=O)c2N)C1 | 431.496 | 3.373 | 6 | 6 | 2 | 185.618 | -3.483 | 0.136 | 85.237 | -2.736 | Yes | Yes | Yes | -0.09 | 0 | -0.872 | -2.701 | No | Yes | No | Yes | Yes | No | Yes | 0.386 | No | Yes | 0.178 | No | Yes | 2.809 | 1.653 | Yes |
| CHEMBL4569451 | O=C(O)c1ccc(-c2cn(-c3cccnc3)nn2)cc1 | 266.26 | 2.0275 | 3 | 5 | 1 | 113.881 | -2.838 | 0.963 | 98.62 | -2.753 | No | No | No | -0.604 | 0.339 | -0.702 | -2.912 | No | No | No | No | No | No | No | 0.677 | No | Yes | -0.217 | No | No | 2.687 | 0.972 | Yes |
| CHEMBL4298140 | Cc1cc(Nc2cc(N)ncn2)c(=O)n2c1C(=O)NC21CCCCC1.Cl | 376.848 | 2.05462 | 2 | 7 | 3 | 156.181 | -4.677 | 0.613 | 85.498 | -2.807 | No | No | No | 0.029 | 0.428 | -1.194 | -3.134 | No | Yes | No | No | No | No | No | -0.689 | No | No | -0.126 | No | No | 2.65 | 0.427 | Yes |
| CHEMBL1201368 | CCC[C@H](N[C@@H](C)C(=O)N1[C@H](C(=O)O)C[C@@H]2CCCC[C@@H]21)C(=O)O | 340.42 | 1.4621 | 7 | 4 | 3 | 141.949 | -1.966 | -0.12 | 22.198 | -2.735 | Yes | No | No | -1.172 | 0.677 | -1.015 | -3.561 | No | No | No | No | No | No | No | 0.353 | No | No | 0.912 | No | No | 1.601 | 2.387 | Yes |
| CHEMBL3644462 | N[C@H]1C[C@@H](N2Cc3nc(-c4ccccc4)[nH]c3C2)CSC1c1cc(F)ccc1F | 412.509 | 4.2446 | 3 | 4 | 2 | 172.071 | -2.943 | 0.357 | 82.759 | -2.735 | Yes | Yes | Yes | 0.328 | 0.081 | 0.445 | -1.872 | No | Yes | Yes | No | Yes | Yes | Yes | 0.638 | Yes | Yes | -0.055 | Yes | Yes | 2.476 | 0.65 | Yes |
| CHEMBL409450 | O=C1Nc2cc(Br)ccc2/C1=C1/Nc2ccccc2/C1=N/O | 356.179 | 3.4163 | 0 | 4 | 3 | 134.019 | -3.788 | 0.811 | 90.66 | -2.751 | Yes | No | No | -0.362 | 0.124 | -0.962 | -1.868 | Yes | Yes | Yes | Yes | Yes | Yes | No | 0.122 | Yes | No | 0.476 | No | Yes | 2.44 | 1.458 | No |
| CHEMBL96862 | O=P(O)(O)OC[C@H]1O[C@@H](n2cnc3cncnc32)[C@H](O)[C@@H]1O | 332.209 | -1.4452 | 4 | 9 | 4 | 122.964 | -2.418 | -0.251 | 62.483 | -2.735 | Yes | No | No | 0.545 | 0.787 | -1.979 | -4.328 | No | No | No | No | No | No | No | 0.458 | No | No | 0.419 | No | No | 2.042 | 3.196 | No |
| CHEMBL3661416 | O=C(c1ccccc1)C1OC1c1ccc(F)cc1 | 242.249 | 3.1485 | 3 | 2 | 0 | 104.018 | -3.855 | 1.454 | 96.397 | -2.685 | No | No | No | 0.124 | 0.103 | 0.292 | -1.523 | No | Yes | Yes | Yes | No | No | No | 0.001 | No | Yes | 0.446 | No | No | 1.711 | 2.058 | No |
| CHEMBL28636 | COc1ccc(-c2cc(C(F)F)nn2-c2ccc(S(N)(=O)=O)cc2)cc1F | 397.378 | 3.272 | 5 | 5 | 1 | 152.594 | -4.495 | 1.236 | 92.839 | -2.765 | No | Yes | Yes | -0.401 | 0.152 | -1.346 | -2.508 | No | Yes | Yes | Yes | Yes | No | Yes | 0.769 | No | No | 0.426 | No | Yes | 2.518 | 0.278 | Yes |
| CHEMBL4594270 | CC(C)(NC(=O)O[C@@H]1CN2CCC1CC2)c1csc(-c2ccc(F)cc2)n1.O=C(O)CC(O)C(=O)O | 523.583 | 2.9113 | 7 | 8 | 4 | 211.273 | -2.756 | -0.572 | 22.296 | -2.735 | Yes | No | No | -1.509 | 0.376 | -1.997 | -4.041 | No | No | No | No | No | No | No | 0.47 | No | No | 0.902 | No | No | 1.851 | 2.465 | Yes |
| CHEMBL3661402 | O=NC(c1ccccc1)C1OC1c1ccccc1 | 239.274 | 3.6342 | 4 | 3 | 0 | 105.386 | -3.988 | 1.556 | 94.762 | -2.717 | Yes | No | No | 0.412 | 0.024 | 0.189 | -1.526 | No | Yes | Yes | Yes | Yes | No | No | 0.2 | Yes | Yes | 0.442 | No | No | 1.767 | 0.85 | No |
| CHEMBL3646118 | O=c1c(-n2ccnn2)c[nH]n1-c1cc(N2CCOCC2)ncn1 | 314.309 | -0.6271 | 3 | 9 | 1 | 129.876 | -3.475 | 0.205 | 77.204 | -2.753 | No | No | No | -0.159 | 0.445 | -1.284 | -3.644 | No | No | No | No | No | No | No | 0.491 | No | No | 0.114 | No | No | 2.193 | -0.324 | Yes |
| CHEMBL2397013 | CS(=O)(=O)O.O=C1Nc2ccc(-c3cccnc3)cc2/C1=C/c1ccc[nH]1 | 383.429 | 3.0734 | 2 | 4 | 3 | 156.085 | -3.233 | 0.309 | 50.705 | -2.735 | Yes | No | No | -0.515 | 0.228 | -1.139 | -2.139 | No | Yes | Yes | No | No | No | No | 0.124 | No | No | 0.5 | No | No | 2.482 | 2.253 | Yes |
| CHEMBL3640070 | CC(C)C[C@H](NC(=O)c1ccc(C(C)(C)C)cc1)C(=O)N1CC[C@H]2OCC(=O)[C@H]21 | 400.519 | 2.6974 | 5 | 4 | 1 | 172.532 | -5.028 | 1.1 | 93.914 | -3.53 | Yes | Yes | Yes | -0.084 | 0.042 | -0.208 | -2.461 | No | Yes | No | No | No | No | Yes | 0.682 | No | No | -0.365 | No | No | 2.58 | 1.728 | Yes |
| CHEMBL3661411 | Cc1cccc(C(O)C2OC2c2ccc(-c3ccccc3)cc2)c1 | 316.4 | 4.83552 | 4 | 2 | 1 | 141.907 | -5.806 | 1.726 | 93.152 | -2.732 | Yes | Yes | Yes | -0.055 | 0.007 | 0.528 | -1.011 | No | Yes | Yes | Yes | Yes | No | Yes | 0.143 | No | Yes | 0.595 | No | Yes | 2.047 | 2.06 | No |
| CHEMBL2106525 | CO/N=C/[C@@H]1[C@@H](c2ccc(Cl)c(Cl)c2)C[C@@H]2CC[C@H]1N2C.O=C(O)/C=C\C(=O)O | 443.327 | 3.9037 | 5 | 5 | 2 | 179.086 | -2.593 | 0.561 | 40.091 | -2.735 | Yes | No | No | -0.536 | 0.521 | -1.171 | -2.387 | No | No | No | No | No | No | No | -0.092 | No | No | 0.545 | No | No | 2.124 | 0.904 | Yes |
| CHEMBL531048 | O=C1C(=O)c2ncccc2-c2cccnc21 | 210.192 | 1.5226 | 0 | 4 | 0 | 90.975 | -1.973 | 1.249 | 100 | -2.553 | No | No | No | -0.303 | 0.32 | -0.281 | -2.922 | No | No | Yes | No | No | No | No | 0.182 | No | No | 0.176 | No | No | 2.592 | 2.928 | Yes |
| CHEMBL535834 | Cl.Cl.Nc1ccc(SC[C@@H]2CO[C@](CCc3ccc(Cl)cc3)(Cn3ccnc3)O2)cc1 | 502.895 | 5.4991 | 8 | 6 | 1 | 204.033 | -3.133 | 0.517 | 86.082 | -2.736 | Yes | Yes | Yes | 0.276 | 0 | -0.189 | -2.124 | No | Yes | No | Yes | Yes | No | Yes | 1.635 | Yes | No | 0.808 | No | Yes | 2.574 | 0.81 | No |
| CHEMBL1877495 | CC(NNC(=O)c1ccccc1)c1ccccc1 | 240.306 | 2.6822 | 4 | 2 | 2 | 106.815 | -3.05 | 1.358 | 90.995 | -2.74 | Yes | No | No | -0.042 | 0.048 | 0.33 | -0.489 | Yes | Yes | Yes | Yes | No | No | No | 0.765 | No | No | 0.357 | No | No | 2.421 | 2.271 | No |
| CHEMBL1231461 | Br | 80.912 | 0.5779 | 0 | 0 | 0 | 17.881 | -0.323 | 1.38 | 100 | -2.497 | Yes | No | No | -0.041 | 0.762 | 0.026 | -2.298 | No | No | No | No | No | No | No | 0.131 | No | No | 1.2 | No | No | 2.401 | 1.412 | No |
| CHEMBL3186534 | N#CC1(c2ccc(NC(=O)c3cccnc3NCc3ccncc3)cc2)CCCC1 | 397.482 | 4.67638 | 6 | 5 | 2 | 175.552 | -4.296 | 1.139 | 93.954 | -2.812 | Yes | Yes | Yes | 0.445 | 0 | 0.046 | -2.148 | No | Yes | Yes | Yes | Yes | No | Yes | 0.371 | No | No | -0.282 | No | Yes | 2.651 | 0.586 | Yes |
| CHEMBL4570801 | C=CC(=O)N1CCN(c2ncnc3c(F)c(-c4cc(O)cc5ccccc45)c(Cl)cc23)CC1 | 462.912 | 4.7827 | 3 | 5 | 1 | 194.095 | -3.783 | 1.108 | 94.79 | -2.735 | Yes | Yes | Yes | -0.88 | 0.296 | -0.491 | -2.125 | No | Yes | No | Yes | Yes | No | No | 0.214 | No | No | 0.387 | No | Yes | 2.953 | 0.25 | Yes |
| CHEMBL13608 | Cc1[nH]c(/C=C2\C(=O)Nc3ccc(F)cc32)c(C)c1C(=O)NCCN1CCCC1 | 396.466 | 3.08894 | 5 | 3 | 3 | 168.724 | -3.5 | 1.005 | 93.358 | -2.852 | Yes | Yes | No | 1.128 | 0.152 | -0.734 | -2.424 | No | Yes | No | No | No | No | No | 1.161 | Yes | No | -0.582 | No | Yes | 2.233 | 2.17 | Yes |
| CHEMBL9298 | N#Cc1ccc(C2CCCc3cncn32)cc1 | 223.279 | 2.68048 | 1 | 3 | 0 | 100.339 | -2.367 | 1.649 | 97.229 | -2.936 | Yes | No | No | 0.43 | 0.268 | 0.399 | -1.759 | No | No | Yes | Yes | No | Yes | No | 0.98 | No | Yes | 0.33 | No | No | 2.399 | 0.872 | No |
| CHEMBL3666065 | COC(=O)c1ccc(/N=C/C2C(=O)Nc3ccccc32)cc1 | 294.31 | 2.9114 | 3 | 4 | 1 | 127.189 | -4.072 | 1.238 | 94.039 | -3.018 | No | No | No | -0.335 | 0 | -0.112 | -2.111 | No | Yes | Yes | Yes | No | No | No | 0.205 | No | Yes | 0.302 | No | Yes | 2.024 | 1.851 | No |
| CHEMBL2105901 | CCC(=O)N1C2CCC1CN(C/C=C\c1ccccc1)C2 | 284.403 | 2.785 | 4 | 2 | 0 | 126.791 | -3.207 | 1.51 | 93.196 | -2.61 | Yes | Yes | No | 0.744 | 0.385 | 0.734 | -2.546 | No | No | No | Yes | No | Yes | No | 0.859 | Yes | No | 0.323 | No | Yes | 1.981 | 0.786 | Yes |
| CHEMBL3707394 | CC(=O)N[C@H]1CC(=O)N(c2ccc(OCc3cccc(F)c3)cc2)C1 | 342.37 | 2.6461 | 5 | 3 | 1 | 144.949 | -4.377 | 1.327 | 95.56 | -2.77 | No | Yes | No | -0.211 | 0.066 | -0.418 | -2.468 | No | Yes | No | Yes | Yes | No | No | 0.274 | No | Yes | 0.309 | No | Yes | 2.181 | 1.609 | Yes |
| CHEMBL3644453 | N[C@H]1C[C@@H](N2Cc3c[nH]nc3C2)C[S+]([O-])C1c1cc(F)cc(F)c1F | 370.4 | 1.7322 | 2 | 4 | 2 | 145.119 | -3.205 | 0.503 | 91.375 | -3.551 | Yes | No | No | 0.843 | 0.45 | -1.148 | -3.368 | No | Yes | No | No | No | No | No | 0.46 | No | No | -0.461 | No | No | 2.718 | 1.043 | Yes |
| CHEMBL325695 | CC(=O)SC[C@@H](Cc1ccc2c(c1)OCO2)C(=O)N[C@@H](C)C(=O)OCc1ccccc1 | 443.521 | 3.1018 | 9 | 7 | 1 | 184.995 | -5.49 | 1.044 | 93.839 | -2.833 | No | Yes | Yes | -0.373 | 0 | -0.919 | -3.16 | No | Yes | No | Yes | Yes | No | Yes | -0.159 | No | No | 0.119 | No | No | 2.367 | 1.538 | Yes |
| CHEMBL1733 | C[C@H](N[C@@H](CCc1ccccc1)C(=O)O)C(=O)N1Cc2ccccc2C[C@H]1C(=O)O | 410.47 | 2.0886 | 8 | 4 | 3 | 174.88 | -2.893 | 0.655 | 27.455 | -2.735 | Yes | No | No | -2.406 | 0.191 | -0.812 | -2.953 | No | No | No | No | No | No | No | 0.524 | No | No | 0.787 | No | No | 2.119 | 3.098 | Yes |
| CHEMBL258004 | c1cc(NC2CCNCC2)nc(-c2cnc3ccccn23)c1 | 293.374 | 2.5602 | 3 | 5 | 2 | 128.965 | -2.365 | 1.252 | 97.265 | -2.735 | Yes | No | No | 0.345 | 0.378 | 0.222 | -2.95 | No | No | Yes | No | No | No | No | 0.884 | No | Yes | 0.433 | No | Yes | 2.549 | -0.114 | Yes |
| CHEMBL84446 | NC(=O)[C@@H]1C[C@]2(NC(=O)NC2=O)c2cc(F)ccc2O1 | 279.227 | -0.5032 | 1 | 4 | 3 | 111.813 | -2.028 | 0.576 | 68.806 | -3.365 | No | No | No | -0.388 | 0.371 | -0.89 | -3.148 | No | No | No | No | No | No | No | 0.373 | No | No | 0.61 | No | No | 2.251 | 1.783 | Yes |
| CHEMBL2105502 | COCCO[C@@H]1c2ccn3c(C)c(C)nc3c2N[C@H](c2ccccc2)[C@H]1O | 367.449 | 3.18294 | 5 | 6 | 2 | 158.311 | -2.969 | 0.952 | 94.292 | -2.735 | Yes | Yes | Yes | 1.285 | 0.303 | -0.588 | -2.482 | No | Yes | Yes | No | No | Yes | Yes | 1.05 | Yes | Yes | 0.193 | No | Yes | 2.794 | -0.415 | Yes |
| CHEMBL18 | CCOc1ccc2nc(S(N)(=O)=O)sc2c1 | 258.324 | 1.3424 | 3 | 5 | 1 | 96.917 | -2.775 | 1.333 | 94.01 | -2.804 | No | No | No | -0.479 | 0.261 | -0.698 | -3.047 | No | No | Yes | No | No | No | No | 0.19 | No | No | 0.707 | No | No | 2.565 | 0.907 | No |
| CHEMBL249097 | Cc1ccc(O)cc1Nc1ccnc(Nc2cccc(C(N)=O)c2)n1 | 335.367 | 3.07672 | 5 | 6 | 4 | 144.11 | -3.305 | 0.278 | 85.027 | -2.737 | Yes | No | No | 0.155 | 0.158 | -1.168 | -2.519 | No | No | Yes | Yes | Yes | No | Yes | -0.154 | No | No | 0.714 | No | Yes | 2.666 | 1.699 | Yes |
| CHEMBL3410535 | O=C1/C(=C/c2ccc([N+](=O)[O-])cc2)CS(=O)(=O)C/C1=C\c1ccc([N+](=O)[O-])cc1 | 414.395 | 2.9675 | 4 | 7 | 0 | 164.751 | -5.569 | -0.398 | 91.393 | -2.761 | No | Yes | Yes | -0.607 | 0 | -1.13 | -2.203 | No | Yes | No | Yes | Yes | No | Yes | 0.58 | No | No | -0.053 | No | No | 2.18 | 2.445 | No |
| CHEMBL1922282 | CS(=O)(=O)Oc1ccc2c(NC(N)=O)c(C(=O)c3ccc(Cl)cc3Cl)oc2c1 | 443.264 | 3.7996 | 5 | 6 | 2 | 168.827 | -4.752 | 0.37 | 86.497 | -2.742 | Yes | Yes | Yes | -0.599 | 0 | -1.089 | -2.599 | No | Yes | No | No | Yes | No | Yes | -0.091 | No | No | 0.127 | No | Yes | 2.3 | 0.718 | Yes |
| CHEMBL161702 | O=C(O)C(Cc1ccccc1)CN(O)C(=O)Cc1ccccc1 | 313.353 | 2.3904 | 7 | 3 | 2 | 134.304 | -3.848 | 1.019 | 93.992 | -2.741 | Yes | No | No | -0.562 | 0.04 | -0.041 | -2.582 | No | Yes | No | No | No | No | No | 0.575 | No | No | -0.073 | No | No | 2.178 | 1.484 | No |
| CHEMBL224325 | Cc1ccc2c(Cl)cc(Cl)c(O)c2n1 | 228.078 | 3.55562 | 0 | 2 | 1 | 91.098 | -3.918 | 1.367 | 92.268 | -2.42 | Yes | No | No | 0.253 | 0.358 | 0.543 | -2.312 | No | No | Yes | No | No | No | No | 0.26 | No | No | 0.9 | No | No | 2.354 | 1.077 | Yes |
| CHEMBL404609 | NC(=O)Nc1sc(-c2ccccc2)cc1C(N)=O | 261.306 | 2.0046 | 3 | 3 | 3 | 107.411 | -2.798 | 1.059 | 83.26 | -3.058 | Yes | No | No | -0.141 | 0.074 | -0.675 | -2.462 | No | Yes | Yes | No | No | No | No | -0.006 | No | No | 0.241 | No | No | 1.722 | 1.874 | No |
| CHEMBL185198 | C#[N+][O-] | 43.025 | 0.4469 | 0 | 1 | 0 | 18.09 | -0.041 | 1.351 | 100 | -2.838 | Yes | No | No | -0.146 | 0.76 | -0.078 | -2.426 | No | No | No | No | No | No | No | 0.635 | No | No | 1.202 | No | No | 2.629 | 1.634 | No |
| CHEMBL1098285 | CN(CCCCCCN(C)C(=O)Oc1ccc[n+](C)c1)C(=O)Oc1ccc[n+](C)c1.[Br-].[Br-] | 576.33 | -3.5346 | 9 | 4 | 0 | 210.822 | -2.712 | 1.586 | 86.257 | -2.742 | Yes | No | No | -0.308 | 0.576 | 0.235 | -3.273 | No | Yes | No | No | No | No | No | 0.875 | No | No | -0.111 | No | Yes | 2.453 | 0.958 | Yes |
| CHEMBL1199307 | CN(CCCCCCN(C)C(=O)Oc1ccc[n+](C)c1)C(=O)Oc1ccc[n+](C)c1 | 416.522 | 2.4574 | 9 | 4 | 0 | 177.797 | -3.411 | 1.299 | 93.778 | -2.741 | Yes | No | No | 0.077 | 0.462 | 0.262 | -3.087 | No | Yes | No | No | No | No | No | 0.531 | No | No | -0.201 | No | Yes | 3.113 | -0.024 | Yes |
| CHEMBL28196 | O=C(O)CCCC/C=C(/c1ccccc1)c1cccnc1 | 281.355 | 4.1583 | 7 | 2 | 1 | 124.529 | -3.854 | 1.516 | 94.252 | -2.612 | No | No | No | -0.335 | 0.14 | 0.018 | -2.124 | No | Yes | No | No | No | No | No | 0.48 | No | No | -0.232 | No | No | 2.487 | 2.144 | Yes |
| CHEMBL1788402 | O=S(=O)(O)c1cc(I)c(O)c2ncccc12 | 351.121 | 1.7917 | 1 | 4 | 2 | 105.017 | -2.62 | 1.027 | 93.671 | -2.735 | No | No | No | -0.602 | 0.254 | -0.691 | -3.08 | No | No | No | No | No | No | No | -0.133 | No | No | 1.163 | No | No | 2.391 | 2.212 | Yes |
| CHEMBL2104578 | O=C(O)[C@H](CCc1ccccc1)N[C@H]1CCCN2CCC[C@@H](C(=O)O)N2C1=O | 389.452 | 1.117 | 7 | 5 | 3 | 163.642 | -2.8 | 0.488 | 28.302 | -2.735 | Yes | No | No | -0.458 | 0.743 | -0.783 | -3.163 | No | Yes | No | No | No | No | No | 0.199 | No | No | -0.178 | No | No | 2.159 | 1.162 | Yes |
| CHEMBL2104660 | CC(=O)SC[C@H](Cc1ccccc1)C(=O)NCC(=O)OCc1ccccc1 | 385.485 | 2.9846 | 9 | 5 | 1 | 163.044 | -5.082 | 0.982 | 94.112 | -2.734 | No | Yes | Yes | -0.179 | 0 | -0.533 | -2.677 | No | Yes | Yes | Yes | Yes | Yes | Yes | 0.075 | No | No | 0.465 | No | Yes | 2.038 | 1.585 | No |
| CHEMBL3661410 | Cc1cccc(C(=O)C2OC2c2ccc(-c3ccccc3)cc2)c1 | 314.384 | 4.98482 | 4 | 2 | 0 | 141.275 | -6.215 | 1.756 | 95.665 | -2.716 | No | No | Yes | 0.108 | 0.052 | 0.43 | -1.195 | No | Yes | Yes | Yes | Yes | No | No | 0.135 | No | No | 0.681 | No | Yes | 2.084 | 2.095 | Yes |
| CHEMBL2105586 | O=C1CCC2NCCc3c2n1c1ccc(Cl)cc31 | 260.724 | 2.9155 | 0 | 3 | 1 | 109.725 | -3.419 | 1.424 | 93.776 | -2.627 | Yes | No | No | 0.989 | 0.307 | 0.379 | -2.116 | Yes | Yes | Yes | No | No | Yes | No | 1.066 | No | Yes | -0.489 | No | No | 2.773 | 1.347 | Yes |
| CHEMBL2107466 | COc1ccc(C(=C(C#N)CCC(=O)O)c2ccc(OC)cc2)cc1 | 337.375 | 3.89408 | 7 | 4 | 1 | 146.294 | -5.102 | 1.006 | 98.718 | -2.73 | No | No | No | -0.83 | 0 | -0.32 | -2.392 | No | Yes | No | No | No | No | No | 0.533 | No | No | 0.459 | No | No | 2.866 | 1.677 | No |
| CHEMBL2170597 | O=C(O)c1ccc(Nc2nccc(Nc3ccccc3)n2)cc1 | 306.325 | 3.662 | 5 | 5 | 3 | 132.405 | -3.384 | 0.763 | 91.082 | -2.728 | Yes | No | No | -0.237 | 0.152 | -1.112 | -2.286 | No | No | Yes | No | No | No | No | 0.034 | No | No | 0.827 | No | No | 2.246 | 1.197 | No |
| CHEMBL1200563 | COCc1c(C(C)C)nc(C(C)C)c(/C=C/[C@@H](O)C[C@@H](O)CC(=O)[O-])c1-c1ccc(F)cc1.[Na+] | 481.54 | 0.55 | 11 | 6 | 2 | 222.729 | -3.593 | 0.131 | 38.148 | -2.735 | Yes | No | No | -1.153 | 0.099 | -0.648 | -3.679 | No | Yes | No | No | Yes | No | No | 0.952 | No | No | -1.367 | No | No | 2.642 | 1.944 | No |
| CHEMBL435176 | CCOC(=O)COC1CCN(C(=O)[C@H](C)NC(=O)c2ccc(C(=N)NO)cc2)CC1 | 420.466 | 0.67977 | 8 | 7 | 4 | 174.54 | -2.662 | -0.053 | 54.377 | -2.854 | Yes | No | No | -0.907 | 0.34 | -1.59 | -4.368 | No | No | No | No | No | No | No | 0.48 | No | No | 0.74 | No | No | 2.994 | 2.081 | Yes |
| CHEMBL70611 | Nc1ccc(SC[C@@H]2CO[C@](CCc3ccc(Cl)cc3)(Cn3ccnc3)O2)cc1 | 429.973 | 4.6555 | 8 | 6 | 1 | 179.726 | -3.128 | 0.579 | 88.158 | -2.736 | Yes | Yes | Yes | 0.314 | 0 | -0.182 | -2.257 | No | Yes | Yes | Yes | Yes | No | Yes | 1.359 | Yes | No | 0.795 | No | Yes | 2.573 | 1.204 | No |
| CHEMBL4298173 | CN1C/C=C/CCOc2cccc(c2)-c2ccnc(n2)Nc2cccc(c2)C1.O=C(O)CC(O)(CC(=O)O)C(=O)O | 564.595 | 3.4092 | 5 | 9 | 5 | 236.124 | -2.886 | -0.949 | 0 | -2.735 | Yes | No | No | -0.331 | 0.321 | -1.941 | -3.582 | No | No | No | No | No | No | No | -0.137 | No | No | 0.309 | No | No | 2.464 | 3.254 | Yes |
| CHEMBL4297623 | NC(=O)/C(=C/n1cnc(-c2cc(C(F)(F)F)cc(C(F)(F)F)c2)n1)c1cncnc1 | 428.296 | 3.2561 | 4 | 6 | 1 | 163.442 | -3.933 | 1.197 | 90.148 | -2.788 | No | Yes | No | -0.658 | 0.082 | -1.819 | -3.132 | No | Yes | Yes | Yes | Yes | No | No | 0.654 | No | No | 0.073 | No | No | 2.428 | 0.721 | Yes |
| CHEMBL1733373 | O=C(c1ccccc1)C1OC1c1ccccc1 | 224.259 | 3.0094 | 3 | 2 | 0 | 99.853 | -3.413 | 1.699 | 96.757 | -2.668 | Yes | No | No | 0.297 | 0.058 | 0.271 | -1.499 | No | Yes | Yes | Yes | No | No | No | 0.113 | Yes | Yes | 0.438 | No | No | 1.659 | 2.158 | No |
| CHEMBL491510 | C[C@H]1CCC[C@@H](O)CCCCCc2cc(O)cc(O)c2C(=O)O1 | 322.401 | 3.2908 | 0 | 5 | 3 | 136.463 | -3.421 | 1.267 | 89.694 | -3.543 | Yes | No | No | 0.028 | 0.366 | -1.04 | -3.206 | No | No | No | No | No | No | Yes | 0.992 | No | No | -0.467 | No | No | 2.372 | 1.582 | No |
| CHEMBL4594293 | CC#CC(=O)N1CCC[C@H]1c1nc(-c2ccc(C(=O)Nc3ccccn3)cc2)c2c(N)nccn12.O=C(O)/C=C\C(=O)O | 581.589 | 3.0244 | 6 | 9 | 4 | 245.261 | -2.894 | -0.364 | 15.408 | -2.735 | Yes | No | No | 0.222 | 0.292 | -1.706 | -3.332 | No | No | No | No | No | No | No | -0.207 | No | No | 0.476 | No | No | 2.477 | 1.784 | No |
| CHEMBL257167 | NC(=O)Nc1sc(-c2ccc(F)cc2)cc1C(N)=O | 279.296 | 2.1437 | 3 | 3 | 3 | 111.577 | -3.193 | 1.02 | 83.817 | -3.041 | Yes | No | No | -0.199 | 0.079 | -0.877 | -2.513 | No | Yes | Yes | No | No | No | No | -0.156 | No | No | 0.266 | No | No | 1.723 | 1.894 | No |
| CHEMBL356301 | CN1Cc2cc(C(=O)N3CCC(C4CCNCC4)CC3)ccc2N[C@@H](CC(=O)O)C1=O | 428.533 | 1.7656 | 4 | 5 | 3 | 182.516 | -3.218 | 0.221 | 49.492 | -2.735 | Yes | No | No | -0.216 | 0.565 | -0.605 | -3.076 | No | Yes | No | No | No | No | No | 0.371 | No | No | 0.335 | No | No | 2.12 | 1.571 | Yes |
| CHEMBL468419 | Cl.N#Cc1ccc(C2CCCc3cncn32)cc1 | 259.74 | 3.10228 | 1 | 3 | 0 | 112.492 | -2.415 | 1.659 | 96.191 | -2.936 | Yes | No | No | 0.421 | 0.256 | 0.395 | -1.745 | No | No | Yes | Yes | No | Yes | No | 1.338 | Yes | Yes | 0.359 | No | No | 2.456 | 0.718 | No |
| CHEMBL2364622 | Cl.NS(=O)(=O)OC[C@@H]1C[C@@H](n2ccc3c(N[C@H]4CCc5ccccc54)ncnc32)C[C@@H]1O | 479.99 | 2.4846 | 6 | 8 | 3 | 192.222 | -3.807 | 0.382 | 88.718 | -2.835 | Yes | Yes | Yes | -0.269 | 0.162 | -1.52 | -3.494 | No | Yes | No | No | No | No | Yes | -0.123 | No | No | -0.503 | No | No | 2.619 | 1.346 | Yes |
| CHEMBL17331 | O=[N+]([O-])c1ccc(-c2nc(-c3ccc(F)cc3)c(-c3ccncc3)[nH]2)cc1 | 360.348 | 4.853 | 4 | 4 | 1 | 153.07 | -2.915 | 0.986 | 87.581 | -2.735 | Yes | Yes | Yes | -0.215 | 0.196 | -0.007 | -1.978 | No | Yes | Yes | Yes | Yes | No | Yes | 0.453 | Yes | Yes | 0.15 | No | Yes | 2.441 | 1.137 | No |
| CHEMBL296602 | CNC1CC(c2ccc(Cl)c(Cl)c2)c2ccccc21 | 292.209 | 4.7895 | 2 | 1 | 1 | 123.099 | -4.862 | 1.422 | 93.258 | -2.603 | Yes | Yes | No | 1.492 | 0.059 | 0.663 | -1.135 | No | Yes | Yes | Yes | No | Yes | Yes | 0.961 | No | No | 0.183 | No | Yes | 2.889 | 0.928 | Yes |
| CHEMBL1084955 | Nc1c(C(=O)Nc2cccc(F)c2)sc2nc(-c3cccs3)ccc12 | 369.446 | 4.9984 | 3 | 5 | 2 | 150.309 | -4.746 | 0.999 | 88.814 | -2.741 | Yes | Yes | Yes | -0.039 | 0.117 | -0.458 | -1.806 | No | Yes | Yes | Yes | Yes | No | Yes | -0.018 | No | Yes | 0.578 | No | Yes | 2.698 | 0.73 | Yes |
| CHEMBL2104358 | COc1ccc(C(C)(C)NC(=O)[C@H]2CC[C@H]3[C@@H]4CC[C@H]5NC(=O)C=C[C@]5(C)[C@H]4CC[C@]23C)cc1 | 464.65 | 4.9599 | 4 | 3 | 2 | 203.648 | -4.84 | 0.944 | 94.092 | -2.957 | Yes | Yes | Yes | 0.037 | 0 | -0.256 | -1.685 | No | Yes | No | No | Yes | No | Yes | 0.292 | No | No | -0.41 | No | No | 3.572 | 0.835 | No |
| CHEMBL3661403 | [O-][S+](c1ccccc1)C1OC1c1ccccc1 | 244.315 | 2.8918 | 3 | 2 | 0 | 103.42 | -3.598 | 1.57 | 96.252 | -2.673 | Yes | No | No | 0.331 | 0.047 | 0.268 | -1.48 | No | Yes | Yes | Yes | No | No | No | 0.378 | Yes | Yes | 0.384 | No | No | 1.751 | 0.824 | No |
| CHEMBL3990145 | COCCCCn1c(C(=O)N(CC(C)C)[C@@H]2CNC[C@H](C(=O)N3CCOCC3)C2)nc2ccccc21 | 499.656 | 2.398 | 10 | 7 | 1 | 213.659 | -3.404 | 1.024 | 88.098 | -2.735 | Yes | No | No | 0.424 | 0.275 | -0.69 | -3.136 | No | Yes | No | No | No | No | No | 0.799 | Yes | Yes | 0.165 | No | Yes | 2.34 | 0.486 | Yes |
| CHEMBL2106789 | C[C@@H]1O[C@@H](CC(=O)O)CC2=C1C(=O)c1c(O)cccc1C2=O | 302.282 | 1.7199 | 2 | 5 | 2 | 125.566 | -2.756 | 0.669 | 63.056 | -2.736 | Yes | No | No | -0.545 | 0.288 | -0.371 | -3.113 | No | No | No | No | No | No | No | 0.329 | No | No | 1.091 | No | No | 2.458 | 1.917 | No |
| CHEMBL3989450 | C[C@H](N[C@@H](CCc1ccccc1)C(=O)O)C(=O)N1C2CCC(CC2)[C@H]1C(=O)O | 388.464 | 1.9047 | 8 | 4 | 3 | 164.277 | -2.676 | 0.681 | 32.465 | -2.735 | Yes | No | No | -2.075 | 0.437 | -0.926 | -3.15 | No | No | No | No | No | No | No | 0.612 | No | No | 0.574 | No | No | 1.848 | 2.772 | Yes |
| CHEMBL259389 | Nc1n[nH]c2nc3ccccc3nc12 | 185.19 | 1.0883 | 0 | 4 | 2 | 78.971 | -2.459 | 1.115 | 54.155 | -2.768 | No | No | No | -0.339 | 0.291 | -1.174 | -2.661 | No | No | Yes | No | No | No | No | 0.436 | No | No | 0.176 | No | No | 2.674 | 1.338 | No |
| CHEMBL324954 | CN[C@H](Cc1ccccc1)C(=O)N1CCC[C@H]1C(=O)N[C@H](C=O)CCCN=C(N)N.O=S(=O)(O)O | 514.605 | -1.1074 | 11 | 7 | 6 | 204.457 | -2.831 | -0.512 | 0 | -2.735 | Yes | No | No | -0.711 | 0.687 | -1.523 | -4.308 | No | Yes | No | No | No | No | No | 0.627 | No | No | 0.603 | No | No | 2.345 | 2.922 | Yes |
| CHEMBL223448 | Oc1c(Br)cc(Br)c2cccnc12 | 302.953 | 3.4654 | 0 | 2 | 1 | 91.862 | -3.256 | 1.387 | 92.419 | -2.062 | No | No | No | -0.021 | 0.361 | 0.336 | -2.301 | No | No | Yes | No | No | No | No | 0.096 | No | No | 0.51 | No | No | 2.305 | 1.906 | No |
| CHEMBL2105038 | NN(Cc1ccccc1)CC1COc2ccccc2O1 | 270.332 | 2.2023 | 4 | 4 | 1 | 118.239 | -2.958 | 1.327 | 93.733 | -2.451 | No | No | No | 0.085 | 0.053 | 0.375 | -1.708 | No | Yes | Yes | Yes | No | No | No | 0.698 | Yes | No | 0.375 | No | No | 2.477 | 1.619 | Yes |
| CHEMBL2107365 | C[C@@H]1NC(C)(C)CO[C@@]1(O)c1cccc(Cl)c1.Cl | 292.206 | 2.6938 | 1 | 3 | 2 | 118.894 | -2.382 | 1.274 | 92.346 | -3.015 | Yes | No | No | 0.444 | 0.334 | 0.38 | -1.934 | No | No | No | No | No | No | No | 1.282 | No | No | 0.181 | No | No | 2.683 | 1.028 | No |
| CHEMBL1598450 | CCCn1c(=O)c2nc[nH]c2n(-c2ccc(Cl)cc2)c1=O | 304.737 | 1.9389 | 3 | 5 | 1 | 124.179 | -3.05 | 1.084 | 94.375 | -2.735 | Yes | No | No | -0.126 | 0.292 | -1.244 | -4.032 | Yes | No | Yes | No | No | No | No | 0.725 | No | Yes | 0.858 | No | No | 2.214 | 1.031 | Yes |
| CHEMBL3644464 | Cc1nc2c([nH]1)CN([C@H]1CSC(c3cc(F)ccc3F)[C@@H](N)C1)C2 | 350.438 | 2.88602 | 2 | 4 | 2 | 143.379 | -2.724 | 1.254 | 90.883 | -2.735 | Yes | No | No | -0.052 | 0.427 | 0.3 | -2.343 | No | No | No | No | No | No | No | 0.668 | Yes | No | 0.471 | No | Yes | 2.387 | 0.744 | Yes |
| CHEMBL3644454 | N[C@H]1C[C@@H](N2Cc3c[nH]nc3C2)CS(=O)(=O)C1c1cc(F)cc(F)c1F | 386.399 | 1.3983 | 2 | 5 | 2 | 147.65 | -2.448 | 0.821 | 68.641 | -3.042 | Yes | No | No | 0.673 | 0.374 | -1.421 | -3.429 | No | Yes | No | No | No | No | No | 0.079 | No | No | -0.352 | No | No | 2.671 | 2.738 | Yes |
| CHEMBL51934 | Nc1c2c(nc3ccccc13)CCCC2O | 214.268 | 2.1867 | 0 | 3 | 2 | 93.921 | -2.572 | 1.197 | 91.578 | -3.049 | Yes | No | No | 0.448 | 0.297 | 0.404 | -2.012 | No | No | Yes | No | No | No | No | 0.246 | No | Yes | 0.123 | No | No | 2.425 | 1.573 | No |
| CHEMBL2107586 | CCCCCCCNC(=O)Oc1ccc2c(c1)[C@]1(C)CCN(C)O[C@@H]1N2C | 375.513 | 4.0461 | 7 | 5 | 1 | 162.306 | -5.126 | 1.227 | 90.387 | -3.069 | No | Yes | Yes | 0.283 | 0 | 0.193 | -2.294 | No | Yes | No | Yes | No | No | No | 1.087 | No | No | -0.264 | No | No | 3.064 | 1.517 | Yes |
| CHEMBL54440 | CC[C@H](C)C(=O)O[C@H]1CCC=C2C=C[C@H](C)[C@H](CC[C@@H]3C[C@@H](O)CC(=O)O3)[C@H]21 | 390.52 | 3.9495 | 6 | 5 | 1 | 167.716 | -4.46 | 1.196 | 95.405 | -3.319 | Yes | Yes | Yes | 0.176 | 0.184 | -0.38 | -2.851 | No | Yes | No | No | No | No | Yes | 1.012 | No | No | -0.164 | No | Yes | 2.221 | 1.574 | No |
| CHEMBL48092 | O=C(O)CCCCCc1cccc2cncn12 | 232.283 | 2.5218 | 6 | 3 | 1 | 99.915 | -2.661 | 1.268 | 93.955 | -2.75 | Yes | No | No | -0.016 | 0.409 | 0.079 | -2.881 | No | No | No | No | No | No | No | 0.948 | No | Yes | 0.093 | No | No | 2.288 | 1.821 | No |
| CHEMBL80937 | CN1C[C@H](CC#N)C[C@@H]2c3cccc4[nH]c(Cl)c(c34)C[C@H]21 | 299.805 | 3.69498 | 1 | 2 | 1 | 129.051 | -4.466 | 1.265 | 93.216 | -2.943 | Yes | No | No | 1.331 | 0.203 | 0.142 | -1.637 | Yes | Yes | Yes | No | No | Yes | No | 0.817 | No | Yes | -0.585 | No | Yes | 2.713 | 1.623 | Yes |
| CHEMBL1290459 | O=C(O)C1=Cc2cc(OC(F)(F)F)ccc2OC1C(F)(F)F | 328.164 | 3.3765 | 2 | 3 | 1 | 118.102 | -4.238 | 1.386 | 89.152 | -2.869 | No | No | No | -0.911 | 0.248 | -0.135 | -2.95 | No | No | No | No | No | No | No | 0.376 | No | No | 0.092 | No | No | 3.314 | 1.503 | Yes |
| CHEMBL461939 | O=C(O)c1ccc(/C=C2\SC(=S)N(c3cccc(C(F)(F)F)c3)C2=O)cc1 | 409.41 | 4.8094 | 3 | 4 | 1 | 160.838 | -5.651 | 1.449 | 89.51 | -2.671 | Yes | No | No | -0.504 | 0 | -0.277 | -1.767 | No | Yes | No | No | Yes | No | No | -0.066 | No | No | -0.323 | No | No | 3.006 | 1.798 | Yes |
| CHEMBL1204135 | CC(C)CN[C@H]1CCS(=O)(=O)c2sc(S(N)(=O)=O)cc21.Cl | 374.937 | 1.2814 | 4 | 6 | 2 | 135.05 | -2.521 | 0.447 | 84.856 | -3.327 | No | No | No | -0.091 | 0.443 | -1.03 | -3.154 | No | No | No | No | No | No | No | 0.464 | No | No | 0.648 | No | No | 2.86 | 1.778 | Yes |
| CHEMBL485569 | COc1cc(-c2ccccc2C)cc(C(C)C#Cc2c(C)nc(N)nc2N)c1 | 372.472 | 4.08864 | 3 | 5 | 2 | 165.332 | -4.59 | 0.8 | 96.548 | -2.764 | Yes | Yes | Yes | -0.27 | 0 | 0.1 | -1.995 | No | Yes | Yes | Yes | Yes | No | Yes | 0.176 | No | Yes | -0.126 | No | Yes | 2.77 | 2.531 | Yes |
| CHEMBL2106052 | NNCC1CCCCC1 | 128.219 | 1.03 | 2 | 2 | 2 | 56.783 | -1.709 | 1.326 | 77.497 | -2.157 | No | No | No | 0.1 | 0.521 | -0.121 | -1.797 | No | No | No | No | No | No | No | 0.329 | No | No | 0.714 | No | No | 2.313 | 1.418 | No |
| CHEMBL2364604 | Cl.Nc1cc(C(F)(F)F)c(-c2cc(N3CCOCC3)nc(N3CCOCC3)n2)cn1 | 446.861 | 2.2346 | 3 | 8 | 1 | 177.157 | -5.29 | 1.084 | 96.403 | -2.902 | No | No | No | -0.777 | 0.35 | -1.54 | -3.136 | No | Yes | No | No | No | No | No | 0.681 | No | No | -0.42 | No | No | 2.589 | 1.037 | Yes |
| CHEMBL459177 | COc1cc(-c2ccccc2)cc([C@@H](C)C#Cc2c(C)nc(N)nc2N)c1 | 358.445 | 3.78022 | 3 | 5 | 2 | 158.967 | -4.474 | 0.997 | 95.754 | -2.758 | Yes | Yes | Yes | -0.424 | 0 | 0.093 | -2.075 | No | Yes | Yes | Yes | Yes | No | Yes | 0.161 | No | Yes | -0.154 | No | Yes | 2.742 | 2.64 | Yes |
| CHEMBL6259 | CN1CCN(c2cc3c(cc2F)c(=O)c(C(=O)O)cn3-c2ccc(F)cc2)CC1 | 399.397 | 2.7189 | 3 | 5 | 1 | 164.752 | -3.849 | 1.184 | 96.517 | -2.735 | Yes | No | No | -0.422 | 0.25 | 0.094 | -2.462 | No | No | No | No | No | No | No | 0.149 | No | No | 0.766 | No | No | 2.903 | 0.959 | Yes |
| CHEMBL3651712 | COc1cc(-n2ccc(SCc3ccc(Cl)cc3)cc2=O)ccc1OCC(C)(C)O | 445.968 | 4.9415 | 8 | 6 | 1 | 184.774 | -5.863 | 1.246 | 95.026 | -2.651 | Yes | Yes | Yes | 0.278 | 0.129 | -0.362 | -2.17 | No | Yes | Yes | Yes | Yes | No | Yes | 0.097 | No | No | 0.4 | No | No | 2.392 | 1.597 | No |
| CHEMBL2107625 | COc1cc2c(cc1OC)[C@@H]1CN(C)CC[C@@H]1N=C2c1ccc(NC(C)=O)cc1 | 393.487 | 3.3009 | 4 | 5 | 1 | 171.085 | -3.486 | 1.092 | 93.495 | -2.908 | Yes | Yes | Yes | 1.288 | 0.209 | 0.055 | -2.237 | No | Yes | No | No | No | No | Yes | 0.763 | No | No | -0.324 | No | Yes | 3.195 | 0.984 | Yes |
| CHEMBL1201365 | C[C@H](N[C@@H](CCc1ccccc1)C(=O)O)C(=O)N1[C@H](C(=O)O)C[C@@H]2CCC[C@@H]21 | 388.464 | 1.9047 | 8 | 4 | 3 | 164.277 | -2.664 | 0.693 | 27.508 | -2.735 | Yes | No | No | -2.088 | 0.422 | -0.929 | -3.083 | No | No | No | No | No | No | No | 0.416 | No | No | 0.572 | No | No | 1.784 | 2.832 | Yes |
| CHEMBL115653 | CN1C(=O)N(C[C@H](C(=O)NO)[C@@H](CC2CCCC2)C(=O)N2CCCCC2)C(=O)C1(C)C | 436.553 | 1.9896 | 7 | 5 | 2 | 183.609 | -3.223 | 0.626 | 65.589 | -2.89 | Yes | No | No | -0.556 | 0.259 | -1.014 | -2.935 | No | No | No | No | No | No | No | 0.949 | No | No | -0.411 | No | No | 2.342 | 2.3 | No |
| CHEMBL4298137 | CC1=C[C@@H]2CN(C(=O)N2OS(=O)(=O)O)[C@@H]1C(N)=O | 277.258 | -1.3592 | 3 | 5 | 2 | 102.68 | -1.927 | -0.122 | 17.712 | -2.735 | No | No | No | -0.765 | 0.753 | -1.107 | -3.324 | No | No | No | No | No | No | No | 0.186 | No | No | 1.379 | No | No | 1.756 | 2.557 | No |
| CHEMBL560910 | O=[14C](O)COc1cc(Cl)c(Cl)cc1Cl | 257.4762 | 3.1102 | 3 | 2 | 1 | 95.14 | -3.272 | 1.321 | 90.557 | -2.721 | No | No | No | -0.675 | 0.3 | 0.202 | -2.052 | No | No | No | No | No | No | No | 0.444 | No | No | 0.891 | No | No | 2.987 | 1.815 | No |
| CHEMBL3661405 | OC(c1ccccc1)C1OC1c1ccc(F)cc1 | 244.265 | 2.9992 | 3 | 2 | 1 | 104.651 | -3.528 | 1.439 | 93.737 | -2.729 | No | No | No | 0.151 | 0.116 | 0.139 | -1.792 | No | Yes | Yes | Yes | Yes | No | No | 0.018 | No | Yes | 0.445 | No | No | 1.754 | 2.062 | No |
| CHEMBL2364611 | Cc1cccc(-c2nn3c(c2-c2ccnc4ccc(C(N)=O)cc24)CCC3)n1 | 369.428 | 3.51382 | 3 | 5 | 1 | 161.594 | -3.1 | 1.121 | 100 | -2.748 | Yes | Yes | Yes | 0.624 | 0.338 | -0.665 | -2.799 | No | Yes | Yes | Yes | No | No | Yes | 0.446 | Yes | No | 0.143 | No | Yes | 2.824 | 0.813 | Yes |
| CHEMBL3989948 | CC#Cc1cncc(-c2ccc3c(c2)[C@@]2(N=C(C)C(N)=N2)[C@]2(CC[C@H](OC)CC2)C3)c1 | 412.537 | 4.236 | 2 | 5 | 1 | 183.408 | -5.014 | 0.728 | 96.521 | -2.856 | Yes | Yes | Yes | 0.763 | 0.048 | -0.178 | -1.752 | No | Yes | No | Yes | Yes | No | Yes | 0.466 | Yes | No | -0.58 | No | Yes | 2.552 | 1.13 | Yes |
| CHEMBL3545213 | N#CC(c1ccnc(OCc2ccc(CN3CCOCC3)cc2)n1)c1nc2ccccc2s1 | 457.559 | 4.15298 | 7 | 8 | 0 | 195.82 | -4.084 | 1.239 | 95.463 | -2.754 | No | Yes | Yes | 0.673 | 0.169 | -0.98 | -2.467 | No | Yes | Yes | Yes | Yes | No | Yes | 0.899 | Yes | No | 0.329 | No | Yes | 3.032 | 0.912 | Yes |
| CHEMBL460785 | Cl.OC[C@H]1NC[C@H](O)[C@@H](O)[C@@H]1O | 199.634 | -2.545 | 1 | 5 | 5 | 76.438 | -0.292 | -0.314 | 43.99 | -3.311 | No | No | No | -0.716 | 0.906 | -1.288 | -5.13 | No | No | No | No | No | No | No | 1.38 | No | No | 2.263 | No | No | 1.849 | 2.351 | No |
| CHEMBL507361 | O=C(NOC[C@H](O)CO)c1ccc(F)c(F)c1Nc1ccc(I)cc1F | 482.196 | 2.4667 | 7 | 5 | 4 | 159.624 | -3.915 | 0.88 | 94.06 | -2.737 | Yes | Yes | No | -0.297 | 0.104 | -1.59 | -3.646 | No | No | No | No | No | No | No | -0.156 | No | No | 0.633 | No | Yes | 3.176 | 2.925 | Yes |
| CHEMBL4084907 | Nc1cc(C(F)(F)F)c(-c2nc(N3CCOCC3)nc(N3CCOCC3)n2)cn1 | 411.388 | 1.2078 | 3 | 9 | 1 | 164.223 | -4.601 | 0.606 | 91.852 | -2.872 | No | No | No | -0.93 | 0.312 | -1.596 | -3.214 | No | Yes | No | No | No | No | No | -0.007 | No | No | -0.015 | No | No | 2.505 | 1.157 | Yes |
| CHEMBL91238 | CC(C)NNC(=O)COc1ccc(Cl)cc1 | 242.706 | 1.748 | 5 | 3 | 2 | 99.904 | -2.725 | 1.294 | 91.931 | -3.012 | Yes | No | No | -0.31 | 0.296 | 0.124 | -2.665 | No | No | No | No | No | No | No | -0.163 | No | No | 1.278 | No | No | 2.751 | 1.716 | Yes |
| CHEMBL3544988 | O=C(O)CNC(=O)C1C(=O)N(C2CCCCC2)C(=O)N(C2CCCCC2)C1=O | 393.44 | 1.2596 | 5 | 5 | 2 | 162.962 | -3.079 | 0.085 | 42.898 | -2.88 | Yes | No | No | -0.44 | 0.599 | -1.086 | -3.63 | No | No | No | No | No | No | No | 1.339 | No | No | 0.575 | No | No | 3.011 | 1.895 | No |
| CHEMBL36591 | CC(N)C(=O)OC(C)(C)Cc1ccc(Cl)cc1 | 255.745 | 2.5515 | 4 | 3 | 1 | 106.904 | -2.192 | 1.32 | 93.542 | -2.68 | No | No | No | 0.54 | 0.382 | 0.17 | -2.263 | No | No | Yes | Yes | No | Yes | Yes | 0.765 | No | No | 0.743 | No | No | 2.771 | 1.682 | Yes |
| CHEMBL3644587 | O=c1[nH]nc2c3c(cccc13)N=C(CN1Cc3ccccc3C1)N2 | 317.352 | 2.3152 | 2 | 4 | 2 | 136.699 | -2.946 | 0.757 | 92.927 | -2.735 | Yes | No | No | 0.322 | 0.167 | -1.302 | -2.513 | Yes | Yes | Yes | No | No | Yes | No | 1.113 | Yes | No | 0.612 | No | Yes | 2.61 | 1.522 | Yes |
| CHEMBL2107832 | O=C(NC[C@H](O)CO)c1ccncc1Nc1ccc(I)cc1F | 431.205 | 1.6519 | 6 | 5 | 4 | 145.445 | -3.262 | -0.336 | 72.205 | -2.865 | No | No | No | -0.565 | 0.38 | -1.561 | -3.853 | No | No | No | No | No | No | No | 0.155 | No | No | 0.65 | No | Yes | 2.673 | 1.579 | Yes |
| CHEMBL4283683 | Cn1cc(-c2ccc(S(=O)(=O)n3ccc(/C=C/C(=O)Nc4ccccc4N)c3)cc2)cn1 | 447.52 | 3.3597 | 6 | 7 | 2 | 185.211 | -4.104 | 0.671 | 94.416 | -2.737 | Yes | Yes | Yes | -0.171 | 0 | -0.639 | -2.461 | No | Yes | Yes | Yes | Yes | No | Yes | 0.606 | No | Yes | 0.093 | No | Yes | 2.802 | 3.068 | Yes |
| CHEMBL86304 | O=C(NCCN1CCOCC1)c1ccc(Cl)cc1 | 268.744 | 1.402 | 4 | 3 | 1 | 111.868 | -2.391 | 1.481 | 93.872 | -2.743 | Yes | No | No | 0.532 | 0.493 | -0.021 | -1.909 | No | No | No | No | No | No | No | 1.057 | No | No | 0.28 | No | No | 2.241 | 1.292 | Yes |
| CHEMBL6437 | CN1CCN2c3ccccc3Cc3ccccc3C2C1 | 264.372 | 3.0839 | 0 | 2 | 0 | 120.186 | -3.963 | 1.466 | 97.375 | -2.433 | No | Yes | No | 1.533 | 0.134 | 0.773 | -1.344 | No | Yes | Yes | No | No | Yes | No | 0.805 | Yes | No | -0.2 | No | Yes | 2.747 | 0.636 | Yes |
| CHEMBL549 | CN(C)CCCC1(c2ccc(F)cc2)OCc2cc(C#N)ccc21 | 324.399 | 3.81298 | 5 | 3 | 0 | 141.834 | -4.601 | 1.488 | 97.476 | -2.569 | No | Yes | No | 1.337 | 0.091 | 0.02 | -1.472 | No | Yes | No | No | No | Yes | No | 0.899 | Yes | No | 0.075 | No | Yes | 2.855 | 1.665 | No |
| CHEMBL1095032 | O=C([C@H](O)CS(=O)(=O)c1ccc2cc(Cl)ccc2c1)N1CCC(N2CCCNC2=O)CC1 | 479.986 | 2.0341 | 5 | 5 | 2 | 191.596 | -3.417 | 0.191 | 69.357 | -2.819 | Yes | Yes | No | 0.169 | 0.084 | -0.968 | -2.952 | No | Yes | No | No | No | No | No | 0.178 | No | No | -0.585 | No | Yes | 2.491 | 2.279 | Yes |
| CHEMBL248183 | NCC1CCC(CNc2nc(NCc3ccccc3OC(F)(F)F)ncc2[N+](=O)[O-])CC1 | 454.453 | 4.0725 | 9 | 8 | 3 | 182.274 | -5.07 | 0.157 | 84.592 | -2.749 | Yes | Yes | Yes | 0.398 | 0.086 | -1.329 | -2.612 | No | Yes | No | No | No | No | Yes | 0.139 | No | Yes | -0.367 | No | Yes | 3.336 | 1.476 | No |
| CHEMBL3675607 | NC(=O)[C@H](CCC(=O)O)NC(=O)CCc1ccc(-c2cc(-c3ccccc3)cs2)cc1 | 436.533 | 3.8496 | 10 | 4 | 3 | 184.274 | -4.753 | 0.418 | 65.66 | -2.735 | Yes | No | No | -1.515 | 0.044 | -0.922 | -2.773 | No | Yes | No | No | Yes | No | No | -0.242 | No | Yes | 0.538 | No | No | 2.296 | 2.721 | Yes |
| CHEMBL287257 | COc1ccc2c(c1)CN(C)CC2c1ccc(Cl)c(Cl)c1 | 322.235 | 4.5793 | 2 | 2 | 0 | 134.787 | -4.152 | 1.457 | 90.901 | -2.586 | Yes | Yes | Yes | 1.426 | 0.154 | 0.821 | -1.326 | No | Yes | Yes | Yes | No | Yes | No | 0.81 | No | No | 0.208 | Yes | Yes | 3.433 | 0.965 | No |
| CHEMBL3889654 | O=C(Nc1cnn2ccc(N3CCC[C@@H]3c3cc(F)ccc3F)nc12)N1CC[C@H](O)C1 | 428.443 | 2.9475 | 3 | 6 | 2 | 176.644 | -3.515 | 1.258 | 94.879 | -2.86 | Yes | No | No | 0.594 | 0.225 | -1.673 | -3.067 | No | Yes | No | Yes | No | No | Yes | 0.068 | No | No | -0.453 | No | Yes | 2.655 | 1.068 | Yes |
| CHEMBL511115 | COc1ccc([C@]2(C#N)CC[C@@H](C(=O)O)CC2)cc1OC1CCCC1 | 343.423 | 4.05278 | 5 | 4 | 1 | 148.104 | -4.074 | 0.721 | 99.38 | -2.728 | No | No | No | -0.702 | 0.015 | -0.276 | -2.154 | No | Yes | No | No | No | No | No | 1.068 | No | No | 0.381 | No | No | 2.563 | 1.888 | No |
| CHEMBL4297676 | Cl.O=C(NC[C@H](O)CO)c1ccncc1Nc1ccc(I)cc1F | 467.666 | 2.0737 | 6 | 5 | 4 | 157.598 | -3.503 | -0.109 | 71.96 | -2.851 | No | No | No | -0.606 | 0.354 | -1.696 | -3.864 | No | No | No | No | No | No | No | -0.051 | No | No | 0.628 | No | Yes | 2.694 | 1.544 | No |
| CHEMBL2364621 | CC(=O)c1c(C)c2cnc(Nc3ccc(N4CCNCC4)cn3)nc2n(C2CCCC2)c1=O.O=S(=O)(O)CCO | 573.676 | 1.83232 | 7 | 12 | 4 | 231.968 | -3.276 | -0.386 | 37.523 | -2.735 | Yes | No | No | -0.164 | 0.393 | -2.01 | -3.419 | No | No | No | No | No | No | No | 0.784 | No | No | 0.629 | No | No | 2.005 | 1.941 | Yes |
| CHEMBL668 | CNCCCC1c2ccccc2C=Cc2ccccc21 | 263.384 | 4.3019 | 4 | 1 | 1 | 120.898 | -4.613 | 1.446 | 97.605 | -2.575 | Yes | Yes | No | 1.703 | 0.051 | 0.818 | -1.287 | No | Yes | No | No | No | Yes | Yes | 0.953 | No | No | 0.366 | No | Yes | 2.71 | 1.253 | Yes |
| CHEMBL1200374 | C=C1C[C@@H]2[C@H](CC[C@]3(C)C(=O)CC[C@@H]23)[C@@]2(C)C=CC(=O)C=C12 | 296.41 | 4.0295 | 0 | 2 | 0 | 131.904 | -4.785 | 1.331 | 98.9 | -2.926 | No | Yes | No | 0.343 | 0.098 | 0.22 | -2.225 | No | Yes | No | No | No | No | No | 1.015 | Yes | No | -0.552 | No | Yes | 1.702 | 1.733 | No |
| CHEMBL2396661 | Cc1nc(NC(=O)N2CCC[C@H]2C(N)=O)sc1-c1ccnc(C(C)(C)C(F)(F)F)c1 | 441.479 | 3.83502 | 4 | 5 | 2 | 175.046 | -4.707 | 0.646 | 78.182 | -3.097 | Yes | No | No | -0.307 | 0.207 | -1.455 | -3.108 | No | No | No | No | No | No | No | 0.025 | No | No | -0.031 | No | No | 2.552 | 0.269 | Yes |
| CHEMBL4297610 | C[C@H](Nc1ccc(C#N)n(C)c1=O)c1cc2cc(Cl)ccc2[nH]c1=O | 354.797 | 2.92498 | 3 | 5 | 2 | 147.848 | -4.556 | 1.059 | 94.695 | -3.01 | Yes | No | No | 0.012 | 0.268 | -0.417 | -2.792 | No | Yes | Yes | Yes | No | No | Yes | -0.374 | No | No | -0.57 | No | Yes | 2.549 | 0.779 | Yes |
| CHEMBL3621988 | Nc1cc(F)ccc1NC(=O)c1ccc(CNC(=O)/C=C/c2cccnc2)cc1 | 390.418 | 3.3848 | 6 | 4 | 3 | 166.829 | -3.773 | 1.135 | 90.24 | -2.752 | Yes | Yes | Yes | 0.179 | 0.033 | -0.904 | -2.429 | No | Yes | No | Yes | Yes | No | Yes | 0.232 | No | No | 0.003 | No | Yes | 1.93 | 1.54 | Yes |
| CHEMBL93 | CC(c1cc2ccccc2s1)N(O)C(N)=O | 236.296 | 2.7322 | 2 | 3 | 2 | 96.858 | -2.783 | 0.818 | 89.46 | -2.769 | No | No | No | 0.048 | 0.225 | 0.197 | -2.186 | No | Yes | Yes | Yes | No | No | No | 0.12 | No | No | 0.815 | No | No | 3.004 | 1.694 | No |
| CHEMBL2105763 | Cn1cc(CNCC2CCN(c3ncc(C(=O)NO)cn3)CC2)c2ccccc21 | 394.479 | 2.0935 | 6 | 7 | 3 | 169.077 | -3.399 | 0.957 | 75.058 | -2.962 | Yes | Yes | No | 0.542 | 0.266 | -0.998 | -2.827 | No | Yes | No | No | No | No | No | 0.648 | No | No | 0.221 | No | Yes | 3.011 | 2.076 | Yes |
| CHEMBL4303214 | CNC(=O)c1c(C)oc2cc(Oc3ncnc4cc(OC)c(OC)cc34)ccc12 | 393.399 | 3.85352 | 5 | 7 | 1 | 165.962 | -3.528 | 1.784 | 98.271 | -2.735 | No | Yes | Yes | -0.3 | 0.244 | -1.086 | -3.357 | No | Yes | Yes | No | Yes | No | Yes | 1.034 | No | No | 0.648 | No | Yes | 2.478 | 0.991 | Yes |
| CHEMBL3545414 | CS(=O)(=O)O.N#CC1(c2ccc(NC(=O)c3cccnc3NCc3ccncc3)cc2)CCCC1 | 493.589 | 4.18038 | 6 | 7 | 3 | 204.552 | -3.568 | -0.563 | 52.704 | -2.735 | Yes | No | No | -0.277 | 0.022 | -1.284 | -3.255 | No | Yes | No | No | Yes | No | No | 0.061 | No | No | 0.163 | No | No | 2.587 | 1.74 | Yes |
| CHEMBL1207745 | CC#CCC(C)[C@H](O)/C=C/[C@@H]1[C@H]2c3cccc(CCCC(=O)O)c3O[C@H]2C[C@H]1O | 398.499 | 3.2859 | 8 | 4 | 3 | 171.956 | -4.355 | 0.881 | 96.296 | -2.733 | Yes | No | Yes | -0.729 | 0.096 | -0.639 | -2.654 | No | Yes | No | No | No | No | No | 1.149 | No | No | -0.926 | No | No | 3.184 | 2.016 | No |
| CHEMBL869 | NC(=O)N/N=C/c1ccc([N+](=O)[O-])o1 | 198.138 | 0.19 | 3 | 5 | 2 | 77.525 | -2.431 | -0.22 | 74.78 | -2.981 | No | No | No | -0.494 | 0.602 | -0.732 | -3.141 | No | No | Yes | No | No | No | No | 0.79 | No | Yes | 0.968 | No | No | 2.563 | 1.37 | No |
| CHEMBL2218861 | CCCN1CC(NS(=O)(=O)N(CC)CC)CC2Cc3c(O)cccc3CC21 | 395.569 | 2.1361 | 7 | 4 | 2 | 163.289 | -3.127 | 1.082 | 94.836 | -3.045 | Yes | No | No | 0.889 | 0.406 | -0.894 | -3.01 | No | No | No | No | No | No | No | 0.509 | No | No | -0.622 | No | Yes | 3.087 | 0.788 | Yes |
| CHEMBL1200904 | C#CCN(C)[C@H](C)Cc1ccccc1.Cl | 223.747 | 2.6044 | 4 | 1 | 0 | 98.898 | -2.811 | 1.554 | 93.838 | -1.891 | No | No | No | 1.082 | 0.355 | 0.814 | -1.576 | Yes | No | Yes | No | No | Yes | No | 1.487 | Yes | No | 0.383 | No | Yes | 2.687 | 1.289 | Yes |
| CHEMBL408194 | COC1=CC(c2cc3ccccc3[nH]2)=N/C1=C\c1[nH]c(C)cc1C | 317.392 | 4.48694 | 3 | 2 | 2 | 140.132 | -4.184 | 1.282 | 89.019 | -2.602 | Yes | No | No | 0.76 | 0.164 | 0.27 | -1.724 | No | Yes | Yes | Yes | No | No | Yes | 0.962 | Yes | Yes | -0.548 | No | Yes | 2.597 | 0.468 | Yes |
| CHEMBL1520 | CCCc1nc(C)c2c(=O)nc(-c3cc(S(=O)(=O)N4CCN(CC)CC4)ccc3OCC)[nH]n12 | 488.614 | 2.07042 | 8 | 8 | 1 | 199.163 | -3.017 | 1.241 | 74.325 | -2.735 | Yes | No | No | 0.814 | 0.411 | -0.992 | -3.299 | No | No | No | No | No | No | Yes | 0.356 | Yes | Yes | 0.35 | No | No | 2.248 | 1.825 | Yes |
| CHEMBL502 | COc1cc2c(cc1OC)C(=O)C(CC1CCN(Cc3ccccc3)CC1)C2 | 379.5 | 4.3611 | 6 | 4 | 0 | 167.005 | -4.632 | 1.321 | 92.768 | -2.647 | Yes | Yes | Yes | 1.179 | 0.01 | 0.479 | -1.445 | Yes | Yes | No | No | No | Yes | Yes | 0.994 | Yes | No | -0.171 | No | Yes | 2.731 | 1.378 | Yes |
| CHEMBL38458 | CCN(CC)CCn1nc2c3c(c(NCCN)ccc31)Sc1cc(O)ccc1-2 | 397.548 | 3.8222 | 8 | 7 | 3 | 168.087 | -3.507 | 0.777 | 90.284 | -2.735 | Yes | Yes | Yes | 1.457 | 0.178 | -1.131 | -2.501 | Yes | Yes | Yes | Yes | No | No | Yes | 1.222 | Yes | Yes | 0.789 | No | Yes | 2.439 | 1.046 | Yes |
| CHEMBL4594440 | Fc1cc(F)c2c(c1)C[C@@H](n1c(CCNCc3ccccc3)c[nH]c1=S)CO2 | 401.482 | 4.33239 | 6 | 4 | 2 | 166.721 | -2.891 | 0.198 | 85.904 | -2.735 | Yes | Yes | Yes | 0.948 | 0.086 | 0.532 | -0.826 | Yes | Yes | Yes | Yes | Yes | Yes | No | 0.981 | Yes | Yes | -0.219 | No | Yes | 2.614 | 1.781 | Yes |
| CHEMBL137 | Cc1ncc([N+](=O)[O-])n1CCO | 171.156 | 0.09202 | 3 | 5 | 1 | 68.612 | -1.496 | 0.625 | 92.553 | -2.824 | Yes | No | No | -0.22 | 0.68 | -0.657 | -3.021 | No | No | No | No | No | No | No | 0.458 | No | Yes | 0.235 | No | No | 1.938 | 1.472 | No |
| CHEMBL1628504 | Cc1cc(/C=C/C#N)cc(C)c1Nc1ccnc(Nc2ccc(C#N)cc2)n1.Cl | 402.889 | 5.4109 | 5 | 6 | 2 | 175.523 | -4.785 | 0.405 | 97.377 | -2.78 | Yes | Yes | Yes | 0.358 | 0.034 | -0.212 | -1.665 | No | Yes | Yes | Yes | Yes | No | Yes | -0.592 | No | No | 0.034 | No | Yes | 2.641 | 1.008 | Yes |
| CHEMBL4297527 | O=C(Nc1c(Cl)c[n+]([O-])cc1Cl)c1ccc(OC(F)F)c2oc3ccncc3c12 | 440.189 | 4.7749 | 4 | 5 | 1 | 170.536 | -4.08 | 1.093 | 90.486 | -2.763 | Yes | Yes | Yes | 0.361 | 0.255 | -1.19 | -2.846 | No | Yes | Yes | No | No | No | Yes | 0.364 | No | Yes | -0.336 | No | Yes | 2.942 | 0.538 | Yes |
| CHEMBL1229 | CCOC(=O)C1=C[C@@H](OC(CC)CC)[C@H](NC(C)=O)[C@@H](N)C1 | 312.41 | 1.2854 | 7 | 5 | 2 | 131.958 | -2.086 | 0.618 | 67.882 | -2.787 | Yes | No | No | -0.154 | 0.764 | -0.312 | -3.424 | No | No | No | No | No | No | No | 0.926 | No | No | 0.185 | No | No | 2.253 | 0.445 | No |
| CHEMBL1064 | CCC(C)(C)C(=O)O[C@H]1C[C@@H](C)C=C2C=C[C@H](C)[C@H](CC[C@@H]3C[C@@H](O)CC(=O)O3)[C@H]21 | 418.574 | 4.5856 | 6 | 5 | 1 | 180.446 | -4.987 | 0.977 | 95.313 | -3.347 | Yes | Yes | Yes | 0.197 | 0.099 | -0.37 | -2.812 | No | Yes | No | No | No | No | Yes | 0.827 | No | No | -0.141 | No | No | 2.14 | 0.445 | No |
| CHEMBL564829 | CNC(=O)c1nn(C)c2c1C(C)(C)Cc1cnc(Nc3ccc(N4CCN(C)CC4)cc3)nc1-2 | 460.586 | 2.5658 | 4 | 8 | 2 | 199.741 | -4.05 | 1.302 | 93.896 | -2.77 | Yes | Yes | Yes | 0.848 | 0.172 | -0.853 | -2.58 | No | Yes | No | No | No | No | Yes | 0.556 | Yes | No | 0.12 | No | Yes | 2.729 | 0.818 | Yes |
| CHEMBL3930624 | O=C(c1cc(Cc2n[nH]c(=O)c3ccccc23)ccc1F)N1CCn2nc(C(F)(F)F)nc2C1 | 472.402 | 2.9193 | 3 | 6 | 1 | 188.391 | -3.658 | 1.166 | 92.403 | -2.735 | Yes | Yes | Yes | -0.185 | 0.029 | -1.579 | -3.385 | Yes | Yes | No | Yes | Yes | No | Yes | 0.02 | No | No | 0.544 | No | Yes | 2.621 | 1.357 | Yes |
| CHEMBL213934 | CC(C)(C)OC(=O)N[C@@H](CCCCCS)C(=O)NC1CCCC1 | 344.521 | 3.4287 | 8 | 4 | 3 | 145.264 | -3.854 | 0.683 | 87.677 | -3.349 | Yes | Yes | No | -0.167 | 0.29 | -0.07 | -2.571 | No | No | No | No | No | No | No | -0.068 | No | No | 0.108 | No | No | 2.673 | 1.238 | No |
| CHEMBL2105796 | O=C(CNC(=O)c1ccc(Cl)cc1)NO | 228.635 | 0.5752 | 3 | 3 | 3 | 91.001 | -2.217 | 0.399 | 78.985 | -3.562 | No | No | No | -0.444 | 0.532 | -0.856 | -3.399 | No | No | No | No | No | No | No | -0.041 | No | No | 0.447 | No | No | 2.366 | 1.878 | No |
| CHEMBL73151 | CCN(CC)C(=O)N[C@H]1C[C@@H]2c3cccc4[nH]cc(c34)C[C@H]2N(C)C1 | 340.471 | 2.9317 | 3 | 2 | 2 | 148.921 | -3.106 | 0.906 | 90.306 | -2.761 | Yes | No | No | 1.633 | 0.596 | 0.185 | -2.19 | No | Yes | No | No | No | Yes | No | 0.863 | No | No | -0.841 | No | Yes | 3.107 | 0.537 | Yes |
| CHEMBL24955 | CN1C(=O)CC[C@@H]2c3ccc(Cl)cc3CC[C@H]21 | 249.741 | 2.9906 | 0 | 1 | 0 | 106.564 | -3.751 | 1.399 | 93.646 | -2.573 | No | No | No | 0.409 | 0.248 | 0.226 | -1.918 | No | Yes | No | Yes | No | No | No | -0.016 | No | No | 0.17 | No | No | 2.63 | 1.153 | No |
| CHEMBL409038 | Nc1nccc(-c2ccc3noc(-c4ccc(Cl)cc4)c3c2)n1 | 322.755 | 4.1874 | 2 | 5 | 1 | 136.275 | -3.864 | 1.115 | 94.139 | -2.727 | No | No | Yes | 0.211 | 0.296 | -0.756 | -1.886 | No | Yes | Yes | Yes | Yes | No | Yes | 0.001 | No | Yes | 0.462 | No | No | 2.596 | 0.378 | Yes |
| CHEMBL3644452 | N[C@H]1C[C@@H](N2Cc3c[nH]nc3C2)CSC1c1cc(F)cc(F)c1F | 354.401 | 2.7167 | 2 | 4 | 2 | 141.148 | -3.447 | 1.267 | 89.478 | -3.616 | Yes | No | No | 1.093 | 0.432 | 0.389 | -3.306 | No | Yes | No | No | No | No | No | 0.691 | No | No | -0.53 | No | No | 2.912 | 0.889 | Yes |
| CHEMBL3644458 | CS(=O)(=O)n1cc2c(n1)CN([C@H]1CSC(c3cc(F)ccc3F)[C@@H](N)C1)C2 | 414.503 | 1.8587 | 3 | 7 | 1 | 160.318 | -2.518 | 1.3 | 91.133 | -2.742 | Yes | No | No | 0.733 | 0.622 | -0.741 | -2.675 | No | No | No | No | No | No | No | 0.416 | No | No | -0.497 | No | No | 2.737 | 0.703 | Yes |
| CHEMBL3039507 | CCOc1ccc(Cc2cc([C@@H]3O[C@H](SC)[C@@H](O)[C@H](O)[C@H]3O)ccc2Cl)cc1 | 424.946 | 3.1726 | 6 | 6 | 3 | 173.803 | -5.057 | 1.101 | 95.173 | -2.788 | Yes | Yes | No | -0.69 | 0.027 | -1.156 | -2.765 | No | Yes | No | No | Yes | No | No | 0.091 | No | No | -0.068 | No | Yes | 2.563 | 2.898 | No |
| CHEMBL4594275 | N#CCC1CCN(c2nc(Nc3ccc(N4CCC(O)CC4)cc3)c3c(=O)[nH]ncc3n2)CC1 | 460.542 | 2.54788 | 5 | 9 | 3 | 196.803 | -3.249 | 1.099 | 74.229 | -2.742 | Yes | Yes | Yes | 0.489 | 0.053 | -1.541 | -2.901 | No | Yes | No | No | No | No | Yes | 0.06 | No | No | 0.315 | No | Yes | 2.903 | 2.332 | Yes |
| CHEMBL1084617 | COc1cccc(C(=O)Nc2ccc(OCCN3CCOCC3)c(-c3ccnn3C)c2)c1 | 436.512 | 3.059 | 8 | 7 | 1 | 187.243 | -4.409 | 1.224 | 94.625 | -2.788 | Yes | Yes | Yes | 0.632 | 0.064 | -0.593 | -2.882 | No | Yes | No | No | Yes | No | Yes | 0.692 | Yes | No | 0.131 | No | Yes | 2.724 | 0.637 | Yes |
| CHEMBL4297378 | CC1=C[C@@H]2CN(C(=O)N2OS(=O)(=O)[O-])[C@@H]1C(N)=O.[Na+] | 299.24 | -4.6978 | 3 | 6 | 1 | 131.094 | -2.052 | 0.12 | 9.993 | -2.735 | No | No | No | -1.07 | 0.796 | -1.328 | -3.412 | No | No | No | No | No | No | No | 0.874 | No | No | 1.243 | No | No | 1.857 | 2.33 | Yes |
| CHEMBL488025 | CC1=C(CC(=O)O)c2cc(F)ccc2/C1=C\c1ccc(S(C)(=O)=O)cc1 | 372.417 | 4.0316 | 4 | 3 | 1 | 150.77 | -5.034 | 0.697 | 96.301 | -2.732 | Yes | No | No | -1.141 | 0 | -0.235 | -2.025 | No | Yes | Yes | No | No | No | No | 0.511 | No | No | 0.361 | No | No | 3.085 | 1.894 | Yes |
| CHEMBL1744447 | CC(C)c1nc(N(C)S(C)(=O)=O)nc(-c2ccc(F)cc2)c1/C=C/[C@@H](O)C[C@@H](O)CC(=O)[O-].CC(C)c1nc(N(C)S(C)(=O)=O)nc(-c2ccc(F)cc2)c1/C=C/[C@@H](O)C[C@@H](O)CC(=O)[O-].[Ca+2] | 1001.154 | 1.7532 | 20 | 16 | 4 | 418.443 | -2.892 | -0.875 | 1.271 | -2.735 | No | No | No | -0.341 | 0.355 | -3.286 | -5.012 | No | Yes | No | No | No | No | No | 0.108 | No | No | 0.423 | No | No | 2.485 | 2.41 | Yes |
| CHEMBL572284 | COc1ccc(/C=C\c2cc(OC)c(OC)c(OC)c2)cc1NC(=O)[C@@H](N)CO | 402.447 | 2.1495 | 9 | 7 | 3 | 169.383 | -3.702 | 0.308 | 71.961 | -2.993 | Yes | Yes | Yes | -0.519 | 0.306 | -1.113 | -2.956 | No | Yes | No | Yes | Yes | No | No | 0.772 | No | Yes | 0.902 | No | Yes | 1.958 | 1.643 | No |
| CHEMBL1144 | CC[C@H](C)C(=O)O[C@H]1C[C@H](O)C=C2C=C[C@H](C)[C@H](CC[C@@H](O)C[C@@H](O)CC(=O)O)[C@H]21 | 424.534 | 2.4404 | 10 | 6 | 4 | 177.991 | -3.015 | 0.615 | 39.495 | -2.735 | Yes | No | No | -0.833 | 0.398 | -1.314 | -3.41 | No | No | No | No | No | No | No | 1.25 | No | No | 0.255 | No | No | 2.347 | 2.559 | No |
| CHEMBL402548 | CO[C@@H](C(=O)N1Cc2n[nH]c(NC(=O)c3ccc(N4CCN(C)CC4)cc3)c2C1)c1ccccc1 | 474.565 | 2.6437 | 6 | 6 | 2 | 204.212 | -3.653 | 1.008 | 77.371 | -2.735 | Yes | Yes | No | 0.374 | 0.076 | -0.967 | -2.916 | No | Yes | Yes | Yes | No | No | No | 0.954 | No | No | 0.161 | No | Yes | 3.006 | 1.189 | Yes |
| CHEMBL2103847 | CC(C)C[C@@H](C(=O)N[C@H](C(=O)OC1CCCC1)c1ccccc1)[C@H](O)C(=O)NO | 406.479 | 1.8583 | 9 | 6 | 4 | 170.14 | -2.908 | 0.842 | 56.571 | -2.802 | Yes | Yes | No | -0.949 | 0.233 | -1.274 | -3.688 | No | No | No | No | No | No | No | 1.409 | No | No | 0.59 | No | No | 2.694 | 3.712 | Yes |
| CHEMBL458875 | Cc1cn([C@H]2O[C@@H](CO)[C@H](O)[C@H]2F)c(=O)[nH]c1=O | 260.221 | -1.56628 | 2 | 6 | 3 | 100.455 | -2.196 | -0.036 | 47.03 | -2.787 | No | No | No | -0.005 | 0.712 | -0.972 | -3.646 | No | No | No | No | No | No | No | 0.661 | No | No | 0.86 | No | No | 2.044 | 3.033 | Yes |
| CHEMBL2216859 | CC(C)N1CCN(Cc2cnc(-c3cc(-c4cccc5[nH]ccc45)cc4[nH]ncc34)o2)CC1 | 440.551 | 4.8922 | 5 | 5 | 2 | 191.963 | -3.188 | 1.128 | 93.462 | -2.735 | Yes | No | Yes | 0.642 | 0.111 | -1.107 | -2.096 | Yes | Yes | Yes | Yes | No | No | Yes | 0.879 | No | Yes | 0.624 | No | Yes | 3.144 | 0.892 | Yes |
| CHEMBL4297063 | CC(O)C(=O)O.CN1CCN(c2ccc3nc(-c4c(N)c5c(F)cccc5[nH]c4=O)[nH]c3c2)CC1.O | 500.531 | 1.1716 | 3 | 7 | 5 | 204.837 | -2.897 | -1.073 | 34.558 | -2.735 | Yes | No | No | 0.346 | 0.233 | -1.578 | -3.777 | No | No | No | No | No | No | No | 0.579 | No | No | 0.42 | No | Yes | 2.446 | 2.978 | Yes |
| CHEMBL1206690 | CCC(=O)NS(=O)(=O)c1ccc(-c2c(-c3ccccc3)noc2C)cc1 | 370.43 | 3.53192 | 5 | 5 | 1 | 151.443 | -4.847 | 1.129 | 96.316 | -2.747 | No | Yes | Yes | -0.223 | 0.15 | -0.737 | -2.414 | No | Yes | Yes | Yes | Yes | No | Yes | 0.865 | No | No | 0.31 | No | Yes | 2.606 | 1.123 | Yes |
| CHEMBL4297624 | CCOC(=O)C12CCC(NCC(=O)N3C[C@@H](F)C[C@H]3C#N)(CC1)CC2 | 351.422 | 1.69468 | 5 | 5 | 1 | 147.23 | -3.498 | 0.725 | 95.05 | -3.431 | No | No | No | 0.323 | 0.643 | -0.653 | -3.168 | No | No | No | No | No | No | No | 1.293 | No | No | -0.519 | No | No | 2.944 | 0.196 | Yes |
| CHEMBL515606 | CCOC(=O)[C@H](CCc1ccccc1)N[C@H]1CCCN2CCC[C@@H](C(=O)O)N2C1=O | 417.506 | 1.5955 | 8 | 6 | 2 | 176.691 | -2.662 | 0.608 | 51.709 | -2.73 | Yes | No | No | -0.083 | 0.697 | -0.524 | -3.122 | No | Yes | No | No | No | No | No | 0.595 | No | No | -0.38 | No | No | 2.557 | 1.799 | Yes |
| CHEMBL3989406 | C[C@H](N[C@@H](CCc1ccccc1)C(=O)O)C(=O)N1CCC[C@H]1C(=O)O.O.O | 384.429 | -0.5233 | 8 | 4 | 3 | 157.149 | -2.626 | -0.485 | 11.401 | -2.735 | Yes | No | No | -2.387 | 0.532 | -0.939 | -3.752 | No | No | No | No | No | No | No | 0.719 | No | No | 0.574 | No | No | 1.774 | 2.91 | Yes |
| CHEMBL3905910 | CN1CC[C@@H](c2c(O)cc(O)c3c(=O)cc(-c4ccc(C(F)(F)F)cc4Cl)oc23)[C@@H]1CO | 469.843 | 4.3234 | 3 | 6 | 3 | 184.628 | -4.281 | 0.763 | 84.9 | -2.744 | Yes | Yes | Yes | 0.714 | 0.098 | -1.525 | -2.224 | No | Yes | Yes | No | Yes | No | No | 0.32 | No | No | 0.504 | No | Yes | 2.667 | 2.341 | Yes |
| CHEMBL1200534 | CCOC(=O)[C@H](CCc1ccccc1)N[C@@H](C)C(=O)N1Cc2cc(OC)c(OC)cc2C[C@H]1C(=O)O.Cl | 535.037 | 3.0061 | 11 | 7 | 2 | 223.039 | -3.198 | 0.363 | 58.076 | -2.735 | Yes | No | No | -0.379 | 0.403 | -1.099 | -3.455 | No | No | No | No | No | No | No | 1.318 | No | No | 0.406 | No | No | 2.258 | 2.472 | Yes |
| CHEMBL1213165 | C[C@@H]1CCO[C@H]2Cn3cc(C(=O)NCc4ccc(F)cc4F)c(=O)c([O-])c3C(=O)N21.[Na+] | 441.366 | -2.2752 | 3 | 6 | 1 | 197.637 | -3.148 | 1.176 | 60.972 | -2.91 | Yes | No | No | -0.633 | 0.222 | -1.026 | -3.21 | No | Yes | No | No | No | No | No | 0.752 | No | No | 0.08 | No | No | 1.92 | 1.653 | Yes |
| CHEMBL188944 | CC(C)C(NC(=O)[C@H](C)C[C@H](O)C(Cc1ccccc1)NC(=O)OC(C)(C)C)C(N)=O | 435.565 | 2.1356 | 10 | 5 | 4 | 184.467 | -3.297 | -0.029 | 53.189 | -2.813 | Yes | Yes | No | -0.38 | 0.24 | -0.965 | -3.044 | No | Yes | No | No | No | No | No | 1.038 | No | No | -0.297 | No | Yes | 3.356 | 2.748 | No |
| CHEMBL3544911 | COc1cccc(OCCCN)c1-c1cc(Nc2cnc(C#N)cn2)n[nH]1 | 365.397 | 2.21818 | 8 | 8 | 3 | 155.716 | -2.834 | 0.185 | 67.668 | -2.735 | Yes | No | No | 1.74 | 0.299 | -1.578 | -3.619 | No | No | No | No | No | No | No | 0.711 | No | No | 0.495 | No | No | 2.479 | 2.117 | Yes |
| CHEMBL2051970 | C[C@H](CSC(=O)C(C)(C)C)C(=O)N(CC(=O)O)C1CCCC1 | 329.462 | 2.7842 | 6 | 4 | 1 | 136.369 | -2.425 | 0.827 | 91.648 | -2.735 | No | No | No | -0.843 | 0.413 | -0.467 | -2.984 | No | No | No | No | No | No | No | 0.353 | No | No | 0.571 | No | No | 2.163 | 0.871 | No |
| CHEMBL280728 | O=C(O)CCCCO/N=C(/c1cccnc1)c1cccc(C(F)(F)F)c1 | 366.339 | 4.1243 | 8 | 4 | 1 | 147.678 | -4.469 | 0.603 | 91.541 | -2.742 | Yes | No | No | -0.556 | 0.173 | -0.961 | -2.501 | No | No | Yes | No | No | No | No | 0.377 | No | No | 0.264 | No | No | 2.82 | 1.273 | Yes |
| CHEMBL1983865 | CCCCCOc1ccc(C2SC(=O)NC2=O)cc1OCC | 323.414 | 3.6784 | 8 | 5 | 1 | 134.298 | -4.486 | 0.964 | 91.51 | -3.212 | No | Yes | No | -0.001 | 0.138 | -0.297 | -2.569 | No | Yes | No | Yes | Yes | No | No | 0.118 | No | No | 0.653 | No | No | 2.53 | 1.598 | No |
| CHEMBL3644451 | N[C@H]1C[C@@H](N2Cc3c[nH]nc3C2)CSC1c1cc(F)ccc1F | 336.411 | 2.5776 | 2 | 4 | 2 | 136.983 | -3.381 | 1.267 | 90.195 | -3.58 | Yes | No | No | 1.161 | 0.433 | 0.141 | -2.31 | Yes | Yes | No | No | No | No | No | 0.724 | No | Yes | -0.633 | No | Yes | 2.893 | 1.119 | Yes |
| CHEMBL1201387 | C[C@H](N[C@@H](CCc1ccccc1)C(=O)O)C(=O)N1[C@H](C(=O)O)C[C@H]2CCCC[C@@H]21 | 402.491 | 2.2948 | 8 | 4 | 3 | 170.642 | -2.678 | 0.688 | 28.34 | -2.735 | Yes | No | No | -2.066 | 0.404 | -0.951 | -2.991 | No | No | No | No | No | No | No | 0.364 | No | No | 0.574 | No | No | 1.796 | 2.795 | Yes |
| CHEMBL1697842 | CCC(c1ccc2cc(OC)ccc2c1)C(C)(C)C(=O)O | 286.371 | 4.4528 | 5 | 2 | 1 | 125.102 | -4.579 | 1.393 | 94.493 | -2.721 | No | No | No | -0.813 | 0.032 | 0.179 | -2.021 | No | No | No | No | No | No | No | 0.245 | No | No | 0.682 | No | No | 2.522 | 1.896 | No |
| CHEMBL3644456 | CS(=O)(=O)n1cc2c(n1)CN([C@H]1CSC(c3cc(F)cc(F)c3F)[C@@H](N)C1)C2 | 432.493 | 1.9978 | 3 | 7 | 1 | 164.484 | -2.571 | 1.126 | 90.805 | -2.736 | Yes | No | No | 0.694 | 0.633 | -0.926 | -3.077 | No | No | No | No | No | No | No | 0.385 | No | No | -0.664 | No | No | 2.56 | 1.724 | Yes |
| CHEMBL277689 | O=C(O)CCC/C=C(\c1ccc(CCNS(=O)(=O)c2ccc(Cl)cc2)cc1)c1cccnc1 | 485.005 | 4.9426 | 11 | 4 | 2 | 198.565 | -4.855 | 0.247 | 71.552 | -2.749 | No | No | No | -0.935 | 0.014 | -0.931 | -2.684 | No | Yes | No | No | No | No | No | -0.327 | No | No | 0.154 | No | No | 2.553 | 0.957 | Yes |
| CHEMBL132586 | CCCC(NC(=O)C(CC(C)C)NC(=O)OCc1ccccc1)C(=O)C(=O)NCc1ccccn1 | 482.581 | 2.8929 | 13 | 6 | 3 | 205.591 | -4.801 | 0.669 | 66.61 | -2.881 | Yes | Yes | Yes | -0.584 | 0.03 | -1.339 | -3.71 | No | Yes | No | No | No | No | Yes | 1.543 | No | No | 0.237 | No | No | 2.67 | 2.192 | Yes |
| CHEMBL3652780 | CC(C)n1cc(C#N)c2cc(-n3cc(C(=O)O)cn3)ccc21 | 294.314 | 2.97778 | 3 | 5 | 1 | 126.37 | -4.092 | 1.207 | 99.861 | -2.727 | Yes | No | No | -0.887 | 0.189 | -0.518 | -2.834 | No | No | No | No | No | No | No | 0.832 | No | No | 0.603 | No | No | 2.507 | 1.982 | Yes |
| CHEMBL4536936 | CCCOc1ccc(/C=C2/SC(=O)NC2=O)cc1 | 263.318 | 2.7993 | 4 | 4 | 1 | 109.4 | -3.633 | 1.335 | 92.477 | -3.089 | No | Yes | No | -0.027 | 0.192 | 0.081 | -2.324 | No | No | Yes | No | No | No | No | -0.032 | No | No | 0.591 | No | No | 2.411 | 1.468 | No |
| CHEMBL206468 | OC[C@H]1CNC[C@@H](O)[C@@H]1O | 147.174 | -2.0801 | 1 | 4 | 4 | 59.491 | 0.514 | 0.546 | 65.075 | -4.323 | No | No | No | -0.257 | 0.955 | -0.703 | -3.927 | No | No | No | No | No | No | No | 0.837 | No | No | 1.765 | No | No | 1.574 | 2.697 | No |
| CHEMBL3661401 | OC(c1ccccc1)C1OC1c1ccccc1 | 226.275 | 2.8601 | 3 | 2 | 1 | 100.485 | -3.005 | 1.684 | 94.097 | -2.705 | Yes | No | No | 0.325 | 0.074 | 0.308 | -1.767 | No | Yes | Yes | Yes | No | No | No | 0.13 | Yes | Yes | 0.448 | No | No | 1.708 | 2.162 | No |
| CHEMBL3661414 | OCC1OC1c1ccccc1 | 150.177 | 1.1188 | 2 | 2 | 1 | 65.428 | -0.905 | 1.604 | 80.627 | -2.999 | No | No | No | 0.116 | 0.412 | -0.1 | -2.325 | No | No | No | No | No | No | No | 0.188 | No | Yes | 0.81 | No | No | 1.849 | 2.531 | No |
| CHEMBL269455 | O=C1NC(=O)C2(N1)c1cc(F)ccc1-c1ccc(F)cc12 | 286.237 | 2.0282 | 0 | 2 | 2 | 117.326 | -4.057 | 1.239 | 95.494 | -2.737 | Yes | No | No | 0.272 | 0.152 | -0.393 | -2.259 | No | No | Yes | No | No | No | No | -0.033 | No | Yes | -0.151 | No | Yes | 2.093 | 1.546 | Yes |
| CHEMBL3545187 | O=C(/C=C\n1cnc(-c2cc(C(F)(F)F)cc(C(F)(F)F)c2)n1)NNc1ccccn1 | 442.323 | 3.9917 | 5 | 6 | 2 | 169.952 | -5.701 | 0.652 | 85.31 | -2.864 | Yes | Yes | No | -1.033 | 0.178 | -1.588 | -2.911 | No | Yes | Yes | Yes | Yes | No | Yes | -0.151 | No | Yes | 0.424 | No | Yes | 3.112 | 0.794 | No |
| CHEMBL3422109 | N#CCCNc1nonc1-c1nc2ccccc2n1CC(=O)c1ccc(N)cc1 | 387.403 | 2.87698 | 7 | 9 | 2 | 165.271 | -2.924 | 0.543 | 86.991 | -2.735 | Yes | Yes | No | -0.253 | 0.279 | -0.952 | -2.802 | No | No | Yes | Yes | Yes | No | Yes | 0.82 | Yes | Yes | 0.354 | No | Yes | 2.398 | 1.543 | Yes |
| CHEMBL1200831 | CCOC(=O)[C@H](CCc1ccccc1)N[C@@H](C)C(=O)N1CC2(C[C@H]1C(=O)O)SCCS2.Cl | 503.086 | 2.8126 | 9 | 7 | 2 | 203.36 | -3.028 | -0.063 | 48.27 | -2.735 | Yes | No | No | -0.61 | 0.528 | -0.922 | -2.838 | No | Yes | No | No | No | No | No | 0.899 | No | No | -0.025 | No | No | 2.249 | 2.035 | Yes |
| CHEMBL186528 | CCC[C@H](C[C@H](O)C(Cc1ccccc1)NC(=O)OC(C)(C)C)C(=O)NC(C(N)=O)C(C)C | 463.619 | 2.9158 | 12 | 5 | 4 | 197.197 | -3.792 | 0.052 | 54.868 | -2.702 | Yes | Yes | Yes | -0.205 | 0.203 | -0.871 | -2.927 | No | Yes | No | No | No | No | Yes | 1.088 | No | No | -0.706 | No | Yes | 3.226 | 2.809 | No |
| CHEMBL70620 | CSCC[C@H](NC(=O)c1ccc(NC(=O)[C@@H](N)CS)cc1)C(=O)O | 371.484 | 0.8183 | 9 | 6 | 5 | 149.712 | -2.657 | -0.181 | 32.317 | -2.735 | Yes | No | No | -1.212 | 0.613 | -1.387 | -3.932 | No | No | No | No | No | No | No | 0.938 | No | No | 0.929 | No | No | 1.853 | 2.194 | Yes |
| CHEMBL3644460 | N#Cc1[nH]nc2c1CN([C@H]1CSC(c3cc(F)ccc3F)[C@@H](N)C1)C2 | 361.421 | 2.44928 | 2 | 5 | 2 | 147.74 | -2.658 | 0.967 | 90.538 | -2.735 | Yes | No | No | 0.95 | 0.588 | -1.11 | -2.446 | No | Yes | No | No | No | No | No | 0.511 | No | No | -0.526 | No | No | 3.175 | 1.116 | Yes |
| CHEMBL154580 | C=CC(=O)c1ccc2ccccc2c1 | 182.222 | 3.2085 | 2 | 1 | 0 | 82.68 | -3.707 | 1.369 | 96.836 | -1.974 | Yes | No | No | 0.461 | 0.086 | 0.479 | -1.568 | No | Yes | Yes | No | No | No | Yes | 0.248 | No | No | 0.766 | No | No | 2.015 | 1.278 | No |
| CHEMBL2170440 | N#Cc1ccccc1Nc1ccnc(Nc2ccc(C(=O)O)cc2)n1 | 331.335 | 3.53368 | 5 | 6 | 3 | 143.163 | -3.702 | 0.442 | 66.028 | -2.735 | Yes | No | No | -0.816 | 0.086 | -1.08 | -2.245 | No | No | Yes | No | No | No | No | -0.022 | No | No | 0.844 | No | No | 3.037 | 1.647 | Yes |
| CHEMBL359790 | CC(C)C(NC(=O)CC[C@H](O)C(Cc1ccccc1)NC(=O)OC(C)(C)C)C(N)=O | 421.538 | 1.8896 | 10 | 5 | 4 | 178.102 | -3.222 | -0.102 | 52.967 | -2.816 | Yes | Yes | No | -0.419 | 0.24 | -0.962 | -3.109 | No | Yes | No | No | No | No | No | 1.094 | No | No | -0.325 | No | Yes | 3.368 | 2.768 | No |
| CHEMBL4650323 | C=CC(=O)N1CCC[C@@H](n2c(=O)n(-c3ccc(Oc4ccccc4)cc3)c3c(N)nccc32)C1 | 455.518 | 3.9112 | 5 | 7 | 1 | 196.056 | -3.01 | 0.994 | 99.813 | -2.735 | Yes | Yes | Yes | 0.353 | 0.195 | -0.814 | -2.518 | No | Yes | No | Yes | Yes | No | Yes | 0.337 | Yes | Yes | -0.066 | No | Yes | 2.604 | 0.259 | Yes |
| CHEMBL4297277 | CC(/C=C1\SC(=S)N(CC(=O)O)C1=O)=C\c1ccccc1.CC(/C=C1\SC(=S)N(CC(=O)[O-])C1=O)=C\c1ccccc1.C[N+](C)(C)CCO | 741.979 | 4.1876 | 10 | 10 | 2 | 305.606 | -3.805 | 0.055 | 24.355 | -2.735 | Yes | No | Yes | -1.429 | 0.118 | -1.267 | -2.943 | No | Yes | No | No | No | No | No | -0.849 | No | No | 0.186 | No | No | 1.738 | 1.198 | Yes |
| CHEMBL73622 | CC1(C)S[C@@H]2[C@H](Br)C(=O)N2[C@H]1C(=O)O | 280.143 | 0.8968 | 1 | 3 | 1 | 94.15 | -1.557 | 1.181 | 91.585 | -2.735 | No | No | No | -0.864 | 0.646 | -0.281 | -3.063 | No | No | No | No | No | No | No | 0.267 | No | No | 1.246 | No | No | 2.532 | 1.421 | Yes |
| CHEMBL2035187 | C1=C/COCc2cc(ccc2OCCN2CCCC2)Nc2nccc(n2)-c2cccc(c2)COC/1 | 472.589 | 4.9648 | 4 | 7 | 1 | 206.313 | -4.006 | 1.244 | 94.059 | -2.74 | Yes | Yes | Yes | 0.889 | 0.182 | -0.872 | -2.123 | No | Yes | No | No | Yes | No | Yes | 0.519 | Yes | No | 0.518 | No | Yes | 3.166 | 1.843 | Yes |
| CHEMBL3987016 | CCS(=O)(=O)Nc1ccc(Oc2ccc(F)cc2F)c(-c2cn(C)c(=O)c3[nH]ccc23)c1 | 459.474 | 4.3657 | 6 | 5 | 2 | 181.878 | -4.02 | 0.543 | 100 | -2.735 | Yes | Yes | Yes | -0.443 | 0.222 | -0.623 | -3.772 | No | Yes | Yes | Yes | Yes | No | Yes | 0.739 | No | No | 0.688 | No | Yes | 3.038 | 1.436 | Yes |
| CHEMBL4297389 | O=C1[C@H](C/C=C(\CO)c2ccc(F)cc2)[C@@H](c2ccc(O)cc2)N1c1ccc(F)cc1 | 421.443 | 4.8405 | 6 | 3 | 2 | 178.246 | -5.06 | 1.149 | 94.349 | -2.741 | Yes | Yes | Yes | -0.429 | 0.177 | -0.694 | -2.096 | No | Yes | No | Yes | Yes | No | Yes | -0.205 | No | No | 0.525 | No | Yes | 2.409 | 1.543 | Yes |
| CHEMBL3989939 | O=C(Nc1cnn2ccc(N3CCC[C@@H]3c3cc(F)ccc3F)nc12)N1CC[C@H](O)C1.O=S(=O)(O)O | 526.522 | 2.2947 | 3 | 8 | 4 | 204.281 | -2.849 | -0.782 | 35.167 | -2.735 | Yes | No | No | -0.737 | 0.237 | -2.486 | -3.73 | No | Yes | No | No | No | No | No | 0.091 | No | No | 0.632 | No | No | 2.409 | 2.627 | Yes |
| CHEMBL3989870 | CNCc1ccc(-c2cc(-c3nc(-c4ccc(S(=O)(=O)C(C)C)cc4)cnc3N)on2)cc1 | 463.563 | 3.9493 | 7 | 8 | 2 | 192.347 | -3.707 | 0.669 | 91.931 | -2.736 | Yes | Yes | Yes | 0.643 | 0.153 | -0.901 | -2.444 | No | Yes | No | Yes | Yes | No | Yes | 0.66 | No | No | 0.364 | No | Yes | 2.852 | 1.858 | Yes |
| CHEMBL1201087 | C=CCN1C[C@H](C(=O)N(CCCN(C)C)C(=O)NCC)C[C@@H]2c3cccc4[nH]cc(c34)C[C@H]21 | 451.615 | 3.1939 | 8 | 4 | 2 | 196.342 | -3.413 | 0.828 | 95.064 | -2.756 | Yes | Yes | Yes | 2.082 | 0.371 | -0.429 | -2.666 | No | Yes | No | No | No | Yes | Yes | 0.795 | No | Yes | -0.941 | No | Yes | 3.28 | 0.727 | No |
| CHEMBL4594405 | Cc1cc(C)c(CNC(=O)c2cc(Cl)c3c(c2C)O[C@@](C)([C@H]2CC[C@H](N(C)C)CC2)O3)c(=O)[nH]1.Cc1ccc(S(=O)(=O)O)cc1 | 660.233 | 5.73308 | 6 | 7 | 3 | 269.39 | -3.799 | -0.269 | 54.034 | -2.735 | Yes | No | No | -0.036 | 0.101 | -1.667 | -2.917 | No | Yes | No | No | No | No | No | 0.45 | No | No | 0.938 | No | No | 3.094 | 2.068 | Yes |
| CHEMBL293743 | CC(C#Cc1cccc(Oc2ccc(F)cc2)c1)N(O)C(N)=O | 314.316 | 3.1279 | 3 | 3 | 2 | 132.601 | -4.584 | 0.971 | 92.827 | -2.789 | Yes | No | No | -0.444 | 0.011 | -0.585 | -2.285 | No | Yes | Yes | Yes | Yes | No | Yes | 0.25 | No | Yes | 0.473 | No | No | 3.152 | 2.756 | No |
| CHEMBL2103775 | CC(=O)SC[C@@H](C)C(=O)N1CCC[C@H]1C(=O)N[C@@H](Cc1ccccc1)C(=O)O | 406.504 | 1.7053 | 8 | 5 | 2 | 168.408 | -2.648 | 0.394 | 43.334 | -2.735 | Yes | No | No | -1.886 | 0.383 | -0.799 | -3.051 | No | No | No | No | No | No | No | 0.183 | No | No | 0.537 | No | No | 1.871 | 2.018 | Yes |
| CHEMBL3982723 | CC1(N)CCN(c2cccnc2NC(=O)c2nc(-c3ncccc3C(F)(F)F)cnc2N)CC1 | 472.475 | 3.1045 | 4 | 8 | 3 | 192.985 | -2.819 | 1.508 | 71.916 | -2.735 | Yes | Yes | No | 1.126 | 0.215 | -1.444 | -3.253 | No | No | No | No | No | No | No | 0.395 | No | No | 0.484 | No | Yes | 2.519 | 1.419 | Yes |
| CHEMBL4116008 | CCS(=O)(=O)N1CCN(c2ccc(Nc3ncc(C(N)=O)c(NC4CC4)n3)cc2)CC1 | 445.549 | 1.3651 | 8 | 8 | 3 | 181.447 | -3.277 | 0.205 | 84.233 | -2.854 | Yes | No | No | -0.18 | 0.252 | -1.372 | -3.639 | No | No | No | No | No | No | Yes | 0.177 | No | No | 0.155 | No | Yes | 2.313 | 0.931 | Yes |
| CHEMBL1232461 | CCNC(=O)C[C@@H]1N=C(c2ccc(Cl)cc2)c2cc(OC)ccc2-n2c(C)nnc21 | 423.904 | 3.65602 | 5 | 6 | 1 | 179.31 | -4.778 | 1.036 | 98.261 | -2.726 | No | Yes | Yes | 0.305 | 0.145 | -0.913 | -2.379 | No | Yes | Yes | Yes | Yes | No | Yes | 0.249 | Yes | No | -0.461 | No | Yes | 3.049 | 1.091 | Yes |
| CHEMBL184 | Nc1nc(O)c2ncn(COCCO)c2n1 | 225.208 | -0.9195 | 4 | 8 | 3 | 90.328 | -1.307 | -0.135 | 51.508 | -2.735 | Yes | No | No | -0.241 | 0.586 | -1.253 | -3.32 | No | No | No | No | No | No | No | 0.98 | No | No | 0.776 | No | No | 1.323 | 2.273 | Yes |
| CHEMBL2105759 | CCS(=O)(=O)N1CC(CC#N)(n2cc(-c3ncnc4[nH]ccc34)cn2)C1 | 371.426 | 1.09568 | 5 | 7 | 1 | 149.888 | -2.798 | 0.642 | 79.611 | -2.777 | Yes | No | No | 0.025 | 0.248 | -0.757 | -3.636 | No | No | No | No | No | No | No | 0.963 | No | No | 0.034 | No | Yes | 2.368 | 1.548 | Yes |
| CHEMBL1967878 | Cc1cc(Nc2ncc(F)c(NC3C4C=CC(C4)C3C(N)=O)n2)ccc1N1CCN(C)CC1 | 451.55 | 2.50742 | 6 | 7 | 3 | 192.616 | -3.246 | 0.973 | 84.461 | -2.834 | Yes | No | No | 1.525 | 0.383 | -0.893 | -3.069 | No | Yes | No | No | No | No | Yes | 0.496 | No | No | -0.194 | No | Yes | 2.462 | 1.567 | Yes |
| CHEMBL1237119 | CCCCC[C@H](O)CC[C@@H]1[C@H]2Cc3cccc(OCC(=O)O)c3C[C@H]2C[C@H]1O | 390.52 | 3.5832 | 10 | 4 | 3 | 167.282 | -4.307 | 0.941 | 91.321 | -2.735 | Yes | No | Yes | -0.545 | 0.147 | -0.747 | -2.732 | No | No | No | No | No | No | No | 1.143 | No | No | -1.312 | No | No | 3.05 | 1.184 | No |
| CHEMBL308954 | Cc1cc(C#N)cc(C)c1Oc1nc(Nc2ccc(C#N)cc2)nc(N)c1Br | 435.285 | 4.7174 | 4 | 7 | 2 | 170.101 | -4.366 | 0.49 | 94.147 | -2.742 | Yes | Yes | Yes | -0.045 | 0.059 | -0.555 | -2.011 | No | No | Yes | Yes | Yes | No | Yes | -0.434 | No | Yes | 0.58 | No | Yes | 3.149 | 1.394 | Yes |
| CHEMBL1201011 | CCOC(=O)[C@H](CCc1ccccc1)N[C@@H](C)C(=O)N1Cc2ccccc2C[C@H]1C(=O)O.Cl | 474.985 | 2.9889 | 9 | 5 | 2 | 200.082 | -3.267 | 0.268 | 49.979 | -2.735 | Yes | No | No | -0.372 | 0.402 | -0.721 | -2.829 | No | No | No | No | No | No | No | 1.132 | No | No | 0.165 | No | No | 2.515 | 2.194 | Yes |
| CHEMBL4297641 | C/C(=N\NC(=O)c1cccc(S(=O)(=O)N2CCN(C)CC2)c1)c1cc(Cl)ccc1O | 450.948 | 2.1357 | 5 | 6 | 2 | 180.357 | -3.34 | 0.98 | 77.793 | -2.809 | Yes | Yes | Yes | 0.534 | 0.152 | -1.118 | -2.667 | No | Yes | No | No | No | No | No | 0.079 | Yes | No | -0.583 | No | Yes | 2.336 | 1.631 | Yes |
| CHEMBL4117187 | COc1ccc2c(c1)c(CN1CCN(C)CC1)cn2S(=O)(=O)c1ccccc1Br | 478.412 | 3.3968 | 5 | 6 | 0 | 179.4 | -3.451 | 0.71 | 91.598 | -2.81 | Yes | Yes | Yes | 1.167 | 0.178 | 0.093 | -2.335 | No | Yes | No | No | No | No | No | 0.198 | No | No | 0.203 | No | Yes | 2.987 | 1.232 | Yes |
| CHEMBL175691 | Cc1cc(/C=C/C#N)cc(C)c1Nc1ccnc(Nc2ccc(C#N)cc2)n1 | 366.428 | 4.9891 | 5 | 6 | 2 | 163.37 | -4.66 | 0.444 | 97.621 | -2.789 | Yes | Yes | Yes | 0.352 | 0.054 | -0.064 | -1.731 | No | Yes | Yes | Yes | Yes | No | Yes | 0.168 | No | No | 0.033 | No | Yes | 2.628 | 1.044 | Yes |
| CHEMBL4297435 | COC(=O)c1nc(C(F)(F)F)n2c1CN(C(=O)C[C@H](N)Cc1cc(F)c(F)cc1F)CC2 | 464.366 | 2.4081 | 5 | 6 | 1 | 177.3 | -3.036 | 1.326 | 92.296 | -2.735 | Yes | No | No | 0.382 | 0.581 | -1.131 | -3.109 | No | No | No | No | No | No | Yes | 0.566 | No | No | 0.706 | No | No | 2.205 | 1.794 | Yes |
| CHEMBL3904602 | CC(C)N1CCN(c2ccc(Nc3ncc4cc5n(c4n3)C3(CCCCC3)CNC5=O)nc2)CC1 | 474.613 | 3.5031 | 4 | 8 | 2 | 205.486 | -3.639 | 1.164 | 92.657 | -2.831 | Yes | Yes | No | 1.146 | 0.318 | -1.087 | -3.01 | No | Yes | No | No | No | No | No | 0.814 | No | No | -0.567 | No | Yes | 3.031 | 1.283 | Yes |
| CHEMBL224060 | Cn1nnc2ccc([C@H](c3ccc(Cl)cc3)n3cncn3)cc21 | 324.775 | 2.8509 | 3 | 6 | 0 | 136.883 | -3.523 | 1.303 | 100 | -2.726 | No | No | Yes | 0 | 0.305 | 0.039 | -2.364 | No | Yes | Yes | Yes | Yes | No | Yes | 0.013 | No | Yes | 0.268 | No | Yes | 2.318 | 0.632 | Yes |
| CHEMBL2001019 | OCCn1cc(-c2cnc3nnn(Cc4ccc5ncccc5c4)c3n2)cn1 | 372.392 | 1.6736 | 5 | 9 | 1 | 158.859 | -2.648 | 0.212 | 87.92 | -2.735 | No | Yes | No | 0.355 | 0.252 | -1.506 | -3.133 | No | Yes | Yes | No | No | No | No | 0.81 | Yes | Yes | 0.354 | No | Yes | 2.09 | 0.717 | Yes |
| CHEMBL148674 | CCc1c(C(=O)C(N)=O)c2c(OCC(=O)O)cccc2n1Cc1ccccc1 | 380.4 | 2.3834 | 8 | 5 | 2 | 160.876 | -4.588 | 1.034 | 60.606 | -2.734 | Yes | No | No | -0.645 | 0.114 | -0.385 | -2.993 | No | No | No | No | No | No | No | 0.498 | No | No | 0.367 | No | No | 2.077 | 1.736 | Yes |
| CHEMBL223228 | O=C1Nc2ccc(Cl)cc2[C@@](C#CC2CC2)(C(F)(F)F)O1 | 315.678 | 4.0731 | 0 | 2 | 1 | 122.963 | -4.911 | 1.639 | 89.072 | -2.816 | Yes | Yes | No | 0.206 | 0.075 | 0.118 | -1.31 | No | Yes | Yes | Yes | No | No | No | -0.145 | No | No | -0.555 | No | No | 2.644 | 1.144 | No |
| CHEMBL545315 | C=CC(=O)Nc1cc2c(Nc3ccc(F)c(Cl)c3)ncnc2cc1OCCCN1CCOCC1.Cl.Cl | 558.869 | 5.2351 | 9 | 7 | 2 | 225.587 | -4.691 | 0.866 | 90.859 | -2.738 | Yes | Yes | Yes | 1.34 | 0.13 | -1.806 | -3.223 | No | Yes | No | No | No | No | Yes | 1.196 | Yes | No | 0.158 | No | Yes | 2.729 | 1.103 | Yes |
| CHEMBL99701 | C[C@H](N[C@@H](CCc1ccccc1)C(=O)O)C(=O)N1C(=O)N(C)C[C@H]1C(=O)O | 377.397 | 0.3977 | 8 | 5 | 3 | 156.109 | -2.716 | 0.192 | 7.93 | -2.735 | Yes | No | No | -2.343 | 0.522 | -0.963 | -3.691 | No | No | No | No | No | No | No | 0.352 | No | No | 0.729 | No | No | 1.891 | 3.49 | Yes |
| CHEMBL2106994 | CCOc1cc2c(cc1OC)C(c1ccc(C(=O)N(C(C)C)C(C)C)cc1)=N[C@@H]1CCN(C)C[C@H]21 | 477.649 | 4.9916 | 7 | 5 | 0 | 209.484 | -5.062 | 1.041 | 93.116 | -2.774 | Yes | Yes | Yes | 0.805 | 0.01 | -0.062 | -2.03 | No | Yes | No | No | No | Yes | Yes | 0.705 | No | No | -0.536 | No | Yes | 2.307 | 1.736 | Yes |
| CHEMBL2103882 | O=C1NC(=O)[C@@H](c2cn3c4c(cccc24)CCC3)[C@@H]1c1c[nH]c2ccccc12 | 369.424 | 3.5926 | 2 | 3 | 2 | 160.896 | -4.127 | 0.758 | 96.812 | -2.74 | Yes | Yes | Yes | 0.279 | 0.121 | 0.015 | -1.832 | Yes | Yes | Yes | Yes | Yes | No | Yes | 0.458 | No | Yes | 0.485 | No | Yes | 3.143 | 1.453 | Yes |
| CHEMBL2220442 | CC(C)n1c(/C=C/C(O)CC(O)CC(=O)O)c(-c2ccc(F)cc2)c2ccccc21 | 411.473 | 4.6281 | 8 | 4 | 3 | 174.259 | -3.59 | 0.902 | 97.87 | -2.735 | Yes | No | No | -1.099 | 0.042 | -1.151 | -2.465 | No | Yes | No | No | No | No | No | 0.937 | No | No | -0.181 | No | No | 2.433 | 2.803 | Yes |
| CHEMBL1770916 | CCC[C@H](N[C@H]1CCc2cc(F)cc(F)c2C1)C(=O)Nc1cn(C(C)(C)CNCC(C)(C)C)cn1 | 489.655 | 4.7864 | 10 | 5 | 3 | 207.833 | -3.064 | 0.821 | 85.5 | -2.735 | Yes | Yes | No | 0.16 | 0.332 | -1.146 | -2.549 | No | Yes | No | No | No | Yes | Yes | 0.922 | No | No | 0.24 | No | Yes | 2.613 | 1.121 | No |
| CHEMBL4594445 | CNCc1cn(S(=O)(=O)c2cccc(F)c2)c(-c2ccc(F)cc2F)c1OC | 410.417 | 3.5374 | 6 | 5 | 1 | 159.98 | -4.598 | 1.155 | 95.206 | -2.739 | No | Yes | Yes | 0.147 | 0.238 | -0.421 | -3.222 | No | Yes | Yes | Yes | No | No | Yes | -0.018 | No | No | 0.712 | No | Yes | 2.532 | 0.905 | Yes |
| CHEMBL4297477 | Cn1cc(Nc2nccc(N3C[C@@H]4CC[C@H](C3)N4C(=O)[C@@H]3CC3(F)F)n2)cn1 | 389.41 | 1.7885 | 4 | 7 | 1 | 160.094 | -4.766 | 1.094 | 95.807 | -3.141 | No | No | No | -0.203 | 0.414 | -1.076 | -3.123 | No | Yes | No | No | No | No | No | -0.009 | No | No | -0.559 | No | No | 2.549 | -0.173 | Yes |
| CHEMBL1689063 | NC(=O)[C@@H]1CC[C@@H]2CN1C(=O)N2OS(=O)(=O)O | 265.247 | -1.5253 | 3 | 5 | 2 | 97.005 | -1.937 | -0.185 | 14.97 | -2.735 | No | No | No | -0.752 | 0.783 | -1.034 | -3.339 | No | No | No | No | No | No | No | 0.546 | No | No | 1.444 | No | No | 1.834 | 2.545 | No |
| CHEMBL463981 | O=S(=O)(N[C@H](CO)C(C(F)(F)F)C(F)(F)F)c1ccc(Cl)s1 | 391.742 | 2.7815 | 5 | 4 | 2 | 129.298 | -4.145 | 0.893 | 85.879 | -3.126 | Yes | No | No | -0.857 | 0.33 | -0.042 | -3.097 | No | No | No | No | No | No | No | 0.488 | No | No | 0.23 | No | No | 3.104 | 1.686 | Yes |
| CHEMBL590799 | CN(C)C(=O)COC(=O)Cc1ccc(OC(=O)c2ccc(NC(=N)N)cc2)cc1 | 398.419 | 1.38507 | 7 | 6 | 3 | 167.455 | -3.194 | -0.197 | 60.586 | -2.735 | Yes | No | No | 0.094 | 0.117 | -1.393 | -3.284 | No | Yes | No | No | No | No | No | 0.706 | No | Yes | -0.253 | No | Yes | 2.537 | 2.94 | Yes |
| CHEMBL3545368 | CC(O)C(=O)O.Cc1[nH]c2ccccc2c1CCNCc1ccc(/C=C/C(=O)NO)cc1 | 439.512 | 2.77902 | 8 | 5 | 6 | 186.104 | -3.077 | 0.001 | 37.128 | -2.735 | Yes | No | No | 0.024 | 0.266 | -1.263 | -2.91 | Yes | Yes | No | No | No | No | No | 0.989 | No | No | 0.284 | No | Yes | 2.345 | 3.228 | Yes |
| CHEMBL3989970 | C=CC(=O)N[C@@H]1CN(c2nc(Nc3cn(C)nc3OC)c3ncn(C)c3n2)C[C@H]1F | 415.433 | 0.6779 | 6 | 10 | 2 | 171.11 | -2.195 | 0.252 | 81.211 | -2.735 | Yes | No | No | 0.474 | 0.564 | -1.942 | -3.407 | No | No | No | No | No | No | No | 0.435 | No | No | 0.837 | No | No | 2.299 | 1.482 | Yes |
| CHEMBL2387080 | Cc1nc(-c2cn3c(n2)-c2ccc(-c4cnn(C(C)(C)C(N)=O)c4)cc2OCC3)n(C(C)C)n1 | 460.542 | 3.17422 | 5 | 9 | 1 | 196.934 | -2.988 | 0.672 | 100 | -2.735 | Yes | Yes | Yes | 0.014 | 0.187 | -1.726 | -3.31 | No | Yes | No | Yes | Yes | No | Yes | 0.464 | Yes | Yes | 0.587 | No | Yes | 2.912 | 0.434 | No |
| CHEMBL3989866 | O=C(NCc1c(F)cc(F)cc1F)c1cn2c(c(O)c1=O)C(=O)N1[C@H]3CC[C@H](C3)O[C@@H]1C2 | 449.385 | 1.6344 | 3 | 6 | 2 | 178.749 | -3.307 | 0.941 | 82.418 | -2.946 | Yes | No | No | 0.055 | 0.249 | -1.676 | -3.686 | No | Yes | No | No | No | No | Yes | 0.411 | No | No | -0.051 | No | No | 2.588 | 1.734 | Yes |
| CHEMBL65067 | O=C(C(c1ccccc1)c1ccccc1)N1CCN(CC(O)COc2cccc3ncccc23)CC1 | 481.596 | 3.9508 | 8 | 5 | 1 | 211.315 | -4.418 | 1.207 | 95.764 | -2.736 | Yes | Yes | Yes | 0.508 | 0 | 0.01 | -2.477 | Yes | Yes | Yes | Yes | No | Yes | Yes | 0.616 | Yes | No | 0.195 | No | Yes | 2.49 | 1.737 | Yes |
| CHEMBL2107825 | CC(C)OC(=O)[C@H](C)N[P@](=O)(CO[C@H](C)Cn1cnc2c(N)ncnc21)Oc1ccccc1 | 476.474 | 2.9729 | 11 | 10 | 2 | 191.722 | -3.28 | -0.287 | 75.674 | -2.735 | Yes | Yes | No | 0.263 | 0.379 | -2.035 | -4.192 | No | No | No | No | Yes | No | No | 0.352 | No | No | 0.928 | No | Yes | 2.379 | 1.601 | Yes |
| CHEMBL833 | Clc1ccccc1CN1CCc2sccc2C1 | 263.793 | 3.9598 | 2 | 2 | 0 | 110.646 | -3.381 | 1.674 | 87.653 | -2.25 | No | No | No | 1.163 | 0.32 | 0.837 | -1.32 | Yes | Yes | Yes | Yes | No | Yes | No | 1.199 | No | No | 0.491 | No | No | 3.347 | 1.035 | No |
| CHEMBL1200340 | CCOC(=O)C1=C[C@@H](OC(CC)CC)[C@H](NC(C)=O)[C@@H](N)C1.O=P(O)(O)O | 410.404 | 0.3568 | 7 | 6 | 5 | 158.896 | -1.878 | 0.144 | 38.065 | -2.739 | Yes | No | No | -0.786 | 0.828 | -1.878 | -4.837 | No | No | No | No | No | No | No | 0.898 | No | No | 0.888 | No | No | 2.653 | 2.713 | Yes |
| CHEMBL4298172 | O=C(c1cccnc1-c1ccncc1)N1CCC(O)(Cc2ccccc2)CC1 | 373.456 | 3.3534 | 4 | 4 | 1 | 164.249 | -3.683 | 1.144 | 95.937 | -2.881 | Yes | Yes | Yes | 0.479 | 0.112 | 0.169 | -2.142 | No | No | Yes | Yes | No | No | Yes | 0.468 | No | No | -0.29 | No | Yes | 2.847 | 0.648 | Yes |
| CHEMBL4650366 | Cn1cc(-c2cc(S(C)(=O)=O)ccc2OCC2CC2)c2ccccc2c1=O | 383.469 | 3.3978 | 5 | 5 | 0 | 157.684 | -5.13 | 0.991 | 100 | -2.657 | No | Yes | Yes | -0.076 | 0.136 | -0.499 | -2.284 | No | Yes | Yes | Yes | Yes | No | Yes | 0.942 | No | Yes | -0.183 | No | Yes | 2.478 | 0.632 | Yes |
| CHEMBL4650314 | O=C(O)CNC(=O)c1c(O)c2ccccc2n(OCC2CC2)c1=O | 332.312 | 0.3601 | 6 | 6 | 3 | 135.782 | -2.965 | -0.037 | 51.436 | -2.782 | Yes | No | No | -0.718 | 0.216 | -1.108 | -3.624 | No | No | No | No | No | No | No | 0.222 | No | No | 0.603 | No | No | 2.246 | 2.464 | Yes |
| CHEMBL1508 | CN(C)CCC[C@@]1(c2ccc(F)cc2)OCc2cc(C#N)ccc21 | 324.399 | 3.81298 | 5 | 3 | 0 | 141.834 | -4.601 | 1.488 | 97.476 | -2.569 | No | Yes | No | 1.337 | 0.091 | 0.02 | -1.472 | No | Yes | No | No | No | Yes | No | 0.899 | Yes | No | 0.075 | No | Yes | 2.855 | 1.665 | No |
| CHEMBL4297583 | Cc1nc2cc(C(=O)N(C)C)cc(O[C@H]3CCOc4cc(F)cc(F)c43)c2[nH]1 | 387.386 | 3.75392 | 3 | 4 | 1 | 159.627 | -3.087 | 1.097 | 87.04 | -2.735 | Yes | No | No | -0.092 | 0.316 | -0.752 | -3.196 | No | No | Yes | Yes | No | No | Yes | 0.416 | Yes | No | 0.096 | No | No | 2.019 | 1.69 | Yes |
| CHEMBL110094 | CN(C)CCC=C1c2ccccc2C(C)(C)c2ccccc21 | 291.438 | 4.7093 | 3 | 1 | 0 | 133.837 | -5.189 | 1.407 | 97.761 | -2.66 | Yes | Yes | Yes | 1.736 | 0.029 | 1.011 | -1.163 | No | Yes | No | No | No | Yes | No | 0.693 | No | No | 0.26 | No | Yes | 2.802 | 1.163 | Yes |
| CHEMBL2338801 | O=c1[nH]nc(CCc2ccc(C(F)(F)F)cc2)cc1O | 284.237 | 2.2795 | 3 | 3 | 2 | 111.443 | -3.171 | 0.932 | 92.667 | -3.048 | Yes | No | No | -0.625 | 0.431 | 0.125 | -2.551 | No | Yes | Yes | No | No | No | No | 0.225 | No | No | -0.146 | No | No | 2.613 | 1.564 | No |
| CHEMBL3907479 | Cc1nc2ccc(-n3ncc(C(=O)c4cc5ccccc5[nH]4)c3N)cc2[nH]1 | 356.389 | 3.35152 | 3 | 5 | 3 | 153.385 | -2.909 | 0.627 | 93.017 | -2.735 | Yes | No | Yes | 0.009 | 0.128 | -0.81 | -2.351 | No | No | Yes | Yes | Yes | No | Yes | 1.078 | Yes | Yes | 0.462 | No | Yes | 2.405 | 2.292 | Yes |
| CHEMBL1277072 | Cc1c(/C=C2\C(=O)Nc3ccc(F)cc32)[nH]c2c1C(=O)N(C[C@H](O)CN1CCOCC1)CCC2 | 468.529 | 2.03642 | 5 | 5 | 3 | 196.931 | -2.804 | 0.607 | 72.758 | -2.743 | Yes | Yes | No | 0.96 | 0.326 | -0.876 | -2.977 | No | No | No | No | No | No | No | 1.16 | No | No | -0.144 | No | Yes | 2.38 | 2.579 | Yes |
| CHEMBL1173655 | CN(C)C/C=C/C(=O)Nc1cc2c(Nc3ccc(F)c(Cl)c3)ncnc2cc1O[C@H]1CCOC1 | 485.947 | 4.3899 | 8 | 7 | 2 | 201.28 | -4.611 | 0.886 | 93.808 | -2.751 | Yes | Yes | Yes | 1.186 | 0.107 | -1.556 | -3.255 | No | Yes | No | Yes | Yes | No | Yes | 0.633 | No | No | 0.204 | No | Yes | 2.949 | 1.219 | Yes |
| CHEMBL3622821 | CC[C@@H]1CN(C(=O)NCC(F)(F)F)C[C@@H]1c1cnc2cnc3[nH]ccc3n12 | 380.374 | 2.9079 | 3 | 4 | 2 | 152.126 | -2.85 | 1.289 | 88.897 | -2.735 | Yes | No | No | -0.001 | 0.398 | -1.315 | -3.612 | No | No | No | No | No | No | No | 0.361 | No | No | 0.283 | No | No | 2.575 | 0.067 | Yes |
| CHEMBL274654 | Cc1[nH]c(/C=C2\C(=O)Nc3ccccc32)c(C)c1CCC(=O)O | 310.353 | 3.14144 | 4 | 2 | 3 | 133.589 | -3.731 | 0.94 | 94.185 | -2.735 | Yes | No | No | -1.067 | 0.082 | -0.559 | -2.175 | No | No | No | No | No | No | No | 0.468 | No | No | 0.353 | No | No | 2.064 | 2.306 | Yes |
| CHEMBL319111 | O=C1N(c2ccccc2)c2ccccc2C1(Cc1ccncc1)Cc1ccncc1 | 391.474 | 4.8781 | 5 | 3 | 0 | 175.417 | -6.861 | 0.96 | 100 | -2.725 | Yes | Yes | Yes | 0.098 | 0.251 | 0.072 | -1.438 | No | Yes | Yes | Yes | Yes | Yes | Yes | 0.061 | No | No | 0.41 | No | Yes | 2.83 | 1.461 | Yes |
| CHEMBL4650328 | Nc1nccc(-c2ccc(O)c(-c3ccnc(N)n3)n2)n1 | 281.279 | 0.8656 | 2 | 8 | 3 | 119.118 | -2.895 | 0.441 | 68.438 | -2.736 | No | No | No | 0.109 | 0.34 | -1.486 | -3.201 | No | No | No | No | No | No | No | 0.558 | No | No | 0.408 | No | No | 2.576 | 1.596 | Yes |
| CHEMBL2338329 | Cc1nc(C(=O)NCC(=O)O)c(O)c2ccc(Oc3ccccc3)cc12 | 352.346 | 2.85552 | 5 | 5 | 3 | 148.424 | -3.966 | 1.285 | 60.514 | -2.735 | Yes | No | No | -0.705 | 0.074 | -1.107 | -3.369 | No | No | No | No | No | No | No | 0.539 | No | No | 0.68 | No | No | 2.056 | 2.023 | Yes |
| CHEMBL98 | O=C(CCCCCCC(=O)Nc1ccccc1)NO | 264.325 | 2.4711 | 8 | 3 | 3 | 112.522 | -3.153 | 0.706 | 88.314 | -3.211 | Yes | No | No | 0.12 | 0.283 | -0.679 | -2.825 | No | Yes | Yes | No | No | No | No | 0.434 | No | No | 0.363 | No | No | 2.022 | 2.405 | No |
| CHEMBL2103852 | COc1cc([C@H]2C(C#N)=C(N)Oc3c2ccc(N)c3N)cc(Br)c1OC | 417.263 | 2.84888 | 3 | 7 | 3 | 158.603 | -3.528 | -0.03 | 79.447 | -2.747 | Yes | No | No | -0.019 | 0.116 | -1.192 | -2.433 | No | No | No | Yes | Yes | No | No | 0.003 | No | No | 0.107 | No | Yes | 2.38 | 1.778 | No |
| CHEMBL1161632 | O=C(O)O | 62.024 | 0.2224 | 0 | 1 | 2 | 22.489 | 0.865 | 1.121 | 83.987 | -3.001 | No | No | No | -1.041 | 0.75 | -0.471 | -3.399 | No | No | No | No | No | No | No | 0.481 | No | No | 1.89 | No | No | 1.42 | 2.854 | No |
| CHEMBL3301599 | CC(C)c1cc(C(=O)N2Cc3ccc(CN4CCN(C)CC4)cc3C2)c(O)cc1O.C[C@H](O)C(=O)O | 499.608 | 2.5764 | 5 | 7 | 4 | 211.736 | -3.013 | 0.54 | 30.8 | -2.735 | Yes | No | No | 0.693 | 0.456 | -1.32 | -3.367 | Yes | Yes | No | No | No | No | No | 0.677 | No | No | 0.28 | No | No | 2.35 | 2.974 | Yes |
| CHEMBL3989690 | CCOC[C@@H]1[C@@H](c2ccc(Cl)c(Cl)c2)C[C@@H]2CC[C@H]1N2C | 328.283 | 4.5962 | 4 | 2 | 0 | 136.914 | -3.47 | 1.432 | 90.635 | -2.354 | No | No | No | 1.269 | 0.398 | 0.819 | -2.69 | No | Yes | Yes | No | No | Yes | No | 0.953 | Yes | No | 0.002 | No | No | 3.311 | 0.601 | No |
| CHEMBL1580 | OC[C@H]1O[C@@H](n2cnc3c2N=CNC[C@H]3O)C[C@@H]1O | 268.273 | -1.1798 | 2 | 8 | 4 | 108.919 | -2.331 | 0.063 | 64.939 | -2.736 | Yes | No | No | -0.128 | 0.782 | -1.009 | -3.341 | No | No | No | No | No | No | No | 0.789 | No | No | -0.258 | No | No | 2.137 | 1.764 | Yes |
| CHEMBL3833368 | CC1(C)[C@@H]2CC[C@@]1(CS(=O)(=O)O)C(=O)C2.CNCc1ccc(-c2[nH]c3cc(F)cc4c3c2CCNC4=O)cc1 | 555.672 | 4.2489 | 5 | 5 | 4 | 227.059 | -3.285 | 0.922 | 50.789 | -2.735 | Yes | No | No | 0.057 | 0.063 | -1.34 | -2.881 | No | Yes | Yes | No | No | No | No | 0.502 | No | No | 0.602 | No | No | 2.626 | 1.75 | Yes |
| CHEMBL3137320 | Cn1ncnc1[C@H]1c2n[nH]c(=O)c3cc(F)cc(c23)N[C@@H]1c1ccc(F)cc1 | 380.358 | 2.6285 | 2 | 6 | 2 | 156.593 | -3.091 | 0.537 | 97.933 | -2.735 | No | No | Yes | -0.542 | 0.178 | -1.546 | -3.717 | No | Yes | Yes | No | No | No | No | 0.054 | No | No | 0.521 | No | Yes | 2.157 | 2.044 | Yes |
| CHEMBL4650321 | C=CC(=O)N1CCC(c2ccc(C(N)=O)c(-c3ccc(Oc4ccccc4)cc3)n2)CC1 | 427.504 | 4.5318 | 6 | 4 | 1 | 186.891 | -5.432 | 1.026 | 93.16 | -2.755 | Yes | Yes | Yes | 0.167 | 0.081 | -0.273 | -2.157 | No | Yes | Yes | Yes | Yes | No | Yes | 0.447 | No | No | 0.205 | No | Yes | 2.599 | 0.526 | Yes |
| CHEMBL2107821 | NCC[C@@H](O)c1cccc(OCC2CCCCC2)c1 | 263.381 | 3.0279 | 6 | 3 | 2 | 115.323 | -3.165 | 1.364 | 90.938 | -3.088 | No | No | No | 0.755 | 0.232 | -0.03 | -2.253 | No | No | Yes | No | No | No | No | 1.033 | No | No | -0.39 | No | No | 2.427 | 2.208 | No |
| CHEMBL3655081 | CCCS(=O)(=O)N[C@H]1C[C@@H](N(C)c2ncnc3[nH]ccc23)C1 | 323.422 | 1.2545 | 6 | 5 | 2 | 129.746 | -3.158 | 1.283 | 81.432 | -2.951 | No | No | No | 0.102 | 0.496 | -1.026 | -3.629 | No | No | No | No | No | No | No | 1.04 | No | No | 0.17 | No | No | 2.09 | 0.678 | Yes |
| CHEMBL377300 | Cc1cc2c(F)c(Oc3ncnn4cc(OC[C@@H](C)O)c(C)c34)ccc2[nH]1 | 370.384 | 3.51844 | 5 | 6 | 2 | 153.917 | -3.216 | 1.344 | 93.766 | -2.735 | Yes | Yes | No | 0.372 | 0.107 | -0.859 | -3.146 | No | No | Yes | No | No | No | Yes | 0.792 | No | No | 0.501 | No | Yes | 2.421 | 0.968 | Yes |
| CHEMBL1352 | O=[N+]([O-])O | 63.012 | -0.3477 | 0 | 2 | 1 | 21.776 | 0.541 | 0.594 | 88.834 | -3.38 | Yes | No | No | -0.414 | 0.753 | -0.498 | -2.986 | No | No | No | No | No | No | No | 0.644 | No | No | 1.408 | No | No | 2.725 | 1.409 | No |
| CHEMBL3182314 | CC(C)(C)C(=O)Oc1ccc(S(=O)(=O)Nc2ccccc2C(=O)NCC(=O)[O-])cc1.O.O.O.O.[Na+] | 528.512 | -5.3762 | 7 | 7 | 2 | 223.997 | -3.332 | -0.096 | 11.28 | -2.735 | Yes | No | No | -1.333 | 0.007 | -1.339 | -4.603 | No | Yes | No | No | No | No | No | 0.766 | No | No | 0.105 | No | No | 1.903 | 2.435 | No |
| CHEMBL4594436 | Cc1cc([N+](=O)[O-])c(NS(=O)(=O)c2cccc(C(=O)O)c2)cc1-c1cccnc1 | 413.411 | 3.46422 | 6 | 6 | 2 | 165.414 | -3.977 | -0.361 | 71.457 | -2.735 | Yes | No | No | -1.265 | 0 | -0.93 | -2.674 | No | No | No | No | No | No | No | 0.545 | No | No | 0.956 | No | No | 2.547 | 1.901 | Yes |
| CHEMBL296913 | CCC(=O)[N-]S(=O)(=O)c1ccc(-c2c(-c3ccccc3)noc2C)cc1.[Na+] | 392.412 | 1.32002 | 5 | 5 | 0 | 179.856 | -4.34 | 0.182 | 63.975 | -2.722 | No | Yes | Yes | -0.272 | 0.159 | -1.05 | -2.883 | No | Yes | Yes | Yes | Yes | No | No | 1.333 | No | No | 0.207 | No | No | 2.706 | 0.987 | Yes |
| CHEMBL3586404 | CO[C@H]1CC[C@H](N2C(=O)CNc3ncc(-c4ccc(C(C)(C)O)nc4)nc32)CC1 | 397.479 | 2.4821 | 4 | 7 | 2 | 169.894 | -3.765 | 1.126 | 85.965 | -2.926 | No | No | No | -0.398 | 0.287 | -0.973 | -3.204 | No | Yes | No | No | No | No | No | 0.877 | No | No | -0.15 | No | No | 2.991 | 1.112 | Yes |
| CHEMBL346977 | CC(CN1c2ccccc2Sc2ccc(S(=O)(=O)N(C)C)cc21)N(C)C | 391.562 | 3.4897 | 5 | 5 | 0 | 160.201 | -3.668 | 0.906 | 90.849 | -2.729 | Yes | Yes | Yes | 1.177 | 0.396 | 0.279 | -1.817 | No | Yes | Yes | No | No | Yes | No | 0.463 | Yes | No | -0.01 | No | Yes | 3.467 | 1.528 | Yes |
| CHEMBL227875 | O=[N+]([O-])c1cn2c(n1)OC[C@@H](OCc1ccc(OC(F)(F)F)cc1)C2 | 359.26 | 2.6677 | 5 | 7 | 0 | 138.436 | -2.754 | 1.074 | 89.878 | -2.738 | Yes | No | No | -0.796 | 0.267 | -1.372 | -2.962 | No | Yes | Yes | Yes | No | No | No | 0.132 | No | Yes | 0.465 | No | No | 2.326 | 1.158 | Yes |
| CHEMBL589390 | Cc1ccc2c(c1)c1c(n2CCc2ccc(C)nc2)CCN(C)C1 | 319.452 | 3.88374 | 3 | 3 | 0 | 143.482 | -3.394 | 1.577 | 95.137 | -2.554 | Yes | Yes | Yes | 1.49 | 0.275 | 0.801 | -1.882 | Yes | Yes | Yes | No | No | Yes | Yes | 1.133 | Yes | Yes | -0.377 | No | Yes | 2.637 | 0.359 | Yes |
| CHEMBL1092581 | CNC(=O)c1c(-c2ccc(F)cc2)oc2cc(N(CCO)S(C)(=O)=O)c(C3CC3)cc12 | 446.5 | 3.2342 | 7 | 5 | 2 | 179.07 | -5.461 | 0.861 | 88.777 | -2.744 | Yes | Yes | Yes | -0.262 | 0.115 | -0.653 | -2.634 | No | Yes | No | Yes | Yes | No | Yes | 0.627 | No | Yes | -0.193 | No | Yes | 2.281 | 1.41 | Yes |
| CHEMBL4297668 | CNC(=O)c1nn(C)c2c1C(C)(C)Cc1cnc(Nc3ccc(N4CCN(C)CC4)cc3)nc1-2.O=C(O)/C=C\C(=O)O | 576.658 | 2.2776 | 6 | 10 | 4 | 243.428 | -3.045 | -0.361 | 29.307 | -2.735 | Yes | No | No | -0.38 | 0.231 | -1.501 | -3.707 | No | No | No | No | No | No | No | -0.058 | No | No | 0.755 | No | No | 2.328 | 1.88 | Yes |
| CHEMBL35033 | CCOCn1c(Cc2ccccc2)c(C(C)C)c(=O)[nH]c1=O | 302.374 | 2.2448 | 6 | 4 | 1 | 129.129 | -3.643 | 0.851 | 93.688 | -2.887 | Yes | No | No | 0.176 | 0.116 | -0.151 | -2.593 | No | Yes | Yes | Yes | No | No | No | 1.032 | No | No | 0.186 | No | Yes | 2.136 | 1.296 | Yes |
| CHEMBL3544985 | COc1c(-c2ccc3cc(NS(C)(=O)=O)ccc3c2)cc(-n2ccc(=O)[n-]c2=O)cc1C(C)(C)C.O.[Na+] | 533.582 | -0.168 | 5 | 6 | 1 | 236.708 | -4.159 | -0.126 | 66.13 | -2.735 | Yes | Yes | Yes | -1.29 | 0.288 | -1.383 | -3.226 | No | Yes | No | No | Yes | No | No | 1.118 | No | No | 0.325 | No | Yes | 3.007 | -0.664 | Yes |
| CHEMBL3286580 | Fc1ccc2c(CCNCc3cccc(OCC(F)(F)C(F)F)c3)c[nH]c2c1 | 398.375 | 4.9185 | 9 | 2 | 2 | 158.06 | -4.635 | 0.888 | 86.065 | -2.805 | Yes | Yes | Yes | 1.11 | 0.098 | 0.274 | -1.367 | Yes | Yes | Yes | Yes | No | Yes | Yes | 1.048 | Yes | No | 0.699 | No | Yes | 2.691 | 1.429 | Yes |
| CHEMBL3935857 | Nc1nc2cc(-c3ccc4ncc(C(=O)N5CCOCC5)n4c3)ccc2o1 | 363.377 | 2.1971 | 2 | 7 | 1 | 153.716 | -2.811 | 1.194 | 93.284 | -2.735 | Yes | Yes | No | 0.652 | 0.203 | -1.138 | -2.551 | No | No | Yes | Yes | No | No | Yes | 0.746 | Yes | Yes | 0.539 | No | Yes | 2.841 | 0.736 | Yes |
| CHEMBL2087361 | C#Cc1cccc(Nc2ncnc3cc4c(cc23)OCCOCCOCCO4)c1 | 391.427 | 3.1591 | 2 | 7 | 1 | 168.526 | -4.302 | 1.479 | 95.447 | -2.776 | No | Yes | Yes | -0.173 | 0.03 | -0.438 | -2.812 | No | Yes | Yes | Yes | No | No | Yes | 0.264 | No | No | 0.022 | No | Yes | 2.593 | 1.228 | Yes |
| CHEMBL2104306 | OCCN(CCO)c1nc(-c2ccccc2)c(-c2ccccc2)o1 | 324.38 | 2.7996 | 7 | 5 | 2 | 140.368 | -3.606 | 0.806 | 93.798 | -2.654 | No | No | Yes | 0.182 | 0.146 | -0.388 | -2.648 | No | Yes | Yes | Yes | Yes | No | Yes | 0.54 | No | No | 0.476 | No | No | 2.341 | 1.001 | Yes |
| CHEMBL572878 | Cc1cc(Nc2cc(N3CCN(C)CC3)nc(Sc3ccc(NC(=O)C4CC4)cc3)n2)n[nH]1 | 464.599 | 3.50322 | 7 | 8 | 3 | 196.924 | -3.241 | 0.152 | 80.718 | -2.735 | Yes | Yes | Yes | 1.525 | 0.362 | -1.629 | -2.765 | Yes | Yes | No | Yes | Yes | No | Yes | 0.645 | No | No | 0.212 | No | Yes | 2.972 | 0.812 | Yes |
| CHEMBL1214827 | CC(C)c1cc(C(=O)N2Cc3ccc(CN4CCN(C)CC4)cc3C2)c(O)cc1O | 409.53 | 3.1246 | 4 | 5 | 2 | 177.886 | -3.205 | 1.122 | 90.548 | -2.739 | Yes | No | No | 1.749 | 0.354 | 0.125 | -2.311 | No | Yes | No | No | No | Yes | No | 0.943 | No | No | 0.184 | No | Yes | 2.57 | 2.064 | Yes |
| CHEMBL371405 | C[C@@]12OC(=O)[C@]1([C@@H](O)[C@@H]1C=CCCC1)NC(=O)[C@@H]2CCCl | 313.781 | 1.1328 | 4 | 4 | 2 | 128.225 | -2.226 | -0.121 | 82.223 | -3.047 | No | No | No | 0.018 | 0.542 | -0.1 | -3.343 | No | No | No | No | No | No | No | 0.174 | No | Yes | 0.01 | No | No | 2.332 | 1.459 | No |
| CHEMBL522892 | CN1CCN(c2ccc3nc(-c4c(N)c5c(F)cccc5[nH]c4=O)[nH]c3c2)CC1 | 392.438 | 2.5445 | 2 | 5 | 3 | 165.506 | -2.912 | 0.8 | 80.981 | -2.735 | Yes | No | No | 0.401 | 0.196 | -1.036 | -2.385 | No | No | Yes | Yes | Yes | No | Yes | 0.854 | Yes | Yes | 0.391 | Yes | Yes | 2.355 | 1.523 | Yes |
| CHEMBL122 | CS(=O)(=O)c1ccc(C2=C(c3ccccc3)C(=O)OC2)cc1 | 314.362 | 2.5577 | 3 | 4 | 0 | 128.518 | -4.408 | 1.324 | 98.645 | -2.606 | No | No | No | -0.238 | 0.011 | -0.007 | -2.258 | No | Yes | Yes | Yes | Yes | No | No | 0.87 | No | Yes | 0.302 | No | No | 2.386 | 1.655 | No |
| CHEMBL2107830 | OC[C@H]1O[C@@H](c2ccc(Cl)c(Cc3ccc(O[C@H]4CCOC4)cc3)c2)[C@H](O)[C@@H](O)[C@@H]1O | 450.915 | 1.6134 | 6 | 7 | 4 | 185.312 | -3.096 | -0.035 | 59.225 | -2.765 | Yes | Yes | No | -0.445 | 0.187 | -1.18 | -3.7 | No | Yes | No | No | No | No | No | 0.402 | No | No | 0.007 | No | No | 2.548 | 3.011 | No |
| CHEMBL2408045 | CNC(=O)CN1CCC(Oc2cc3c(Nc4cccc(Cl)c4F)ncnc3cc2OC)CC1 | 473.936 | 3.7637 | 7 | 7 | 2 | 195.605 | -4.699 | 0.973 | 92.224 | -2.762 | Yes | Yes | Yes | 0.918 | 0.102 | -1.653 | -3.319 | No | Yes | No | Yes | Yes | No | Yes | 0.612 | Yes | No | 0.221 | No | Yes | 2.634 | 1.86 | Yes |
| CHEMBL279785 | CNC(=O)[C@@H](NC(=O)[C@H](CC(C)C)[C@H](O)C(=O)NO)C(C)(C)C | 331.413 | -0.2081 | 7 | 5 | 5 | 136.525 | -2.431 | -0.405 | 39.114 | -2.749 | Yes | No | No | -0.848 | 0.376 | -1.081 | -4.102 | No | No | No | No | No | No | No | 1.692 | No | Yes | 0.196 | No | No | 2.338 | 3.055 | No |
| CHEMBL12856 | Nc1cc(-c2ccncc2)c[nH]c1=O | 187.202 | 1.0191 | 1 | 3 | 2 | 80.429 | -2.943 | 0.702 | 83.4 | -3.042 | No | No | No | -0.169 | 0.522 | -0.123 | -2.89 | No | No | Yes | No | No | No | No | 0.489 | No | No | 0.15 | No | No | 2.584 | 0.919 | No |
| CHEMBL480 | Cc1c(OCC(F)(F)F)ccnc1C[S+]([O-])c1nc2ccccc2[nH]1 | 369.368 | 3.51522 | 5 | 4 | 1 | 143.983 | -2.885 | 1.299 | 84.135 | -2.735 | Yes | Yes | No | -0.047 | 0.127 | -0.572 | -2.483 | No | No | Yes | Yes | No | Yes | Yes | 0.616 | Yes | Yes | 0.024 | No | No | 2.138 | 0.786 | Yes |
| CHEMBL1442422 | CN(C)CCN1C(=O)c2ccccc2N(C)c2ccccc21 | 295.386 | 2.9763 | 3 | 3 | 0 | 131.113 | -3.579 | 1.185 | 97.686 | -2.541 | No | No | No | 1.158 | 0.149 | 0.401 | -1.576 | No | Yes | Yes | No | No | Yes | No | 0.71 | No | No | 0.083 | No | Yes | 2.814 | 0.452 | No |
| CHEMBL182 | Nc1nc(O)c2ncn(COC(CO)CO)c2n1 | 255.234 | -1.5586 | 5 | 9 | 4 | 101.487 | -1.381 | -0.951 | 44.5 | -2.735 | Yes | No | No | -0.273 | 0.592 | -1.371 | -3.798 | No | No | No | No | No | No | No | 1.003 | No | No | 0.763 | No | No | 1.325 | 2.584 | Yes |
| CHEMBL2064032 | CNCc1cc(-c2ccccc2F)n(S(=O)(=O)c2cccnc2)c1.O=C(O)/C=C/C(=O)O | 461.471 | 2.3574 | 7 | 7 | 3 | 183.078 | -3.199 | -0.316 | 27.675 | -2.735 | Yes | No | No | -0.818 | 0.252 | -1.711 | -3.732 | No | No | No | No | No | No | No | -0.195 | No | No | 1.25 | No | No | 1.971 | 1.907 | Yes |
| CHEMBL1091644 | C[C@]1(O)C[C@@H](c2nc(-c3ccc4ccc(-c5ccccc5)nc4c3)c3c(N)nccn32)C1 | 421.504 | 4.8221 | 3 | 6 | 2 | 184.957 | -2.934 | 1.129 | 92.11 | -2.735 | Yes | Yes | Yes | -0.034 | 0.229 | -0.436 | -1.985 | No | Yes | Yes | Yes | Yes | No | Yes | 0.664 | Yes | Yes | 0.205 | No | Yes | 2.457 | 0.144 | No |
| CHEMBL2107762 | CCCN(c1ccncc1F)n1cc(C)c2ccccc21 | 283.35 | 4.16352 | 4 | 3 | 0 | 123.164 | -3.914 | 1.757 | 96.733 | -2.291 | No | No | No | 0.466 | 0.23 | 0.428 | -1.648 | No | Yes | Yes | Yes | Yes | No | No | 0.29 | Yes | No | -0.087 | No | Yes | 2.143 | 1.418 | Yes |
| CHEMBL1519 | CCOC(=O)[C@H](CCc1ccccc1)N[C@@H](C)C(=O)N1[C@H](C(=O)O)C[C@H]2CCCC[C@@H]21 | 430.545 | 2.7733 | 9 | 5 | 2 | 183.691 | -2.766 | 0.228 | 51.11 | -2.735 | Yes | No | No | -0.221 | 0.652 | -0.718 | -2.921 | No | Yes | No | No | No | No | No | 0.707 | No | No | 0.014 | No | No | 2.108 | 2.013 | Yes |
| CHEMBL2218894 | CC(C)n1c(/C=C/C(O)CC(O)CC(=O)[O-])c(-c2ccc(F)cc2)c2ccccc21.[Na+] | 433.455 | 0.2974 | 8 | 5 | 2 | 202.672 | -3.454 | 0.219 | 43.867 | -2.735 | Yes | No | No | -1.134 | 0.096 | -0.193 | -3.327 | No | Yes | No | No | No | No | No | 1.249 | No | No | -0.328 | No | No | 2.392 | 1.588 | No |
| CHEMBL760 | O=C1CN2Cc3c(ccc(Cl)c3Cl)N=C2N1 | 256.092 | 1.9263 | 0 | 3 | 1 | 101.853 | -2.782 | 1.266 | 85.302 | -2.74 | No | No | No | 0.257 | 0.499 | 0.213 | -1.928 | No | Yes | Yes | No | No | No | No | 0.947 | Yes | Yes | 0.357 | No | No | 3.258 | 1.482 | No |
| CHEMBL2105762 | CS(=O)(=O)n1cc2c(n1)CN([C@H]1CO[C@H](c3cc(F)ccc3F)[C@@H](N)C1)C2 | 398.435 | 1.1421 | 3 | 7 | 1 | 155.309 | -2.385 | 1.411 | 87.73 | -2.736 | Yes | No | No | 0.645 | 0.642 | -0.339 | -3.109 | No | No | No | No | No | No | No | 0.521 | No | No | -0.441 | No | No | 2.345 | 1.687 | Yes |
| CHEMBL415 | CN(C)CCCN1c2ccccc2CCc2ccc(Cl)cc21 | 314.86 | 4.5284 | 4 | 2 | 0 | 137.86 | -4.94 | 1.335 | 94.882 | -2.394 | No | Yes | Yes | 1.723 | 0.078 | 0.915 | -1.438 | Yes | Yes | Yes | No | No | Yes | No | 0.792 | Yes | No | 0.337 | No | Yes | 2.77 | 0.664 | Yes |
| CHEMBL3707219 | COC(=O)c1ccc(OC(=O)CCCCCCC(=O)NO)cc1 | 323.345 | 2.2246 | 9 | 6 | 2 | 134.091 | -3.419 | 1.039 | 74.984 | -2.972 | Yes | No | No | -0.907 | 0.2 | -0.924 | -3.24 | No | No | No | No | No | No | No | 0.694 | No | Yes | 0.856 | No | No | 2.15 | 2.419 | Yes |
| CHEMBL4650327 | O=C(O)Cc1nn(Cc2nc3cc(C(F)(F)F)ccc3s2)c(=O)c2cscc12 | 425.413 | 3.7619 | 4 | 7 | 1 | 162.044 | -4.115 | 1.248 | 92.03 | -2.736 | No | No | Yes | -0.35 | 0.177 | -1.305 | -2.412 | No | Yes | No | No | No | No | No | 0.463 | No | No | 0.71 | No | No | 2.63 | 0.405 | Yes |
| CHEMBL3545065 | CN(C)C(C(=O)Nc1ccc2c(=O)[nH]ccc2c1)c1ccsc1 | 327.409 | 2.831 | 4 | 4 | 2 | 137.123 | -3.821 | 1.134 | 89.871 | -2.776 | Yes | No | No | 0.62 | 0.077 | 0.05 | -2.271 | No | Yes | Yes | No | No | No | No | 0.929 | No | No | 0.229 | No | Yes | 2.276 | 1.514 | Yes |
| CHEMBL2105684 | Cn1cnc2c(F)c(Nc3ccc(Br)cc3Cl)c(C(=O)NOCCO)cc21.O=S(=O)(O)O | 555.766 | 2.8728 | 6 | 8 | 5 | 194.366 | -2.913 | -0.81 | 15.091 | -2.735 | Yes | No | No | 0.147 | 0.343 | -2.294 | -3.695 | No | No | No | No | No | No | No | 0.629 | No | No | 0.451 | No | No | 2.348 | 3.136 | No |
| CHEMBL152 | Nc1ccn(C[C@@H](CO)OCP(=O)(O)O)c(=O)n1 | 279.189 | -1.6618 | 6 | 7 | 4 | 102.001 | -2.12 | 0.226 | 66.53 | -2.782 | No | No | No | -0.843 | 0.65 | -1.429 | -3.84 | No | No | No | No | No | No | No | 0.274 | No | No | 0.863 | No | No | 1.896 | 3.327 | Yes |
| CHEMBL3187723 | Cn1cnc2c(F)c(Nc3ccc(Br)cc3F)c(C(=O)NOCCO)cc21 | 441.232 | 3.0113 | 6 | 6 | 3 | 160.59 | -3.348 | 0.998 | 91.289 | -2.735 | Yes | Yes | No | 0.662 | 0.248 | -1.505 | -3.237 | No | No | Yes | No | Yes | No | Yes | 0.747 | No | No | 0.236 | No | Yes | 2.356 | 2.255 | No |
| CHEMBL2107817 | NC(=O)[C@@H]1CC[C@@H]2CN1C(=O)N2OS(=O)(=O)[O-].[Na+] | 287.229 | -4.8639 | 3 | 6 | 1 | 125.418 | -2.059 | 0.056 | 7.251 | -2.735 | No | No | No | -1.043 | 0.821 | -1.305 | -3.427 | No | No | No | No | No | No | No | 0.909 | No | No | 1.298 | No | No | 1.956 | 0.875 | Yes |
| CHEMBL110 | O=C(Cn1ccnc1[N+](=O)[O-])NCc1ccccc1 | 260.253 | 1.1077 | 5 | 5 | 1 | 108.586 | -2.445 | 0.729 | 76.335 | -2.744 | Yes | No | No | 0.017 | 0.266 | -0.977 | -2.696 | No | No | No | No | No | No | No | 0.456 | Yes | Yes | 0.687 | No | No | 1.839 | 1.564 | Yes |
| CHEMBL644 | CC(CN(C)C)CN1c2ccccc2CCc2ccccc21 | 294.442 | 4.121 | 4 | 2 | 0 | 133.922 | -4.265 | 1.468 | 96.095 | -2.599 | No | Yes | No | 1.836 | 0.091 | 0.92 | -1.35 | Yes | Yes | No | No | No | Yes | Yes | 0.712 | Yes | No | 0.318 | No | Yes | 2.909 | 0.69 | Yes |
| CHEMBL4303323 | C[C@H](Nc1ncnc2[nH]cnc12)c1nc2ccc(F)cc2c(=O)n1-c1ccccc1 | 401.405 | 3.3642 | 4 | 7 | 2 | 169.033 | -2.897 | 1.167 | 89.282 | -2.735 | No | Yes | Yes | -0.093 | 0.35 | -1.697 | -3.866 | No | No | No | No | Yes | No | Yes | 0.497 | No | Yes | 0.413 | No | Yes | 2.538 | 2.489 | Yes |
| CHEMBL2079130 | CNCc1cc(-c2ccccc2F)n(S(=O)(=O)c2cccnc2)c1 | 345.399 | 2.6456 | 5 | 5 | 1 | 139.39 | -3.055 | 1.269 | 97.288 | -2.697 | No | Yes | No | 0.677 | 0.198 | -0.23 | -2.604 | No | Yes | Yes | No | No | No | Yes | 0.286 | No | Yes | -0.164 | No | Yes | 2.645 | 0.847 | Yes |
| CHEMBL126224 | CN(C)CCCn1c2c(c3ccccc31)CCCCCC2 | 284.447 | 4.252 | 4 | 2 | 0 | 128.301 | -4.053 | 1.461 | 93.251 | -2.109 | Yes | Yes | No | 1.695 | 0.183 | 0.678 | -2.131 | Yes | Yes | Yes | No | No | Yes | No | 1.09 | Yes | No | -0.153 | No | Yes | 2.681 | 0.778 | Yes |
| CHEMBL1289926 | CNC(=O)c1ccccc1Sc1ccc2c(/C=C/c3ccccn3)n[nH]c2c1 | 386.48 | 4.6391 | 5 | 4 | 2 | 166.449 | -4.297 | 0.788 | 92.892 | -2.751 | Yes | Yes | Yes | -0.02 | 0.025 | -0.89 | -1.983 | No | Yes | Yes | Yes | Yes | Yes | Yes | 0.123 | No | No | 0.335 | No | Yes | 2.827 | 1.66 | Yes |
| CHEMBL85164 | CN(C)C(=O)COC(=O)Cc1ccc(OC(=O)c2ccc(NC(=N)N)cc2)cc1.CS(=O)(=O)O | 494.526 | 0.88907 | 7 | 8 | 4 | 196.454 | -3.015 | -0.595 | 18.554 | -2.735 | Yes | No | No | 0.133 | 0.149 | -1.891 | -4.018 | No | Yes | No | No | No | No | No | 0.567 | No | No | -0.037 | No | No | 2.46 | 3.264 | Yes |
| CHEMBL49642 | O=C(Nc1ccncc1)C(=O)c1cn(Cc2ccc(Cl)cc2)c2ccccc12 | 389.842 | 4.5594 | 5 | 4 | 1 | 166.137 | -4.742 | 0.594 | 94.514 | -2.69 | Yes | Yes | Yes | 0.214 | 0.18 | -0.099 | -1.911 | No | Yes | Yes | Yes | Yes | No | Yes | -0.056 | No | No | 0.17 | No | Yes | 2.749 | 0.841 | Yes |
| CHEMBL225071 | Cc1nc(=O)c2cc(CN(C)c3ccc(C(=O)N[C@@H](CCC(=O)O)C(=O)O)s3)ccc2[nH]1 | 458.496 | 1.97722 | 9 | 7 | 4 | 186.079 | -2.929 | -0.552 | 28.919 | -2.735 | Yes | No | No | -0.531 | 0.144 | -1.461 | -3.813 | No | Yes | No | No | No | No | No | 0.016 | No | No | -0.442 | No | No | 2.44 | 2.463 | Yes |
| CHEMBL799 | O=C1CCc2cc(OCCCCc3nnnn3C3CCCCC3)ccc2N1 | 369.469 | 3.4647 | 7 | 6 | 1 | 159.029 | -3.917 | 1.314 | 94.269 | -2.979 | Yes | Yes | Yes | 0.487 | 0.109 | -0.728 | -3.024 | No | Yes | No | No | No | No | Yes | 0.962 | No | No | -0.125 | No | No | 2.472 | 1.43 | Yes |
| CHEMBL518520 | Cc1nnc(C(=O)NC(C)(C)c2nc(C(=O)NCc3ccc(F)cc3)c([O-])c(=O)n2C)o1.[K+] | 482.513 | -2.71648 | 6 | 9 | 2 | 229.783 | -3.232 | 0.891 | 48.565 | -2.739 | No | No | No | -1.11 | 0.068 | -1.581 | -3.397 | No | Yes | No | No | No | No | No | 1.454 | No | No | 0.713 | No | Yes | 1.727 | 1.809 | Yes |
| CHEMBL2105733 | CNCc1ccc(-c2[nH]c3cc(F)cc4c3c2CCNC4=O)cc1.O=P(O)(O)O | 421.365 | 2.0507 | 3 | 3 | 6 | 165.563 | -3.313 | -0.118 | 62.463 | -2.743 | Yes | No | No | -0.068 | 0.141 | -1.829 | -3.468 | No | Yes | No | No | No | No | No | 0.797 | No | No | 0.549 | No | Yes | 2.476 | 2.863 | Yes |
| CHEMBL1173055 | CNCc1ccc(-c2[nH]c3cc(F)cc4c3c2CCNC4=O)cc1 | 323.371 | 2.9793 | 3 | 2 | 3 | 138.625 | -3.517 | 1.212 | 92.282 | -2.768 | Yes | No | Yes | 0.894 | 0.124 | 0.278 | -2.067 | Yes | Yes | Yes | No | No | Yes | Yes | 1.008 | No | No | 0.504 | No | Yes | 2.492 | 0.904 | Yes |
| CHEMBL25263 | O=C(O)[C@H]1CCCN1C(=O)CCCCC(=O)N1CCC[C@@H]1C(=O)O | 340.376 | 0.698 | 7 | 4 | 2 | 139.956 | -1.966 | -0.132 | 23.236 | -2.735 | Yes | No | No | -1.33 | 0.563 | -1.151 | -3.242 | No | No | No | No | No | No | No | 1.441 | No | No | 0.502 | No | No | 1.617 | 0.644 | Yes |
| CHEMBL3137312 | COc1c(-c2ccc3cc(NS(C)(=O)=O)ccc3c2)cc(-n2ccc(=O)[nH]c2=O)cc1C(C)(C)C | 493.585 | 4.0236 | 5 | 6 | 2 | 202.813 | -3.813 | -0.396 | 83.938 | -2.735 | Yes | Yes | Yes | -1.535 | 0.167 | -1.194 | -2.32 | No | Yes | No | Yes | Yes | No | Yes | 0.766 | No | Yes | 0.272 | No | Yes | 2.974 | 2.139 | Yes |
| CHEMBL1339 | CCCc1nc(C)c2c(O)nc(-c3cc(S(=O)(=O)N4CCN(CC)CC4)ccc3OCC)nn12.Cl | 525.075 | 2.90452 | 8 | 9 | 1 | 211.949 | -3.208 | 1.347 | 81.321 | -2.735 | Yes | Yes | No | 0.252 | 0.35 | -1.154 | -3.228 | No | Yes | No | No | Yes | No | Yes | 0.63 | Yes | Yes | 0.488 | No | No | 2.207 | 1.812 | Yes |
| CHEMBL2107358 | COC1=CC(c2cc3ccccc3[nH]2)=N/C1=C\c1[nH]c(C)cc1C.CS(=O)(=O)O | 413.499 | 3.99094 | 3 | 4 | 3 | 169.131 | -3.994 | 0.326 | 66.93 | -2.68 | Yes | No | Yes | -0.468 | 0.207 | -1.145 | -2.135 | No | Yes | No | No | No | No | Yes | 0.587 | Yes | No | -0.582 | No | No | 2.437 | 0.942 | Yes |
| CHEMBL363387 | O=C(O)Cn1cc(Cc2nc3c(F)c(F)cc(F)c3s2)c2ccccc21 | 376.359 | 4.3437 | 4 | 4 | 1 | 148.678 | -3.966 | 1.145 | 94.602 | -2.735 | No | No | Yes | -0.424 | 0.254 | 0.606 | -3.15 | No | Yes | Yes | No | Yes | No | No | 0.284 | No | No | 0.779 | No | No | 2.741 | 0.708 | Yes |
| CHEMBL4650338 | CC#CC(=O)N1CC[C@@H](n2cc(-c3ccc(Oc4c(F)cccc4F)cc3)c3c(N)n[nH]c(=O)c32)C1 | 489.482 | 3.841 | 4 | 6 | 2 | 203.834 | -3.407 | 0.028 | 83.364 | -2.735 | Yes | Yes | Yes | -0.602 | 0.087 | -1.673 | -3.676 | No | Yes | No | Yes | Yes | No | Yes | -0.009 | No | No | 0.484 | No | Yes | 2.728 | 2.366 | Yes |
| CHEMBL521686 | O=C(c1cc(Cc2n[nH]c(=O)c3ccccc23)ccc1F)N1CCN(C(=O)C2CC2)CC1 | 434.471 | 2.3474 | 4 | 4 | 1 | 183.385 | -3.605 | 1.052 | 93.805 | -2.735 | Yes | No | Yes | 0.208 | 0.035 | -0.974 | -2.589 | Yes | Yes | Yes | Yes | Yes | No | No | 0.494 | No | No | 0.482 | No | Yes | 2.311 | 2.882 | Yes |
| CHEMBL1168 | CCOC(=O)[C@H](CCc1ccccc1)N[C@@H](C)C(=O)N1[C@H](C(=O)O)C[C@@H]2CCC[C@@H]21 | 416.518 | 2.3832 | 9 | 5 | 2 | 177.326 | -2.714 | 0.167 | 50.278 | -2.735 | Yes | No | No | -0.236 | 0.671 | -0.696 | -3.012 | No | Yes | No | No | No | No | No | 0.759 | No | No | 0.05 | No | No | 2.087 | 2.047 | Yes |
| CHEMBL713 | C=C1[C@@H](n2cnc3c(=O)[nH]c(N)nc32)C[C@H](O)[C@H]1CO | 277.284 | -0.8278 | 2 | 7 | 4 | 113.14 | -2.982 | 0.063 | 65.435 | -2.735 | Yes | No | No | 0.253 | 0.758 | -1.225 | -4.018 | No | No | No | No | No | No | No | 0.745 | No | Yes | 0.314 | No | No | 2.526 | 2.109 | Yes |
| CHEMBL2110588 | CCCCCCCC(=O)N[C@H](CN1CCCC1)[C@H](O)c1ccc2c(c1)OCCO2 | 404.551 | 3.4323 | 11 | 5 | 2 | 174.116 | -3.729 | 0.811 | 92.757 | -2.789 | Yes | Yes | No | 0.629 | 0.24 | -0.887 | -2.958 | No | Yes | No | No | No | Yes | Yes | 1.315 | No | No | -1.078 | No | Yes | 2.832 | 0.655 | Yes |
| CHEMBL777 | O=C(O)[C@H]1/C(=C/CO)O[C@@H]2CC(=O)N21 | 199.162 | -1.0956 | 2 | 4 | 2 | 79.377 | -1.359 | 0.557 | 70.393 | -3.132 | No | No | No | -0.633 | 0.764 | -0.705 | -3.238 | No | No | No | No | No | No | No | 0.529 | No | No | 0.765 | No | No | 1.935 | 2.156 | Yes |
| CHEMBL1083390 | O=S(=O)(c1ccccc1)c1cnc2c(N3CCNCC3)cccc2c1 | 353.447 | 2.4772 | 3 | 5 | 1 | 146.779 | -3.608 | 1.224 | 97.631 | -2.754 | Yes | No | Yes | 0.664 | 0.167 | 0.23 | -2.274 | No | Yes | Yes | No | No | No | No | 0.589 | No | No | 0.021 | No | Yes | 2.776 | 1.327 | Yes |
| CHEMBL4594446 | O=C(c1cc(Cl)c(O)c(Cl)c1)N1CS(=O)(=O)c2ccccc21 | 358.202 | 3.0906 | 1 | 4 | 1 | 136.16 | -4.309 | 1.207 | 94.49 | -2.942 | No | No | No | -0.146 | 0.077 | -0.745 | -2.067 | No | Yes | Yes | Yes | Yes | No | No | 0.16 | No | Yes | 0.438 | No | No | 2.569 | 1.202 | No |
| CHEMBL3099695 | N#Cc1ccc([C@H]2CCc3cncn32)c(F)c1 | 227.242 | 2.42948 | 1 | 3 | 0 | 98.139 | -2.344 | 1.72 | 97.678 | -2.988 | Yes | No | No | 0.234 | 0.312 | 0.209 | -1.809 | No | No | Yes | No | No | Yes | No | 0.896 | No | Yes | 0.298 | No | No | 2.445 | 1.664 | No |
| CHEMBL3545185 | O=C(/C=C\n1cnc(-c2cc(C(F)(F)F)cc(C(F)(F)F)c2)n1)NNc1cnccn1 | 443.311 | 3.3867 | 5 | 7 | 2 | 169.171 | -5.509 | 0.116 | 87.946 | -2.839 | Yes | Yes | No | -1.266 | 0.21 | -1.808 | -2.987 | No | Yes | Yes | No | Yes | No | Yes | 0.36 | No | Yes | 0.455 | No | Yes | 3.045 | 0.723 | Yes |
| CHEMBL3894860 | CN1CCN(c2ccc(Nc3ncc4cc5n(c4n3)C3(CCCCC3)CNC5=O)nc2)CC1 | 446.559 | 2.7245 | 3 | 8 | 2 | 192.756 | -3.756 | 1.194 | 94.452 | -2.843 | Yes | No | No | 0.895 | 0.346 | -1.109 | -3.065 | No | Yes | No | No | No | No | No | 0.76 | No | No | -0.591 | No | Yes | 2.839 | 1.2 | Yes |
| CHEMBL2048484 | Cc1ccc([C@@H]2O[C@H](CO)[C@@H](O)[C@H](O)[C@H]2O)cc1Cc1ccc(-c2ccc(F)cc2)s1 | 444.524 | 2.96832 | 5 | 6 | 4 | 183.556 | -4.447 | 0.711 | 98.263 | -2.735 | Yes | Yes | Yes | -1.18 | 0.029 | -1.186 | -3.303 | No | Yes | No | Yes | Yes | No | No | 0.039 | No | No | 0.28 | No | Yes | 3.125 | 4.301 | No |
| CHEMBL1161681 | O=NO | 47.013 | 0.142 | 0 | 2 | 1 | 16.817 | 0.794 | 1.164 | 95.091 | -3.4 | Yes | No | No | -0.322 | 0.8 | -0.445 | -2.976 | No | No | No | No | No | No | No | 0.555 | No | No | 1.432 | No | No | 2.705 | 1.61 | No |
| CHEMBL1789941 | N#CC[C@H](C1CCCC1)n1cc(-c2ncnc3[nH]ccc23)cn1 | 306.373 | 3.46638 | 4 | 5 | 1 | 133.732 | -3.462 | 1.303 | 94.482 | -2.861 | Yes | No | No | 0.727 | 0.178 | -0.143 | -2.382 | No | Yes | Yes | Yes | No | No | No | 0.965 | No | No | -0.196 | No | No | 2.56 | 0.221 | Yes |
| CHEMBL3545110 | CN(C)C(=O)c1cc2cnc(Nc3ccc(N4CCNCC4)cn3)nc2n1C1CCCC1 | 434.548 | 2.7964 | 5 | 8 | 2 | 187.397 | -3.625 | 1.174 | 94.012 | -2.83 | Yes | Yes | No | 0.687 | 0.299 | -1.204 | -3.198 | No | Yes | No | No | No | No | No | 0.685 | No | No | -0.557 | No | Yes | 2.596 | -0.527 | Yes |
| CHEMBL1206245 | COc1cc(OC)c(/C=C/S(=O)(=O)Cc2ccc(OC)c(OP(=O)(O)O)c2)c(OC)c1 | 474.424 | 2.7782 | 10 | 8 | 2 | 179.685 | -3.827 | 0.557 | 61.26 | -2.735 | Yes | Yes | No | -0.736 | 0.081 | -1.174 | -3.741 | No | No | No | No | No | No | Yes | 0.608 | No | No | 0.55 | No | No | 2.754 | 3.298 | No |
| CHEMBL1089318 | Cc1c(Cl)c(C)[n+]([O-])c(Cl)c1-c1noc(-c2cc(O)c(O)c([N+](=O)[O-])c2)n1 | 413.173 | 3.28004 | 3 | 8 | 2 | 160.486 | -3.456 | -0.069 | 85.314 | -2.739 | Yes | Yes | No | 0.475 | 0.196 | -1.327 | -2.299 | No | No | Yes | No | No | No | Yes | -0.036 | No | No | 0.448 | No | Yes | 2.787 | 2.059 | Yes |
| CHEMBL166863 | CC1(C)OC(c2ccc(S(N)(=O)=O)cc2)=C(c2cccc(F)c2)C1=O | 361.394 | 2.7193 | 3 | 4 | 1 | 144.524 | -4.606 | 1.195 | 95.444 | -2.975 | No | Yes | No | -0.269 | 0.034 | -0.652 | -2.327 | No | Yes | No | Yes | No | No | No | 0.431 | No | Yes | -0.081 | No | Yes | 2.361 | 1.179 | Yes |
| CHEMBL1771 | COC(=O)[C@H](c1ccccc1Cl)N1CCc2sccc2C1 | 321.829 | 3.6739 | 3 | 4 | 0 | 132.651 | -3.157 | 1.882 | 89.628 | -2.571 | No | No | No | 0.794 | 0.277 | 0.427 | -1.694 | No | Yes | Yes | Yes | No | Yes | No | 0.948 | Yes | No | 0.359 | No | No | 3.434 | 1.514 | No |
| CHEMBL3661404 | COC(c1ccccc1)C1OC1c1ccccc1 | 240.302 | 3.5142 | 4 | 2 | 0 | 107.17 | -3.883 | 1.759 | 96.319 | -2.653 | No | No | No | 0.334 | 0.024 | 0.402 | -1.556 | No | Yes | Yes | Yes | No | No | No | 0.258 | No | Yes | 0.696 | No | No | 1.632 | 2.08 | No |
| CHEMBL115794 | C[C@@H]1N[C@H](C)CO[C@@]1(O)c1cc(F)cc(F)c1 | 243.253 | 1.5067 | 1 | 3 | 2 | 98.404 | -1.677 | 1.112 | 94.218 | -3.127 | Yes | No | No | 0.226 | 0.406 | 0.006 | -1.92 | No | No | No | No | No | No | No | 0.819 | No | No | 0.676 | No | No | 2.52 | 1.648 | No |
| CHEMBL1908332 | CC(C)NC(=O)N(C(=O)[C@H]1CC[C@H]2[C@@H]3CC[C@H]4N(C)C(=O)CC[C@]4(C)[C@H]3CC[C@]12C)C(C)C | 459.675 | 4.821 | 3 | 3 | 1 | 199.759 | -4.449 | 1.133 | 93.714 | -2.791 | Yes | Yes | Yes | -0.507 | 0 | -0.421 | -1.601 | No | Yes | No | No | No | No | No | 0.419 | No | No | -0.666 | No | No | 4.532 | 0.76 | No |
| CHEMBL1029 | CCCCN1C[C@H](O)[C@@H](O)[C@H](O)[C@H]1CO | 219.281 | -1.4543 | 4 | 5 | 4 | 89.955 | -0.767 | 0.382 | 53.669 | -3.332 | No | No | No | -0.374 | 0.858 | -1.388 | -4.675 | No | No | No | No | No | No | No | 0.88 | No | No | 2.005 | No | No | 1.895 | 3.755 | No |
| CHEMBL272980 | Nc1ccccc1NC(=O)c1ccc(CNc2nccc(-c3cccnc3)n2)cc1 | 396.454 | 3.9852 | 6 | 6 | 3 | 173.593 | -3.478 | 1.17 | 89.026 | -2.739 | Yes | Yes | Yes | 0.341 | 0.076 | -1.165 | -2.362 | No | Yes | Yes | Yes | No | No | Yes | 0.288 | No | Yes | 0.235 | No | Yes | 2.799 | 0.69 | Yes |
| CHEMBL1694 | CCOC(=O)[C@H](CCc1ccccc1)N[C@H]1CCc2ccccc2N(CC(=O)O)C1=O.Cl | 460.958 | 2.9949 | 9 | 5 | 2 | 193.717 | -3.369 | 0.226 | 55.591 | -2.733 | Yes | No | No | -0.023 | 0.394 | -0.596 | -2.813 | No | No | No | No | No | No | No | 0.955 | No | No | -0.105 | No | No | 2.774 | 2.069 | Yes |
| CHEMBL953 | CCN(CC)C(=O)/C(C#N)=C/c1cc(O)c(O)c([N+](=O)[O-])c1 | 305.29 | 1.78138 | 5 | 6 | 2 | 126.217 | -2.283 | 0.03 | 75.611 | -2.75 | Yes | No | No | -0.262 | 0.181 | -1.021 | -2.764 | No | No | No | No | No | No | No | 0.911 | No | No | 0.19 | No | No | 2.73 | 1.821 | No |
| CHEMBL3989967 | CNc1nc(-c2cccc(NC(=O)c3ccc(C(=O)OC)cc3)c2)c2cc(OC)c(OC)cc2n1 | 472.501 | 4.3946 | 7 | 8 | 2 | 201.62 | -4.033 | 1.49 | 89.868 | -2.735 | Yes | Yes | Yes | -0.569 | 0.159 | -0.625 | -3.142 | No | Yes | No | Yes | Yes | No | Yes | 0.539 | No | No | 0.348 | No | Yes | 2.242 | 1.65 | Yes |
| CHEMBL76222 | CNC(=O)[C@@H](NC(=O)[C@H](CC(C)C)NC(=O)[C@@H](S)CCN1C(=O)N(C)C(C)(C)C1=O)C(C)(C)C | 499.678 | 1.1553 | 10 | 6 | 4 | 207.894 | -3.1 | -0.161 | 47.158 | -2.714 | Yes | No | No | -0.819 | 0.325 | -1.237 | -3.514 | No | No | No | No | No | No | No | 0.162 | No | No | -0.053 | No | No | 3.456 | 2.266 | No |
| CHEMBL4297615 | CCOc1c([C@H](C)n2nc(C)c3c(N)ncnc32)cc(Cl)c(F)c1[C@@H]1CNC(=O)C1 | 432.887 | 3.12092 | 5 | 7 | 2 | 177.129 | -3.828 | 0.807 | 89.545 | -2.742 | Yes | Yes | No | 0.157 | 0.175 | -1.168 | -3.109 | No | Yes | No | No | Yes | No | Yes | 0.336 | No | No | 0.175 | No | Yes | 2.36 | 1.283 | Yes |
| CHEMBL1503 | COc1ccc2[nH]c([S+]([O-])Cc3ncc(C)c(OC)c3C)nc2c1 | 345.424 | 2.89974 | 5 | 5 | 1 | 142.965 | -3.046 | 1.358 | 85.646 | -2.735 | Yes | No | No | -0.022 | 0.184 | -0.337 | -2.928 | No | No | Yes | Yes | No | No | Yes | 0.872 | Yes | Yes | -0.147 | No | No | 2.487 | 0.68 | Yes |
| CHEMBL75094 | CC1(C)SCCN(S(=O)(=O)c2ccc(Oc3ccncc3)cc2)[C@H]1C(=O)NO | 423.516 | 2.264 | 5 | 7 | 2 | 167.474 | -3.771 | 1.235 | 73.963 | -2.772 | Yes | No | No | -0.721 | 0.117 | -1.329 | -3.537 | No | No | No | No | No | No | No | -0.076 | No | No | 0.42 | No | No | 3.309 | 2.349 | Yes |
| CHEMBL3187812 | CC(=O)S[C@H](C(=O)N[C@H]1Cc2ccccc2[C@H]2CCC[C@@H](C(=O)O)N2C1=O)C(C)C | 432.542 | 2.5386 | 5 | 5 | 2 | 180.132 | -3.502 | 0.718 | 55.05 | -2.733 | Yes | No | No | -1.031 | 0.274 | -0.87 | -2.596 | No | No | No | No | No | No | No | 0.065 | No | No | 0.219 | No | No | 2.009 | 1.892 | Yes |
| CHEMBL4130229 | Cc1ccc(C(=O)NNC(=O)c2c(C)cccc2Cl)cc1C#Cc1cnc2ccccc2c1 | 453.929 | 4.97964 | 2 | 3 | 2 | 196.332 | -5.556 | 1.131 | 93.059 | -2.737 | Yes | Yes | Yes | -0.648 | 0.034 | 0.288 | -1.461 | No | Yes | Yes | Yes | Yes | No | Yes | -0.353 | No | Yes | 0.381 | No | Yes | 2.723 | 1.618 | Yes |
| CHEMBL273264 | N=C(N)Nc1ccc(C(=O)Oc2ccc3cc(C(=N)N)ccc3c2)cc1 | 347.378 | 2.64844 | 4 | 4 | 5 | 149.14 | -3.555 | -0.167 | 72.997 | -2.735 | Yes | Yes | No | 1.283 | 0.042 | -1.135 | -2.557 | No | Yes | Yes | No | No | No | No | 0.541 | No | Yes | 0.378 | No | Yes | 2.302 | 2.608 | No |
| CHEMBL1467 | O=c1ncnc2[nH][nH]cc1-2 | 136.114 | -0.4022 | 0 | 3 | 2 | 55.466 | -1.355 | 0.222 | 75.453 | -3.393 | No | No | No | -0.451 | 0.79 | -0.776 | -3.874 | No | No | No | No | No | No | No | 0.567 | No | Yes | 1.568 | No | No | 1.822 | 1.606 | No |
| CHEMBL1115 | CN(C)C(=O)Oc1ccc[n+](C)c1 | 181.215 | 0.5715 | 1 | 2 | 0 | 77.356 | -0.643 | 1.673 | 97.882 | -2.778 | No | No | No | -0.043 | 0.775 | -0.119 | -3.085 | No | No | No | No | No | No | No | 0.444 | No | No | 0.623 | No | No | 2.774 | 1.895 | Yes |
| CHEMBL4650443 | Cn1cc2cc(C(F)(F)c3nnc4ccc(-c5cnn(C6CC6)c5)nn34)c(F)cc2n1 | 424.39 | 3.4886 | 4 | 8 | 0 | 172.471 | -2.884 | 1.177 | 100 | -2.735 | No | Yes | Yes | 0.402 | 0.262 | -1.63 | -3.056 | No | Yes | Yes | No | No | No | Yes | 0.419 | Yes | No | 0.171 | No | Yes | 2.137 | 0.692 | Yes |
| CHEMBL2111084 | CC[C@@]1(O)C(=O)OCc2c1cc1n(c2=O)Cc2c-1nc1ccccc1c2CCNC(C)C | 433.508 | 2.6201 | 5 | 7 | 2 | 185.603 | -3.819 | 1.304 | 87.551 | -2.735 | Yes | Yes | Yes | 0.337 | 0.174 | -0.331 | -2.697 | No | Yes | Yes | No | No | No | No | 1.321 | No | No | 0.273 | No | Yes | 2.566 | 1.974 | Yes |
| CHEMBL271068 | Cc1cc(NC(=O)C(=O)c2cc(Cc3ccc(C#N)cc3)n3ccccc23)sn1 | 400.463 | 3.9881 | 5 | 6 | 1 | 170.755 | -3.728 | 1.135 | 98.049 | -2.747 | No | Yes | Yes | 0.308 | 0.153 | -0.715 | -2.856 | No | Yes | Yes | Yes | Yes | No | Yes | -0.087 | No | No | -0.513 | No | Yes | 2.697 | 1.206 | Yes |
| CHEMBL4459585 | Cn1cc(-c2cn3nccc3c(-c3cnn([C@]4(CC#N)C[C@@H](C#N)C4)c3)n2)cn1 | 383.419 | 2.53586 | 4 | 9 | 0 | 165.985 | -3.17 | 1.11 | 99.748 | -2.746 | No | Yes | No | 0.275 | 0.316 | -1.268 | -2.927 | No | Yes | Yes | No | No | No | Yes | 0.81 | No | No | -0.817 | No | Yes | 2.296 | 0.979 | Yes |
| CHEMBL4296717 | CN(C)Cc1ccc(S(=O)(=O)n2ccc(/C=C/C(=O)NO)c2)cc1 | 349.412 | 1.3053 | 6 | 6 | 2 | 140.388 | -3.29 | 0.048 | 72.174 | -2.991 | Yes | No | No | 0.071 | 0.21 | -1.001 | -3.082 | No | Yes | No | No | No | No | No | 0.376 | No | No | 0.611 | No | Yes | 2.722 | 2.806 | Yes |
| CHEMBL3989908 | CC(C)(O)CNc1nc(Nc2ccnc(C(F)(F)F)c2)nc(-c2cccc(C(F)(F)F)n2)n1 | 473.381 | 4.2926 | 6 | 8 | 3 | 182.721 | -4.364 | 0.422 | 82.864 | -2.805 | No | No | No | -0.743 | 0.23 | -2.234 | -3.732 | No | Yes | No | No | No | No | No | -0.826 | No | No | 0.005 | No | Yes | 2.354 | 0.869 | Yes |
| CHEMBL4650343 | Cc1cc(Nc2cc3ncccc3c(N[C@H]3C[C@@H]4CC[C@H](C3)N4CCC#N)n2)n[nH]1 | 402.506 | 3.7259 | 6 | 7 | 3 | 175.262 | -2.984 | 0.296 | 84.346 | -2.735 | Yes | No | No | 1.815 | 0.478 | -1.755 | -3.822 | Yes | Yes | No | No | No | No | No | 0.953 | No | No | 0.318 | No | Yes | 2.612 | 0.354 | Yes |
| CHEMBL2110666 | N#C[C@@H]1C[C@H](F)CN1C(=O)[C@@H](N)C(c1ccc(F)cc1)c1ccc(F)cc1 | 373.378 | 2.88658 | 4 | 3 | 1 | 154.553 | -4.276 | 0.875 | 95.145 | -2.741 | Yes | Yes | No | 0.707 | 0.14 | -0.055 | -2.374 | No | No | No | No | No | No | Yes | 0.501 | No | Yes | -0.343 | No | Yes | 2.234 | 1.466 | Yes |
| CHEMBL3916243 | C[C@@]1(c2cc(NC(=O)c3ccc(C#N)cn3)ccc2F)C=CSC(N)=N1 | 367.409 | 3.13508 | 3 | 6 | 2 | 153.195 | -4.636 | 0.939 | 89.119 | -3.306 | Yes | No | No | 0.015 | 0.17 | -0.669 | -2.942 | No | Yes | No | Yes | Yes | No | No | -0.431 | No | Yes | 0.084 | No | No | 2.446 | 0.939 | No |
| CHEMBL2105987 | CC1=CC(=O)C=C2CC[C@H]3[C@@H]4CCC(=O)[C@@]4(C)CC[C@@H]3[C@@]12C | 298.426 | 4.2535 | 0 | 2 | 0 | 132.594 | -4.505 | 1.266 | 100 | -2.583 | No | Yes | No | 0.241 | 0.057 | 0.102 | -2.153 | No | Yes | No | Yes | No | No | Yes | 0.98 | Yes | No | -0.153 | No | Yes | 1.81 | 1.78 | No |
| CHEMBL3707235 | N[C@@H](CC(=O)N1CCc2c(nc(C(F)(F)F)nc2C(F)(F)F)C1)CN1CC(F)(F)CCC1=O | 489.367 | 2.374 | 4 | 5 | 1 | 181.475 | -5.475 | 0.742 | 76.66 | -3.055 | No | No | No | -0.422 | 0.439 | -1.446 | -3.291 | No | Yes | No | No | No | No | No | 0.279 | No | No | -0.092 | No | No | 3.263 | 0.806 | Yes |
| CHEMBL488 | CCC1(c2ccc(N)cc2)CCC(=O)NC1=O | 232.283 | 1.3532 | 2 | 3 | 2 | 100.193 | -2.544 | 0.749 | 92.846 | -3.506 | Yes | No | No | 0.168 | 0.45 | 0.027 | -2.393 | No | No | No | No | No | No | No | 0.138 | No | No | 0.351 | No | No | 2.424 | 1.489 | No |
| CHEMBL552212 | CCC[C@H](Nc1nc(-c2ccc(NC(=O)NCC)c(OC)c2)ncc1C)c1cccnc1 | 434.544 | 4.95032 | 9 | 6 | 3 | 188.414 | -4.554 | 0.925 | 85.15 | -2.792 | Yes | Yes | Yes | 0.331 | 0.049 | -1.627 | -2.972 | No | Yes | No | Yes | Yes | No | Yes | 0.561 | No | No | 0.33 | No | Yes | 2.758 | 1.465 | Yes |
| CHEMBL4650272 | CN1CCN(c2ccc(Nc3ncc4cc5n(c4n3)C3(CCCCC3)CNC5=O)nc2)CC1.Cl.Cl | 519.481 | 3.5681 | 3 | 8 | 2 | 217.062 | -4.005 | 1.18 | 92.376 | -2.807 | Yes | Yes | No | 0.827 | 0.29 | -1.38 | -3.086 | No | Yes | No | No | No | No | No | 1.02 | No | No | -0.558 | No | Yes | 2.762 | 1.087 | Yes |
| CHEMBL3039517 | Cc1cnc(NC(=O)Nc2cc(Br)c(C)cc2OC[C@@H]2CNCCO2)cn1 | 436.31 | 2.86714 | 5 | 6 | 3 | 165.747 | -3.831 | 0.777 | 78.468 | -3.076 | Yes | No | No | 0.427 | 0.368 | -1.378 | -3.032 | No | Yes | No | No | No | No | No | 0.756 | No | No | -0.04 | No | Yes | 2.324 | 1.29 | Yes |
| CHEMBL2177390 | CC(C)NC[C@@H](C(=O)N1CCN(c2ncnc3c2[C@H](C)C[C@H]3O)CC1)c1ccc(Cl)cc1 | 458.006 | 3.101 | 6 | 6 | 2 | 194.354 | -3.737 | 1.438 | 91.826 | -3.113 | Yes | Yes | Yes | 0.848 | 0.166 | -0.708 | -2.569 | No | Yes | No | No | No | No | Yes | 0.74 | Yes | No | -0.135 | No | Yes | 2.302 | 0.434 | Yes |
| CHEMBL2107357 | CC1(C)CNc2cc(NC(=O)c3cccnc3NCc3ccncc3)ccc21.O=P(O)(O)O.O=P(O)(O)O | 569.448 | 2.1869 | 5 | 7 | 9 | 217.856 | -3.011 | -0.788 | 31.25 | -2.735 | Yes | No | No | -1.054 | 0.255 | -2.957 | -5.742 | No | No | No | No | No | No | No | -0.801 | No | No | 0.564 | No | Yes | 3.038 | 3.76 | Yes |
| CHEMBL4297421 | O=C(Nc1nc(-c2ccc(O)c(O)c2)c(-c2ccccc2)s1)c1ccc(O)c(O)c1 | 420.446 | 4.5518 | 4 | 7 | 5 | 174.716 | -3.07 | -0.087 | 93.604 | -2.735 | Yes | Yes | Yes | -1.013 | 0.201 | -1.806 | -3.181 | No | No | No | Yes | Yes | No | No | -0.021 | No | No | 0.505 | No | Yes | 2.445 | 3.084 | Yes |
| CHEMBL1835207 | CCOc1ccc(-c2cc(C)cn2-c2ccc(S(N)(=O)=O)cc2)cc1 | 356.447 | 3.49882 | 5 | 4 | 1 | 147.274 | -4.413 | 1.048 | 96.211 | -2.716 | No | No | Yes | 0.013 | 0.166 | -0.637 | -2.231 | No | Yes | Yes | Yes | Yes | No | Yes | 0.924 | No | No | 0.53 | No | Yes | 2.296 | 0.776 | Yes |
| CHEMBL4571518 | O=c1[nH]c(NCc2ccc(Cl)c(Cl)c2)nc2ncn(CCN3CCOCC3)c12 | 423.304 | 2.3707 | 6 | 7 | 2 | 170.988 | -2.719 | 0.933 | 94.144 | -2.735 | Yes | No | No | 0.217 | 0.378 | -1.525 | -3.474 | No | Yes | No | No | No | No | Yes | 1.105 | Yes | No | 0.744 | No | Yes | 2.55 | 0.898 | Yes |
| CHEMBL483254 | Cc1[nH]c2ccccc2c1CCNCc1ccc(/C=C/C(=O)NO)cc1 | 349.434 | 3.32722 | 7 | 3 | 4 | 152.254 | -3.249 | 0.796 | 83.603 | -2.739 | Yes | Yes | Yes | 0.923 | 0.208 | -0.726 | -2.35 | Yes | Yes | Yes | Yes | No | Yes | Yes | 1.25 | No | No | 0.721 | No | Yes | 2.253 | 3.273 | Yes |
| CHEMBL4204869 | C[C@H]1OC[C@]2(c3cc(NC(=O)c4cnc(C(F)F)cn4)ccc3F)N=C(N)SC[C@H]12 | 437.447 | 3.0973 | 4 | 7 | 2 | 173.88 | -3.815 | 1.048 | 85.668 | -2.937 | Yes | No | No | -0.015 | 0.077 | -1.176 | -3.114 | No | Yes | No | No | No | No | No | 0.231 | No | No | 0.072 | No | Yes | 2.932 | 1.463 | Yes |
| CHEMBL3590106 | CC(C)Nc1cc(-c2c[nH]c(C(=O)N[C@H](CO)c3cccc(Cl)c3)c2)c(Cl)cn1 | 433.339 | 4.6672 | 7 | 4 | 4 | 178.826 | -4.705 | 0.951 | 90.303 | -2.816 | Yes | Yes | Yes | 0.076 | 0.088 | -1.146 | -2.829 | No | Yes | Yes | Yes | Yes | No | Yes | 0.137 | No | No | -0.11 | No | Yes | 2.582 | 1.154 | Yes |
| CHEMBL4594253 | Cn1cc(-c2nc(N[C@@H]3CCCC[C@@H]3N)c(F)c3c2C(=O)NC3)cn1.O=C(O)CC(O)(CC(=O)O)C(=O)O | 536.517 | 0.2979 | 8 | 10 | 7 | 215.212 | -2.858 | -0.988 | 0 | -2.735 | Yes | No | No | -0.977 | 0.45 | -2.374 | -4.219 | No | No | No | No | No | No | No | 0.557 | No | No | 0.731 | No | No | 2.406 | 3.613 | Yes |
| CHEMBL812 | CN(C)C(=O)Oc1ccc[n+](C)c1.[Br-] | 261.119 | -2.4245 | 1 | 2 | 0 | 93.868 | -0.243 | 1.684 | 100 | -2.97 | No | No | No | -0.173 | 0.867 | -0.073 | -3.13 | No | No | No | No | No | No | No | 0.94 | No | No | 0.509 | No | No | 2.32 | 1.156 | No |
| CHEMBL99 | CC(/C=C/C(=O)NO)=C\[C@@H](C)C(=O)c1ccc(N(C)C)cc1 | 302.374 | 2.5793 | 6 | 4 | 2 | 130.448 | -3.772 | 0.972 | 94.338 | -3.529 | Yes | No | No | -0.352 | 0.179 | -0.281 | -2.583 | No | No | No | No | No | No | No | 0.733 | No | Yes | 0.577 | No | Yes | 1.81 | 1.876 | No |
| CHEMBL1201772 | CC(=O)Oc1cc2c(s1)CCN(C(C(=O)C1CC1)c1ccccc1F)C2 | 373.449 | 3.891 | 5 | 5 | 0 | 155.129 | -3.566 | 1.367 | 91.262 | -2.953 | Yes | Yes | Yes | 0.227 | 0.137 | 0.394 | -1.459 | No | Yes | No | No | No | Yes | Yes | 1.141 | Yes | No | 0.161 | No | Yes | 3.088 | 1.858 | Yes |
| CHEMBL3989931 | CC(C)(O)CNc1nc(Nc2ccnc(C(F)(F)F)c2)nc(-c2cccc(C(F)(F)F)n2)n1.CS(=O)(=O)O | 569.488 | 3.7966 | 6 | 10 | 4 | 211.72 | -3.151 | -0.469 | 40.833 | -2.735 | No | No | No | -1.185 | 0.285 | -2.732 | -3.932 | No | Yes | No | No | No | No | No | -1.166 | No | No | 0.699 | No | No | 2.02 | 1.308 | Yes |
| CHEMBL2079588 | CNC(=O)c1cc(COc2nnc(Nc3ccc(Cl)cc3)c3ccoc23)ccn1 | 409.833 | 3.9534 | 6 | 7 | 2 | 170.422 | -3.147 | 1.823 | 90.62 | -2.735 | No | Yes | Yes | 0.179 | 0.074 | -0.873 | -2.766 | No | Yes | Yes | Yes | No | No | Yes | -0.123 | No | No | 0.384 | No | Yes | 2.831 | 1.673 | Yes |
| CHEMBL3989922 | Cc1ccc(S(=O)(=O)O)cc1.NC(=O)c1cccc2cn(-c3ccc([C@@H]4CCCNC4)cc3)nc12.O | 510.616 | 3.00842 | 4 | 6 | 3 | 209.55 | -3.599 | -0.363 | 57.947 | -2.735 | Yes | No | Yes | -0.754 | 0.044 | -0.96 | -2.453 | No | No | No | No | No | No | No | 0.641 | No | No | 0.885 | No | No | 3.137 | 2.169 | Yes |
| CHEMBL371064 | CCCN1CCN(c2ccc(C(=O)NC3(C(=O)NCC#N)CCCCC3)cc2)CC1 | 411.55 | 2.29108 | 7 | 5 | 2 | 178.959 | -3.631 | 1.007 | 92.24 | -3.422 | Yes | No | No | 0.602 | 0.395 | -0.454 | -2.978 | No | Yes | No | No | No | No | No | 0.817 | No | No | -0.38 | No | Yes | 2.811 | 1.081 | Yes |
| CHEMBL511099 | Cc1ccc(C23CNCC2C3)cc1 | 173.259 | 1.85592 | 1 | 1 | 1 | 79.159 | -2.103 | 1.349 | 94.811 | -2.135 | No | No | No | 1.455 | 0.435 | 0.719 | -1.68 | Yes | Yes | Yes | No | No | Yes | No | 1.125 | Yes | No | 0.059 | No | No | 2.509 | 1.941 | Yes |
| CHEMBL924 | O=P(O)(O)C(O)(Cn1ccnc1)P(=O)(O)O | 272.09 | -1.1154 | 4 | 5 | 5 | 89.697 | -2.646 | -0.533 | 35.926 | -2.735 | Yes | No | No | -0.871 | 0.777 | -2.44 | -5.661 | No | No | No | No | No | No | No | 0.426 | No | Yes | 0.19 | No | No | 2.593 | 4.397 | Yes |
| CHEMBL4435170 | [2H]C([2H])([2H])NC(=O)c1nnc(NC(=O)C2CC2)cc1Nc1cccc(-c2ncn(C)n2)c1OC | 425.4673 | 1.7324 | 8 | 9 | 3 | 181.995 | -3.596 | -0.157 | 80.621 | -2.766 | Yes | Yes | No | 0.195 | 0.104 | -1.553 | -3.249 | No | Yes | No | No | No | No | Yes | -0.223 | No | No | 0.094 | No | Yes | 2.866 | 1.237 | Yes |
| CHEMBL4594292 | CN1CCC(COc2cnc(-c3cccc(Cn4nc(-c5cccc(C#N)c5)ccc4=O)c3)nc2)CC1.Cl.O | 547.059 | 3.60498 | 7 | 8 | 0 | 233 | -3.576 | 0.497 | 82.618 | -2.736 | No | Yes | Yes | 1.01 | 0.197 | -1.558 | -2.622 | No | Yes | No | No | Yes | No | Yes | 0.698 | No | No | 0.461 | No | Yes | 2.878 | 1.022 | Yes |
| CHEMBL603469 | C[C@]12O[C@H](C[C@]1(O)CO)n1c3ccccc3c3c4c(c5c6ccccc6n2c5c31)CNC4=O | 439.471 | 3.4744 | 1 | 6 | 3 | 187.312 | -3.27 | 0.946 | 98.81 | -2.735 | Yes | Yes | Yes | -1.224 | 0.19 | -0.701 | -2.274 | No | Yes | No | Yes | Yes | No | No | 0.548 | No | Yes | 0.297 | No | Yes | 2.557 | 0.737 | Yes |
| CHEMBL1324 | Cc1ccc(C(=O)c2cc(O)c(O)c([N+](=O)[O-])c2)cc1 | 273.244 | 2.54542 | 3 | 5 | 2 | 113.621 | -3.396 | 0.322 | 80.9 | -2.736 | Yes | No | No | -0.084 | 0.034 | -0.185 | -2.292 | No | No | Yes | Yes | Yes | No | No | 0.305 | No | No | 0.202 | No | No | 2.402 | 2.405 | No |
| CHEMBL766 | CC1(C)C[C@@H]1C(=O)N/C(=C\CCCCSC[C@H](N)C(=O)O)C(=O)O | 358.46 | 1.4326 | 11 | 5 | 4 | 145.604 | -2.17 | -0.399 | 15.91 | -2.735 | No | No | No | -1.092 | 0.57 | -1.164 | -3.611 | No | No | No | No | No | No | No | 0.415 | No | No | -0.021 | No | No | 2.082 | 1.638 | Yes |
| CHEMBL1515 | Cn1cc[nH]c1=S | 114.173 | 1.08269 | 0 | 2 | 1 | 46.794 | -2.012 | 1.465 | 97.365 | -2.735 | Yes | No | No | -0.261 | 0.699 | -0.199 | -2.41 | No | No | No | No | No | No | No | 0.696 | No | No | -0.761 | No | No | 2.64 | 1.392 | No |
| CHEMBL3707348 | CC#CC(=O)N1CCC[C@H]1c1nc(-c2ccc(C(=O)Nc3ccccn3)cc2)c2c(N)nccn12 | 465.517 | 3.3126 | 4 | 7 | 2 | 201.573 | -2.988 | 1.547 | 82.298 | -2.735 | Yes | Yes | No | 0.521 | 0.226 | -1.059 | -3.123 | No | Yes | No | Yes | Yes | No | Yes | 0.394 | Yes | Yes | 0.297 | No | Yes | 2.48 | 0.146 | Yes |
| CHEMBL1950289 | O[C@H]1CC[C@H](Nc2ncc3nc(Nc4c(F)cc(F)cc4F)n([C@H]4CCOC4)c3n2)CC1 | 448.449 | 3.664 | 5 | 8 | 3 | 181.39 | -2.973 | 1.405 | 96.045 | -2.735 | Yes | No | No | 0.826 | 0.548 | -1.88 | -3.229 | No | No | No | No | No | No | Yes | 0.852 | No | No | 0.386 | No | Yes | 2.158 | 1.527 | Yes |
| CHEMBL183419 | C#N | 27.026 | 0.13978 | 0 | 1 | 0 | 13.132 | 0.162 | 1.346 | 100 | -2.88 | Yes | No | No | -0.109 | 0.778 | -0.061 | -2.375 | No | No | No | No | No | No | No | 0.745 | No | No | 1.253 | No | No | 2.351 | 1.576 | No |
| CHEMBL84 | CC[C@@]1(O)C(=O)OCc2c1cc1n(c2=O)Cc2cc3c(CN(C)C)c(O)ccc3nc2-1 | 421.453 | 1.8468 | 3 | 8 | 2 | 177.877 | -3.367 | 0.852 | 82.762 | -2.737 | Yes | Yes | No | 0.75 | 0.191 | -1.229 | -3.53 | No | Yes | No | No | No | No | No | 1.207 | No | No | 0.377 | No | Yes | 2.982 | 2.014 | Yes |
| CHEMBL31634 | O=c1[nH]c(=O)n([C@H]2C[C@H](O)[C@@H](CO)O2)cc1/C=C/Br | 333.138 | -0.4571 | 3 | 6 | 3 | 115.832 | -2.659 | 0.343 | 61.702 | -2.859 | No | No | No | -0.383 | 0.631 | -1.204 | -3.541 | No | No | No | No | No | No | No | 0.212 | No | No | 0.745 | No | No | 2.215 | 2.487 | Yes |
| CHEMBL783 | CC(C)[C@H]1CC[C@H](C(=O)N[C@H](Cc2ccccc2)C(=O)O)CC1 | 317.429 | 3.2609 | 6 | 2 | 2 | 137.837 | -3.227 | 0.906 | 100 | -2.735 | Yes | No | No | -1.768 | 0.078 | -0.032 | -2.249 | No | No | No | No | No | No | No | 1.282 | No | No | 0.229 | No | No | 2.016 | 1.949 | No |
| CHEMBL1614644 | C[N+](C)([O-])CCCN1c2ccccc2CCc2ccccc21 | 296.414 | 3.8876 | 4 | 2 | 0 | 132.515 | -5.113 | 1.456 | 98.889 | -2.255 | No | Yes | No | 0.991 | 0.027 | 0.431 | -1.495 | No | Yes | Yes | Yes | No | No | No | 0.46 | Yes | No | 0.286 | No | Yes | 2.29 | 0.447 | No |
| CHEMBL238804 | CC(C)N(CCCCOCC(=O)NS(C)(=O)=O)c1cnc(-c2ccccc2)c(-c2ccccc2)n1 | 496.633 | 3.898 | 12 | 7 | 1 | 206.98 | -5.687 | 0.617 | 91.229 | -2.724 | No | Yes | Yes | 0.116 | 0.126 | -1.351 | -3.439 | No | Yes | No | Yes | Yes | No | Yes | 0.872 | No | No | 0.475 | No | Yes | 2.553 | 0.362 | Yes |
| CHEMBL4297584 | CC[C@H](Nc1ncnc2[nH]cnc12)c1oc2ccccc2c(=O)c1-c1cccc(F)c1 | 415.428 | 4.8286 | 5 | 6 | 2 | 175.554 | -2.908 | 0.784 | 89.243 | -2.735 | Yes | Yes | Yes | -0.11 | 0.323 | -1.612 | -2.365 | No | No | Yes | Yes | Yes | No | Yes | 0.513 | No | No | 0.417 | No | Yes | 2.592 | 2.398 | No |
| CHEMBL779 | CN1CC(=O)N2[C@H](Cc3c([nH]c4ccccc34)[C@H]2c2ccc3c(c2)OCO3)C1=O | 389.411 | 2.2113 | 1 | 4 | 1 | 166.351 | -3.782 | 0.986 | 97.096 | -2.743 | Yes | Yes | Yes | 0.243 | 0.171 | -0.567 | -2.181 | No | Yes | No | Yes | No | No | No | 0.335 | No | No | 0.161 | No | No | 2.696 | 0.534 | Yes |
| CHEMBL939 | COc1cc2ncnc(Nc3ccc(F)c(Cl)c3)c2cc1OCCCN1CCOCC1 | 446.91 | 4.2756 | 8 | 7 | 1 | 184.642 | -4.608 | 1.197 | 92.629 | -2.736 | Yes | Yes | Yes | 1.032 | 0.146 | -0.719 | -3.259 | No | Yes | Yes | No | No | No | Yes | 0.963 | Yes | No | 0.304 | No | Yes | 2.688 | 1.491 | Yes |
| CHEMBL4297507 | C[C@H]1CN(C(=O)CC#N)[C@]12CCN(c1ncnc3[nH]ccc13)C2 | 310.361 | 1.29878 | 2 | 5 | 1 | 133.473 | -3.381 | 1.343 | 79.291 | -3.212 | Yes | No | No | 0.209 | 0.418 | -0.768 | -3.529 | No | No | Yes | No | No | No | No | 0.742 | No | No | -0.455 | No | No | 2.425 | 0.406 | Yes |
| CHEMBL838 | CCOC(=O)[C@H](CCc1ccccc1)N[C@H]1CCc2ccccc2N(CC(=O)O)C1=O | 424.497 | 2.5731 | 9 | 5 | 2 | 181.564 | -3.33 | 0.264 | 55.836 | -2.733 | Yes | No | No | 0.018 | 0.41 | -0.431 | -2.879 | No | No | No | No | No | No | No | 0.631 | No | No | -0.096 | No | No | 2.739 | 2.125 | Yes |
| CHEMBL14762 | CC[C@H](CO)Nc1nc(NCc2ccccc2)c2ncn(C(C)C)c2n1 | 354.458 | 3.2021 | 8 | 7 | 3 | 153.062 | -3.118 | 1.186 | 86.717 | -2.735 | Yes | Yes | No | 0.391 | 0.186 | -1.02 | -2.776 | No | No | Yes | No | Yes | Yes | Yes | 1.072 | No | Yes | 0.163 | No | Yes | 2.249 | 1.708 | No |
| CHEMBL435966 | O=[N+]([O-])c1cncn1CCN1CCOCC1 | 226.236 | 0.1235 | 4 | 6 | 0 | 92.78 | -2.219 | 0.854 | 99.531 | -2.779 | Yes | No | No | -0.485 | 0.493 | -0.655 | -3.065 | No | No | No | No | No | No | No | 0.665 | No | Yes | 0.282 | No | No | 2.192 | 0.801 | No |
| CHEMBL19 | CC(=O)/N=c1/sc(S(N)(=O)=O)nn1C | 236.278 | -1.4238 | 1 | 6 | 1 | 83.942 | -2.732 | 0.046 | 73.63 | -3.118 | No | No | No | -0.781 | 0.726 | -1.005 | -3.222 | No | No | No | No | No | No | No | -0.082 | No | No | 1.227 | No | No | 2.962 | 0.521 | No |
| CHEMBL1770248 | CCOc1ccc(Cc2cc([C@]34OC[C@](CO)(O3)[C@@H](O)[C@H](O)[C@H]4O)ccc2Cl)cc1 | 436.888 | 1.3565 | 6 | 7 | 4 | 178.947 | -4.216 | -0.012 | 47.712 | -2.735 | Yes | Yes | No | -0.701 | 0.109 | -1.236 | -3.797 | No | No | No | No | No | No | No | 0.254 | No | No | -0.194 | No | Yes | 3.114 | 3.621 | No |
| CHEMBL231779 | COc1ccc(-n2nc(C(N)=O)c3c2C(=O)N(c2ccc(N4CCCCC4=O)cc2)CC3)cc1 | 459.506 | 2.6996 | 5 | 6 | 1 | 196.248 | -4.387 | 0.872 | 97.951 | -2.75 | No | Yes | Yes | -0.175 | 0.071 | -0.916 | -2.62 | No | Yes | No | Yes | Yes | No | Yes | 0.33 | No | No | 0.234 | No | Yes | 2.671 | 1.259 | Yes |
| CHEMBL266349 | N=C(N)c1ccc(CNC(=O)[C@@H]2CCN2C(=O)[C@H](NCC(=O)O)C2CCCCC2)cc1 | 429.521 | 0.81087 | 9 | 5 | 5 | 181.422 | -3.508 | -0.423 | 34.59 | -2.735 | Yes | No | No | 0.006 | 0.531 | -0.8 | -3.222 | No | No | No | No | No | No | No | 0.791 | No | No | 0.015 | No | No | 2.034 | 2.415 | Yes |
| CHEMBL3916717 | Cc1cc(F)c(C(=O)Nc2cccc(-c3nncn3C(C)C)n2)cc1-n1cnc(C2CC2)c1 | 445.502 | 4.68392 | 6 | 7 | 1 | 190.079 | -2.925 | 1.342 | 86.971 | -2.735 | Yes | Yes | Yes | -0.423 | 0.229 | -0.867 | -3.342 | No | Yes | Yes | Yes | Yes | No | Yes | 0.72 | Yes | No | 0.085 | No | Yes | 2.431 | 0.95 | No |
| CHEMBL46618 | COC(=O)[C@H](Cc1cccc(C(=N)N)c1)[C@@H](C)NC(=O)c1ccc(-c2cc[n+]([O-])cc2)cc1 | 446.507 | 2.42127 | 8 | 5 | 3 | 191.655 | -4.169 | 0.519 | 75.121 | -2.769 | Yes | Yes | Yes | 0.145 | 0.114 | -0.865 | -2.886 | No | Yes | No | Yes | No | No | Yes | 0.599 | No | No | 0.044 | No | Yes | 2.925 | 0.662 | Yes |
| CHEMBL4802152 | O=C(c1cc(Cn2c(=O)[nH]c(=O)c3c(F)cccc32)ccc1F)N1CCN(c2ncccn2)CC1 | 478.459 | 1.7687 | 4 | 7 | 1 | 196.835 | -3.784 | 0.29 | 78.957 | -2.735 | Yes | Yes | Yes | -0.02 | 0.123 | -1.458 | -3.497 | No | Yes | No | No | Yes | No | Yes | 0.399 | No | Yes | 0.685 | No | Yes | 2.57 | 1.315 | Yes |
| CHEMBL4448462 | Cc1cncc2cccc(S(=O)(=O)N3CCN(C(=O)CN)CC[C@H]3C)c12 | 376.482 | 1.11342 | 3 | 5 | 1 | 153.393 | -3.038 | 1.014 | 84.521 | -2.938 | Yes | No | No | 0.294 | 0.3 | -0.787 | -3.008 | No | No | No | No | No | No | No | 0.072 | No | Yes | 0.359 | No | Yes | 2.335 | 2.04 | Yes |
| CHEMBL189134 | CC(C)C[C@H](C[C@H](O)C(Cc1ccccc1)NC(=O)OC(C)(C)C)C(=O)NC(C(N)=O)C(C)C | 477.646 | 3.1618 | 12 | 5 | 4 | 203.562 | -3.955 | 0.13 | 55.011 | -2.698 | Yes | Yes | Yes | -0.276 | 0.167 | -0.825 | -2.85 | No | Yes | No | No | No | No | Yes | 0.94 | No | No | -0.791 | No | Yes | 3.341 | 2.864 | No |
| CHEMBL590754 | COc1ccc(/C(C)=N/OC(N)=O)cc1OC1CCCC1 | 292.335 | 2.8359 | 5 | 5 | 1 | 123.401 | -3.492 | 1.086 | 92.139 | -3.647 | No | No | No | -0.282 | 0.327 | -0.539 | -2.932 | No | No | No | Yes | No | No | No | 0.422 | No | No | 0.56 | No | No | 2.648 | 1.352 | No |
| CHEMBL1164729 | Cc1nc(-c2ccc(OCC(C)C)c(C#N)c2)sc1C(=O)O | 316.382 | 3.7232 | 5 | 5 | 1 | 132.365 | -3.181 | 0.89 | 94.132 | -2.735 | No | No | No | -1.308 | 0.288 | -0.576 | -2.217 | No | No | No | No | No | No | No | 0.303 | No | No | 0.817 | No | No | 2.682 | 1.139 | No |
| CHEMBL2105720 | O=C(O)CSc1nnc(Br)n1-c1ccc(C2CC2)c2ccccc12 | 404.289 | 4.2371 | 5 | 5 | 1 | 150.762 | -4.775 | 0.991 | 94.013 | -2.73 | No | No | Yes | -0.314 | 0.132 | -0.707 | -2.286 | No | Yes | Yes | No | Yes | No | No | 0.024 | No | No | 0.104 | No | No | 2.538 | 0.511 | Yes |
| CHEMBL4285417 | C[C@@H]1COCCN1c1cc(C2([S@](C)(=N)=O)CC2)nc(-c2cc[nH]c3nccc2-3)n1 | 412.519 | 2.86227 | 4 | 7 | 2 | 169.607 | -4.351 | 0.773 | 90.342 | -3.228 | Yes | No | No | -0.181 | 0.253 | -1.387 | -3.541 | No | Yes | No | No | No | No | No | 0.589 | No | No | -0.473 | No | No | 3.489 | 1.555 | Yes |
| CHEMBL2219422 | Cn1ncc(Cl)c1-c1cc(C(=O)N[C@H](CN)Cc2cccc(F)c2)sc1Cl | 427.332 | 3.8943 | 6 | 5 | 2 | 169.441 | -4.847 | 1.059 | 89.827 | -2.802 | Yes | Yes | Yes | 0.618 | 0.09 | -0.586 | -2.254 | No | Yes | Yes | No | No | No | Yes | 0.6 | Yes | No | 0.144 | No | Yes | 2.05 | 2.124 | Yes |
| CHEMBL1308 | Cc1cn[nH]c1 | 82.106 | 0.71812 | 0 | 1 | 1 | 36.194 | -0.604 | 1.603 | 92.157 | -3.126 | No | No | No | -0.079 | 0.745 | -0.321 | -2.819 | No | No | No | No | No | No | No | 0.608 | No | No | 0.836 | No | No | 2.151 | 1.687 | No |
| CHEMBL374731 | Cc1cn([C@@H]2C[C@@H](O)[C@H](CO)O2)c(=O)[nH]c1=O | 242.231 | -1.51428 | 2 | 6 | 3 | 96.29 | -2.77 | -0.123 | 60.686 | -3.029 | No | No | No | -0.295 | 0.761 | -0.982 | -3.649 | No | No | No | No | No | No | No | 0.729 | No | No | 0.969 | No | No | 2.094 | 2.752 | Yes |
| CHEMBL370753 | OCCN1CCN(CCCN2c3ccccc3C=Cc3ccccc32)CC1 | 363.505 | 3.3085 | 6 | 4 | 1 | 161.875 | -3.224 | 0.963 | 94.273 | -2.751 | Yes | Yes | Yes | 2.293 | 0.131 | 0.652 | -2.007 | Yes | Yes | Yes | No | No | Yes | No | 0.762 | Yes | No | 0.109 | No | Yes | 2.725 | 0.189 | No |
| CHEMBL3707393 | N#Cc1ccc([C@H]2CCc3cncn32)c(F)c1.O=P(O)(O)O | 325.236 | 1.50088 | 1 | 4 | 3 | 125.077 | -2.414 | 0.371 | 77.493 | -2.819 | Yes | No | No | -0.213 | 0.414 | -1.629 | -4.182 | No | No | No | No | No | No | No | 0.71 | No | Yes | 0.527 | No | No | 2.322 | 2.766 | Yes |
| CHEMBL4297190 | Cc1cc2cc(Oc3ccnc(Nc4cccc(CS(=O)(=O)NCCN(C)C)c4)n3)ccc2[nH]1 | 480.594 | 3.78322 | 10 | 7 | 3 | 198.799 | -3.554 | 0.337 | 76.705 | -2.735 | Yes | Yes | Yes | 1.047 | 0.049 | -1.24 | -2.916 | No | No | Yes | Yes | No | No | Yes | 0.553 | No | No | 0.665 | No | Yes | 2.949 | 2.127 | Yes |
| CHEMBL2338397 | C[C@H](NC(=O)CCC(F)(F)F)C(=O)N[C@@H]1C(=O)N(CCO)c2ncccc2-c2ccccc21 | 464.444 | 2.092 | 7 | 5 | 3 | 187.352 | -4.355 | 0.936 | 74.352 | -2.741 | Yes | Yes | No | -0.797 | 0.026 | -1.242 | -3.254 | No | Yes | No | No | No | No | Yes | 0.037 | No | No | 0.417 | No | Yes | 2.357 | 1.016 | Yes |
| CHEMBL3982690 | Cc1noc(C)c1-c1cc(C(O)(c2ccccn2)c2ccccn2)c2nc(C3CC3)[nH]c2c1 | 437.503 | 4.78634 | 5 | 6 | 2 | 190.096 | -2.892 | 0.387 | 100 | -2.735 | Yes | No | Yes | 0.011 | 0.339 | -1.335 | -2.422 | No | No | No | Yes | Yes | No | Yes | 1.043 | Yes | No | 0.396 | No | Yes | 2.476 | 1.381 | Yes |
| CHEMBL192 | CCCc1nn(C)c2c(=O)[nH]c(-c3cc(S(=O)(=O)N4CCN(C)CC4)ccc3OCC)nc12 | 474.587 | 1.6109 | 7 | 8 | 1 | 192.781 | -3.341 | 0.222 | 76.297 | -2.741 | Yes | Yes | No | 0.528 | 0.221 | -1.196 | -3.237 | No | Yes | No | No | No | No | Yes | 0.22 | No | No | -0.032 | No | Yes | 2.373 | 1.656 | Yes |
| CHEMBL2336325 | CNC(=O)c1cccc(-c2ccc3c(N4CCOC[C@@H]4C)nc(N4CCOC[C@@H]4C)nc3n2)c1 | 462.554 | 2.5016 | 4 | 8 | 1 | 198.655 | -3.716 | 0.561 | 100 | -2.901 | No | Yes | No | -0.218 | 0.153 | -1.021 | -3.071 | No | Yes | No | No | No | No | Yes | 0.122 | No | No | -0.957 | No | Yes | 3.296 | 1.305 | Yes |
| CHEMBL18116 | Cc1cccc(N2CC(CO)OC2=O)c1 | 207.229 | 1.31252 | 2 | 3 | 1 | 88.079 | -1.66 | 1.244 | 87.372 | -3.041 | No | No | No | -0.039 | 0.412 | -0.168 | -2.856 | No | No | Yes | No | No | No | No | 0.207 | No | No | 0.709 | No | No | 2.388 | 2.377 | No |
| CHEMBL2105279 | COC(=O)Nc1cccc(C)c1CNc1cccn2c(C)c(C)nc12 | 338.411 | 4.04996 | 4 | 5 | 2 | 146.391 | -2.551 | 0.743 | 93.336 | -2.735 | Yes | No | No | 0.604 | 0.357 | -0.579 | -2.855 | No | Yes | Yes | No | No | No | No | 0.912 | No | Yes | -0.048 | No | Yes | 2.796 | 0.192 | Yes |
| CHEMBL571546 | O=C(Cc1ccc(-c2ccc(OCCN3CCOCC3)cc2)cn1)NCc1ccccc1 | 431.536 | 3.3185 | 9 | 5 | 1 | 188.742 | -4.16 | 1.014 | 94.818 | -2.755 | Yes | Yes | Yes | 1.225 | 0.108 | -0.118 | -2.571 | Yes | Yes | No | No | Yes | No | Yes | 1.018 | Yes | No | -0.144 | No | Yes | 2.599 | 1.011 | Yes |
| CHEMBL3039513 | CC[C@@](C)(Nc1ccnc(-c2c[nH]c3ncccc23)n1)C(=O)NCC(F)(F)F | 392.385 | 3.279 | 6 | 5 | 3 | 158.351 | -3.483 | 0.626 | 82.755 | -2.76 | Yes | No | No | 0.418 | 0.255 | -1.793 | -3.504 | No | No | Yes | No | No | No | No | 0.362 | No | No | 0.33 | No | Yes | 2.51 | 0.388 | Yes |
| CHEMBL1851943 | CCCCc1nc2cc(/C=C/C(=O)NO)ccc2n1CCN(CC)CC | 358.486 | 3.2393 | 10 | 5 | 2 | 155.026 | -2.935 | 0.891 | 79.781 | -2.735 | Yes | Yes | No | -0.582 | 0.129 | -0.612 | -2.929 | No | No | No | No | No | No | No | 1.163 | Yes | Yes | 0.073 | No | Yes | 2.519 | 1.597 | No |
| CHEMBL2005186 | CC(C)NC(=O)COc1cccc(-c2nc(Nc3ccc4[nH]ncc4c3)c3ccccc3n2)c1 | 452.518 | 4.8201 | 7 | 6 | 3 | 195.945 | -3.131 | 0.712 | 95.105 | -2.735 | Yes | Yes | Yes | -0.625 | 0.178 | -1.424 | -2.421 | No | Yes | Yes | Yes | Yes | Yes | Yes | 0.324 | No | Yes | 0.484 | No | Yes | 2.95 | 1.743 | Yes |
| CHEMBL1678 | COc1cc2c(cc1OC)C(=O)C(CC1CCN(Cc3ccccc3)CC1)C2.Cl | 415.961 | 4.7829 | 6 | 4 | 0 | 179.158 | -5.094 | 1.318 | 91.73 | -2.655 | Yes | Yes | Yes | 1.157 | 0 | 0.475 | -1.431 | Yes | Yes | No | No | No | Yes | Yes | 1.241 | Yes | No | -0.189 | No | Yes | 2.879 | 2.176 | Yes |
| CHEMBL4072833 | C=CC(=O)N1CCC(CNc2ncnc(N)c2-c2ccc(Oc3ccccc3)cc2)CC1 | 429.524 | 4.3546 | 7 | 6 | 2 | 187.499 | -5.004 | 0.901 | 91.607 | -2.738 | Yes | Yes | Yes | 0.092 | 0.085 | -0.9 | -2.382 | No | Yes | Yes | Yes | Yes | No | Yes | 0.401 | No | No | 0.602 | No | Yes | 2.905 | 0.803 | Yes |
| CHEMBL4297497 | NCc1ccc2c(c1)nc(CN1C(=O)C3(CC3)c3ccc(F)cc31)n2CCCC(F)(F)F | 446.448 | 4.555 | 6 | 4 | 1 | 181.37 | -2.902 | 1.401 | 81.643 | -2.735 | Yes | Yes | Yes | -0.632 | 0.258 | -0.275 | -2.049 | No | Yes | No | Yes | Yes | No | Yes | 1.215 | Yes | Yes | -0.281 | No | Yes | 2.282 | 0.942 | No |
| CHEMBL4802130 | CC(C)NC(=O)COc1cccc(-c2nc(Nc3ccc4[nH]ncc4c3)c3ccccc3n2)c1.CS(=O)(=O)O | 548.625 | 4.3241 | 7 | 8 | 4 | 224.944 | -2.985 | -0.791 | 53.073 | -2.735 | No | No | Yes | -0.875 | 0.161 | -1.922 | -3.632 | No | Yes | No | No | Yes | No | No | 0.012 | No | No | 0.499 | No | Yes | 2.613 | 2.196 | Yes |
| CHEMBL3545376 | COc1cc(OC)cc(N(CCNC(C)C)c2ccc3ncc(-c4cnn(C)c4)nc3c2)c1 | 446.555 | 4.1836 | 9 | 8 | 1 | 193.73 | -4.694 | 1.034 | 96.555 | -2.74 | Yes | Yes | Yes | 0.781 | 0.17 | -0.752 | -2.591 | No | Yes | Yes | Yes | Yes | No | Yes | 0.786 | Yes | No | 0.563 | No | Yes | 3.23 | 1.106 | Yes |
| CHEMBL227529 | Cn1c(=O)cc(N2CCC[C@@H](N)C2)n(Cc2ccccc2C#N)c1=O.O=C(O)c1ccccc1 | 461.522 | 1.77928 | 4 | 8 | 2 | 196.46 | -4.044 | -0.043 | 56.303 | -2.735 | Yes | No | No | -0.29 | 0.194 | -0.652 | -2.524 | No | Yes | No | No | No | No | No | 0.144 | No | No | -0.137 | No | No | 2.351 | 2.238 | Yes |
| CHEMBL376359 | Cn1c(=O)cc(N2CCC[C@@H](N)C2)n(Cc2ccccc2C#N)c1=O | 339.399 | 0.39448 | 3 | 7 | 1 | 145.077 | -2.57 | 0.281 | 79.038 | -3.086 | No | No | No | 0.381 | 0.369 | -0.566 | -2.685 | No | Yes | No | No | No | No | No | 0.365 | No | Yes | -0.685 | No | Yes | 2.401 | 1.438 | Yes |
| CHEMBL4594251 | CS(=O)(=O)O.CS(=O)(=O)O.Cc1ccc(C(=O)OCc2ccc([C@@H](CN)C(=O)Nc3ccc4cnccc4c3)cc2)c(C)c1 | 645.756 | 3.89764 | 7 | 9 | 4 | 256.327 | -2.954 | -0.672 | 13.258 | -2.735 | Yes | No | Yes | -0.777 | 0.118 | -1.962 | -3.481 | No | Yes | No | No | No | No | No | 0.344 | No | No | 0.438 | No | Yes | 2.42 | 2.804 | Yes |
| CHEMBL4442620 | C[C@@H](O)[C@@H](C)Oc1nc(Nc2ccc(S(=N)(=O)C3CC3)cc2)ncc1C(F)(F)F | 430.452 | 3.95517 | 7 | 7 | 3 | 165.864 | -3.697 | 0.593 | 81.062 | -2.743 | Yes | Yes | Yes | 0.271 | 0.131 | -1.584 | -2.769 | No | No | No | No | Yes | No | No | 0.694 | No | No | 0.411 | No | No | 2.412 | 0.181 | Yes |
| CHEMBL1401 | CC(=O)Oc1ccccc1C(=O)Nc1ncc([N+](=O)[O-])s1 | 307.287 | 2.2289 | 4 | 7 | 1 | 122.083 | -3.923 | 1.156 | 87.937 | -2.759 | No | No | No | -0.753 | 0.081 | -0.94 | -2.654 | No | No | Yes | Yes | No | No | Yes | -0.041 | No | Yes | 0.669 | No | No | 2.414 | 0.967 | Yes |
| CHEMBL1278146 | CCN(CC)CCN1CCc2[nH]c(/C=C3\C(=O)Nc4ccc(F)cc43)c(C)c2C1=O | 410.493 | 3.29502 | 6 | 3 | 2 | 175.299 | -3.773 | 1.027 | 91.668 | -3.015 | Yes | Yes | Yes | 1.007 | 0.136 | -0.871 | -2.436 | Yes | Yes | No | No | No | No | No | 1.078 | No | No | -0.585 | No | Yes | 2.841 | 0.785 | Yes |
| CHEMBL2103840 | CCc1cnn2c(NCc3ccc[n+]([O-])c3)cc(N3CCCC[C@H]3CCO)nc12 | 396.495 | 2.2785 | 7 | 7 | 2 | 169.94 | -4.016 | 0.943 | 84.948 | -2.986 | Yes | No | No | 0.328 | 0.46 | -0.879 | -3.02 | No | Yes | No | No | No | No | No | 1.015 | No | No | -0.51 | No | Yes | 2.573 | 0.665 | Yes |
| CHEMBL3039502 | C[C@H](Nc1ncnc2nc[nH]c12)c1cc2cccc(Cl)c2c(=O)n1-c1ccccc1 | 416.872 | 4.4835 | 4 | 6 | 2 | 175.951 | -2.914 | 1.176 | 95.371 | -2.735 | Yes | Yes | Yes | -0.099 | 0.268 | -1.338 | -2.291 | No | No | Yes | No | Yes | No | Yes | 0.566 | Yes | Yes | 0.435 | No | Yes | 2.663 | 2.029 | Yes |
| CHEMBL853 | Nc1ccn([C@H]2CC[C@@H](CO)O2)c(=O)n1 | 211.221 | -0.5046 | 2 | 6 | 2 | 86.309 | -1.669 | 0.809 | 80.864 | -3.214 | No | No | No | -0.432 | 0.685 | -0.571 | -3.137 | No | No | No | No | No | No | No | 0.482 | No | Yes | 0.836 | No | No | 2.151 | 0.892 | Yes |
| CHEMBL3137303 | COc1cc(OC)c(/C=C/S(=O)(=O)Cc2ccc(OC)c(OP(=O)([O-])[O-])c2)c(OC)c1.[Na+].[Na+] | 518.388 | -4.4778 | 10 | 10 | 0 | 236.511 | -3.602 | 0.666 | 40.182 | -2.735 | No | Yes | No | -0.836 | 0.088 | -2.067 | -4.484 | No | Yes | No | No | No | No | No | -1.137 | No | No | 0.866 | No | No | 2.522 | 1.533 | No |
| CHEMBL1332032 | CC(=O)Oc1cc(C(F)(F)F)ccc1C(=O)O | 248.156 | 2.3289 | 2 | 3 | 1 | 93.619 | -2.032 | 1.118 | 98.82 | -2.719 | No | No | No | -1.638 | 0.466 | -0.217 | -2.94 | No | No | No | No | No | No | No | 0.637 | No | No | 1.036 | No | No | 2.532 | 2.402 | No |
| CHEMBL4297596 | Cn1c(=O)oc2cc(-n3cc(C(=O)O)c(=O)n([C@@H]4CCc5c4cccc5C(F)(F)F)c3=O)ccc21 | 487.39 | 2.6967 | 3 | 8 | 1 | 192.431 | -3.817 | 0.492 | 74.615 | -2.734 | No | No | No | -1.236 | 0.149 | -1.101 | -2.467 | No | Yes | No | No | No | No | No | 0.639 | No | No | 0.721 | No | No | 3.07 | 0.491 | Yes |
| CHEMBL887 | C#CCN[C@@H]1CCc2ccccc21 | 171.243 | 1.8967 | 2 | 1 | 1 | 79.164 | -1.822 | 1.521 | 94.671 | -2.049 | No | No | No | 1.078 | 0.458 | 0.624 | -1.723 | No | No | No | No | No | Yes | No | 1.209 | No | No | 0.311 | No | No | 2.538 | 1.13 | Yes |
| CHEMBL4594441 | COc1cc(Nc2ncc(C)c(Nc3ccc4oc(=O)[nH]c4c3)n2)cc(C)c1F | 395.394 | 4.16284 | 5 | 7 | 3 | 164.316 | -3.735 | 0.658 | 98.206 | -2.737 | Yes | Yes | Yes | 0.095 | 0.09 | -1.433 | -2.314 | No | No | Yes | Yes | Yes | No | Yes | 0.125 | No | No | 0.883 | No | Yes | 2.891 | 1.005 | Yes |
| CHEMBL3989553 | CS(=O)(=O)O.CS(=O)(=O)O.N=C(N)Nc1ccc(C(=O)Oc2ccc3cc(C(=N)N)ccc3c2)cc1 | 539.592 | 1.65644 | 4 | 8 | 7 | 207.139 | -2.892 | -1.163 | 0 | -2.735 | Yes | No | No | 0.07 | 0.424 | -2.097 | -3.936 | No | No | No | No | No | No | No | -0.076 | No | No | 0.411 | No | Yes | 2.482 | 3.992 | No |
| CHEMBL4081711 | CC[C@H]1[C@@H](COc2nccc3cc(C(N)=O)c(OC)cc23)NC(=O)[C@H]1F | 361.373 | 1.5838 | 6 | 5 | 2 | 149.217 | -2.734 | 1.042 | 82.065 | -2.797 | Yes | No | No | 0.756 | 0.292 | -0.327 | -3.11 | No | No | No | No | No | No | No | 0.213 | No | No | 0.01 | No | No | 2.133 | 1.979 | Yes |
| CHEMBL1615993 | COc1ccc2[nH]c(-c3cccc(F)c3)cc(=O)c2c1OP(=O)(O)O | 365.253 | 2.8143 | 4 | 4 | 3 | 140.447 | -3.827 | -0.136 | 87.707 | -2.74 | Yes | No | Yes | -0.353 | 0.149 | -1.457 | -3.288 | No | No | Yes | No | No | No | No | 0.154 | No | No | 0.744 | No | No | 2.396 | 2.939 | Yes |
| CHEMBL1477 | COCc1c(C(C)C)nc(C(C)C)c(/C=C/[C@@H](O)C[C@@H](O)CC(=O)O)c1-c1ccc(F)cc1 | 459.558 | 4.8807 | 11 | 5 | 3 | 194.316 | -3.706 | 0.523 | 57.658 | -2.735 | Yes | No | No | -1.13 | 0.053 | -1.531 | -3.197 | No | Yes | No | No | No | No | No | 0.887 | No | No | -1.283 | No | No | 2.684 | 2.731 | No |
| CHEMBL2105721 | CC(C)[C@@]1(C(=O)N[C@H]2CC(=O)O[C@]2(O)CF)CC(c2nccc3ccccc23)=NO1 | 415.421 | 1.8438 | 5 | 7 | 2 | 171.609 | -3.912 | 1.224 | 65.749 | -2.916 | Yes | Yes | No | -0.11 | 0.201 | -0.59 | -3.588 | No | Yes | No | No | No | No | No | 0.895 | No | No | 0.352 | No | No | 2.16 | 2.218 | Yes |
| CHEMBL3334567 | C[C@@H](c1ccc2nccn2c1)n1nnc2ncc(-c3cnn(C)c3)nc21 | 345.37 | 1.8788 | 3 | 9 | 0 | 147.502 | -2.859 | 1.66 | 100 | -2.735 | No | No | No | 0.044 | 0.356 | -1.708 | -3.17 | No | No | Yes | No | No | No | Yes | 0.451 | No | Yes | 0.521 | No | Yes | 2.398 | 0.947 | Yes |
| CHEMBL2107823 | Cc1cc(Nc2cc(CN3CCOCC3)c3nc(C)c(Cc4ccc(Cl)cc4F)n3n2)n[nH]1 | 469.952 | 4.02834 | 6 | 7 | 2 | 195.275 | -2.966 | 0.987 | 92.633 | -2.735 | Yes | Yes | No | 1.126 | 0.246 | -1.658 | -2.606 | No | No | No | No | No | No | Yes | 0.853 | Yes | Yes | 0.581 | No | Yes | 2.409 | 2.511 | Yes |
| CHEMBL4297504 | CO[C@H]1[C@H]([C@@]2(C)O[C@@H]2CC=C(C)C)[C@]2(CC[C@H]1OC(=O)/C=C/c1ccc(OCCN(C)C)cc1)CO2 | 499.648 | 4.2598 | 11 | 7 | 0 | 214.917 | -4.529 | 0.965 | 96.678 | -2.964 | Yes | Yes | Yes | 0.529 | 0.14 | -0.834 | -2.908 | No | Yes | No | No | No | No | Yes | 0.985 | Yes | No | -0.305 | No | Yes | 2.599 | 2.064 | Yes |
| CHEMBL1540 | Nc1nc(O)c2ncn(CCC(CO)CO)c2n1 | 253.262 | -0.895 | 5 | 8 | 4 | 102.738 | -1.56 | 0.076 | 60.633 | -2.735 | Yes | No | No | -0.306 | 0.565 | -1.297 | -3.73 | No | No | No | No | No | No | No | 0.933 | No | No | 0.802 | No | No | 1.4 | 2.493 | Yes |
| CHEMBL1328913 | CN(C)CCCN1c2ccccc2C(C)(C)c2ccccc21 | 294.442 | 4.4157 | 4 | 2 | 0 | 133.922 | -4.929 | 1.401 | 95.542 | -2.465 | Yes | Yes | Yes | 1.81 | 0.052 | 0.941 | -1.384 | Yes | Yes | Yes | No | No | Yes | No | 0.726 | No | No | 0.376 | No | Yes | 2.777 | 0.739 | Yes |
| CHEMBL1201168 | Cc1cc(C(=O)NNCc2ccccc2)no1 | 231.255 | 1.41762 | 4 | 4 | 2 | 98.78 | -2.601 | 0.595 | 93.879 | -3.127 | No | No | No | -0.602 | 0.334 | -0.226 | -2.616 | No | No | Yes | No | No | No | No | 0.663 | No | No | 0.373 | No | No | 2.2 | 1.266 | No |
| CHEMBL4650485 | COc1ccc(F)cc1C(=O)NCc1ccc(-c2nn([C@@H](C)C(F)(F)F)c(N)c2C(N)=O)cc1 | 479.434 | 3.4322 | 7 | 6 | 3 | 191.036 | -4.354 | 0.648 | 78.796 | -2.736 | Yes | Yes | Yes | -0.411 | 0 | -1.486 | -2.793 | No | No | No | Yes | Yes | No | Yes | -0.2 | No | No | 0.768 | No | Yes | 2.322 | 1.366 | Yes |
| CHEMBL59356 | C[C@H](C#Cc1ccc(Cc2ccc(F)cc2)s1)N(O)C(N)=O | 318.373 | 2.9879 | 3 | 3 | 2 | 131.493 | -4.389 | 0.887 | 92.221 | -2.837 | Yes | No | No | -0.128 | 0.022 | -0.043 | -2.257 | No | Yes | Yes | Yes | Yes | No | No | -0.108 | No | Yes | 0.379 | No | Yes | 2.931 | 2.831 | No |
| CHEMBL541388 | Cl.O=S(=O)(c1cccc2cnccc12)N1CCCNCC1 | 327.837 | 1.6406 | 2 | 4 | 1 | 130.375 | -2.911 | 1.298 | 96.844 | -2.838 | Yes | No | No | 0.477 | 0.381 | -0.043 | -2.96 | No | Yes | No | No | No | No | No | 0.684 | No | No | -0.391 | No | No | 2.512 | 1.456 | Yes |
| CHEMBL429910 | CCOc1ccc(Cc2cc([C@@H]3O[C@H](CO)[C@@H](O)[C@H](O)[C@H]3O)ccc2Cl)cc1 | 408.878 | 1.8444 | 6 | 6 | 4 | 168.474 | -4.346 | 0.939 | 51.729 | -2.738 | Yes | Yes | No | -0.832 | 0.096 | -1.224 | -3.632 | No | No | No | No | No | No | No | 0.198 | No | No | 0.035 | No | Yes | 2.872 | 3.44 | No |
| CHEMBL2105015 | Cl.Clc1cccc(C(c2ccc3[nH]cnc3c2)n2ccnc2)c1 | 345.233 | 4.4723 | 3 | 3 | 1 | 144.116 | -2.892 | 1.403 | 78.116 | -2.735 | Yes | Yes | Yes | 0.221 | 0.311 | 0.448 | -0.167 | No | No | Yes | Yes | Yes | Yes | Yes | 1.244 | Yes | Yes | 0.422 | Yes | Yes | 2.482 | 1.373 | No |
| CHEMBL389433 | Clc1cccc(C(c2ccc3nc[nH]c3c2)n2ccnc2)c1 | 308.772 | 4.0505 | 3 | 3 | 1 | 131.963 | -2.892 | 1.377 | 79.088 | -2.735 | Yes | Yes | Yes | 0.186 | 0.316 | 0.341 | -0.18 | No | No | Yes | Yes | Yes | Yes | Yes | 0.9 | Yes | Yes | 0.426 | Yes | Yes | 2.481 | 1.47 | No |
| CHEMBL636 | CCN(C)C(=O)Oc1cccc([C@H](C)N(C)C)c1 | 250.342 | 2.7597 | 4 | 3 | 0 | 109.146 | -2.522 | 1.499 | 89.471 | -2.956 | No | No | No | 0.584 | 0.524 | 0.439 | -2.285 | No | No | No | No | No | Yes | No | 0.551 | No | No | 0.32 | No | No | 3.302 | 1.676 | No |
| CHEMBL294951 | CN1CCC(=C2c3ccccc3CCc3sccc32)CC1 | 295.451 | 4.3742 | 0 | 2 | 0 | 130.472 | -5.072 | 1.479 | 96.704 | -2.362 | Yes | Yes | No | 1.752 | 0.056 | 0.764 | -1.297 | No | Yes | No | No | No | Yes | No | 0.926 | Yes | No | -0.146 | No | Yes | 2.896 | 1.002 | Yes |
| CHEMBL2403238 | C[C@H]1CO[C@@H]2Cn3cc(C(=O)NCc4ccc(F)cc4F)c(=O)c(O)c3C(=O)N12 | 405.357 | 0.9627 | 3 | 6 | 2 | 162.859 | -3.149 | 0.926 | 77.452 | -2.779 | Yes | No | No | -0.098 | 0.212 | -1.077 | -3.153 | No | No | No | No | No | No | No | -0.095 | No | No | 0.562 | No | No | 2.067 | 2.111 | Yes |
| CHEMBL1206232 | COc1ccc(/C=C\c2cc(OC)c(OC)c(OC)c2)cc1OP(=O)(O)O | 396.332 | 3.3629 | 8 | 6 | 2 | 156.694 | -4.199 | 0.286 | 83.609 | -2.909 | Yes | Yes | No | -1.136 | 0.174 | -1.349 | -2.946 | No | Yes | Yes | Yes | Yes | No | Yes | 0.354 | No | Yes | 0.973 | No | No | 2.149 | 1.33 | No |
| CHEMBL1380 | Nc1nc(NC2CC2)c2ncn([C@H]3C=C[C@@H](CO)C3)c2n1 | 286.339 | 1.0923 | 4 | 7 | 3 | 121.458 | -1.543 | 0.124 | 83.46 | -2.735 | Yes | No | No | 0.094 | 0.645 | -1.008 | -3.213 | No | No | No | No | No | No | No | 1.165 | No | No | 0.194 | No | No | 2.201 | 1.673 | Yes |
| CHEMBL3301625 | C=CC(=O)Nc1cccc(Nc2nc(Nc3ccc(OCCOC)cc3)ncc2F)c1 | 423.448 | 4.2526 | 10 | 7 | 3 | 178.689 | -4.376 | 0.652 | 91.74 | -2.75 | Yes | Yes | Yes | 0.326 | 0.03 | -1.521 | -3.366 | No | Yes | No | Yes | Yes | No | Yes | -0.398 | No | No | 0.286 | No | Yes | 2.635 | 1.86 | Yes |
| CHEMBL273575 | CN1Cc2c(N)cccc2C(c2ccccc2)C1 | 238.334 | 2.8461 | 1 | 2 | 1 | 108.042 | -3.216 | 1.296 | 94.55 | -2.78 | Yes | No | No | 1.099 | 0.21 | 0.429 | -0.631 | Yes | Yes | Yes | No | No | Yes | No | 0.972 | Yes | Yes | 0.218 | No | Yes | 2.778 | 2.477 | Yes |
| CHEMBL4112930 | C[C@]12CCCN1CC1=NNC(=O)c3cc(F)cc4[nH]c2c1c34 | 298.321 | 2.3681 | 0 | 3 | 2 | 124.677 | -3.043 | 0.859 | 97.307 | -2.737 | No | No | No | 1.136 | 0.204 | 0.314 | -3.35 | Yes | Yes | Yes | No | No | No | No | 1.223 | No | Yes | -0.062 | No | Yes | 2.456 | 0.752 | Yes |
| CHEMBL1230165 | O=C(O)c1ccc2c(c1)nc(Nc1cccc(Cl)c1)c1ccncc12 | 349.777 | 4.8782 | 3 | 4 | 2 | 147.465 | -3.935 | 0.712 | 92.788 | -2.723 | Yes | No | No | -0.157 | 0.139 | -0.173 | -1.845 | No | Yes | Yes | No | No | No | No | 0.1 | No | No | 0.641 | No | No | 2.975 | 0.642 | Yes |
| CHEMBL629 | CN(C)CCC=C1c2ccccc2CCc2ccccc21 | 277.411 | 4.1686 | 3 | 1 | 0 | 127.472 | -4.751 | 1.47 | 98.51 | -2.57 | Yes | Yes | No | 1.706 | 0.046 | 0.972 | -1.21 | No | Yes | No | No | No | Yes | No | 0.827 | No | No | 0.312 | No | Yes | 2.708 | 1.18 | Yes |
| CHEMBL497 | Oc1c(I)cc(Cl)c2cccnc12 | 305.502 | 3.1984 | 0 | 2 | 1 | 93.692 | -3.129 | 1.387 | 93.122 | -2.103 | No | No | No | -0.03 | 0.372 | 0.332 | -2.301 | No | No | Yes | No | No | No | No | -0.019 | No | No | 0.496 | No | No | 2.254 | 1.834 | No |
| CHEMBL2017974 | Nc1cc(C(F)(F)F)c(-c2cc(N3CCOCC3)nc(N3CCOCC3)n2)cn1 | 410.4 | 1.8128 | 3 | 8 | 1 | 165.004 | -4.897 | 1.091 | 97.441 | -2.926 | No | No | No | -0.746 | 0.384 | -1.375 | -3.126 | No | Yes | No | No | No | No | No | 0.109 | No | No | -0.378 | No | No | 2.583 | 1.095 | Yes |
| CHEMBL859 | O=c1[nH]c(=O)c2c[nH]nc2[nH]1 | 152.113 | -1.0605 | 0 | 3 | 3 | 59.273 | -2.17 | -0.205 | 67.922 | -2.745 | No | No | No | -0.128 | 0.73 | -1.116 | -4.635 | No | No | No | No | No | No | No | 0.7 | No | No | 0.383 | No | No | 2.151 | 1.545 | No |
| CHEMBL3545366 | CCn1c(=O)c(-c2cc[nH]n2)cc2c(C)nc(N)nc21 | 270.296 | 1.09212 | 2 | 6 | 2 | 114.033 | -3.035 | 0.28 | 83.082 | -2.742 | Yes | No | No | 0.365 | 0.45 | -1.214 | -3.5 | No | No | Yes | No | No | No | No | 0.522 | No | No | 0.287 | No | No | 2.708 | 0.091 | Yes |
| CHEMBL972 | C#CCN(C)[C@H](C)Cc1ccccc1 | 187.286 | 2.1826 | 4 | 1 | 0 | 86.745 | -2.29 | 1.545 | 94.876 | -1.88 | No | No | No | 1.1 | 0.387 | 0.826 | -1.53 | Yes | No | Yes | No | No | Yes | No | 1.045 | Yes | No | 0.463 | No | No | 2.499 | 1.443 | Yes |
| CHEMBL2103879 | CC(C)c1cc(-c2n[nH]c(=O)n2-c2ccc3c(ccn3C)c2)c(O)cc1O | 364.405 | 3.2539 | 3 | 6 | 3 | 154.683 | -2.96 | 0.369 | 75.801 | -2.735 | Yes | Yes | Yes | -0.526 | 0.044 | -1.722 | -2.453 | Yes | No | Yes | Yes | No | No | No | 0.966 | No | No | 0.744 | No | Yes | 2.686 | 3.133 | Yes |
| CHEMBL1650443 | Cn1c(=O)cc(N2CCC[C@@H](N)C2)n(Cc2cc(F)ccc2C#N)c1=O | 357.389 | 0.53358 | 3 | 7 | 1 | 149.242 | -3.136 | 0.335 | 82.527 | -3.126 | Yes | No | No | 0.296 | 0.376 | -0.787 | -2.952 | No | Yes | No | No | No | No | No | 0.299 | No | Yes | -0.515 | No | No | 2.379 | 1.518 | Yes |
| CHEMBL419213 | NCCCC[C@H](N[C@@H](CCc1ccccc1)C(=O)O)C(=O)N1CCC[C@H]1C(=O)O.O.O | 441.525 | -0.4142 | 12 | 5 | 4 | 181.584 | -2.759 | -0.525 | 3.751 | -2.735 | Yes | No | No | -1.832 | 0.634 | -1.004 | -4.167 | No | No | No | No | No | No | No | 0.83 | No | No | 0.441 | No | No | 2.077 | 2.976 | Yes |
| CHEMBL3989937 | CNC(=O)c1ccc(-c2cnc3ncc(Cc4ccc5ncccc5c4)n3n2)cc1F.Cl.Cl.O | 503.365 | 3.4479 | 4 | 6 | 1 | 205.951 | -2.902 | 0.743 | 96.737 | -2.735 | Yes | Yes | Yes | -0.357 | 0.282 | -1.66 | -3.442 | No | Yes | Yes | Yes | Yes | Yes | Yes | 0.978 | Yes | No | 0.338 | No | Yes | 2.49 | 0.417 | Yes |
| CHEMBL1078685 | N#Cc1cc(-c2n[nH]c(-c3ccncc3)n2)ccn1 | 248.249 | 1.80038 | 2 | 5 | 1 | 108.359 | -3.227 | 1.034 | 98.776 | -2.745 | No | No | No | 0.615 | 0.37 | -1.233 | -3.403 | No | No | Yes | No | No | No | No | 0.768 | No | Yes | 0.475 | No | No | 2.472 | 0.128 | Yes |
| CHEMBL1170047 | NC(=O)c1ccc(I)c([N+](=O)[O-])c1 | 292.032 | 1.2983 | 2 | 3 | 1 | 87.213 | -2.963 | 0.206 | 71.329 | -2.637 | No | No | No | -0.046 | 0.281 | -0.475 | -2.439 | No | No | Yes | No | No | No | No | -0.169 | No | Yes | 0.848 | No | No | 1.982 | 1.628 | No |
| CHEMBL4297522 | CCN1C(=O)N(c2c(F)c(OC)cc(OC)c2F)Cc2cnc3[nH]c(CN4CCOCC4)cc3c21 | 487.507 | 3.657 | 6 | 6 | 1 | 200.714 | -3.645 | 1.032 | 96.286 | -2.735 | Yes | Yes | No | 0.772 | 0.166 | -1.787 | -3.695 | No | Yes | No | No | No | No | Yes | 0.821 | No | No | -0.391 | No | Yes | 2.926 | 2.311 | Yes |
| CHEMBL2105659 | CCc1c(C(=O)C(N)=O)c2c(OCC(=O)OC)cccc2n1Cc1ccccc1 | 394.427 | 2.4718 | 8 | 6 | 1 | 167.56 | -5.277 | 1.201 | 83.927 | -2.79 | No | Yes | Yes | -0.312 | 0.052 | -0.528 | -2.938 | No | Yes | No | No | Yes | No | Yes | 0.547 | No | No | 0.463 | No | Yes | 2.206 | 1.561 | Yes |
| CHEMBL4297629 | COc1ccc([C@@H](O)c2cc(-c3ncnc4cc(N5CCOCC5)ccc34)c(F)cc2Cl)nn1 | 481.915 | 3.8062 | 5 | 8 | 1 | 199.508 | -3.62 | 0.955 | 99.801 | -2.735 | Yes | Yes | Yes | -0.19 | 0.298 | -1.101 | -3.624 | No | Yes | No | No | No | No | Yes | 0.602 | No | No | 0.727 | No | Yes | 2.738 | 0.67 | Yes |
| CHEMBL431 | CCOC(=O)[C@H](CCc1ccccc1)N[C@@H](C)C(=O)N1CC2(C[C@H]1C(=O)O)SCCS2 | 466.625 | 2.3908 | 9 | 7 | 2 | 191.207 | -2.962 | -0.025 | 48.514 | -2.735 | Yes | No | No | -0.56 | 0.547 | -0.787 | -2.904 | No | Yes | No | No | No | No | No | 0.595 | No | No | -0.018 | No | No | 2.217 | 2.092 | Yes |
| CHEMBL788 | O=c1[nH]c(=O)n([C@H]2C[C@H](O)[C@@H](CO)O2)cc1I | 354.1 | -1.2181 | 2 | 6 | 3 | 109.186 | -2.905 | 0.044 | 58.632 | -3.201 | No | No | No | -0.462 | 0.728 | -1.179 | -3.639 | No | No | No | No | No | No | No | -0.017 | No | No | 0.96 | No | No | 2.161 | 2.465 | No |
| CHEMBL338975 | CNC(=O)Oc1ccc2c(c1)[C@]1(C)CCN(C)[C@@H]1N2C.O=C(O)c1ccccc1O | 413.474 | 2.8643 | 2 | 6 | 3 | 175.227 | -3.564 | 1.033 | 59.891 | -2.735 | Yes | No | No | -0.191 | 0.167 | -0.992 | -2.343 | No | Yes | No | No | No | No | No | 0.189 | No | No | -0.264 | No | No | 2.36 | 1.123 | Yes |
| CHEMBL3260567 | Cc1cccc(-c2[nH]c(CNc3ccccc3F)nc2-c2ccc3ncnn3c2)n1 | 399.433 | 4.24102 | 5 | 6 | 2 | 171.222 | -2.894 | 0.978 | 92.181 | -2.735 | Yes | Yes | Yes | -0.059 | 0.187 | -1.671 | -2.155 | No | No | Yes | No | No | No | Yes | 0.798 | Yes | Yes | 0.359 | No | Yes | 2.537 | -0.254 | Yes |
| CHEMBL3693786 | O=C(CCCCCCNC(=O)c1cnc(N(c2ccccc2)c2ccccc2Cl)nc1)NO | 467.957 | 4.7855 | 11 | 6 | 3 | 197.139 | -4.85 | -0.167 | 86.001 | -2.735 | Yes | Yes | Yes | 0.044 | 0.018 | -1.359 | -2.822 | No | Yes | No | Yes | Yes | No | Yes | 0.032 | No | Yes | 0.08 | No | Yes | 3.369 | 2.554 | Yes |
| CHEMBL142703 | N#C[C@@H]1CCCN1C(=O)CNC12CC3CC(CC(O)(C3)C1)C2 | 303.406 | 1.17428 | 3 | 4 | 2 | 131.213 | -3.337 | 0.593 | 69.814 | -3.201 | No | No | No | 0.552 | 0.575 | -0.507 | -3.14 | No | No | No | No | No | No | No | 1.006 | No | No | 0.203 | No | No | 2.841 | 0.605 | No |
| CHEMBL1743259 | CCc1nn(CCCN2CCN(c3cccc(Cl)c3)CC2)c(=O)n1CC | 377.92 | 2.4928 | 7 | 6 | 0 | 159.518 | -3.825 | 1.11 | 95.507 | -2.885 | Yes | No | No | 0.819 | 0.291 | 0.258 | -1.901 | No | Yes | No | No | No | Yes | No | 0.8 | Yes | No | -0.292 | No | Yes | 2.951 | 1.482 | Yes |
| CHEMBL504535 | CCCN1CC(c2ccc(S(C)(=O)=O)cc2)=C(c2ccc(C)cc2)C1=O | 369.486 | 3.56152 | 5 | 3 | 0 | 154.624 | -4.936 | 1.019 | 96.374 | -2.725 | Yes | Yes | Yes | 0.37 | 0 | -0.027 | -2.117 | No | Yes | Yes | Yes | Yes | No | Yes | 0.862 | No | No | -0.106 | No | Yes | 2.851 | 1.418 | Yes |
| CHEMBL1201057 | CC1(C)C[C@@H]1C(=O)N/C(=C\CCCCSC[C@H](N)C(=O)O)C(=O)[O-].[Na+] | 380.442 | -2.8981 | 11 | 6 | 3 | 174.017 | -2.243 | -0.03 | 0 | -2.735 | No | No | No | -1.208 | 0.681 | -0.624 | -4.474 | No | Yes | No | No | No | No | No | 0.853 | No | No | -0.017 | No | No | 2.094 | 2.321 | Yes |
| CHEMBL2107355 | Cl.OC[C@H]1NC[C@H](O)[C@@H](O)[C@H]1O | 199.634 | -2.545 | 1 | 5 | 5 | 76.438 | -0.292 | -0.314 | 43.99 | -3.311 | No | No | No | -0.716 | 0.906 | -1.288 | -5.13 | No | No | No | No | No | No | No | 1.38 | No | No | 2.263 | No | No | 1.849 | 2.351 | No |
| CHEMBL1232767 | F | 20.006 | 0.1525 | 0 | 0 | 0 | 6.097 | 0.093 | 1.382 | 100 | -2.589 | Yes | No | No | -0.069 | 0.789 | 0.05 | -2.298 | No | No | Yes | No | No | No | No | 0.291 | No | No | 1.225 | No | No | 2.414 | 1.71 | No |
| CHEMBL880 | CC(=O)OCC(CCn1cnc2cnc(N)nc21)COC(C)=O | 321.337 | 0.541 | 7 | 9 | 1 | 132.366 | -2.41 | 0.12 | 76.999 | -2.737 | Yes | No | No | -0.234 | 0.64 | -1.473 | -3.254 | No | No | No | No | No | No | No | 1.026 | No | Yes | 0.44 | No | No | 2.268 | 0.761 | Yes |
| CHEMBL637 | COc1ccc(C(CN(C)C)C2(O)CCCCC2)cc1 | 277.408 | 3.0356 | 5 | 3 | 1 | 122.108 | -2.624 | 1.313 | 92.32 | -3.055 | Yes | No | No | 0.832 | 0.449 | 0.137 | -2.651 | Yes | Yes | No | No | No | Yes | No | 0.835 | No | No | -0.056 | No | Yes | 2.95 | 1.374 | Yes |
| CHEMBL27759 | Nc1ccccc1NC(=O)c1ccc(CNC(=O)OCc2cccnc2)cc1 | 376.416 | 3.3425 | 6 | 5 | 3 | 162.101 | -3.763 | 0.853 | 85.001 | -2.775 | Yes | Yes | Yes | -0.055 | 0 | -1.019 | -2.529 | No | Yes | No | Yes | Yes | No | Yes | 0.171 | No | No | -0.026 | No | Yes | 2.34 | 1.551 | Yes |
| CHEMBL17 | NS(=O)(=O)c1cc(Cl)c(Cl)c(S(N)(=O)=O)c1 | 305.164 | 0.2882 | 2 | 4 | 2 | 102.239 | -3.169 | 0.153 | 79.961 | -2.898 | No | No | No | -0.784 | 0.457 | -1.21 | -3.205 | No | No | No | No | No | No | No | 0.041 | No | No | 1.088 | No | No | 2.756 | 1.947 | No |
| CHEMBL4297674 | CC#CC(=O)N[C@H]1CCCN(c2c(F)cc(C(N)=O)c3[nH]c(C)c(C)c23)C1 | 370.428 | 2.13104 | 3 | 3 | 3 | 156.833 | -3.93 | 1.047 | 94.02 | -3.566 | Yes | No | No | 0.024 | 0.261 | -0.626 | -3.37 | No | No | No | No | No | No | Yes | 0.495 | No | No | 0.054 | No | Yes | 2.225 | 0.858 | Yes |
| CHEMBL520733 | CC(C)[C@H](O)C(=O)N[C@@H](C)C(=O)N[C@@H]1C(=O)N(C)CCc2ccccc21 | 361.442 | 0.38 | 5 | 4 | 3 | 153.308 | -2.632 | 0.38 | 57.116 | -3.032 | Yes | No | No | -0.546 | 0.334 | -0.449 | -3.127 | No | No | No | No | No | No | No | 1.413 | No | No | 0.544 | No | No | 1.712 | 2.558 | No |
| CHEMBL2180602 | CCN(CC)CCNC(=O)c1c(C)[nH]c2c1CCC/C2=C1/C(=O)Nc2ccc(F)cc21 | 424.52 | 3.73302 | 6 | 3 | 3 | 181.454 | -4.444 | 0.932 | 89.275 | -2.804 | Yes | Yes | Yes | 0.905 | 0.065 | -0.791 | -2.352 | Yes | Yes | Yes | No | No | No | Yes | 1.055 | No | Yes | -0.439 | No | Yes | 2.939 | 0.936 | Yes |
| CHEMBL3545063 | C=CC(=O)Nc1cc(Nc2nccc(-c3cn(C)c4ccccc34)n2)c(OC)cc1N(C)CCN(C)C.CS(=O)(=O)O | 595.726 | 4.0138 | 10 | 10 | 3 | 245.939 | -3.479 | -0.313 | 46.606 | -2.735 | Yes | No | Yes | 0.592 | 0.2 | -1.746 | -3.53 | No | Yes | No | No | No | No | No | 0.381 | No | No | 0.676 | No | No | 2.536 | 1.984 | Yes |
| CHEMBL1201174 | N[C@@H](CC(=O)N1CCn2c(nnc2C(F)(F)F)C1)Cc1cc(F)c(F)cc1F.O.O=P(O)(O)O | 523.327 | 0.2632 | 4 | 6 | 4 | 186.902 | -3.473 | 0.244 | 45.588 | -2.741 | Yes | No | No | -1.586 | 0.217 | -2.524 | -4.622 | No | Yes | No | No | No | No | No | 0.193 | No | No | 0.363 | No | No | 2.639 | 2.865 | Yes |
| CHEMBL1422 | N[C@@H](CC(=O)N1CCn2c(nnc2C(F)(F)F)C1)Cc1cc(F)c(F)cc1F | 407.318 | 2.0165 | 4 | 5 | 1 | 154.484 | -4.059 | 1.304 | 90.666 | -2.964 | No | No | No | -0.305 | 0.226 | -1.479 | -3.106 | No | Yes | No | No | No | No | No | 0.489 | No | No | 0.372 | No | No | 2.715 | 1.167 | Yes |
| CHEMBL3112741 | O=C(NC1CCNCC1)[C@@H]1CC[C@@H]2CN1C(=O)N2OS(=O)(=O)O | 348.381 | -1.1424 | 4 | 6 | 3 | 133.584 | -2.265 | -0.058 | 14.587 | -2.735 | No | No | No | -0.485 | 0.826 | -1.257 | -3.418 | No | No | No | No | No | No | No | 0.662 | No | No | 1.097 | No | No | 1.523 | 2.497 | Yes |
| CHEMBL189963 | CC(=O)c1c(C)c2cnc(Nc3ccc(N4CCNCC4)cn3)nc2n(C2CCCC2)c1=O | 447.543 | 2.96582 | 5 | 9 | 2 | 191.809 | -3.594 | 1.222 | 89.954 | -2.854 | Yes | Yes | No | 0.582 | 0.319 | -1.131 | -3.122 | No | Yes | No | No | No | No | No | 0.742 | No | No | -0.684 | No | Yes | 2.683 | 1.318 | Yes |
| CHEMBL1444 | N#Cc1ccc(C(c2ccc(C#N)cc2)n2cncn2)cc1 | 285.31 | 2.65916 | 3 | 5 | 0 | 127.252 | -3.78 | 1.077 | 100 | -2.472 | No | No | No | 0.081 | 0.238 | -0.402 | -2.07 | No | Yes | Yes | Yes | No | No | Yes | 0.786 | Yes | No | -0.545 | No | No | 2.176 | 1.26 | No |
| CHEMBL2364614 | CN1Cc2cc(-c3ccc(N)nn3)ccc2[C@H](c2ccc3ccccc3c2)C1 | 366.468 | 4.4563 | 2 | 4 | 1 | 164.189 | -5.366 | 0.976 | 94.477 | -2.753 | Yes | Yes | Yes | 0.118 | 0.144 | 0.299 | -1.481 | No | Yes | Yes | No | No | Yes | Yes | 0.661 | No | Yes | 0.513 | No | Yes | 2.456 | 0.983 | Yes |
| CHEMBL522038 | CCOC(=O)CN[C@@H](C(=O)N1CC[C@H]1C(=O)NCc1ccc(/C(N)=N\O)cc1)C1CCCCC1 | 473.574 | 1.0998 | 10 | 7 | 4 | 199.43 | -3.445 | 0.137 | 57.251 | -2.733 | Yes | Yes | No | -0.05 | 0.329 | -0.913 | -3.382 | No | Yes | No | No | No | No | No | 0.677 | No | No | -0.084 | No | Yes | 2.671 | 1.385 | Yes |
| CHEMBL578 | CCOC(=O)[C@H](CCc1ccccc1)N[C@@H](C)C(=O)N1CCC[C@H]1C(=O)O | 376.453 | 1.6046 | 9 | 5 | 2 | 159.237 | -2.647 | 0.334 | 43.99 | -2.735 | Yes | No | No | -0.255 | 0.753 | -0.698 | -3.22 | No | No | No | No | No | No | No | 0.804 | No | No | 0.32 | No | No | 2.076 | 2.099 | Yes |
| CHEMBL2364628 | O=C(CCCCCCNC(=O)c1cnc(N(c2ccccc2)c2ccccc2)nc1)NO | 433.512 | 4.1321 | 11 | 6 | 3 | 186.835 | -4.631 | -0.066 | 84.893 | -2.735 | Yes | Yes | Yes | 0.045 | 0.016 | -1.185 | -2.936 | No | Yes | No | Yes | Yes | No | Yes | 0.263 | No | Yes | 0.09 | No | Yes | 3.357 | 2.359 | Yes |
| CHEMBL2325741 | NC1(C(=O)N[C@@H](CCO)c2ccc(Cl)cc2)CCN(c2ncnc3[nH]ccc23)CC1 | 428.924 | 2.1489 | 6 | 6 | 4 | 179.006 | -3.061 | 1.023 | 71.801 | -2.818 | Yes | No | No | 0.553 | 0.2 | -1.036 | -3.589 | No | No | No | No | No | No | No | 0.942 | No | No | -0.118 | No | Yes | 2.469 | 1.276 | Yes |
| CHEMBL4208229 | O=C(Nc1ccc(OC(F)(F)Cl)cc1)c1cnc(N2CC[C@@H](O)C2)c(-c2cc[nH]n2)c1 | 449.845 | 3.4629 | 6 | 6 | 3 | 180.36 | -3.78 | 0.587 | 89.114 | -2.755 | Yes | Yes | Yes | -0.06 | 0.126 | -1.66 | -2.869 | No | Yes | No | Yes | Yes | No | Yes | 0.163 | No | Yes | 0.035 | No | Yes | 2.904 | 1.805 | Yes |
| CHEMBL2105671 | O=C(O)C(O)C(O)C(=O)O.OC[C@H]1CNC[C@@H](O)[C@@H]1O | 297.26 | -4.2027 | 4 | 8 | 8 | 113.456 | -2.709 | -1.199 | 0 | -2.735 | No | No | No | -1.031 | 0.538 | -2.209 | -6.502 | No | No | No | No | No | No | No | 0.515 | No | No | 0.924 | No | No | 1.814 | 5.115 | No |
| CHEMBL4297587 | Cc1[nH]c(/C=C2\C(=O)Nc3ccc(F)cc32)c(C)c1C(=O)N[C@H]1CCN(C(=O)N(C)C)C1 | 439.491 | 2.74904 | 3 | 3 | 3 | 185.01 | -3.999 | 0.712 | 79.372 | -2.934 | Yes | Yes | No | 0.017 | 0.067 | -0.988 | -2.436 | No | Yes | No | Yes | No | No | No | 0.39 | No | No | -0.319 | No | Yes | 1.922 | 3.091 | Yes |
| CHEMBL514800 | CCOc1cc([C@@H](CS(C)(=O)=O)N2C(=O)c3cccc(NC(C)=O)c3C2=O)ccc1OC | 460.508 | 2.4342 | 8 | 7 | 1 | 185.779 | -4.479 | 0.362 | 99.422 | -2.775 | Yes | Yes | Yes | -0.532 | 0.027 | -0.536 | -3.574 | No | Yes | No | No | Yes | No | No | 0.229 | Yes | No | 0.246 | No | No | 2.434 | 1.497 | Yes |
| CHEMBL1200759 | Cl.O=C1CN2Cc3c(ccc(Cl)c3Cl)N=C2N1 | 292.553 | 2.3481 | 0 | 3 | 1 | 114.006 | -2.829 | 1.299 | 84.264 | -2.74 | No | No | No | 0.209 | 0.484 | 0.209 | -1.973 | No | Yes | Yes | No | No | No | No | 1.328 | Yes | No | 0.332 | No | No | 3.3 | 1.447 | No |
| CHEMBL1083385 | COC(=O)[C@H](c1ccccc1Cl)N1CCc2sccc2C1.O=S(=O)(O)O | 419.908 | 3.0211 | 3 | 6 | 2 | 160.289 | -3.063 | 0.925 | 43.851 | -2.735 | Yes | No | No | -1.136 | 0.428 | -1.302 | -3.232 | No | No | No | No | No | No | No | 0.72 | No | No | 1.068 | No | No | 2.424 | 2.102 | Yes |
| CHEMBL252164 | CCNC(=O)c1noc(-c2cc(C(C)C)c(O)cc2O)c1-c1ccc(CN2CCOCC2)cc1 | 465.55 | 4.1251 | 7 | 7 | 3 | 198.693 | -4.326 | 0.774 | 84.697 | -2.735 | Yes | Yes | Yes | 0.075 | 0.137 | -1.203 | -2.636 | No | Yes | No | Yes | No | Yes | Yes | 0.661 | No | No | 0.249 | No | Yes | 2.373 | 1.336 | Yes |
| CHEMBL334830 | O=C1C[C@@]2(C(=O)N1)C(=O)N(Cc1ccc(Br)cc1F)C(=O)c1cccn12 | 420.194 | 1.3141 | 2 | 5 | 1 | 154.439 | -3.322 | 1.334 | 72.866 | -3.005 | No | No | No | -0.661 | 0.178 | -0.375 | -3.129 | No | Yes | No | No | No | No | No | -0.122 | No | No | 0.084 | No | No | 2.207 | 1.488 | Yes |
| CHEMBL1201142 | C#CCN[C@@H]1CCc2ccccc21.CS(=O)(=O)O | 267.35 | 1.4007 | 2 | 3 | 2 | 108.164 | -1.367 | 0.667 | 90.848 | -2.735 | No | No | No | -0.005 | 0.539 | -0.085 | -2.401 | No | No | No | No | No | No | No | 1.114 | No | No | 0.543 | No | No | 2.123 | 2.479 | No |
| CHEMBL3137301 | CCOC(=O)[C@H](C)C[C@@H](Cc1ccc(-c2ccccc2)cc1)NC(=O)CCC(=O)O | 411.498 | 3.835 | 11 | 4 | 2 | 176.81 | -4.219 | -0.363 | 58.502 | -2.735 | Yes | No | No | -0.761 | 0.01 | -0.601 | -2.716 | No | Yes | No | No | No | No | No | 1.242 | No | No | 0.43 | No | No | 2.837 | 1.992 | Yes |
| CHEMBL3286830 | C[C@H]1Oc2cc(cnc2N)-c2c(nn(C)c2C#N)CN(C)C(=O)c2ccc(F)cc21 | 406.421 | 2.80068 | 0 | 7 | 1 | 171.854 | -3.437 | 1.56 | 89.113 | -2.736 | Yes | No | No | 0.374 | 0.115 | -0.102 | -2.933 | No | No | No | No | No | No | Yes | 0.518 | No | No | 0.347 | No | Yes | 2.214 | 1.094 | Yes |
| CHEMBL4802155 | Nc1nnc(CN[C@@H]2C[C@H]2c2ccc(OCc3ccccc3)cc2)o1 | 336.395 | 2.8765 | 7 | 6 | 2 | 145.57 | -3.26 | 0.894 | 91.818 | -2.678 | Yes | Yes | Yes | 0.398 | 0.041 | -0.42 | -2.598 | No | Yes | Yes | Yes | No | No | Yes | 1.084 | Yes | No | 0.2 | No | Yes | 2.55 | 1.32 | Yes |
| CHEMBL1561 | OCCN1C[C@H](O)[C@@H](O)[C@H](O)[C@H]1CO | 207.226 | -3.2621 | 3 | 6 | 5 | 82.019 | -0.599 | -0.458 | 29.58 | -3.409 | No | No | No | -0.509 | 0.928 | -1.468 | -4.842 | No | No | No | No | No | No | No | 0.822 | No | Yes | 2.173 | No | No | 1.755 | 3.794 | No |
| CHEMBL1257015 | COc1ccc(C(=O)NCc2cccnc2)cc1C(=O)NCc1cccnc1 | 376.416 | 2.3452 | 7 | 5 | 2 | 162.346 | -3.661 | 1.223 | 89.244 | -2.808 | Yes | Yes | No | -0.373 | 0.047 | -0.613 | -3.012 | No | Yes | Yes | No | No | No | Yes | 0.553 | No | No | 0.09 | No | Yes | 2.546 | 0.905 | Yes |
| CHEMBL52939 | COc1cccc(CCc2ccccc2OCC(CN(C)C)OC(=O)CCC(=O)O)c1 | 429.513 | 3.1974 | 13 | 6 | 1 | 183.086 | -4.654 | 1.054 | 94.781 | -2.735 | Yes | No | Yes | -0.141 | 0.067 | -0.888 | -3.151 | No | Yes | No | No | No | No | No | 0.798 | No | No | 0.481 | No | No | 2.37 | 2.142 | Yes |
| CHEMBL41 | CNCCC(Oc1ccc(C(F)(F)F)cc1)c1ccccc1 | 309.331 | 4.435 | 6 | 2 | 1 | 127.473 | -4.823 | 1.093 | 92.14 | -2.583 | No | Yes | No | 1.055 | 0.047 | 0.508 | -1.441 | No | Yes | Yes | Yes | No | Yes | Yes | 0.699 | No | Yes | 0.659 | No | Yes | 2.844 | 1.664 | Yes |
| CHEMBL498847 | Cc1ncc([N+](=O)[O-])n1CC(C)O | 185.183 | 0.48052 | 3 | 5 | 1 | 74.977 | -1.54 | 0.613 | 92.649 | -2.834 | Yes | No | No | -0.216 | 0.644 | -0.717 | -3.002 | No | No | No | No | No | No | No | 0.417 | No | Yes | 0.22 | No | No | 1.966 | 1.461 | No |
| CHEMBL2018096 | OC[C@H]1O[C@@H](c2ccc(F)c(Cc3cc4ccccc4s3)c2)[C@H](O)[C@@H](O)[C@@H]1O | 404.459 | 2.1461 | 4 | 6 | 4 | 164.815 | -3.66 | 1.055 | 93.499 | -2.74 | Yes | Yes | Yes | -1.015 | 0.008 | -0.999 | -3.444 | No | Yes | Yes | No | No | No | No | 0.116 | No | No | 0.128 | No | Yes | 2.194 | 3.484 | No |
| CHEMBL160038 | O=C(Cc1ccccc1)c1cc(O)c(O)c([N+](=O)[O-])c1 | 273.244 | 2.4314 | 4 | 5 | 2 | 113.621 | -3.151 | 0.051 | 83.644 | -2.761 | Yes | No | No | -0.433 | 0.082 | -0.376 | -2.406 | No | No | Yes | No | Yes | No | No | 0.264 | No | No | 0.149 | No | No | 2.783 | 3.158 | No |
| CHEMBL4071161 | CC#CC(=O)N1CC[C@@H](n2c(=O)n(-c3ccc(Oc4ccccc4)cc3)c3c(N)ncnc32)C1 | 454.49 | 2.7534 | 4 | 8 | 1 | 194.965 | -2.996 | 0.997 | 90.501 | -2.735 | Yes | Yes | Yes | 0.28 | 0.225 | -0.985 | -2.73 | No | Yes | No | Yes | Yes | No | Yes | 0.229 | Yes | Yes | 0.02 | No | Yes | 2.526 | 1.622 | Yes |
| CHEMBL1200814 | CC(=O)Nc1nnc(S([NH-])(=O)=O)s1.[Na+] | 244.233 | -2.7586 | 2 | 6 | 1 | 106.435 | -2.545 | 0.145 | 48.986 | -2.836 | No | No | No | -0.857 | 0.63 | -1.222 | -3.287 | No | No | No | No | No | No | No | -0.702 | No | No | 1.325 | No | No | 2.631 | 0.682 | Yes |
| CHEMBL1201773 | CC(=O)Oc1cc2c(s1)CCN(C(C(=O)C1CC1)c1ccccc1F)C2.Cl | 409.91 | 4.3128 | 5 | 5 | 0 | 167.282 | -3.865 | 1.364 | 90.224 | -2.937 | Yes | Yes | Yes | 0.197 | 0.117 | 0.39 | -1.445 | No | Yes | No | No | No | Yes | Yes | 1.438 | Yes | No | 0.109 | No | Yes | 3.203 | 1.662 | Yes |
| CHEMBL237500 | CC#CCn1c(N2CCC[C@@H](N)C2)nc2c1c(=O)n(Cc1nc(C)c3ccccc3n1)c(=O)n2C | 472.553 | 1.14742 | 4 | 10 | 1 | 201.585 | -2.922 | 0.273 | 91.112 | -2.735 | No | Yes | No | 0.66 | 0.199 | -1.542 | -2.941 | No | Yes | No | No | No | No | Yes | 0.379 | No | No | 0.345 | No | Yes | 2.515 | 1.257 | Yes |
| CHEMBL1213492 | CCN(CC)Cc1ccc2cc(COC(=O)Nc3ccc(C(=O)NO)cc3)ccc2c1 | 421.497 | 4.5493 | 8 | 5 | 3 | 181.134 | -5.314 | 1.094 | 84.952 | -2.739 | Yes | Yes | Yes | 0.253 | 0 | -0.98 | -2.388 | No | Yes | No | Yes | Yes | No | Yes | 0.87 | No | Yes | 0.281 | No | Yes | 2.631 | 2.755 | Yes |
| CHEMBL1229211 | C[C@@H]1CCO[C@H]2Cn3cc(C(=O)NCc4ccc(F)cc4F)c(=O)c(O)c3C(=O)N21 | 419.384 | 1.3528 | 3 | 6 | 2 | 169.224 | -3.281 | 0.934 | 75.313 | -2.786 | Yes | No | No | -0.072 | 0.191 | -1.1 | -3.134 | No | No | No | No | No | No | No | -0.044 | No | No | 0.495 | No | No | 2.14 | 2.09 | Yes |
| CHEMBL121810 | CCc1ccccc1NC(=O)Oc1ccc2c(c1)[C@]1(C)CCN(C)O[C@@H]1N2C | 381.476 | 4.1606 | 3 | 5 | 1 | 165.538 | -4.585 | 1.016 | 91.468 | -2.881 | No | Yes | Yes | 0.263 | 0 | 0.285 | -1.809 | No | Yes | Yes | Yes | Yes | No | Yes | 0.273 | No | No | -0.505 | No | Yes | 2.577 | 1.292 | Yes |
| CHEMBL1201753 | O=C(O)C[C@H](O)C[C@H](O)/C=C/c1c(C2CC2)nc2ccccc2c1-c1ccc(F)cc1 | 421.468 | 4.5181 | 8 | 4 | 3 | 179.054 | -4.461 | 0.883 | 98.585 | -2.735 | Yes | No | Yes | -0.989 | 0.208 | -1.335 | -3.181 | No | Yes | No | No | No | No | No | 1.019 | No | No | 0.79 | No | Yes | 3.38 | 0.744 | Yes |
| CHEMBL403 | CC1(C)[C@H](C(=O)O)N2C(=O)C[C@H]2S1(=O)=O | 233.245 | -0.795 | 1 | 4 | 1 | 86.785 | -1.099 | 0.137 | 40.362 | -2.744 | No | No | No | -0.661 | 0.74 | -0.418 | -3.497 | No | No | No | No | No | No | No | 0.509 | No | No | 1.041 | No | No | 1.832 | 2.341 | Yes |
| CHEMBL1241855 | COc1cc(OC)c(/C=C/S(=O)(=O)Cc2ccc(OC)c(NCC(=O)O)c2)c(OC)c1 | 451.497 | 2.8032 | 11 | 8 | 2 | 180.669 | -3.603 | 0.886 | 57.793 | -2.735 | Yes | No | No | -0.655 | 0.052 | -1.264 | -3.548 | No | No | No | No | No | No | No | 0.683 | No | No | 0.429 | No | No | 2.45 | 2.64 | No |
| CHEMBL3137308 | NC(=O)c1cnc2[nH]ccc2c1N[C@H]1[C@H]2CC3C[C@@H]1C[C@](O)(C3)C2 | 326.4 | 2.0133 | 3 | 4 | 4 | 139.384 | -2.755 | 0.914 | 86.656 | -2.824 | Yes | No | No | 0.468 | 0.398 | -0.968 | -4.268 | No | No | Yes | No | No | No | No | 0.239 | No | No | -0.555 | No | No | 2.572 | 1.268 | Yes |
| CHEMBL4297627 | C[C@@H]1CCc2ncc(F)cc2[C@H]2CCCN2c2ccn3ncc(c3n2)C(=O)N1 | 380.427 | 2.6694 | 0 | 6 | 1 | 161.144 | -3.571 | 1.288 | 98.61 | -2.804 | Yes | No | No | 0.038 | 0.249 | -0.692 | -2.995 | No | Yes | Yes | No | No | No | No | 0.657 | No | No | -0.391 | No | Yes | 2.899 | 1.294 | Yes |
| CHEMBL1421 | Cc1nc(Nc2ncc(C(=O)Nc3c(C)cccc3Cl)s2)cc(N2CCN(CCO)CC2)n1 | 488.017 | 3.31354 | 7 | 9 | 3 | 201.002 | -3.619 | 0.527 | 73.447 | -2.772 | Yes | Yes | No | 0.708 | 0.165 | -1.576 | -3.161 | No | Yes | No | No | No | No | Yes | 0.472 | No | No | -0.075 | No | No | 2.667 | 1.549 | Yes |
| CHEMBL1188395 | NCc1cnc(S)n1[C@H]1CCc2c(F)cc(F)cc2C1 | 295.358 | 2.6387 | 2 | 4 | 2 | 120.772 | -2.497 | 1.248 | 91.297 | -2.765 | Yes | No | No | 0.281 | 0.528 | 0.138 | -2.013 | No | No | Yes | No | No | No | No | 0.994 | No | No | -0.063 | No | No | 2.675 | 1.485 | Yes |
| CHEMBL4297181 | CO[C@@H](C)Cn1c(=O)n(C)c2cnc3ccc(-c4cncc(C(C)(C)O)c4)cc3c21 | 406.486 | 3.2125 | 5 | 7 | 1 | 174.129 | -3.104 | 1.448 | 95.859 | -2.735 | Yes | Yes | Yes | 0.411 | 0.273 | -0.971 | -2.672 | No | Yes | Yes | Yes | Yes | No | Yes | 1.011 | Yes | Yes | 0.496 | No | Yes | 2.528 | 0.671 | Yes |
| CHEMBL4297066 | CCCCCCCC(=O)N[C@H](CN1CCCC1)[C@H](O)c1ccc2c(c1)OCCO2.CCCCCCCC(=O)N[C@H](CN1CCCC1)[C@H](O)c1ccc2c(c1)OCCO2.O=C(O)C(O)C(O)C(=O)O | 959.188 | 4.742 | 25 | 14 | 8 | 400.83 | -2.892 | -0.919 | 0 | -2.735 | Yes | No | No | -0.508 | 0.29 | -3.046 | -4.924 | No | Yes | No | No | No | No | No | 0.984 | No | No | 0.452 | No | No | 2.463 | 4.255 | Yes |
| CHEMBL3137336 | Cn1ncc(Cl)c1-c1cc(C(=O)N[C@H](CN)Cc2ccc(F)c(F)c2)oc1Cl | 429.254 | 3.5649 | 6 | 5 | 2 | 168.761 | -4.193 | 1.109 | 93.157 | -2.835 | Yes | Yes | No | 0.534 | 0.173 | -0.906 | -2.958 | No | Yes | No | No | No | No | Yes | 0.595 | No | No | -0.138 | No | Yes | 2.382 | 1.638 | Yes |
| CHEMBL477772 | Cc1ccc(Nc2nccc(N(C)c3ccc4c(C)n(C)nc4c3)n2)cc1S(N)(=O)=O | 437.529 | 3.13904 | 5 | 8 | 2 | 180.144 | -3.7 | 0.018 | 94.544 | -2.761 | Yes | Yes | Yes | -0.11 | 0.152 | -1.3 | -2.462 | No | Yes | Yes | Yes | No | No | Yes | 0.275 | No | No | 0.21 | No | Yes | 2.83 | 1.23 | Yes |
| CHEMBL107747 | CC1(C)CCC[C@H](NC(=O)[C@@H](S)Cc2ccccc2)C(=O)N1CC(=O)O | 378.494 | 1.888 | 6 | 4 | 3 | 158.913 | -3.136 | 0.854 | 94.702 | -2.735 | Yes | No | No | -1.336 | 0.398 | -0.942 | -2.869 | No | No | No | No | No | No | No | -0.018 | No | No | 1.047 | No | No | 1.456 | 2.866 | Yes |
| CHEMBL445 | CNCCC=C1c2ccccc2CCc2ccccc21 | 263.384 | 3.8264 | 3 | 1 | 1 | 120.898 | -4.426 | 1.453 | 98.72 | -2.559 | Yes | Yes | No | 1.639 | 0.058 | 0.823 | -1.236 | No | Yes | Yes | No | No | Yes | No | 0.929 | Yes | No | 0.271 | No | Yes | 2.682 | 1.23 | Yes |
| CHEMBL3265032 | c1cn2cc(-c3ccc4cn[nH]c4c3)nc(Nc3ccc(N4CCOCC4)cc3)c2n1 | 411.469 | 3.8528 | 4 | 7 | 2 | 178.059 | -2.914 | 1.029 | 90.499 | -2.735 | Yes | No | Yes | 0.444 | 0.166 | -1.227 | -2.214 | No | Yes | Yes | Yes | Yes | No | Yes | 0.91 | Yes | No | 0.327 | No | Yes | 2.452 | 0.637 | Yes |
| CHEMBL270995 | Cc1cc2c(F)c(Oc3ncnn4cc(OC[C@@H](C)OC(=O)[C@H](C)N)c(C)c34)ccc2[nH]1 | 441.463 | 3.41654 | 7 | 8 | 2 | 182.832 | -3.256 | 1.935 | 84.385 | -2.735 | Yes | Yes | No | 0.702 | 0.124 | -0.807 | -3.251 | No | Yes | No | No | Yes | No | Yes | 0.576 | No | No | 0.387 | No | Yes | 2.363 | 1.037 | Yes |
| CHEMBL494 | CC#CCC(C)[C@H](O)/C=C/[C@@H]1[C@H]2C/C(=C/CCCC(=O)O)C[C@H]2C[C@H]1O | 360.494 | 3.5413 | 8 | 3 | 3 | 156.556 | -3.534 | 0.767 | 95.206 | -2.734 | Yes | No | Yes | -1.152 | 0.151 | -0.533 | -2.499 | No | Yes | No | No | No | No | No | 1.408 | No | No | -1.958 | No | No | 2.822 | 2.095 | No |
| CHEMBL1165 | CCOC(=O)[C@H](CCc1ccccc1)N[C@@H](C)C(=O)N1Cc2cc(OC)c(OC)cc2C[C@H]1C(=O)O | 498.576 | 2.5843 | 11 | 7 | 2 | 210.886 | -3.183 | 0.402 | 58.321 | -2.735 | Yes | No | No | -0.345 | 0.418 | -0.963 | -3.487 | No | No | No | No | No | No | No | 0.995 | No | No | 0.397 | No | No | 2.238 | 2.528 | Yes |
| CHEMBL4297511 | N[C@@H](CCS(=O)(=O)O)CSSC[C@@H](N)CCS(=O)(=O)O | 368.524 | -0.4218 | 11 | 8 | 4 | 126.877 | -2.357 | -0.547 | 0 | -2.735 | Yes | No | No | -0.523 | 0.756 | -1.512 | -3.469 | No | No | No | No | No | No | No | -0.046 | No | No | 0.324 | No | No | 2.223 | 2.629 | No |
| CHEMBL4297593 | CONC(=N)c1ccc(CNC(=O)[C@@H]2CCN2C(=O)[C@H](O)c2cc(Cl)cc(OC(F)F)c2)cc1 | 496.898 | 2.36847 | 9 | 6 | 4 | 198.61 | -3.668 | 0.625 | 56.052 | -2.735 | Yes | Yes | No | 0.209 | 0.124 | -1.48 | -3.226 | No | No | No | No | No | No | No | -0.054 | No | No | 0.242 | No | No | 2.707 | 3.104 | Yes |
| CHEMBL591118 | COc1cc2c(cc1OC)[C@@H]1C[C@H](N)[C@@H](N3C[C@@H](CF)CC3=O)CN1CC2 | 377.46 | 1.5206 | 4 | 5 | 1 | 158.937 | -2.226 | 0.362 | 93.079 | -2.769 | Yes | No | No | 0.305 | 0.618 | -0.998 | -2.952 | Yes | Yes | No | No | No | No | No | 0.858 | No | No | -0.196 | No | Yes | 3.176 | 0.744 | Yes |
| CHEMBL4594348 | C=CC(=O)N1CCC[C@@H](c2ccc(C(N)=O)c3[nH]ccc23)C1 | 297.358 | 2.1588 | 3 | 2 | 2 | 128.334 | -3.751 | 0.922 | 93.214 | -3.491 | Yes | No | No | 0.161 | 0.318 | -0.565 | -3.314 | No | No | Yes | No | No | No | No | 0.551 | No | No | 0.106 | No | No | 2.405 | 1.087 | No |
| CHEMBL204656 | COc1cc2c(cc1Cc1cccc(Cl)c1F)c(=O)c(C(=O)O)cn2[C@H](CO)C(C)C | 447.89 | 4.281 | 7 | 5 | 2 | 183.013 | -4.328 | 1.013 | 97.598 | -2.735 | Yes | No | No | -1.131 | 0.088 | -0.855 | -2.977 | No | No | No | No | Yes | No | No | 0.437 | No | No | 0.576 | No | No | 3.288 | 2.112 | No |
| CHEMBL1738797 | CCc1cc2c(cc1N1CCC(N3CCOCC3)CC1)C(C)(C)c1[nH]c3cc(C#N)ccc3c1C2=O | 482.628 | 4.77328 | 3 | 5 | 1 | 212.388 | -4.253 | 0.665 | 96.017 | -2.83 | Yes | Yes | Yes | 1.532 | 0.114 | 0.017 | -1.685 | Yes | Yes | Yes | Yes | No | Yes | Yes | 1.147 | No | Yes | 0.014 | No | Yes | 2.633 | 0.523 | Yes |
| CHEMBL1256841 | O=C(CCNNC(=O)c1ccncc1)NCc1ccccc1 | 298.346 | 1.0225 | 7 | 4 | 3 | 128.476 | -3.189 | 0.882 | 61.447 | -3.203 | Yes | No | No | -0.191 | 0.305 | -0.724 | -3.088 | No | No | Yes | Yes | Yes | Yes | No | 0.746 | No | Yes | 0.266 | No | No | 2.083 | 1.308 | No |
| CHEMBL4297644 | Cc1n[nH]cc1-c1cc2nc([C@@H]3CC4CCN3CC4)[nH]c(=O)c2s1 | 341.44 | 2.83992 | 2 | 5 | 2 | 142.476 | -3.012 | 0.714 | 91.657 | -2.744 | Yes | No | No | 1.467 | 0.231 | -1.18 | -3.41 | Yes | Yes | Yes | No | No | No | No | 1.126 | Yes | Yes | -0.256 | No | Yes | 2.723 | 1.356 | Yes |
| CHEMBL4303201 | COc1cc2c(Oc3ccc4[nH]c(C)cc4c3F)ccnc2cc1OCC1(N)CC1 | 407.445 | 4.83462 | 6 | 5 | 2 | 172.311 | -3.748 | 1.113 | 92.503 | -2.736 | Yes | Yes | Yes | 0.944 | 0.19 | -0.872 | -2.292 | No | Yes | Yes | Yes | No | No | Yes | 1.185 | Yes | No | 0.402 | No | Yes | 2.99 | 1.105 | Yes |
| CHEMBL4297619 | O=C(O)CNC(=O)c1c(O)cc(CCc2ccccc2)n2ncnc12 | 340.339 | 1.0345 | 6 | 6 | 3 | 142.3 | -3.13 | 0.754 | 49.412 | -2.748 | Yes | No | No | -0.973 | 0.086 | -1.223 | -3.893 | No | Yes | No | No | No | No | No | 0.599 | No | No | 0.486 | No | No | 2.084 | 2.623 | Yes |
| CHEMBL2013119 | COc1cc(OC)c(/C=C/S(=O)(=O)Cc2ccc(OC)c(NCC(=O)[O-])c2)c(OC)c1.[Na+] | 473.479 | -1.5275 | 11 | 9 | 1 | 209.082 | -3.441 | 0.577 | 35.923 | -2.735 | Yes | No | No | -0.792 | 0.085 | -1.377 | -4.015 | No | No | No | No | No | No | No | 1.257 | No | No | 0.592 | No | No | 2.239 | 1.637 | No |
| CHEMBL991 | Cc1cn([C@H]2C=C[C@@H](CO)O2)c(=O)[nH]c1=O | 224.216 | -0.70908 | 2 | 5 | 2 | 90.806 | -2.868 | 0.773 | 78.893 | -3.664 | No | No | No | -0.159 | 0.721 | -0.796 | -3.18 | No | No | No | No | No | No | No | 0.709 | No | No | 0.762 | No | No | 2.081 | 1.125 | Yes |
| CHEMBL4073443 | Cc1cc(Nc2cc(N)ncn2)c(=O)n2c1C(=O)NC21CCCCC1 | 340.387 | 1.63282 | 2 | 7 | 3 | 144.028 | -4.443 | -0.026 | 85.743 | -2.821 | No | No | No | 0.074 | 0.461 | -1.059 | -3.123 | No | No | No | No | No | No | No | 0.537 | No | No | -0.119 | No | No | 2.645 | 0.462 | Yes |
| CHEMBL1581 | CCC[C@H](N[C@@H](C)C(=O)N1[C@H](C(=O)O)C[C@@H]2CCCC[C@@H]21)C(=O)OCC | 368.474 | 1.9406 | 8 | 5 | 2 | 154.999 | -2.322 | 0.715 | 46.318 | -2.735 | No | No | No | -0.46 | 0.66 | -0.957 | -3.136 | No | No | No | No | No | No | No | 0.607 | No | No | 0.202 | No | No | 1.776 | 2.096 | Yes |
| CHEMBL225304 | NN=C(N)N | 74.087 | -1.8665 | 0 | 2 | 3 | 29.624 | -0.992 | 0.58 | 68.204 | -2.842 | Yes | No | No | -0.234 | 0.854 | -0.398 | -3.498 | No | No | No | No | No | No | No | 0.402 | No | Yes | 0.769 | No | No | 2.649 | 0.402 | No |
| CHEMBL3301601 | CN1C(=N)N[C@](C)(c2cc(NC(=O)c3ccc(F)cn3)ccc2F)CS1(=O)=O | 409.418 | 1.62687 | 3 | 5 | 3 | 159.69 | -3.168 | 0.779 | 72.579 | -2.749 | Yes | No | No | 0.19 | 0.349 | -1.679 | -3.472 | No | Yes | No | No | No | No | No | 0.24 | No | No | 0.065 | No | No | 2.397 | 1.799 | Yes |
| CHEMBL3989972 | COc1cn(-c2ccc(-n3cccn3)cc2F)nc(-c2ccnn2-c2ccccc2)c1=O | 428.427 | 3.4186 | 5 | 8 | 0 | 181.298 | -3.851 | 0.97 | 100 | -2.734 | No | Yes | Yes | 0.494 | 0.343 | -1.394 | -3.51 | No | Yes | Yes | No | No | No | No | 0.675 | No | Yes | 0.598 | No | Yes | 2.658 | 0.401 | Yes |
| CHEMBL1449676 | Cc1ncc([N+](=O)[O-])n1CC(O)CCl | 219.628 | 0.69942 | 4 | 5 | 1 | 85.28 | -1.781 | 0.576 | 94.84 | -2.759 | Yes | No | No | -0.229 | 0.65 | -0.824 | -3.023 | No | No | No | No | No | No | No | 0.624 | No | Yes | 0.142 | No | No | 2.158 | 1.503 | No |
| CHEMBL1592 | CCOC(=O)[C@H](CCc1ccccc1)N[C@@H](C)C(=O)N1Cc2ccccc2C[C@H]1C(=O)O | 438.524 | 2.5671 | 9 | 5 | 2 | 187.929 | -3.241 | 0.306 | 50.224 | -2.735 | Yes | No | No | -0.333 | 0.417 | -0.586 | -2.896 | No | No | No | No | No | No | No | 0.82 | No | No | 0.165 | No | No | 2.491 | 2.25 | Yes |
| CHEMBL2105754 | Cn1c(=O)cc(N2CCC[C@@H](N)C2)n(Cc2cc(F)ccc2C#N)c1=O.O=C(O)CCC(=O)O | 475.477 | 0.46938 | 6 | 9 | 3 | 193.619 | -3.001 | -0.407 | 21.552 | -2.735 | Yes | No | No | -0.837 | 0.455 | -1.38 | -3.186 | No | No | No | No | No | No | No | 0.034 | No | No | 1.186 | No | No | 1.922 | 1.934 | Yes |
| CHEMBL3989910 | O=C(O)c1ccc(-c2ccc3cc(O)ccc3n2)c(Cl)c1 | 299.713 | 3.959 | 2 | 3 | 2 | 124.808 | -3.315 | 0.812 | 91.833 | -2.7 | Yes | No | No | -0.115 | 0.229 | -0.307 | -1.911 | No | No | Yes | No | No | No | No | 0.294 | No | No | 0.601 | No | No | 2.392 | 1.176 | No |
| CHEMBL4466233 | CC(=O)N1CCC(Nc2cc(C(=O)NC[C@H](O)CN3CCc4ccccc4C3)ncn2)CC1 | 452.559 | 1.0484 | 7 | 7 | 3 | 193.762 | -3.077 | 0.073 | 65.7 | -2.753 | Yes | No | No | -0.063 | 0.46 | -0.909 | -3.41 | No | No | No | No | No | No | No | 0.802 | No | No | 0.198 | No | No | 2.263 | 0.879 | Yes |
| CHEMBL3545369 | NS(=O)(=O)NCCNc1nonc1/C(=N/O)Nc1ccc(F)c(Br)c1 | 438.239 | 0.4241 | 7 | 8 | 5 | 149.282 | -3.262 | 0.396 | 59.151 | -2.749 | Yes | No | No | -1.091 | 0.271 | -1.664 | -3.885 | No | No | No | No | No | No | No | -0.464 | No | No | 0.796 | No | No | 2.746 | 2.189 | Yes |
| CHEMBL2105685 | Cl.S=C(NCc1ccc2c(c1)OCO2)N1CCN(c2ncnc3c2oc2ccccc23)CC1 | 483.981 | 3.7232 | 3 | 7 | 1 | 201.437 | -3.785 | 1.356 | 89.811 | -2.765 | Yes | Yes | Yes | 0.361 | 0.024 | -0.702 | -2.186 | No | Yes | Yes | Yes | Yes | No | Yes | -0.188 | No | No | 0.022 | No | Yes | 2.346 | 0.51 | Yes |
| CHEMBL331378 | C[C@H](CSC(=O)c1ccccc1)C(=O)N1C[C@@H](Sc2ccccc2)C[C@H]1C(=O)O | 429.563 | 4.0425 | 7 | 5 | 1 | 178.417 | -4.604 | 0.688 | 91.706 | -2.735 | Yes | No | No | -1.142 | 0 | -0.562 | -2.304 | No | Yes | No | No | No | No | No | 0.29 | No | No | 0.738 | No | No | 2.457 | 2.475 | Yes |
| CHEMBL4249337 | Nc1nc2cc(CC[C@H]3C[C@@H](n4ccc5c(N)ncnc54)[C@H](O)[C@@H]3O)ccc2cc1Br | 483.37 | 2.822 | 4 | 8 | 4 | 186.614 | -3.261 | 0.422 | 89.509 | -2.739 | Yes | No | No | 0.242 | 0.035 | -1.351 | -2.66 | No | Yes | No | Yes | Yes | No | Yes | -0.056 | No | No | 0.141 | No | Yes | 2.602 | 1.898 | Yes |
| CHEMBL1518 | CCCc1cc(=O)[nH]c(=S)[nH]1 | 170.237 | 1.38499 | 2 | 2 | 2 | 69.486 | -2.888 | 1.208 | 93.101 | -3.154 | No | No | No | 0.066 | 0.623 | -0.303 | -2.993 | No | No | No | No | No | No | No | 0.075 | No | No | 0.915 | No | No | 2.388 | 1.317 | Yes |
| CHEMBL2110627 | CCOC(=O)[C@H](CCc1ccccc1)N[C@H]1CS[C@H](c2cccs2)CN(CC(=O)O)C1=O | 476.62 | 2.9719 | 10 | 7 | 2 | 195.692 | -3.153 | -0.029 | 50.734 | -2.735 | Yes | No | No | -0.16 | 0.458 | -0.961 | -2.968 | No | Yes | No | No | No | No | No | 0.586 | No | No | -0.261 | No | No | 2.866 | 2.219 | Yes |
| CHEMBL4298138 | C[C@H]1CNC(=O)c2cnn3ccc(nc23)N[C@H](C)c2cc(F)ccc2O1 | 355.373 | 2.5522 | 0 | 6 | 2 | 148.739 | -3.126 | 1.362 | 95.194 | -2.821 | No | No | No | 0.331 | 0.186 | -0.038 | -2.902 | No | Yes | Yes | No | No | No | No | 0.417 | No | No | -0.305 | No | Yes | 2.271 | 1.194 | Yes |
| CHEMBL4204794 | Cn1cc(-c2cc3c(N4CCN(c5ncc([C@@](C)(N)c6ccc(F)cc6)cn5)CC4)ncnn3c2)cn1 | 498.57 | 2.6077 | 5 | 10 | 1 | 212.749 | -3.255 | 0.33 | 100 | -2.748 | Yes | Yes | Yes | 0.747 | 0.264 | -1.496 | -2.983 | No | Yes | No | No | No | No | Yes | 0.311 | Yes | No | -0.194 | No | Yes | 3.381 | 0.874 | Yes |
| CHEMBL289351 | COc1ccc(/C=C\c2cc(OC)c(OC)c(OC)c2)cc1OP(=O)([O-])[O-].[Na+].[Na+] | 440.296 | -3.8931 | 8 | 8 | 0 | 213.521 | -3.638 | 0.359 | 48.251 | -2.836 | Yes | Yes | No | -1.32 | 0.355 | -1.411 | -3.047 | No | Yes | No | No | No | No | No | -1.374 | No | Yes | 0.676 | No | No | 1.15 | 1.941 | No |
| CHEMBL4297534 | CN1CC[C@@H](NC(=O)Nc2ccc(C#N)cc2)C[C@@H]1c1nc2ccccc2[nH]1.O=C(O)/C=C\C(=O)O | 490.52 | 3.10338 | 5 | 6 | 5 | 206.614 | -2.893 | -0.595 | 24.464 | -2.735 | Yes | No | No | -0.095 | 0.232 | -1.707 | -3.313 | No | No | No | No | No | No | No | 0.172 | No | No | 0.435 | No | No | 2.479 | 3.233 | Yes |
| CHEMBL1200343 | CC(C)(C)N.CCC[C@H](N[C@@H](C)C(=O)N1[C@H](C(=O)O)C[C@@H]2CCCC[C@@H]21)C(=O)OCC | 441.613 | 2.6842 | 8 | 6 | 3 | 186.804 | -2.75 | 0.563 | 40.612 | -2.735 | No | No | No | -0.376 | 0.705 | -0.927 | -3.12 | No | Yes | No | No | No | No | No | 0.647 | No | No | 0.148 | No | No | 1.757 | 2.194 | Yes |
| CHEMBL1551724 | NC(=O)c1ncn([C@@H]2O[C@H](CO)[C@@H](O)[C@H]2O)c1N | 258.234 | -2.8243 | 3 | 8 | 5 | 101.592 | -2.11 | -0.92 | 41.668 | -2.735 | Yes | No | No | -0.598 | 0.753 | -1.032 | -4.199 | No | No | No | No | No | No | No | 0.768 | No | No | 1.012 | No | No | 1.83 | 3.391 | No |
| CHEMBL2216870 | CC[C@H](Nc1ncnc2[nH]cnc12)c1nc2cccc(F)c2c(=O)n1-c1ccccc1 | 415.432 | 3.7543 | 5 | 7 | 2 | 175.398 | -2.898 | 1.182 | 85.014 | -2.735 | Yes | Yes | Yes | -0.099 | 0.349 | -1.697 | -3.84 | No | No | No | Yes | Yes | No | Yes | 0.461 | No | Yes | 0.41 | No | Yes | 2.545 | 2.679 | No |
| CHEMBL3900409 | Cn1c(=O)oc2ccc(-c3ccc(C[C@@H](C#N)NC(=O)[C@@H]4CNCCCO4)cc3)cc21 | 420.469 | 1.72788 | 5 | 7 | 2 | 178.967 | -3.812 | 0.088 | 81.723 | -2.726 | Yes | Yes | Yes | 0.71 | 0.128 | -0.796 | -2.696 | No | Yes | No | No | No | No | No | 0.593 | Yes | No | -0.301 | No | Yes | 2.243 | 2.421 | Yes |
| CHEMBL518924 | COc1ccc(/C=C\c2cc(OC)c(OC)c(OC)c2)c(O)c1O | 332.352 | 3.3026 | 6 | 6 | 2 | 140.031 | -3.689 | 1.076 | 90.33 | -3.067 | Yes | Yes | No | -0.732 | 0.175 | -0.702 | -2.786 | No | No | Yes | Yes | No | No | Yes | 0.152 | No | No | 1.05 | No | No | 1.885 | 1.49 | No |
| CHEMBL36255 | CC(C)OCCn1c(=S)[nH]c(=O)c2[nH]ccc21 | 253.327 | 1.81219 | 4 | 4 | 2 | 103.43 | -4.344 | 0.734 | 94.087 | -3.176 | No | No | No | 0.187 | 0.5 | 0 | -3.082 | No | No | Yes | No | No | No | No | 0.347 | No | No | 0.864 | No | No | 2.286 | 1.294 | Yes |
| CHEMBL4297594 | O=C(O)/C=C1\CN([C@H](C(=O)O)c2ccccc2Cl)CCC1S | 341.816 | 2.4808 | 4 | 4 | 3 | 138.15 | -2.863 | 0.738 | 91.526 | -2.735 | No | No | No | -0.629 | 0.46 | -1.22 | -3.376 | No | No | No | No | No | No | No | 0.066 | No | No | 0.777 | No | No | 1.961 | 1.94 | No |
| CHEMBL2347655 | CCCCC(CC)COC(=O)C=Cc1ccc(OC)cc1 | 290.403 | 4.468 | 9 | 3 | 0 | 127.51 | -5.28 | 1.242 | 95.799 | -2.488 | No | Yes | No | 0.247 | 0.059 | 0.054 | -1.84 | No | Yes | Yes | Yes | No | No | No | 0.92 | No | No | 1.068 | No | Yes | 1.919 | 2.28 | No |
| CHEMBL1201398 | CCOC(=O)COc1ccc2c(C)c(CCN(CC)CC)c(=O)oc2c1Cl | 395.883 | 3.58102 | 9 | 6 | 0 | 163.545 | -4.341 | 1.326 | 92.786 | -2.878 | No | Yes | Yes | 0.786 | 0.199 | -0.844 | -2.736 | No | Yes | No | No | No | No | Yes | 0.837 | Yes | No | 0.44 | No | Yes | 2.878 | 0.662 | Yes |
| CHEMBL3183184 | CC(C)n1nc(-c2ccc3oc(N)nc3c2)c2c(N)ncnc21 | 309.333 | 2.3798 | 2 | 8 | 2 | 130.687 | -2.874 | 0.48 | 87.693 | -2.741 | Yes | No | No | 0.058 | 0.141 | -0.879 | -2.469 | No | Yes | Yes | No | No | No | Yes | 0.382 | No | No | 0.129 | No | Yes | 2.957 | 0.72 | Yes |
| CHEMBL255066 | CCn1c(C(=O)N(C2CC2)C2CC2)cc2c3c(ncn3C)c(Nc3cc(C)n(C)n3)nc21 | 432.532 | 3.49542 | 6 | 8 | 1 | 185.575 | -2.73 | 1.269 | 95.428 | -2.735 | Yes | No | No | 0.431 | 0.369 | -1.152 | -3.161 | No | No | No | No | Yes | No | No | 1.101 | Yes | Yes | 0.485 | No | Yes | 2.4 | -0.995 | Yes |
| CHEMBL3545097 | COc1cc(C(=O)c2c[nH]c(-c3c[nH]c4ccccc34)n2)cc(OC)c1OC | 377.4 | 3.8148 | 6 | 5 | 2 | 160.827 | -2.987 | 0.965 | 90.489 | -2.735 | Yes | No | Yes | 0.273 | 0.081 | -1.314 | -2.48 | Yes | No | Yes | Yes | Yes | No | Yes | 0.997 | Yes | Yes | 0.37 | No | Yes | 2.032 | 2.395 | No |
| CHEMBL1944698 | COc1ccc(/C=C\c2cc(OC)c(OC)c(OC)c2)cc1OP(=O)(O)O.NC(CO)(CO)CO | 517.468 | 1.0237 | 11 | 10 | 6 | 202.883 | -3.797 | -0.431 | 49.07 | -2.745 | Yes | Yes | No | -1.414 | 0.413 | -2.044 | -3.405 | No | Yes | No | No | No | No | No | 0.75 | No | No | 1.045 | No | Yes | 1.867 | 1.611 | No |
| CHEMBL3545215 | CC(C)(C#N)c1cc(Cn2cncn2)cc(C(C)(C)C#N)c1 | 293.374 | 2.92876 | 4 | 5 | 0 | 130.385 | -4.111 | 1.122 | 99.544 | -2.67 | No | No | No | 0.011 | 0.172 | -0.349 | -2.751 | No | Yes | Yes | Yes | No | No | No | 1.524 | No | Yes | 0.394 | No | No | 2.196 | 1.42 | Yes |
| CHEMBL107360 | CN(C)c1cc2c(cc1Sc1nc3c(N)nccc3n1CCNCC(C)(C)C)OCO2 | 442.589 | 3.5951 | 7 | 9 | 2 | 186.672 | -2.872 | 0.85 | 88.006 | -2.735 | Yes | Yes | Yes | 0.449 | 0.202 | -1.007 | -2.482 | No | Yes | No | No | Yes | No | Yes | 0.563 | Yes | Yes | 0.406 | No | Yes | 2.631 | 1.294 | Yes |
| CHEMBL2163631 | Cc1ccc(S(=O)(=O)O)cc1.N#C[C@@H]1C[C@H](F)CN1C(=O)[C@@H](N)C(c1ccc(F)cc1)c1ccc(F)cc1 | 545.583 | 4.1283 | 5 | 5 | 2 | 218.609 | -3.298 | 0.632 | 58.968 | -2.735 | Yes | No | Yes | -0.267 | 0.107 | -1.416 | -3.094 | No | No | No | No | No | No | No | 0.352 | No | No | 0.477 | No | No | 2.335 | 1.692 | Yes |
| CHEMBL2105643 | CC(C)(NC(=O)O[C@@H]1CN2CCC1CC2)c1csc(-c2ccc(F)cc2)n1 | 389.496 | 4.0047 | 4 | 5 | 1 | 162.102 | -3.805 | 1.27 | 90.947 | -2.742 | Yes | No | No | 0.897 | 0.259 | 0.338 | -2.976 | No | Yes | No | Yes | No | No | No | 0.849 | No | No | -0.799 | No | Yes | 3.112 | 0.033 | Yes |
| CHEMBL1399 | Cc1ccc(Nc2nccc(N(C)c3ccc4c(C)n(C)nc4c3)n2)cc1S(N)(=O)=O.Cl | 473.99 | 3.56084 | 5 | 8 | 2 | 192.297 | -3.833 | -0.02 | 94.3 | -2.755 | Yes | Yes | Yes | -0.081 | 0.137 | -1.465 | -2.396 | No | Yes | Yes | Yes | Yes | No | Yes | -0.461 | No | No | 0.232 | No | Yes | 2.845 | 1.195 | Yes |
| CHEMBL2419346 | O=C(CCCCNC(=O)c1nc(-c2nccs2)sc1C1CC1)NO | 366.468 | 2.5495 | 8 | 7 | 3 | 146.658 | -3.373 | 1.371 | 76.293 | -2.795 | Yes | No | No | -0.053 | 0.146 | -1.413 | -3.24 | No | Yes | No | No | No | No | No | -0.102 | No | No | 0.454 | No | No | 2.922 | 1.53 | Yes |
| CHEMBL2104962 | N#CCNC(=O)c1ccc(-c2ccnc(Nc3ccc(N4CCOCC4)cc3)n2)cc1 | 414.469 | 2.97718 | 6 | 7 | 2 | 180.061 | -4.171 | 0.853 | 95.566 | -2.785 | Yes | Yes | Yes | 0.108 | 0.126 | -0.434 | -2.569 | No | Yes | Yes | Yes | No | No | Yes | 0.39 | No | No | 0.263 | No | Yes | 3.065 | 0.528 | Yes |
| CHEMBL4297611 | Cc1ccc(-c2ncc(Cl)cc2-c2ccc(S(C)(=O)=O)cc2)cn1 | 358.85 | 4.17592 | 3 | 4 | 0 | 145.643 | -3.884 | 1.363 | 99.297 | -2.672 | No | No | Yes | 0.325 | 0.286 | 0.295 | -1.892 | No | Yes | Yes | Yes | Yes | No | Yes | 0.183 | No | No | -0.034 | No | No | 2.499 | 0.581 | Yes |
| CHEMBL1201733 | C=CC(=O)Nc1cccc(Oc2nc(Nc3ccc(N4CCN(C)CC4)c(F)c3)nc3[nH]ccc23)c1.O.O.O=C(O)/C=C\C(=O)O | 639.641 | 2.5716 | 9 | 9 | 5 | 261.809 | -2.964 | -0.744 | 19.72 | -2.735 | Yes | No | No | 0.308 | 0.053 | -2.157 | -4.479 | No | No | No | No | No | No | No | -0.222 | No | No | 0.588 | No | No | 2.422 | 3.139 | Yes |
| CHEMBL3287255 | CC1(C)CNc2cc(NC(=O)c3cccnc3NCc3ccncc3)ccc21 | 373.46 | 4.0441 | 5 | 5 | 3 | 163.98 | -3.657 | 1.053 | 94.845 | -2.798 | Yes | Yes | Yes | 0.53 | 0.089 | -0.818 | -2.884 | No | Yes | Yes | Yes | Yes | No | Yes | 0.293 | No | Yes | -0.152 | No | Yes | 3.091 | 0.354 | Yes |
| CHEMBL1078178 | Cc1cc(C)c(Nc2ccnc(Nc3ccc(C#N)cc3)n2)c(C)c1 | 329.407 | 4.76074 | 4 | 5 | 2 | 146.937 | -4.341 | 0.805 | 93.189 | -2.806 | Yes | No | Yes | 0.127 | 0.136 | 0.115 | -1.675 | No | Yes | Yes | Yes | No | No | Yes | 0.026 | No | No | 0.344 | No | Yes | 2.763 | 0.851 | Yes |
| CHEMBL416146 | C=CC(=O)N1CC[C@H](n2nc(C#Cc3cc(OC)cc(OC)c3)c3c(N)ncnc32)C1 | 418.457 | 1.7849 | 4 | 8 | 1 | 179.023 | -3.665 | 0.1 | 73.982 | -2.751 | Yes | Yes | No | -0.193 | 0.133 | -0.924 | -3.721 | No | Yes | No | No | Yes | No | Yes | 0.618 | No | No | -0.028 | No | No | 2.271 | 1.551 | Yes |
| CHEMBL4297866 | Cc1ccc(-c2cc(C(F)(F)F)nn2-c2ccc(S(N)(=O)=O)cc2)cc1 | 381.379 | 3.51392 | 3 | 4 | 1 | 147.48 | -4.441 | 1.005 | 92.728 | -2.736 | Yes | No | Yes | -0.287 | 0.153 | -1.024 | -2.065 | No | Yes | Yes | Yes | Yes | No | Yes | 0.461 | No | No | 0.261 | No | No | 2.389 | 1.027 | Yes |
| CHEMBL572881 | NCCCC(N)(C(=O)O)C(F)F | 182.17 | -0.2275 | 5 | 3 | 3 | 68.531 | -0.505 | 0.833 | 72.865 | -2.735 | No | No | No | -0.327 | 0.915 | -0.803 | -3.546 | Yes | No | No | No | No | No | No | 0.189 | No | Yes | 1.339 | No | No | 1.607 | 1.891 | No |
| CHEMBL70663 | I | 127.912 | 0.618 | 0 | 0 | 0 | 24.512 | -0.463 | 1.379 | 100 | -2.477 | Yes | No | No | -0.028 | 0.756 | 0.013 | -2.298 | No | No | No | No | No | No | No | 0.089 | No | No | 1.182 | No | No | 2.357 | 1.154 | No |
| CHEMBL3701238 | CCC(=O)N1CC[C@H](Nc2ncnc3c2CN(c2cnc(OC)c(C(F)(F)F)c2)CC3)C1.O=P(O)(O)O | 548.459 | 1.9559 | 5 | 8 | 4 | 210.294 | -3.572 | 0.393 | 61.429 | -2.748 | No | No | No | -1.675 | 0.235 | -2.338 | -4.627 | No | Yes | No | No | No | No | No | 0.271 | No | No | 0.736 | No | No | 3.352 | 2.443 | Yes |
| CHEMBL118 | CC(C)(C)c1ccc(C(=O)Nc2cn3cc(-n4ccnc4)ccc3n2)cc1.Cl.Cl | 432.355 | 4.9134 | 3 | 5 | 1 | 181.364 | -2.892 | 1.425 | 93.251 | -2.735 | Yes | Yes | Yes | 0.087 | 0.355 | 0.12 | -1.824 | No | No | Yes | Yes | Yes | No | Yes | 1.035 | Yes | Yes | 0.466 | No | Yes | 2.442 | 1.212 | No |
| CHEMBL830 | CC(C)(C)OC[C@@H]1C(=O)NCCN1C(=O)C[C@H](N)Cc1cc(F)c(F)cc1F | 401.429 | 1.5059 | 6 | 4 | 2 | 161.753 | -3.506 | 0.305 | 95.995 | -2.791 | Yes | No | No | 0.158 | 0.355 | -0.881 | -3.268 | No | No | No | No | No | No | No | 0.677 | No | No | -0.303 | No | No | 2.875 | 1.946 | Yes |
| CHEMBL1233550 | NCCCC[C@H](N[C@@H](CCc1ccccc1)C(=O)O)C(=O)N1CCC[C@H]1C(=O)O | 405.495 | 1.2352 | 12 | 5 | 4 | 170.622 | -2.737 | 0.141 | 13.571 | -2.735 | Yes | No | No | -1.647 | 0.58 | -0.992 | -3.721 | No | No | No | No | No | No | No | 0.548 | No | No | 0.388 | No | No | 2.077 | 2.979 | Yes |
| CHEMBL3707320 | S=C(NCc1ccc2c(c1)OCO2)N1CCN(c2ncnc3c2oc2ccccc23)CC1 | 447.52 | 3.3014 | 3 | 7 | 1 | 189.284 | -3.633 | 1.345 | 90.85 | -2.771 | Yes | Yes | Yes | 0.343 | 0.036 | -0.554 | -2.252 | No | Yes | Yes | Yes | Yes | No | Yes | -0.056 | No | No | -0.008 | No | Yes | 2.312 | 0.545 | Yes |
| CHEMBL3989909 | CC1(C)CN(C(=O)c2ccc(-c3cccc4nc(NC(=O)C5CC5)nn34)cc2)C1 | 389.459 | 3.2268 | 4 | 5 | 1 | 168.285 | -3.672 | 1.014 | 95.21 | -2.952 | Yes | Yes | Yes | 0.519 | 0.164 | -0.364 | -2.905 | No | Yes | Yes | Yes | Yes | No | Yes | 0.144 | No | No | -0.575 | No | Yes | 3.064 | 0.304 | Yes |
| CHEMBL4778541 | O=C(O)c1cc2cc(Cc3cccnc3)ccc2o1 | 253.257 | 3.1168 | 3 | 3 | 1 | 108.87 | -3.075 | 0.873 | 94.706 | -2.609 | No | No | No | -0.458 | 0.174 | 0.168 | -2.127 | No | Yes | No | No | No | No | No | 0.775 | No | Yes | -0.278 | No | No | 2.507 | 1.946 | Yes |
| CHEMBL1779710 | CC(C)[C@H](N)C(=O)OCCOCn1cnc2c(=O)nc(N)[nH]c21 | 324.341 | -0.7976 | 7 | 9 | 3 | 131.341 | -2.629 | 0.278 | 44.23 | -2.735 | Yes | No | No | -0.499 | 0.754 | -1.38 | -3.925 | No | No | No | No | No | No | No | 0.803 | No | No | -0.055 | No | No | 2.285 | 2.585 | Yes |
| CHEMBL1237 | CC(C)NNC(=O)c1ccncc1 | 179.223 | 0.7244 | 3 | 3 | 2 | 77.342 | -1.704 | 1.274 | 76.025 | -2.958 | No | No | No | -0.444 | 0.499 | -0.221 | -3.318 | No | No | No | No | No | No | No | 0.868 | No | No | 1.222 | No | No | 2.309 | 2.753 | No |
| CHEMBL2103851 | Cc1c(F)cc(C(=O)NC2CC2)cc1-c1ccc(C(=O)NCC(C)(C)C)cn1 | 383.467 | 3.86422 | 5 | 3 | 2 | 164.305 | -4.968 | 1.183 | 92.362 | -3.18 | Yes | Yes | Yes | -0.005 | 0.104 | -0.221 | -2.203 | No | Yes | No | Yes | Yes | No | Yes | 0.629 | No | No | 0.141 | No | No | 2.332 | 1.214 | Yes |
| CHEMBL3301606 | CCCc1nc(C)c2c(=O)nc(-c3cc(S(=O)(=O)N4CCN(CC)CC4)ccc3OCC)[nH]n12.Cl.Cl | 561.536 | 2.91402 | 8 | 8 | 1 | 223.469 | -3.005 | 1.163 | 73.836 | -2.735 | Yes | No | No | 0.78 | 0.401 | -1.262 | -3.32 | No | No | No | No | No | No | Yes | 0.704 | Yes | Yes | 0.345 | No | No | 2.267 | 1.712 | Yes |
| CHEMBL1204948 | NS(=O)(=O)OC[C@@H]1C[C@@H](n2ccc3c(N[C@H]4CCc5ccccc54)ncnc32)C[C@@H]1O | 443.529 | 2.0628 | 6 | 8 | 3 | 180.069 | -3.582 | 0.42 | 88.963 | -2.859 | Yes | Yes | Yes | -0.247 | 0.188 | -1.385 | -3.525 | No | Yes | No | No | No | No | Yes | 0.751 | No | No | -0.501 | No | No | 2.588 | 1.403 | Yes |
| CHEMBL1349 | N=C(N)c1ccc(OCCCCCOc2ccc(C(=N)N)cc2)cc1 | 340.427 | 2.88284 | 10 | 4 | 4 | 147.321 | -3.553 | 0.873 | 77.098 | -2.793 | Yes | No | No | 0.135 | 0.106 | -0.914 | -2.929 | No | Yes | No | Yes | Yes | No | Yes | 0.871 | Yes | No | 0.111 | No | Yes | 2.564 | 2.356 | No |
| CHEMBL92401 | CCOC(=O)[C@H](CCc1ccccc1)N[C@@H](C)C(=O)N1C(=O)N(C)C[C@H]1C(=O)O | 405.451 | 0.8762 | 9 | 6 | 2 | 169.158 | -2.739 | 0.288 | 30.7 | -2.735 | Yes | No | No | -0.714 | 0.778 | -0.734 | -3.609 | No | No | No | No | No | No | No | 0.699 | No | No | 0.955 | No | No | 1.679 | 2.597 | Yes |
| CHEMBL1088752 | CCN(CC)CCNC(=O)c1c(C)[nH]c(/C=C2\C(=O)Nc3ccc(F)cc32)c1C | 398.482 | 3.33494 | 7 | 3 | 3 | 169.73 | -3.653 | 1.053 | 89.86 | -3.043 | Yes | Yes | Yes | 0.826 | 0.137 | -0.897 | -2.553 | Yes | Yes | No | No | No | No | Yes | 0.942 | No | No | -0.226 | No | Yes | 2.621 | 0.903 | Yes |
| CHEMBL2106480 | O=C(O)CNC(=O)c1ncc(-c2cccc(Cl)c2)cc1O | 306.705 | 1.922 | 4 | 4 | 3 | 124.568 | -2.805 | 0.131 | 52.497 | -2.72 | No | No | No | -0.495 | 0.271 | -1.185 | -2.879 | No | No | No | No | No | No | No | 0.012 | No | No | 0.71 | No | No | 1.861 | 2.715 | Yes |
| CHEMBL1231160 | CS(=O)(=O)O.Cc1cc(C(=O)Nc2nc3cccc(Cl)c3n2[C@@H]2CCCCN(C(=O)/C=C/CN(C)C)C2)ccn1.O.O.O | 645.179 | 1.34672 | 6 | 8 | 2 | 255.58 | -2.948 | -0.544 | 31.864 | -2.735 | Yes | No | No | -0.021 | 0.321 | -1.453 | -3.312 | No | No | No | No | No | No | No | 0.224 | No | No | 0.585 | No | No | 2.357 | 1.966 | Yes |
| CHEMBL55 | Cc1cc(C(=O)Nc2nc3cccc(Cl)c3n2[C@@H]2CCCCN(C(=O)/C=C/CN(C)C)C2)ccn1 | 495.027 | 4.31682 | 6 | 6 | 1 | 210.138 | -3.17 | 1.177 | 85.362 | -2.735 | Yes | Yes | No | -0.024 | 0.234 | -0.937 | -2.902 | No | No | No | No | No | No | Yes | 0.5 | Yes | Yes | 0.407 | No | Yes | 2.322 | 1.161 | Yes |
| CHEMBL317094 | C[C@H]1CO[C@@H]2Cn3cc(C(=O)NCc4ccc(F)cc4F)c(=O)c([O-])c3C(=O)N12.[Na+] | 427.339 | -2.6653 | 3 | 6 | 1 | 191.273 | -2.997 | 1.126 | 63.111 | -2.891 | Yes | No | No | -0.659 | 0.246 | -1.031 | -3.229 | No | Yes | No | No | No | No | No | 0.701 | No | No | 0.183 | No | No | 1.825 | 1.687 | Yes |
| CHEMBL2110816 | COc1ccc([N-]S(=O)(=O)c2c(F)c(F)c(F)c(F)c2F)cc1F.[Na+] | 393.24 | 0.9279 | 4 | 3 | 0 | 159.684 | -3.583 | 2.022 | 100 | -2.735 | No | Yes | No | -0.758 | 0.261 | 0.107 | -3.548 | No | Yes | No | No | No | No | No | -0.04 | No | No | 0.958 | No | No | 2.654 | 1.456 | Yes |
| CHEMBL535 | Cc1nnc(C(=O)NC(C)(C)c2nc(C(=O)NCc3ccc(F)cc3)c(O)c(=O)n2C)o1 | 444.423 | 0.91152 | 6 | 9 | 3 | 180.861 | -3.312 | 0.653 | 67.374 | -2.735 | Yes | No | No | -0.787 | 0.069 | -1.581 | -3.336 | No | No | No | No | No | No | No | 0.277 | No | No | 0.846 | No | Yes | 1.922 | 2.064 | Yes |
| CHEMBL3646221 | Cc1ccc(Nc2c(F)cccc2Cl)c(CC(=O)O)c1 | 293.725 | 4.15822 | 4 | 2 | 2 | 120.558 | -3.777 | 1.33 | 92.136 | -2.735 | Yes | No | No | -1.222 | 0.117 | -0.053 | -1.954 | No | No | No | No | No | No | No | 0.165 | No | No | 1.111 | No | No | 2.591 | 2.071 | No |
| CHEMBL4297222 | O=C(O)CCCCCCNC1c2ccccc2CCc2ccccc21 | 337.463 | 4.4993 | 8 | 2 | 2 | 149.638 | -3.448 | 0.994 | 92.431 | -2.722 | Yes | No | No | 1.01 | 0.179 | -0.03 | -2.082 | No | Yes | Yes | No | No | No | No | 0.922 | No | No | 0.001 | No | Yes | 2.797 | 2.155 | Yes |
| CHEMBL3787344 | C[C@H](Nc1ccc2c(c1)OCCn1cc(N3C(=O)OC[C@H]3C(F)F)nc1-2)C(N)=O | 407.377 | 1.8185 | 5 | 7 | 2 | 163.931 | -2.994 | 0.785 | 86.745 | -2.735 | Yes | No | No | 0.242 | 0.342 | -1.304 | -3.019 | No | Yes | No | No | No | No | No | 0.564 | Yes | Yes | 0.044 | No | No | 2.674 | 0.559 | Yes |
| CHEMBL3137330 | O=C1[C@H](CC[C@H](O)c2ccc(F)cc2)[C@@H](c2ccc(O)cc2)N1c1ccc(F)cc1 | 409.432 | 4.8883 | 6 | 3 | 2 | 172.57 | -4.799 | 1.163 | 92.535 | -2.738 | Yes | Yes | Yes | -0.309 | 0.22 | -0.612 | -2.067 | No | Yes | No | Yes | Yes | No | Yes | -0.219 | No | No | 0.553 | No | Yes | 2.399 | 1.536 | Yes |
| CHEMBL2105902 | C[C@@H](N)COc1ccc(-c2cnc3ccc(N[C@H](C)c4cccc(F)c4)nn23)cc1 | 405.477 | 4.4345 | 7 | 6 | 2 | 174.065 | -2.955 | 1.188 | 87.605 | -2.735 | Yes | Yes | Yes | -0.078 | 0.039 | -0.482 | -2.294 | No | Yes | Yes | Yes | Yes | No | Yes | 0.737 | Yes | Yes | 0.119 | No | Yes | 2.417 | 0.364 | Yes |
| CHEMBL254316 | C=CC(=O)Nc1cccc(Oc2nc(Nc3ccc(N4CCN(C)CC4)cc3)nc3ccoc23)c1 | 470.533 | 4.635 | 7 | 8 | 2 | 202.579 | -3.988 | 1.51 | 96.599 | -2.74 | Yes | Yes | Yes | 1.035 | 0.08 | -1.196 | -2.529 | No | Yes | Yes | No | Yes | No | Yes | 0.634 | Yes | No | 0.4 | No | Yes | 3.514 | 0.945 | Yes |
| CHEMBL404108 | NC(=O)NC[C@@H]1CCCc2cc(S(=O)(=O)c3cccc(F)c3)ccc21 | 362.426 | 2.7467 | 4 | 3 | 2 | 145.515 | -4.448 | 1.282 | 95.559 | -2.744 | Yes | Yes | No | -0.552 | 0.03 | -0.414 | -2.463 | No | Yes | No | Yes | No | No | No | 0.527 | No | Yes | 0.051 | No | Yes | 2.544 | 1.408 | Yes |
| CHEMBL418995 | O=c1[nH]cnc2c1ncn2[C@H]1CC[C@@H](CO)O1 | 236.231 | -0.2105 | 2 | 6 | 2 | 96.079 | -2.218 | -0.163 | 75.647 | -2.735 | Yes | No | No | -0.202 | 0.632 | -1.325 | -3.572 | No | No | No | No | No | No | No | 0.928 | No | Yes | 0.622 | No | No | 2.557 | 1.25 | No |
| CHEMBL4650215 | NS(=O)(=O)Oc1ccc2c3c(c(=O)oc2c1)CCCCC3 | 309.343 | 1.6442 | 2 | 5 | 1 | 121.322 | -3.225 | 0.015 | 88.004 | -2.764 | No | No | No | -0.477 | 0.119 | -0.784 | -2.642 | No | Yes | Yes | Yes | No | No | No | 0.926 | No | No | 0.31 | No | No | 2.791 | 0.949 | Yes |
| CHEMBL1138 | CCC[C@H](N[C@@H](C)C(=O)N1[C@H](C(=O)O)C[C@@H]2CCCC[C@@H]21)C(=O)OCC.N=C(N)NCCC[C@H](N)C(=O)O | 542.678 | 0.60217 | 13 | 8 | 7 | 224.065 | -2.826 | -0.54 | 0 | -2.735 | Yes | No | No | -0.862 | 0.855 | -1.74 | -4.359 | No | Yes | No | No | No | No | No | 0.362 | No | No | 0.69 | No | No | 2.116 | 2.763 | Yes |
| CHEMBL4650989 | C[C@@H](Nc1nc(N[C@H](C)C(F)(F)F)nc(-c2cccc(Cl)n2)n1)C(F)(F)F | 414.741 | 4.3124 | 5 | 6 | 2 | 153.953 | -6.241 | 0.737 | 87.615 | -3.13 | No | No | No | -0.651 | 0.346 | -1.668 | -3.155 | No | No | Yes | Yes | No | No | No | 0.209 | No | No | 0.266 | No | No | 2.728 | -0.135 | Yes |
| CHEMBL4163691 | CC(C)c1nc(N(C)S(C)(=O)=O)nc(-c2ccc(F)cc2)c1/C=C/[C@@H](O)C[C@@H](O)CC(=O)O | 481.546 | 2.4017 | 10 | 7 | 3 | 191.848 | -3.43 | -0.602 | 50.125 | -2.735 | Yes | No | No | -1.158 | 0.131 | -1.945 | -3.778 | No | No | No | No | No | No | No | 0.925 | No | No | 0.017 | No | No | 2.497 | 2.816 | Yes |
| CHEMBL1082508 | CNC(=O)Oc1ccc2c(c1)[C@]1(C)CCN(C)[C@@H]1N2C.CNC(=O)Oc1ccc2c(c1)[C@]1(C)CCN(C)[C@@H]1N2C.O=S(=O)(O)O | 648.783 | 2.895 | 2 | 10 | 4 | 264.367 | -3.073 | -0.534 | 30.291 | -2.735 | Yes | No | No | -0.342 | 0.224 | -1.475 | -3.475 | No | Yes | No | No | No | No | No | -0.31 | No | No | 0.102 | No | No | 2.23 | 2.181 | No |
| CHEMBL1460 | CN(C)CC(c1ccc(O)cc1)C1(O)CCCCC1.O=C(O)CCC(=O)O | 381.469 | 2.6684 | 7 | 5 | 4 | 159.8 | -2.69 | 0.247 | 27.339 | -2.735 | Yes | No | No | -0.34 | 0.53 | -1.196 | -3.212 | No | No | No | No | No | No | No | 0.542 | No | No | 0.75 | No | No | 1.951 | 2.457 | Yes |
| CHEMBL286738 | CN(C)CC(c1ccc(O)cc1)C1(O)CCCCC1 | 263.381 | 2.7326 | 4 | 3 | 2 | 115.423 | -3.062 | 1.224 | 92.131 | -3.174 | Yes | No | No | 1.078 | 0.492 | 0.048 | -2.334 | Yes | No | No | No | No | No | No | 0.821 | No | No | -0.565 | No | No | 2.476 | 1.378 | No |
| CHEMBL3544986 | CCOC(=O)[C@H](CCc1ccccc1)N[C@@H](C)C(=O)N(CC(=O)O)C1Cc2ccccc2C1 | 452.551 | 2.6096 | 11 | 5 | 2 | 194.294 | -3.171 | 0.05 | 49.348 | -2.735 | Yes | No | No | -0.125 | 0.387 | -0.591 | -3.095 | No | Yes | No | No | No | No | No | 0.903 | No | No | -0.261 | No | No | 2.17 | 2.087 | Yes |
| CHEMBL4279047 | CCCOc1ccc([C@H]2CNC(=O)N2c2ccc3nc[nH]c3c2)cc1 | 336.395 | 3.6226 | 5 | 3 | 2 | 145.368 | -3.051 | 1.12 | 84.581 | -2.735 | Yes | Yes | Yes | -0.021 | 0.022 | -0.835 | -2.387 | No | No | Yes | Yes | No | No | Yes | 1.098 | Yes | Yes | 0.087 | No | Yes | 2.227 | 1.319 | Yes |
| CHEMBL1496 | CN(C)C(=O)Oc1cccc([N+](C)(C)C)c1.[Br-] | 303.2 | -1.0523 | 2 | 2 | 0 | 113.138 | -1.132 | 1.528 | 93.141 | -2.749 | No | No | No | 0.066 | 0.726 | 0.157 | -1.959 | No | No | No | No | No | No | No | 0.979 | No | No | 0.382 | No | No | 2.958 | 2.358 | No |
| CHEMBL2105891 | O.O=C(NC1CCNCC1)[C@@H]1CC[C@@H]2CN1C(=O)N2OS(=O)(=O)O | 366.396 | -1.9671 | 4 | 6 | 3 | 139.065 | -2.378 | -0.545 | 9.677 | -2.735 | No | No | No | -0.595 | 0.837 | -1.263 | -3.487 | No | No | No | No | No | No | No | 0.908 | No | No | 1.106 | No | No | 1.506 | 2.496 | Yes |
| CHEMBL94 | C=CC(=O)Nc1cc(Nc2nccc(-c3cn(C)c4ccccc34)n2)c(OC)cc1N(C)CCN(C)C | 499.619 | 4.5098 | 10 | 8 | 2 | 216.94 | -4.215 | 0.867 | 95.619 | -2.736 | Yes | Yes | Yes | 1.312 | 0.204 | -1.248 | -2.627 | No | Yes | No | No | Yes | No | Yes | 0.545 | Yes | No | 0.568 | No | Yes | 3.067 | 1.262 | Yes |
| CHEMBL1201728 | N#C[C@@H]1C[C@@H]2C[C@@H]2N1C(=O)[C@@H](N)C12CC3CC(CC(O)(C3)C1)C2 | 315.417 | 1.15798 | 2 | 4 | 2 | 136.362 | -3.462 | 0.194 | 60.938 | -2.792 | Yes | No | No | 0.631 | 0.436 | -0.53 | -3.067 | No | No | No | No | No | No | No | 0.892 | No | No | -0.658 | No | No | 2.539 | 1.002 | No |
[truncated: 21,568 more chars]
